# The Nonadiabatic Nature of the Substituent Effects in Azobenzene

**DOI:** 10.1002/anie.202523613

**Published:** 2026-02-20

**Authors:** Jacob Jan van der Wal, Roman Yu. Peshkov, Jorn D. Steen, Nadja A. Simeth, Stefano Crespi

**Affiliations:** ^1^ Department of Chemistry – Ångström Laboratory Uppsala University Uppsala Sweden; ^2^ Institute For Organic and Biomolecular Chemistry Department of Chemistry University of Göttingen Göttingen Germany; ^3^ Cluster of Excellence “Multiscale Bioimaging: from Molecular Machines to Networks of Excitable Cells” (MBExC) University of Göttingen Göttingen Germany

**Keywords:** Azobenzene, Isomerization, Linear free energy relationships, Reaction mechanisms, Thermochemistry

## Abstract

The mechanism of thermal *Z* → *E* isomerization in azobenzenes has been debated for nearly a century, with inversion, rotation, and nonadiabatic pathways proposed to account for the nonlinear substituent dependence of the reaction rate. Here, we combine systematic kinetic analysis with temperature‐dependent Eyring and isokinetic evaluations to experimentally evaluate the origin of this behavior. A series of *para*‐substituted azobenzenes exhibits uniformly negative entropies of activation, suggesting a single nonadiabatic rotational mechanism is operative across all substituents. We found that the characteristic “V‐shaped” Hammett correlation of azobenzene arises not from a mechanistic change, but from the inadequacy of the *σ_p_* scale to describe the stabilization of the open‐shell, diradicaloid species involved in the nonadiabatic pathway. The Creary *σ·* radical parameter restores linearity, confirming that both electron‐donating and electron‐withdrawing substituents increase the reaction rate, stabilizing the diradicaloid species. Complementary calculations using different multireference spin‐flip and single‐reference approaches reproduce the experimental trends and support the predominance of the nonadiabatic pathway, whereas density functional theory (DFT) systematically fails to reproduce these trends.

## Graphical abstract

**Unified nonadiabatic switching**: thermal *Z*→*E* isomerization of *para*‐substituted azobenzenes proceeds through a single nonadiabatic rotational pathway. The iconic bell‐shaped Hammett plot is a σ_p_ artifact, not a reflection of a mechanistic change, exposing the limits of single‐reference density functional theory (DFT) and establishing activation entropy as the key diagnostic for azo photoswitch design.

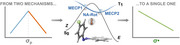

## 1 Introduction

Molecular photoswitches are compounds that can reversibly interconvert between distinct isomeric states upon irradiation of one or both isomers, enabling optical control over their physical and chemical properties. Among these, azobenzene derivatives represent one of the most established and versatile classes, widely utilized across diverse scientific disciplines owing to their synthetic accessibility and the remarkable tunability of their photophysical and photochemical properties [1, 2, 3, 4, 5, 6, 7, 8]. The suitability of a given azobenzene for specific applications is primarily dictated by the thermal lifetime (*τ*) of its metastable isomer, which can be systematically tuned through targeted structural modifications, such as the introduction of tetra‐*ortho*‐fluoro substituents or heteroaryl units [9, 10, 11]. Remarkably, despite the development of numerous strategies to control this parameter, the fundamental mechanism underlying the thermal isomerization of azobenzenes has remained a topic of active debate for nearly 90 years, ever since Hartley first reported the process in the late 1930s [12, 13].

One of the most intriguing early observations was the pronounced non‐linearity of substituent effects on the rates of thermal isomerization in substituted azobenzenes relative to the widely used Hammett *σ_p_* scale [14, 15, 16], obtaining a “V‐” or “bell‐shaped” Hammett plot. Studies from the late 1950s and 1960s already revealed that both electron‐withdrawing and electron‐donating substituents within a given azobenzene series tend to accelerate the thermal isomerization process [14, 15]. This trend extends beyond classical azobenzenes to heteroaryl derivatives such as azopyrazoles, as recently demonstrated by us and others [17, 18, 19, 20]. These observations have long suggested that substituent effects in azobenzenes cannot be rationalized within a single transition state (TS) framework, but necessitate a more complex solution.

**FIGURE 1:**
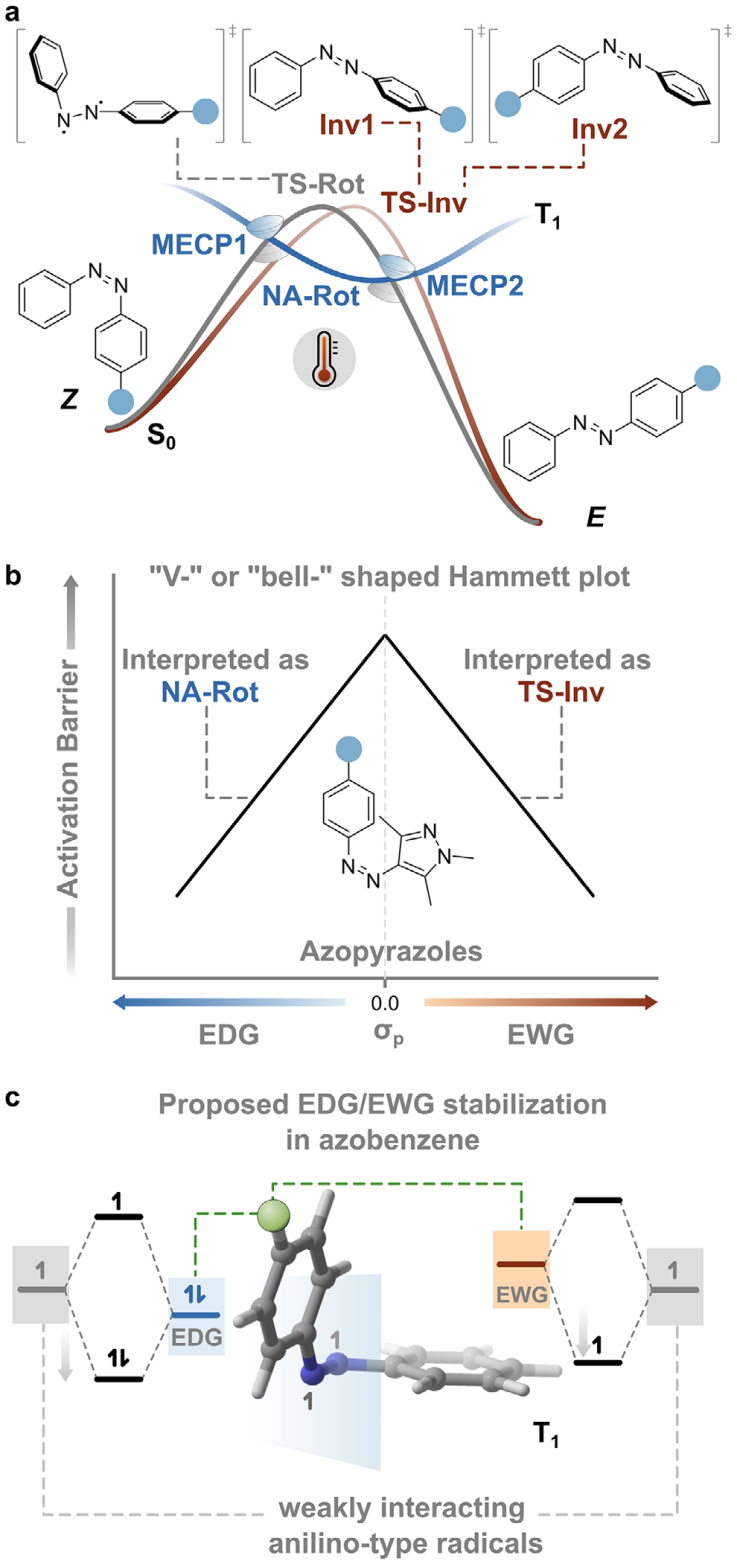
(a) Pictorial representation of the different thermal isomerization paths of the *Z*‐form of monosubstituted azobenzenes (Rotation, TS‐Rot; Inversion, TS‐Inv; nonadiabatic rotation, NA‐Rot). The two possible inversion transition states are highlighted as TS‐Inv1 and TS‐Inv2. MECP stands for Minimum Energy Crossing Point. (b) Conceptual illustration of the characteristic “V‐” or “bell‐shaped” substituent dependence observed in Hammett‐type analyses of azobenzene thermal isomerization, using the recent work on azopyrazoles [17] as an example of a change in mechanism between nonadiabatic rotation and inversion. (c) Qualitative depiction of the investigated open‐shell electronic structure of T_1_ of azobenzene, accessed near the nonadiabatic crossing region, where the diradicaloid character of azobenzene resembles two weakly interacting anilino‐type radicals. Both electron‐donating and electron‐withdrawing substituents can stabilize partially occupied orbitals, providing a rationale for the non‐linear substituent dependency in relation to the Hammett‐Taft parameter.

When restricting the discussion to solvents and systems in which only a monomolecular isomerization pathway is accessible, thus excluding mechanisms involving hydrazone intermediates or the stabilization of quinoidal or push‐pull structures in highly polar media [21], the thermal isomerization of azobenzenes has historically been described in terms of two possible transition states (TSs): one involving inversion at a nitrogen atom without rupture of the N═N bond (TS‐Inv, Figure 1a), and the other one corresponding to rotation about the N═N bond (TS‐Rot, Figure 1a), accompanied by cleavage of the double bond. Early research initially supported a rotation‐based mechanism [22, 23], which was later rejected for energetic reasons [14] in favor of an inversion mechanism. Over the following decades, numerous studies alternately supported one pathway or the other based on different experimental findings, including solvent‐transfer enthalpies [24], solvent and substituent effects [15, 25, 26, 27, 28], changes in the activation volume under elevated pressures [25, 29, 30, 31], and investigations in systems with geometrical constraints, under confinement [32, 33, 34] or in liquid‐crystalline environments [35, 36].

In monosubstituted azobenzenes, as examined in this work, the inversion transition state can adopt two distinct forms depending on whether the substituent is attached to the phenyl ring whose nitrogen atom rehybridizes to a linear *sp*‐configuration (TS‐Inv1, Figure 1a) or to the opposite, *sp^2^*‐retaining ring (TS‐Inv2; in the text we will refer as *TS‐Inv* when both Inv1 and Inv2 will be taken together into consideration as a sum of rates). Electron‐withdrawing groups preferentially stabilize the former, while the latter is favored by electron‐donating substituents, a phenomenon long considered responsible for the nonlinear Hammett correlation in azobenzenes [14, 15]. However, discrepancies emerge when activation parameters are analyzed in detail [14, 33, 34]. In particular, DFT calculations often reproduce experimental rate constants for thermal isomerization but fail to capture the experimentally observed negative entropies of activation.

For parent azobenzene, this so‐called *“entropy puzzle”* was solved by Hecht and co‐workers through combined experimental and computational studies, which identified a third, nonadiabatic pathway involving ultrafast S_0_‐T_1_‐S_0_ crossings (minimum energy crossing points, MECPs, Figure 1a) along the rotational coordinate (NA‐Rot, Figure 1a). This mechanism, first hypothesized by Eyring in 1941 [37] and later discussed by others [23, 38], rationalizes the negative entropies of activation as arising from a transmission coefficient (γ) lower than unity in the Eyring equation (Section 3, Supporting Information) [39].

Recent studies have extended this discussion to heteroaryl derivatives of azobenzenes, particularly azopyrazoles [18, 40]. González and co‐workers proposed that the dominant thermal relaxation mechanism depends on the para substituent on the phenyl ring, with the apparent rates representing weighted averages of triplet‐assisted rotational and inversion pathways (see Figure 1b) [17, 41]. In this framework, electron‐withdrawing groups favor the inversion component, accounting for the nonlinear substituent dependence in Hammett plots constructed against *σ*_p_. However, these analyses were primarily based on rate constants measured at a single temperature and did not explicitly consider activation parameters, leaving unresolved the question of whether substituent effects reflect genuine mechanistic competition or instead arise from a common underlying pathway. This ambiguity is even more pronounced when considering the minimum‐energy crossing points (MECPs) between singlet and triplet surfaces (Figure 1a). In the region close to these points, as well as on the triplet surface, the two N‐aryl moieties are nearly orthogonal: the electronic structure can be described as two weakly interacting anilino‐type radicals. In this regime, both electron‐donating and electron‐withdrawing substituents can stabilize the partially occupied orbitals, naturally accounting for the experimentally observed nonlinear substituent dependence.

Thus, qualitative depiction suggests that substituent effects may be governed by stabilization of open‐shell electronic structures accessed along the nonadiabatic pathway, and motivated us to experimentally and computationally test whether such effects are reflected in the thermodynamic activation parameters of the thermal *Z* → *E* isomerization.

## 2 Results and Discussion

We synthesized the target azobenzenes from nitrosobenzene via a Baeyer‐Mills reaction to afford a series of molecules with substituents with different electron demand (Section 1, Supporting Information and Table 1). The thermal *Z → E* isomerization kinetics were investigated by measuring rate constants at five different temperatures in triplicate (Table 1; Section 4, Supporting Information). Because of the relatively slow thermal relaxation of azobenzene, toluene was chosen as the solvent for its higher boiling point, enabling Eyring analysis over a broader temperature range (from 303 to 363 K, Sections 3–6, Supporting Information). The substituents representing the extremes in the *σ*_p_ values (NO_2_, NMe_2_) and the parent azobenzene were also studied under argon‐sparged conditions to assess the influence of oxygen on the relaxation rate. Linearized Eyring plots (Section 5, Supporting Information) yielded the activation parameters Δ*G* ^‡^, Δ*H* ^‡^, and Δ*S* ^‡^ for each compound (Table 1). The obtained activation parameters were used to construct relationships (Section 6, Supporting Information) with multiple substituent parameters to test the mechanistic hypothesis, and to construct Exner plots (Section 7, Supporting Information) based on rates at various temperatures.

**TABLE 1:** Overview of the experimentally obtained rates and activation parameters alongside the respective Hammett‐Taft and Creary parameters for each substituent.

Substituent	*σ*_p_ ^a^	*σ*·^b^	Rate (s^−1^)^c^	*τ* (h)	*t*_1/2_ (h)^d^	Δ*G* ^‡^ (kJ/mol)^c^	Δ*H* ^‡^ (kJ/mol)^c^	Δ*S* ^‡^ (J/mol K)	*ΔS* ^‡^ (comp.)^e^ (J/mol K)
NO_2_	0.78	0.57	4.16 x 10^−5^	7	5	98.0 ± 0.2	92 ± 2	−22 ± 7	−29
NO_2_ (Ar)	0.78	0.57	3.99 x 10^−5^	7	5	98.1 ± 0.2	91 ± 3	−24 ± 8	
CN	0.66	0.46	5.99 x 10^−5^	16	32	102.8 ± 0.2	101 ± 2	−5 ± 5	−28
CF_3_	0.54	0.08	1.58 x 10^−6^	176	122	106.1 ± 0.2	102 ± 1	−14 ± 4	−16
Cl	0.23	0.12	2.37 x 10^−6^	117	81	105.1 ± 0.1	97 ± 1	−27 ± 4	−30
Br	0.23	0.13	2.32 x 10^−6^	120	83	105.15 ± 0.06	95.2 ± 0.5	−33 ± 1	−30
I	0.18	n/a	1.94 x 10^−6^	144	99	105.6 ± 0.1	97.4 ± 0.7	−28 ± 2	−30
F	0.06	−0.08	1.67 x 10^−6^	167	115	105.97 ± 0.08	97.2 ± 0.9	−30 ± 3	−30
H	0	0	1.52 x 10^−6^	183	127	106.2 ± 0.1	97 ± 1	−32 ± 4	−30
H (Ar)	0	0	1.40 x 10^−6^	198	137	106.4 ± 0.1	98.0 ± 0.8	−28 ± 2	
*t*Bu	−0.2	0.13	2.81 x 10^−6^	99	69	104.68 ± 0.09	95.5 ± 0.8	−31 ± 2	−31
OMe	−0.27	0.24	4.30 x 10^−6^	65	45	103.62 ± 0.07	98.3 ± 0.9	−18 ± 3	−30
NMe_2_	−0.83	0.9	5.27 x 10^−5^	5	4	97.41 ± 0.04	85.0 ± 0.4	−42 ± 1	−43
NMe_2_ (Ar)	−0.83	0.9	4.42 x 10^−5^	6	4	97.85 ± 0.09	86.1 ± 0.9	−39 ± 3	

^a^ Hammett‐Taft parameters from Ref [16].

^b^ Creary parameters from Ref [42].

^c^ Values at 298 K.

^d^ Half‐life.

^e^ Values computed at the XMS‐CASPT2/ANO‐RCC‐VTZP//ωB97X‐3c/SMD(toluene) level.

We observed that replacing the hydrogen with any electron‐donating or electron‐withdrawing substituent leads to larger rate constants (Table 1; Section 6 in Supporting Information, and Figure S51), as previously noted by Talaty and Nishimura [14, 15]. This result is reflected in the lifetime, *τ*; the effect is most pronounced in the strongest electron‐donating and electron‐withdrawing substituents (NO_2_ *τ* = 7 h, and NMe_2_ *τ* = 5 h vs. H *τ* = 183 h). To probe the intermediacy of long‐lived triplet states, we measured the rates for –NO_2_, –NMe_2_, and unsubstituted azobenzenes in argon‐sparged solutions. We observed no significant deviation from ambient conditions, excluding any measurable effect of oxygen on the rate constants of the azobenzenes explored in this study (Table 1; Section 6, Supporting Information).

**FIGURE 2:**
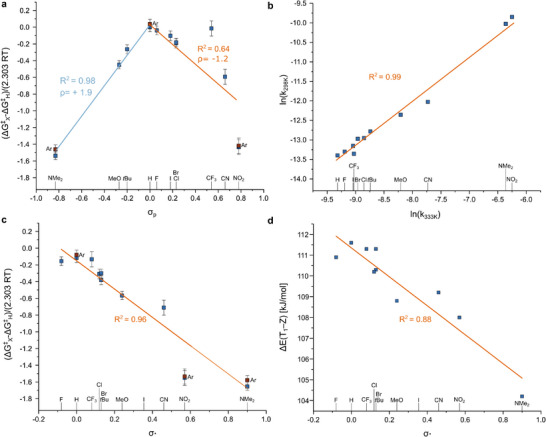
(a) Hammett plot of the experimentally obtained Δ*G* ^‡^ at 298 K vs. the Hammett–Taft parameter, where the linear correlation for electron‐donating groups is depicted in orange (*R* ^2^ = 0.98, ρ = +1.9), and the linear correlation for the electron‐withdrawing groups is depicted in green (*R* ^2^ = 0.64, ρ = ‐1.2); Ar indicates argon sparged (these points were not included in the linear correlations). (b) Exner plot of the natural logarithm of the rate at 298 K against the rate at 333 K (*R* ^2^ = 0.99). (c) Hammett plot of the experimentally obtained *ΔG* ^‡^ at 298 K vs. the Creary parameter (*R* ^2^ = 0.96). (d) Plots of the computed triplet excitation energies (ΔE(T_1_–*Z*)) at the MRSF‐BH&HLYP/def2‐TZVP level plotted against the Creary parameter, showing a good linearity comparable to the experimental results.

Across the temperature range for each substituent, we obtained linear Eyring plots with no clear breaks and excellent correlations (*R* ^2^ = 0.99; Section 6, Supporting Information), suggesting no change in mechanism over this temperature range. Still, no information about the nature of the mechanism for each substituent could be obviously derived. We observed that *ΔG* ^‡^ decreases for any substituent on the azobenzene core (Table 1, and Figure 2a), consistent with a lower activation barrier and a faster thermal relaxation time. We plotted the change in Gibbs free energy as a function of the *σ*_p_ parameter and observed a “bell‐shaped” distribution with the maximum near zero (*σ*_p_ = H, Figure 2a). CF_3_ and NO_2_ deviate from the linearity observed in the electron‐withdrawing section of the Hammett parameter. The obtained ρ values for the linear correlations — ρ = +1.9, R^2^ = 0.98 and ρ = −1.2, R^2^ = 0.64 — are relatively steep, and in the case of the electron‐withdrawing substituent, moderately poor. This result would imply a significant charge buildup at the transition state during the *Z* → E isomerization, which is inconsistent with the relatively neutral nature of the transition states for the involved mechanisms in an apolar solvent such as toluene [39, 43].

Moreover, the measured ΔS^‡^ values are significantly negative across the entire substituent range, ranging from −5 to −42 J/(mol K) (Table 1). Hecht and Kaupp have previously highlighted this phenomenon as a strong indication of a nonadiabatic rotation mechanism, hinting that the same mechanism is operative across the entire substituent series [39]. Analysis of the plot of *ΔS* ^‡^ shows a linear correlation between the electronic nature of the substituent and the apparent *ΔS* ^‡^; a more negative value for *ΔS* ^‡^ is observed for stronger electron‐withdrawing groups (except for NO_2_, see Table 1 and Figure S53). For the 4‐methoxy, 4‐(trifluoromethyl), and 4‐cyano derivatives, a deviation is observed, where the *ΔS* ^‡^ is significantly less negative than the other compounds (Table 1). By plotting *ΔH* ^‡^ against *σ*_p_, we observe a similar trend as the one observed in *ΔS* ^‡^ (Figure S54).

Given the wide range of temperatures, we applied isokinetic analysis to our data and constructed Exner plots (Figure 2b; Section 7, Supporting Information). This analysis was already used to test the presence of a single mechanism in heteroaryl azobenzenes [44]. In our case, we report linear correlations (*R* ^2^ = 0.98‐0.99, isokinetic temperature β = 163 K) with every substituent on the line for all the measured temperatures. The linearity indicates that all compounds across the substituent series follow the same entropy‐enthalpy compensation, strongly suggesting that the same mechanism governs the thermal *Z* → *E* isomerization. This compensation is qualitatively evident by comparing Figures S53 and S54. We note that plotting *ΔH* ^‡^ against *ΔS* ^‡^ (Figure S55) does not yield a perfect correlation owing to the sensitivity of *ΔH* ^‡^ and *ΔS* ^‡^ to experimental errors, as in similar cases documented in the literature [45, 46].

Based on these findings, we concluded that the clear break observed in the Hammett plot may originate from limitations of the σ_p_ scale in describing open‐shell diradicaloid transition states, rather than from a change in mechanism, as previously proposed [17, 18, 20]. Consequently, we attempted to correlate our experimental data to different *σ* parameters [42, 47, 48, 49, 50, 51] (Section 6, Supporting Information). We focused on substituent scales specifically constructed for radical reactions (Figure 2c and 2d, and Section 6, Supporting Information) and obtained the best correlations using the *σ*· parameter from Creary [42, 47]. Following the same approach as described earlier, we plotted the ΔΔ*G* ^‡^, ΔΔ*H* ^‡^, ΔΔ*S* ^‡^, and log(*k/k*_0_) (Figure 2c and Section 6, Supporting Information), finding excellent correlation for ΔΔ*G* ^‡^ (*R* ^2^ = 0.95). While ΔΔ*H* ^‡^ shows a moderate systematic dependence on σ·, the ΔΔ*S* ^‡^ values cluster around a common negative value for most substituents, such that the apparent scatter and low formal correlation largely reflect limited substituent sensitivity and statistical uncertainty rather than distinct mechanisms (see Figures S53 and S54). This data further supports our hypothesis that a single mechanism governs the series and that the break in the Hammett plot observed across the substituent series using *σ*_p_ is attributable to the choice of substituent parameter rather than to a change in mechanism.

To further rationalize the experimentally observed substituent effects, we examined the dependence of the computed triplet excitation energies (*ΔE*(T_1_–Z)) on substituent parameters. Figure 2d shows the triplet energies of *para*‐substituted azobenzenes calculated at the MRSF‐BH&HLYP level plotted against Creary σ· constants. Despite its simplicity, this analysis reveals a clear and systematic substituent dependence (*R* ^2^ = 0.88), indicating that both electron‐donating and electron‐withdrawing groups stabilize the triplet state relative to the *Z* isomer. This observation supports the view that substituent effects in azobenzenes are governed by stabilization of open‐shell electronic structures accessed along the rotational coordinate and highlights the relevance of radical‐specific substituent descriptors. The limited scope of this correlation underscores the need for a more detailed and quantitative treatment of the full reaction pathways, motivating the comprehensive benchmarking presented below.

We consequently performed a systematic computational analysis of the inversion, adiabatic rotational, and nonadiabatic rotational pathways to complement the experimental investigation and assess the mechanistic origin of the thermal Z → *E* isomerization. Particular care was taken to address the well‐known multireference character of azobenzene in the region of N═N rotation and near the *S*_0_/*T*_1_ crossing seam, where conventional single‐reference approaches become unreliable (Section 8.1.5, Supporting Information). Geometry optimizations were benchmarked across several density‐functional approximations against CASSCF reference structures (see Section 8.2, Supporting Information). Among the tested methods, (broken–symmetry) ωB97X‐3c [52] provided the best results, reproducing CASSCF geometries of minima, transition states, and MECPs with minor structural deviations. This level was therefore used consistently to generate geometries and thermal corrections across the substituent series, ensuring a uniform structural reference.

We evaluated electronic energies using methods designed to describe near‐degenerate and open‐shell singlet electronic structures (see Section 8.2, Supporting Information). In particular, we employed spin‐flip TDDFT (SF‐TDDFT), the more recent mixed‐reference spin‐flip TDDFT (MRSF‐TDDFT) [53], and multireference perturbative approaches (CASPT2 and XMS‐CASPT2). We compared these results to single‐reference DLPNO‐(U)CCSD(T) and to DFT. Spin‐flip methods (without and with implicit solvation) were used with BH&HLYP, following established benchmarks for azobenzene [54], and spin contamination was carefully monitored to ensure consistent state assignment (see Section 8.3, Supporting Information). We chose BH&HLYP and ωB97X‐D4 for the calculations with MRSF‐TDDFT, while XMS‐CASPT2 was employed to obtain a balanced multistate description of the crossing region, particularly for strongly electron‐withdrawing substituents (see Section 8.3.2, Supporting Information).

Benchmarking of electronic activation energies against literature reference data (Kaupp et al., [39], Wu et al., [40].) reveals a consistent mechanistic picture across all methods that properly account for near‐degeneracy (see Section 8.3.4, Supporting Information). Except for DFT, all approaches place the MECPs below the adiabatic rotational and inversion transition states, identifying nonadiabatic rotation as the energetically preferred pathway. Spin‐flip TDDFT reproduces inversion barriers and MECP energies in close agreement with reference values, while XMS‐CASPT2 provides a robust description of the crossing region (see Table S9). In contrast, single‐reference DLPNO‐(U)CCSD(T) substantially overestimates the adiabatic rotational barrier, reflecting its limitations in regions of pronounced multireference character, as previously observed by Kaupp [39]. Broken‐symmetry DFT shows the opposite tendency, overstabilizing TS‐Rot and enhancing the contribution of adiabatic rotation (see Table S8).

After computing the spin‐orbit coupling matrix elements (Section 8.5, Supporting Information), we applied the thermal corrections to the electronic energies to model the nonadiabatic pathway consistently across the substituent series (see Section 8.4, Supporting Information and Table S16). We evaluated mean absolute errors (MAE) and root‐mean‐square errors (RMSE) and compared them with the experimental *ΔG* ^‡^ to assess the quantitative performance of the computational methods. All methods systematically underestimate the experimental barriers, indicating a largely uniform offset, as evidenced by the close correspondence between MAE and RMSE. Among the tested approaches, DLPNO‐(U)CCSD(T) and SF‐BH&HLYP‐D4 (both in the gas phase and with implicit SMD solvation) show the smallest deviations (MAE ca. 19–22 kJ/mol, Figure S68), while MRSF‐ωB97X‐D4 shows intermediate performance. CASPT2‐based methods and MRSF‐BH&HLYP exhibit larger underestimations of absolute barriers, underlining a tendency of these approaches to compress barrier heights [55]. Conventional (broken‐symmetry) DFT exhibits the largest deviations, highlighting its limitations for activation energies in regions of pronounced multireference character, as it predicts substantial contributions from adiabatic pathways, leading to near‐zero activation entropies (see Tables S13 and S16). Importantly, despite these quantitative differences, all multireference and spin‐flip methods yield a consistent mechanistic ordering of stationary points, identifying the nonadiabatic rotational pathway as dominant across the substituent series, with inversion remaining a minor contribution.

All multireference and spin‐flip approaches consistently yield negative activation entropies of comparable magnitude, consistent with the experiment (*e.g*., −32 ± 4 J/(mol K) for azobenzene experimentally *vs*. ‐30 at the XMS‐CASPT2 level; see Table 1 and Table S13). The computed activation entropies reproduce both the sign and the typical magnitude of the experimentally determined ΔS^‡^ values across the substituent series. Deviations in individual cases are comparable to the experimental uncertainty and reflect the intrinsic difficulty of extracting entropic parameters, rather than differences in the isomerization mechanism. The consistent observation of negative ΔS^‡^ in both experiment and theory provides independent and mutually reinforcing evidence for a nonadiabatic isomerization mechanism for all substituents studied.

**FIGURE 3:**
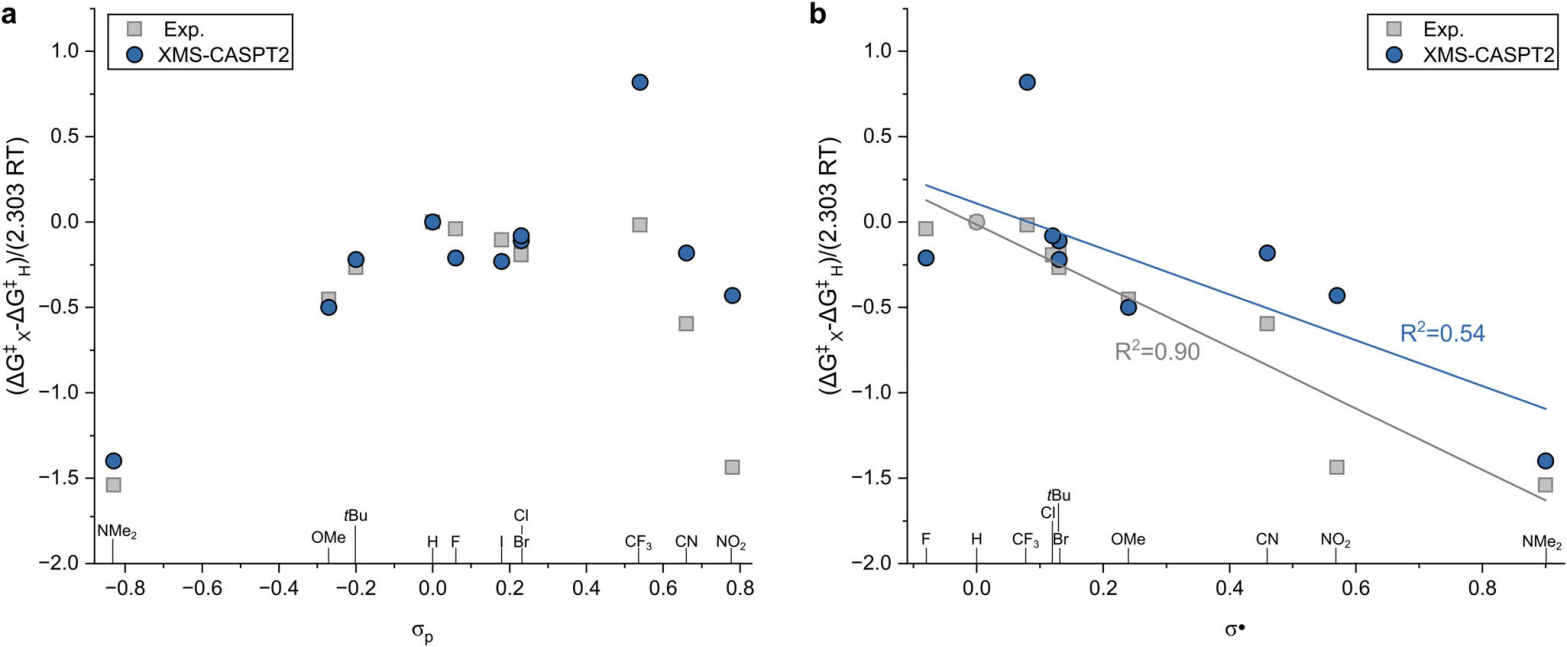
(a) Hammett plot of the experimentally obtained ΔG^‡^ at 298 K (grey) vs. the *ΔG* ^‡^ at 298 K computed at the XMS‐CASPT2/ANO‐RCC‐VTZP//B97X‐3c/SMD(toluene) level (blue) against σ_p_ parameters and (b) corresponding representation using the Creary σ·parameters.

Substituent effects were further examined by comparing experimental and computed free‐energy differences as a function of substituent parameters. In this analysis, Hammett σ_p_ parameters were used to probe the relative substituent response rather than to imply any linear free‐energy behavior. When viewed in this way, XMS‐CASPT2 emerges as the only method that consistently reproduces the qualitative shape and symmetry of the experimental substituent trend across the whole series, including strongly electron‐withdrawing groups (see Figure 3a). Other approaches either compress the response or introduce distortions that become more pronounced at the electronic extremes (see Section 8.7, Supporting Information). A comparable level of qualitative agreement is obtained when the data are represented using radical‐specific Creary σ· parameters, supporting the interpretation that substituent effects primarily reflect stabilization of open‐shell electronic structures accessed along the nonadiabatic pathway (see Figure 3b). Moreover, the computed *ΔH* ^‡^ values display a modest but systematic dependence on substituent electronics, whereas *ΔS* ^‡^ remains weakly substituent‐dependent and clustered around a common negative value, comparable to what is experimentally observed (*cf*. Figures S56 with S53 and S54).

Overall, these results emphasize that reproducing relative substituent behavior, together with a consistent description of entropic contributions, provides a meaningful criterion for evaluating computational descriptions of azobenzene thermal isomerization. Analyses based solely on rate constants or single‐reference electronic energies may lead to mechanistic conclusions that are unsupported when activation entropies are considered.

## 3 Conclusion

This study resolves a long‐standing ambiguity in the interpretation of substituent effects on the thermal *Z *→ *E* isomerization of azobenzenes. By combining temperature‐dependent kinetic measurements with activation‐parameter analysis, we show that all *para*‐substituted azobenzenes investigated proceed predominantly through the same nonadiabatic rotational pathway. Hence, the characteristic “bell‐shaped” dependence observed in Hammett‐type analyses does not indicate a change in mechanism, but instead reflects the inadequacy of σ_p_ parameters to describe transition states with pronounced open‐shell, diradicaloid character.

While the nonadiabatic nature of thermal isomerization in parent azobenzene had previously been established, the present work provides *experimental* evidence that this mechanistic picture extends across substituted derivatives. Uniformly negative activation entropies are observed throughout the series, and substituent‐dependent rate trends are consistently rationalized by stabilization of a common nonadiabatic transition region, as supported by Exner analysis and radical‐specific substituent parameters. Consequently, both electron‐donating and electron‐withdrawing groups modulate the same underlying electronic structure rather than promoting distinct mechanistic channels.

A key outcome of this work is the identification of the activation entropy as an indispensable diagnostic parameter for mechanistic assignment. Analyses based solely on rate constants or *ΔG* ^‡^ values can obscure the proper reaction pathway. In contrast, the sign and magnitude of *ΔS* ^‡^ directly reflect the involvement of nonadiabatic processes and should be considered alongside kinetic data in azobenzenes and related photoswitches.

Complementary computational benchmarking supports these conclusions. Methods that can describe near‐degenerate and open‐shell singlet electronic structures consistently identify nonadiabatic rotation as the dominant pathway. On the other hand, deviations arise primarily from single‐reference DFT approaches that fail to reproduce the experimental entropic signatures. Although no single computational protocol provides quantitatively accurate rate constants across the full substituent series, the strong correlation between computed triplet energies and radical‐specific substituent parameters demonstrates that simple open‐shell electronic descriptors can serve as a valuable qualitative tool for anticipating substituent effects. Together, these results define experimentally grounded criteria and methodological requirements for future predictive modelling of azo‐based photoswitches.

## Conflicts of Interest

The authors declare no conflicts of interest.

## Supporting information




**Supporting File 1**: The authors have cited additional references within the Supporting Information. The following files are provided as additional Supporting Information: the Python scripts kisc.py and qrrho.py to calculate the final computed rates; the Excel file ComputedEnergies_Rates_EyringAnalysis.xlsx containing the detailed output of the computations.

**Supporting File 2**: anie71529‐sup‐0002‐Data.zip.

## Data Availability

The data that support the findings of this study are available in the supplementary material of this article.
