# Supplementary material for: The Nonadiabatic Nature of the Substituent Effects in Azobenzene
Source: Angew Chem Int Ed Engl. 2026 Feb 20;65(14):e23613. doi: 10.1002/anie.202523613 (PMC13023701; doi:10.1002/anie.202523613)
Supplement: Supplementary file 1 — Supporting File 1: The authors have cited additional references within the Supporting Information. The following files are provided as additional Supporting Information: the Python scripts kisc.py and qrrho.py to calculate the final computed rates; the Excel file ComputedEnergies_Rates_EyringAnalysis.xlsx containing the detailed output of the computations. [file ANIE-65-e23613-s001.pdf]

Table of contents

1 Experimental Procedures ... 3
1.1 General Details ... 3
1.2 Reagent Information ... 4
1.3 Synthetic Procedures ... 5
2 NMR Spectra ... 11
3 Thermal Activation Analysis ... 23
3.1 Eyring Equation and Eyring Analysis ... 23
3.2 Rate Constants ... 24
3.3 Error Analysis ... 24
3.3.1 Errors on the Rate Constant ... 24
3.3.2 Errors on the Enthalpy and Entropy of Activation ... 24
3.3.3 Simple Linear Regression ... 25
3.3.4 The error in the Fitting Parameter and the Gibbs free Energy ... 27
4 Thermal Kinetics ... 28
5 Eyring Plots ... 42
6 Hammett and Activation Parameters Plots ... 49
7 Exner Plots ... 54
8 Computational Analysis ... 58
8.1 General Details for Optimizations ... 58
8.1.1 Ground State Minima Optimization ... 58
8.1.2 Triplet State Minima Optimization ... 58
8.1.3 Transition State for the Inversion Mechanism ... 58
8.1.4 Transition State for the Adiabatic Rotation Mechanism ... 59
8.1.5 Non-adiabatic Rotation Mechanism ... 59
8.1.6 On the Significance of the Conformational Space in the Study ... 59
8.2 On the Choice of DFT Method for Optimizations ... 59
8.3 Specific Details on the Methods Employed ... 60
8.3.1 Coupled Cluster Calculations ... 60
8.3.2 CASSCF/CASPT2 Calculations ... 61
8.3.3 SF-DFT and MRSF-TDDFT ... 64
8.3.4 Comparison of Electronic Energies Obtained with Different Methods ... 64
8.4 Wentzel-Kramers-Brillouin (WKB) theory ... 65
8.5 Calculation of the Spin-Orbit Coupling Matrix Elements ... 67
8.6 Thermal Corrections to the Electronic Energy ... 68
8.6.1 Error Analysis ... 72
8.7 Computed LFER Plots ... 74

8.8 Active Spaces 79
9 References 156

1 Experimental Procedures

1.1 General Details

All air-sensitive manipulations were performed under a positive atmosphere of purified argon using the standard high vacuum Schlenk-line technique. Air Liquide Europe supplied argon. All glassware was oven-dried and purged with argon before use.

NMR spectra were recorded on a Jeol Eclipse+ 400 (^{1}H, 400 MHz; ^{13}C, 101 MHz) and a Bruker Avance III HD 300 (^{1}H, 400 MHz; ^{13}C, 101 MHz; ^{19}F, 376 MHz) spectrometer at 298 K unless noted otherwise. Chemical shift values are quoted in δ (ppm) and coupling constants in *J* (Hz). ^{1}H chemical shift values are reported relative to tetramethylsilane (TMS) and referenced to the residual proton resonances of the corresponding deuterated solvent signal. ^{13}C{^{1}H} NMR spectra are reported relative to TMS using the natural abundance carbon resonances of the deuterated solvents. The following abbreviations (or combinations thereof) were used to describe multiplicities: s, singlet; d, doublet; t, triplet; m, multiplet; brs, broad singlet.

Reactions were recorded by thin-layer chromatography (TLC) using Merck TLC Silica Gel 60 F_{254} aluminium sheets or Merck TLC Aluminium oxide 60 F_{254} aluminium sheets and visualized by UV-light (λ = 365 nm or 254 nm). Chromatographic purifications of products were accomplished using flash column chromatography (FC) on pre-packed silica gel columns from Silicycle 60 (0.04 – 0.063 nm/230 – 400 mesh) and reversed-phase flash column chromatography (RPFC) on a pre-packed C18 flash column from TELOS on an automated low-pressure Biotage SP4 using eluent gradients. Otherwise, flash chromatography was performed using silica gel (pore size 60 Å, 400 mesh, 40-63 µm particle size) on a Biotage Isolera One using eluent gradients. Reversed-phase flash column chromatography (RP-FC) was performed on a Biotage Isolera One, with a Biotage Sfär C18 Duo column (100 Å, 30 µm), and MeCN/H_2O gradients with 0.1% of formic acid.

High-performance liquid chromatography (HPLC) analysis was performed on an Agilent 1260 Infinity II combined with an InfinityLab LC/MSD iQ mass detector (ESI) using an InfinityLab Poroshell 120 EC-C18 column at 40 °C eluted with MeCN/H_2O with 0.1% formic acid or with an Agilent 1290 Infinity II HPLC system equipped with a 1290 Infinity II high-speed pump and a 1260 II Infinity DAD HS UV-Vis detector, using an InfinityLab Poroshell 120 EC-C18 column with dimensions of 50 mm x 2.1 mm and 1.9 µm particle size. The HPLC is coupled to an InfinityLab LC/MSD G6125B detector equipped with an ESI source for ionization.

Irradiation experiments were performed in a screw-cap fluorescence cuvette (Hellma 117.100F-QS) containing a 5 mm PTFE-coated stirring bar (VWR), using a home-built fibre-coupled irradiation setup where LEDs (Thorlabs M340F4 and M395FP1) are coupled to an LED-driver (Thorlabs LEDD18) connected to a 400 µm 1-to-4 fan-out fibre (Thorlabs BF44LS01).

Kinetic measurements were recorded in a screw-cap fluorescence cuvette (Hellma 117.100F-QS) at various temperatures (ranging between 40 and 90 °C). The kinetic traces were measured on a Varian Cary 5000 UV-Visible spectrophotometer, using a thermostatted multicell holder accessory (Agilent Cary4000/5000/6000i/7000 UV-Vis NIR 6x6 Multicell Holder).

The temperatures inside the cuvette were measured with a two-channel K thermocouple thermometer (Sauermann (Si-TT3 HVACr 2-Channel Thermometer).

1.2 Reagent Information

All solvents and reagents were purchased from commercial suppliers (Sigma-Aldrich, TCI and ThermoFisher) and used as received. All solvents were reagent grade for both synthesis and purification and purchased from VWR. 4-(Dimethylamino)azobenzene was commercially bought from Sigma-Aldrich and used without further purification.

1.3 Synthetic Procedures

General procedure for the Baeyer-Mills reaction for azobenzene synthesis – **GP**

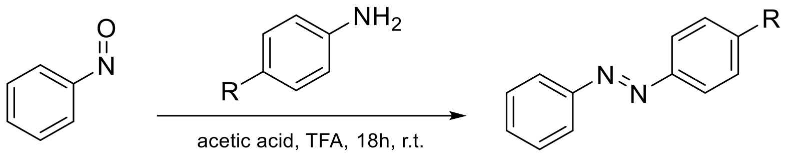

To a 50 mL crimp-top vial, nitrosobenzene *(1.0 eq.)* and the respective aniline *(1.1 eq.)* were added and dissolved in glacial acetic acid and trifluoroacetic acid. The reaction mixture was purged with argon and capped off. The reaction was stirred for 18 hours at room temperature and followed by TLC *(5% ethyl acetate in pentane)*. The mixture was poured on water (*200 mL per mmol of nitrosobenzene*) and dichloromethane *(200 mL)*, the resulting layers were separated, and the aqueous layer was extracted with dichloromethane *(2 x 100 mL)*. The combined organic layers were washed with saturated aqueous sodium bicarbonate solution *(2 x 150 mL)* and dried over anhydrous sodium sulphate. Afterwards, the mixture was concentrated in vacuo and purified by FC chromatography *(0.5% ethyl acetate in pentane)* and RP-FC *(5-95% acetonitrile in water containing 0.1% formic acid)*. The yield was determined, and the resulting compounds were characterized by ^{1}H, ^{13}C and ^{19}F NMR, and LC-MS.

Synthesis of azobenzene

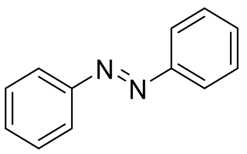

According to **GP**: nitrosobenzene *(95 mg, 0.9 mmol, 1.0 eq.)*, aniline *(0.1 mL, 1.0 mmol, 1.2 eq.)* were dissolved in glacial acetic acid *(10 mL)* and trifluoroacetic acid *(0.5 mL)*. The reaction mixture was concentrated in vacuo and purified by FC. This afforded an orange crystalline solid *(139 mg, 86%)*.

^{1}H-NMR ($CDCl_3$, 400 MHz): δ 7.96 – 7.91 (m, 4H), 7.58 – 7.45 (m, 6H).

^{13}C-NMR ($CDCl_3$, 101 MHz): δ 152.8, 131.1, 129.2, 123.0.

LCMS-ESI^{+} (m/z): calcd. for $C_{12}H_{11}N_2^{+}$ $[M+H]^{+}$ 183.1 found 183.0.

The analytical data are in accordance with published data.[1]

Synthesis of 4-(*tert*-butyl)azobenzene

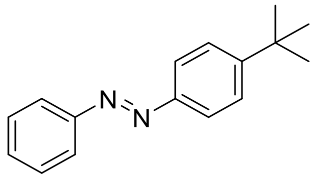

According to **GP**: nitrosobenzene *(252 mg, 2.4 mmol, 1.0 eq.)*, 4-*tert*-butylaniline *(409 µL, 2.6 mmol, 1.1 eq.)* were dissolved in glacial acetic acid *(25 mL)* and trifluoroacetic acid *(0.5 mL)*. The reaction mixture was concentrated in vacuo and purified by FC. This afforded an orange oil *(121 mg, 22%)*.

^{1}H-NMR ($CDCl_3$, 300 MHz): δ 7.97 – 7.82 (m, 4H), 7.60 – 7.41 (m, 5H), 1.39 (s, 9H).

^{13}C-NMR ($CDCl_3$, 101 MHz): δ 154.7, 153.0, 150.8, 130.8, 129.2, 126.2, 122.9, 122.7, 35.2, 31.4.

LCMS-ESI$^+$ (m/z): calcd. for $C_{16}H_{19}N_2^+$ $[M+H]^+$ 239.2; found 239.1.

The analytical data are in accordance with previously published data.[1]

Synthesis of 4-fluoroazobenzene

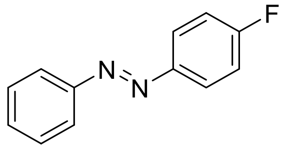

According to **GP**: nitrosobenzene *(504 mg, 4.7 mmol, 1.0 eq.)*, 4-fluoroaniline *(486 µL, 5.1 mmol, 1.1 eq.)* were dissolved in glacial acetic acid *(25 mL)* and trifluoroacetic acid *(0.5 mL)*. The reaction mixture was concentrated in vacuo and purified by FC and RPFC. This afforded an orange crystalline solid *(390 mg, 41%)*.

^{1}H-NMR ($CDCl_3$, 300 MHz): δ 8.02 – 7.84 (m, 4H), 7.51 (td, *J* = 8.1, 5.6 Hz, 3H), 7.20 (t, *J* = 8.6 Hz, 2H).

^{13}C-NMR ($CDCl_3$, 101 MHz): δ 166.2, 162.9, 152.6, 149.3, 131.2, 129.3, 125.1, 125.0, 123.0, 116.3, 116.0.

^{19}F-NMR ($CDCl_3$, 376 MHz): δ -109.4.

LCMS-ESI$^+$ (m/z): calcd. for $C_{12}H_{10}FN_2^+$ $[M+H]^+$ 201.1; found 201.0.

The analytical data are in accordance with published data.[2]

Synthesis of 4-chloroazobenzene

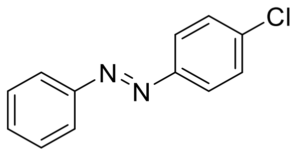

According to **GP**: nitrosobenzene *(95 mg, 0.9 mmol, 1.0 eq.)*, 4-chloroaniline *(124 mg, 1.0 mmol, 1.1 eq.)* were dissolved in glacial acetic acid *(10 mL)* and trifluoroacetic acid *(0.5 mL)*. The reaction mixture was concentrated in vacuo and purified by FC. This afforded an orange crystalline solid *(145 mg, 75%)*.

^{1}H-NMR ($CDCl_3$, 400 MHz): δ 7.97 – 7.83 (m, 4H), 7.58 – 7.44 (m, 5H).

^{13}C-NMR ($CDCl_3$, 101 MHz): δ 152.6, 151.1, 137.1, 131.4, 129.5, 129.3, 124.3, 123.1.

LCMS-ESI$^+$ (m/z): calcd. for $C_{12}H_{10}ClN_2^+$ $[M+H]^+$ 217.1; found 217.0 (^{35}Cl), 219.0 (^{37}Cl).

The analytical data are in accordance with published data.[1]

Synthesis of 4-bromoazobenzene

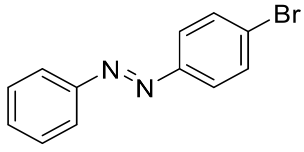

According to **GP**: nitrosobenzene *(86 mg, 0.8 mmol, 1.0 eq.)*, 4-bromoaniline *(150 mg, 0.9 mmol, 1.1 eq.)* were dissolved in glacial acetic acid *(15 mL)* and trifluoroacetic acid *(0.5 mL)*. The reaction mixture was concentrated in vacuo and purified by FC and RPFC. This afforded an orange crystalline solid *(47 mg, 22%)*.

^{1}H-NMR ($CDCl_3$, 300 MHz): δ 7.97 – 7.85 (m, 2H), 7.85 – 7.61 (AA’BB’, 4H), 7.59 – 7.44 (m, 3H).

^{13}C-NMR ($CDCl_3$, 101 MHz): δ 152.6, 151.5, 132.5, 131.5, 129.3, 125.5, 124.5, 123.1.

LCMS-ESI$^+$ (m/z): calcd. for $C_{12}H_{10}BrN_2^+$ $[M+H]^+$ 261.0; found 261.0 (^{79}Br), 262.9 (^{81}Br).

The analytical data are in accordance with published data.[1]

Synthesis of 4-iodoazobenzene

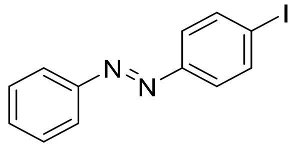

According to **GP**: nitrosobenzene *(249 mg, 2.3 mmol, 1.0 eq.)*, 4-iodoaniline *(574 mg, 2.6 mmol, 1.1 eq.)* were dissolved in glacial acetic acid *(25 mL)* and trifluoroacetic acid *(0.5 mL)*. The reaction mixture was concentrated in vacuo and purified by FC. This afforded an orange crystalline solid *(278 mg, 39%)*.

^{1}H-NMR ($CDCl_3$, 400 MHz): δ 7.97 – 7.82 (m, 4H), 7.66 (m, 2H), 7.59 – 7.47 (m, 3H).

^{13}C-NMR ($CDCl_3$, 101 MHz): δ 152.6, 152.1, 138.5, 131.5, 129.3, 124.6, 123.1, 97.8.

LCMS-ESI$^+$ (m/z): calcd. for $C_{12}H_{10}IN_2^+$ $[M+H]^+$ 309.0; found 308.9.

The analytical data are in accordance with published data.[1]

Synthesis of 4-(trifluoromethyl)azobenzene

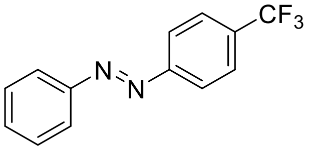

According to **GP**: nitrosobenzene *(232 mg, 2.2 mmol, 1.0 eq.)*, 4-(trifluoromethyl)aniline *(388 mg, 2.4 mmol, 1.1 eq.)* were dissolved in glacial acetic acid *(25 mL)* and trifluoroacetic acid *(0.5 mL)*. The reaction mixture was concentrated in vacuo and purified by FC. This afforded an orange crystalline solid *(231 mg, 42%)*.

^{1}H-NMR ($CDCl_3$, 400 MHz): δ 8.06 – 7.91 (m, 4H), 7.79 (d, J = 8.3 Hz, 2H), 7.60 – 7.49 (m, 3H).

^{13}C-NMR ($CDCl_3$, 101 MHz): δ 154.6, 152.6, 132.4 (q, $^2J_{CF}$ =32.4 Hz), 132.0, 129.3, 126.4 (q, $^3J_{CF}$ = 3.7 Hz), 124.1 (q, $^1J_{CF}$ = 272.3 Hz), 123.3, 123.2.

^{19}F-NMR ($CDCl_3$, 376 MHz): δ -62.6.

LCMS-ESI$^+$ (m/z): calcd. for $C_{13}H_{10}F_3N_2^+$ $[M+H]^+$ 251.1; found 251.0.

The analytical data are in accordance with published data.[1]

Synthesis of 4-nitroazobenzene

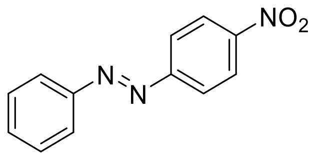

According to **GP**: nitrosobenzene *(251 mg, 2.3 mmol, 1.0 eq.)*, 4-nitroaniline *(356 mg, 2.6 mmol, 1.1 eq.)* were dissolved in glacial acetic acid *(25 mL)* and trifluoroacetic acid *(0.5 mL)*. The reaction mixture was concentrated in vacuo and purified by FC and RPFC. This afforded an orange crystalline solid *(66 mg, 12%)*.

^{1}H-NMR ($CDCl_3$, 300 MHz): δ 8.44 – 8.35 (m, 2H), 8.08 – 7.93 (m, 4H), 7.62 – 7.51 (m, 3H).

^{13}C-NMR ($CDCl_3$, 101 MHz): δ 155.9, 152.5, 148.9, 132.6, 129.5, 124.9, 123.6, 123.6.

LCMS-ESI$^+$ (m/z): calcd. for $C_{12}H_{10}N_3O_2^+$ $[M+H]^+$ 228.1; found 228.0.

The analytical data are in accordance with published data.[3]

Synthesis of 4-cyanoazobenzene

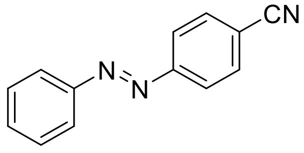

According to **GP**: nitrosobenzene *(250 mg, 2.3 mmol, 1.0 eq.)*, 4-cyanoaniline *(310 mg, 2.6 mmol, 1.1 eq.)* were dissolved in glacial acetic acid *(25 mL)* and trifluoroacetic acid *(0.5 mL)*. The reaction mixture was concentrated in vacuo and purified by FC. This afforded an orange crystalline solid *(105 mg, 22%)*.

^{1}H-NMR ($CDCl_3$, 400 MHz): δ 8.05 – 7.91 (m, 4H), 7.86 – 7.78 (m, 2H), 7.60 – 7.49 (m, 3H).

^{13}C-NMR ($CDCl_3$, 101 MHz): δ 154.6, 152.5, 133.4, 132.4, 129.4, 123.5, 123.5, 118.6, 114.1.

LCMS-ESI$^+$ (m/z): calcd. for $C_{13}H_{10}N_3^+$ $[M+H]^+$ 208.1; found 208.1.

The analytical data are in accordance with published data.[3]

Synthesis of 4-methoxyazobenzene

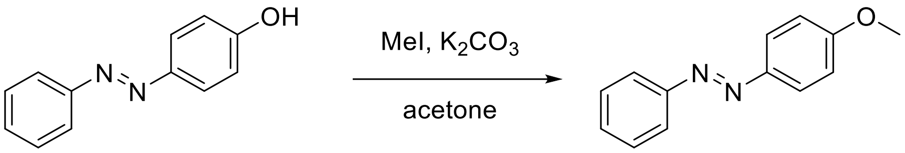

To a 25 mL crimp top vial, 4-hydroxyazobenzene *(199 mg, 1.0 mmol, 1.0 eq.)* was added and dissolved in acetone *(10 mL),* followed by the addition of potassium carbonate *(160 mg, 1.1 mmol, 1.1 eq.)* and iodomethane *(0.1 mL, 1.6 mmol, 1.6 eq.)*. The reaction was stirred overnight at room temperature. After full completion *(TLC, 50% ethyl acetate in pentane),* the organic layer was washed with 1 M sodium hydroxide *(3 x 10 mL)*, brine *(10 mL)*, dried over anhydrous sodium sulphate, and concentrated in vacuo. This afforded an orange crystalline solid *(200 mg, 94%)*.

^{1}H-NMR ($CDCl_3$, 400 MHz): δ 7.96 – 7.85 (m, 4H), 7.54 – 7.40 (m, 3H), 7.05 – 7.00 (m, 2H), 3.90 (s, 3H).

^{13}C-NMR ($CDCl_3$, 101 MHz): δ 162.2, 152.9, 147.2, 130.5, 129.2, 124.9, 122.7, 114.4, 55.7.

LCMS-ESI$^+$ (m/z): calcd. for $C_{13}H_{13}N_2O^+$ $[M+H]^+$ 213.1; found 213.1.

The analytical data are in accordance with published data.[1]

2 NMR Spectra

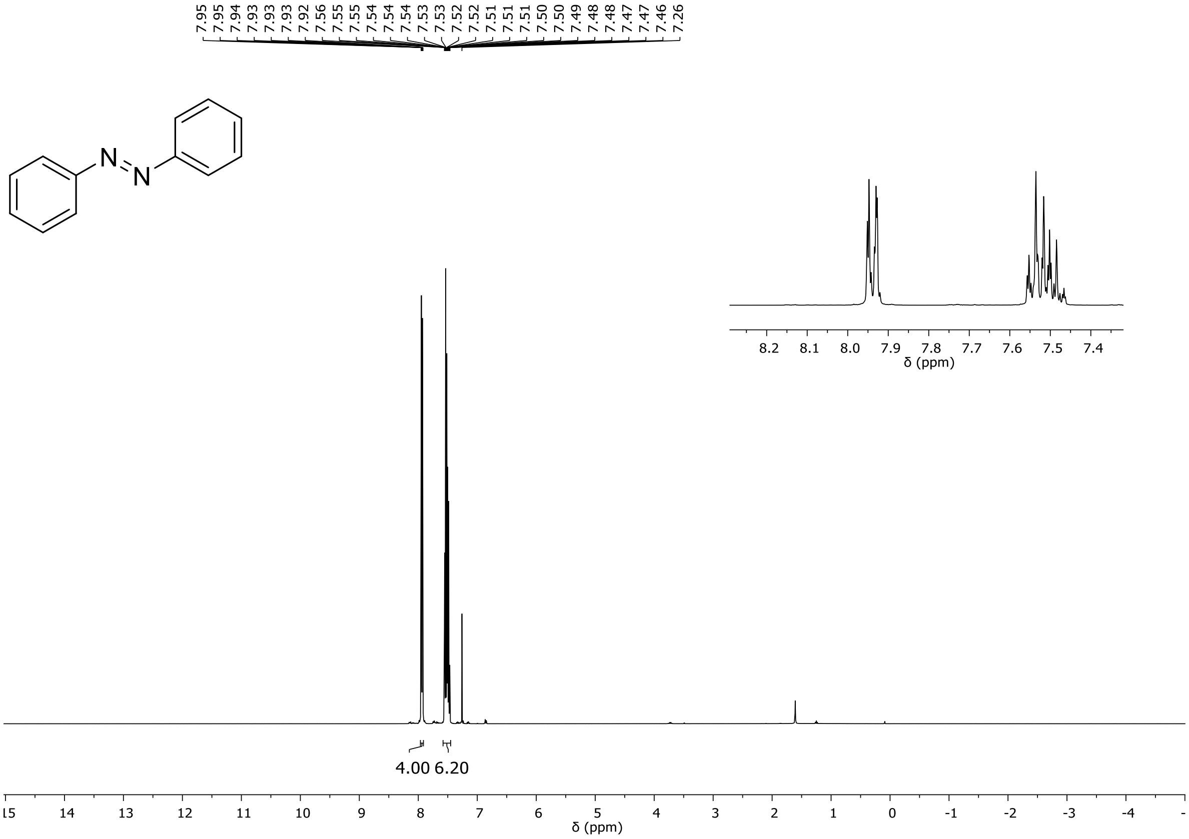

Figure S1. ^1H NMR of azobenzene in $CDCl_3$ at 25 °C.

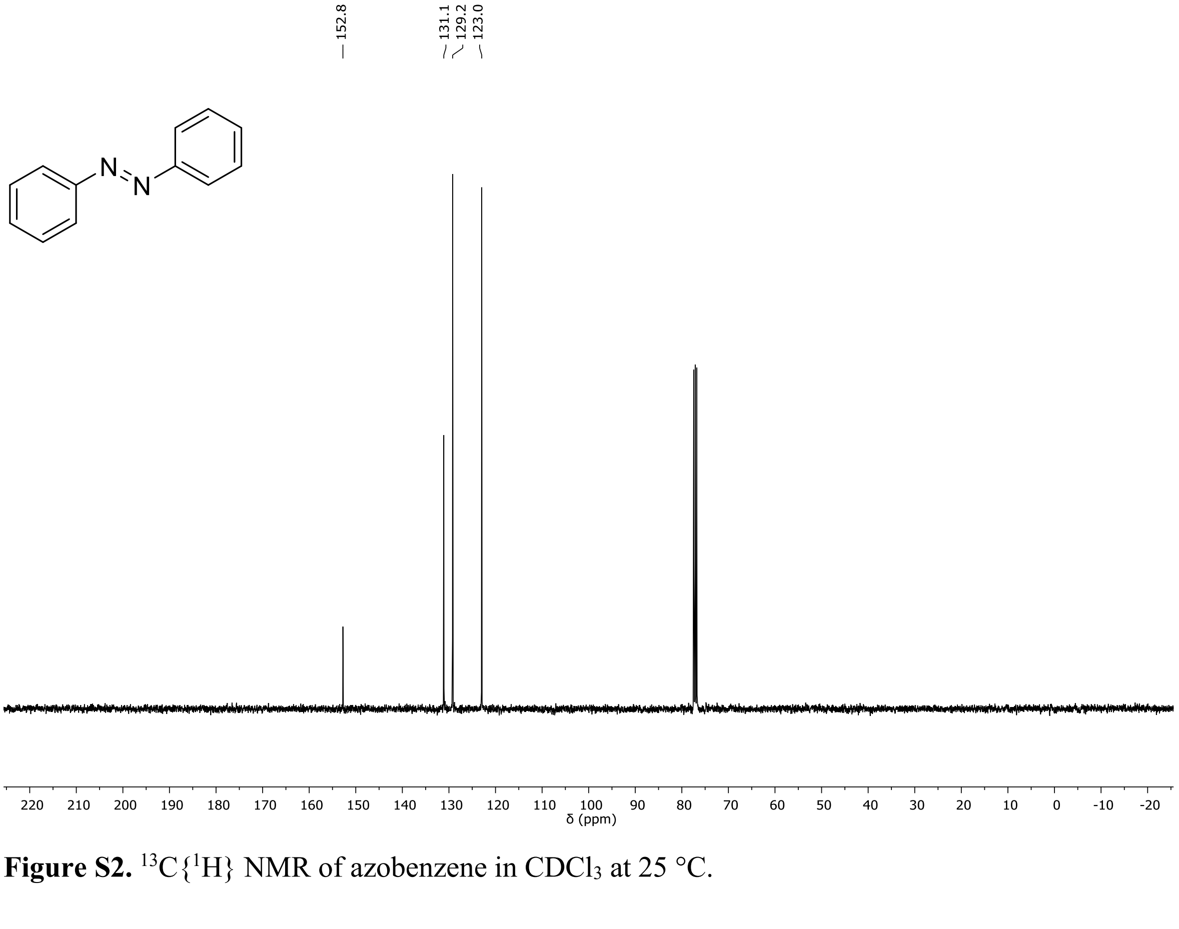

Figure S2. $^{13}C\{^1H\}$ NMR of azobenzene in $CDCl_3$ at 25 °C.

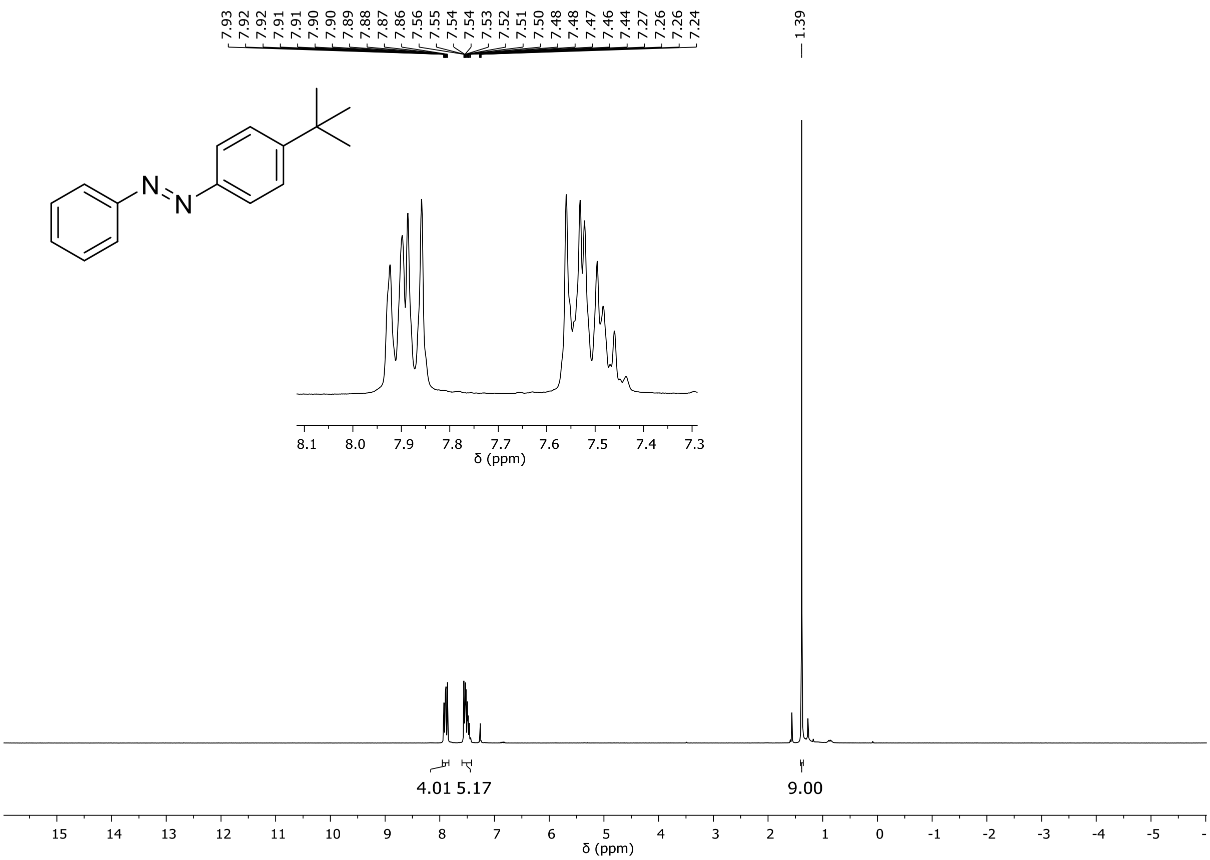

Figure S3. ^{1}H NMR of 4-(tert)butylazobenzene in $CDCl_3$ at 25 °C.

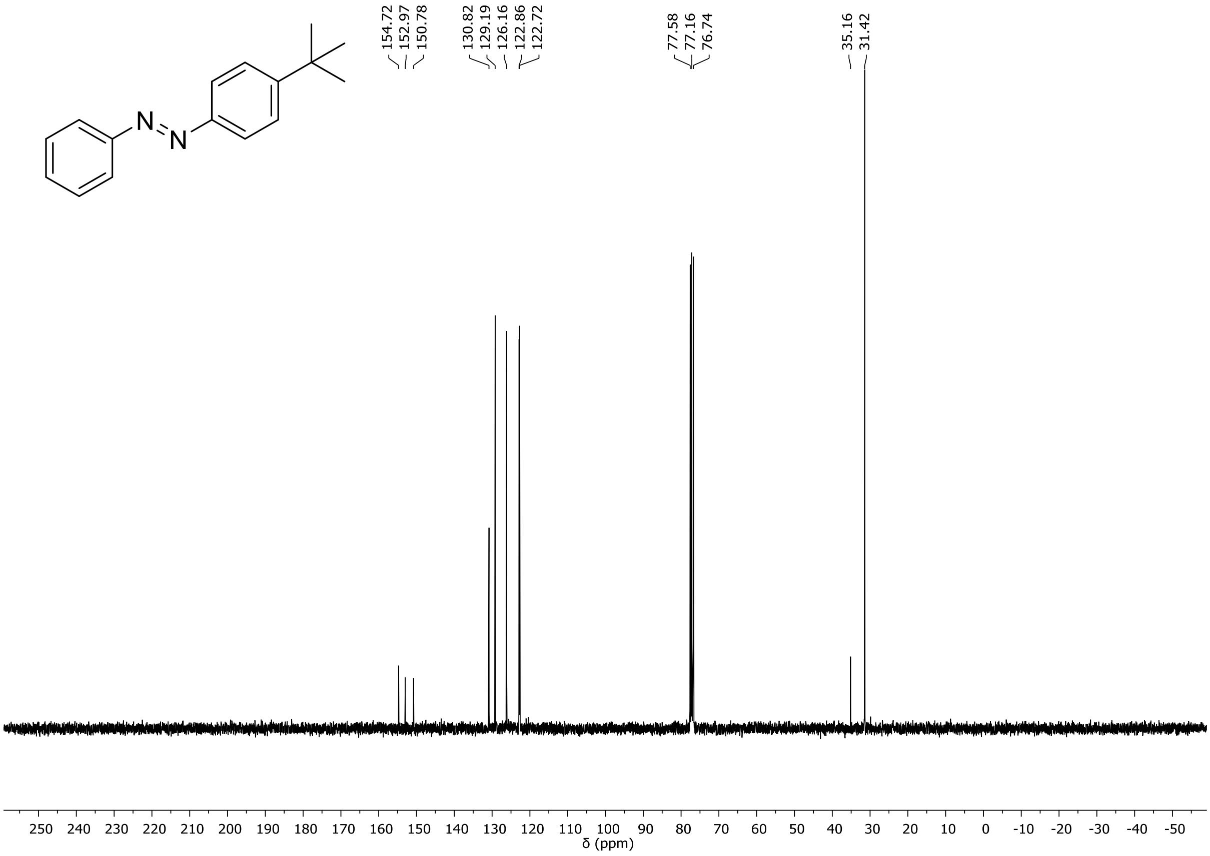

Figure S4. $^{13}C\{^{1}H\}$ NMR of 4-(tert)butylazobenzene in $CDCl_3$ at 25 °C.

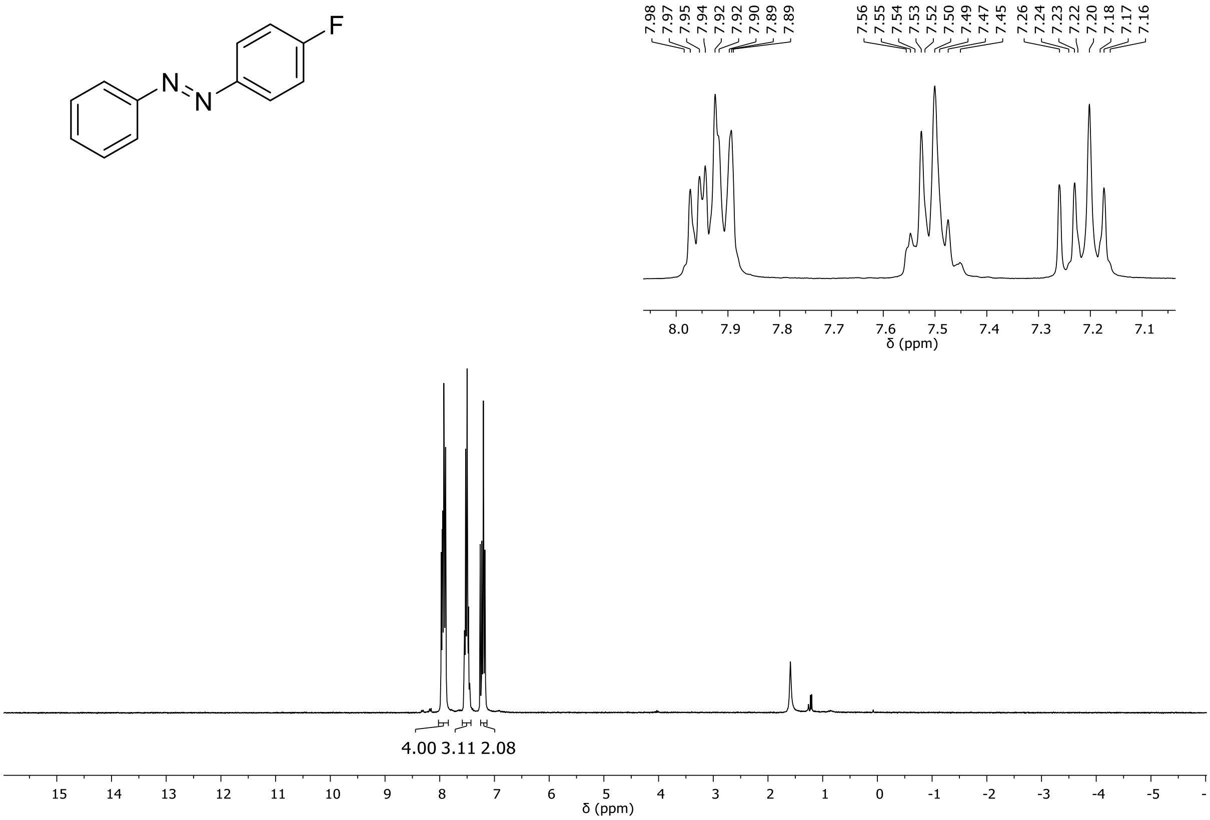

Figure S5. ^{1}H NMR of 4-fluoroazobenzene in $CDCl_3$ at 25 °C.

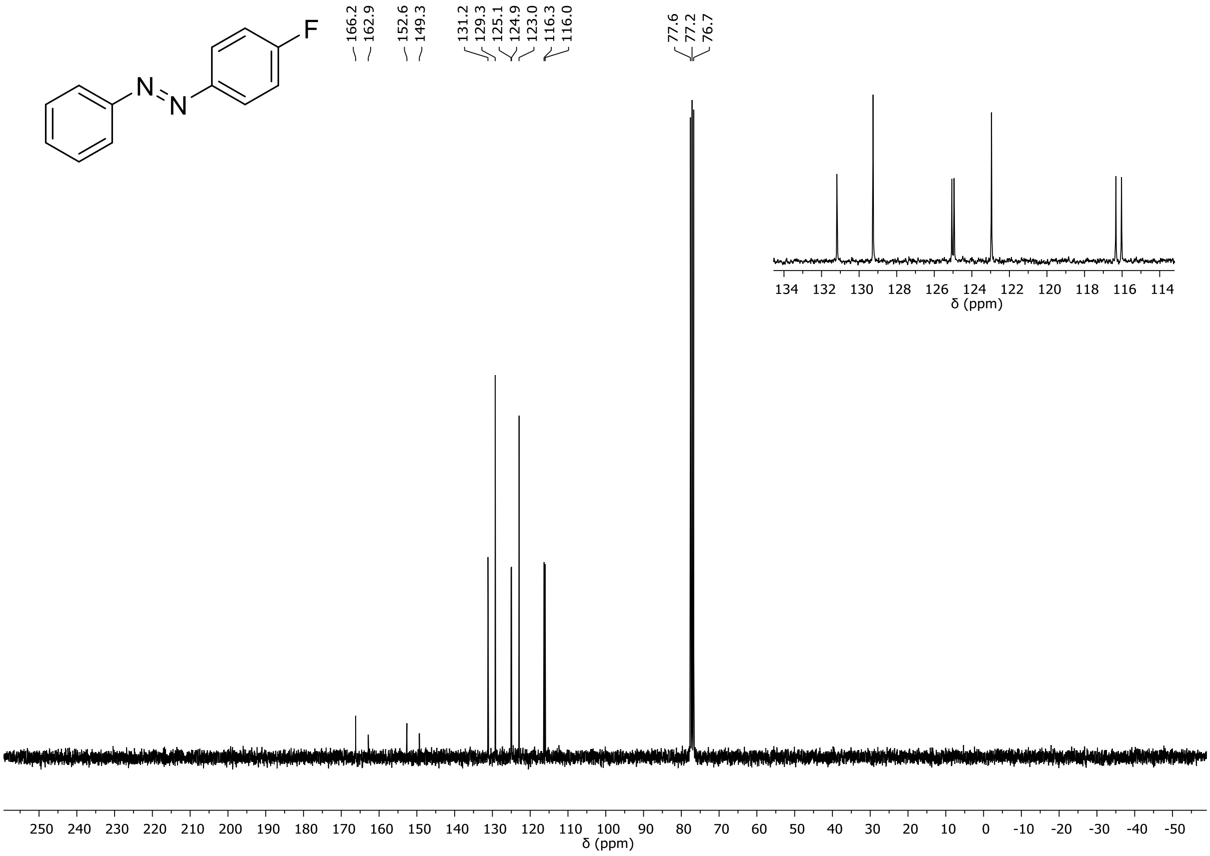

Figure S6. $^{13}C\{^{1}H\}$ NMR of 4-fluoroazobenzene in $CDCl_3$ at 25 °C.

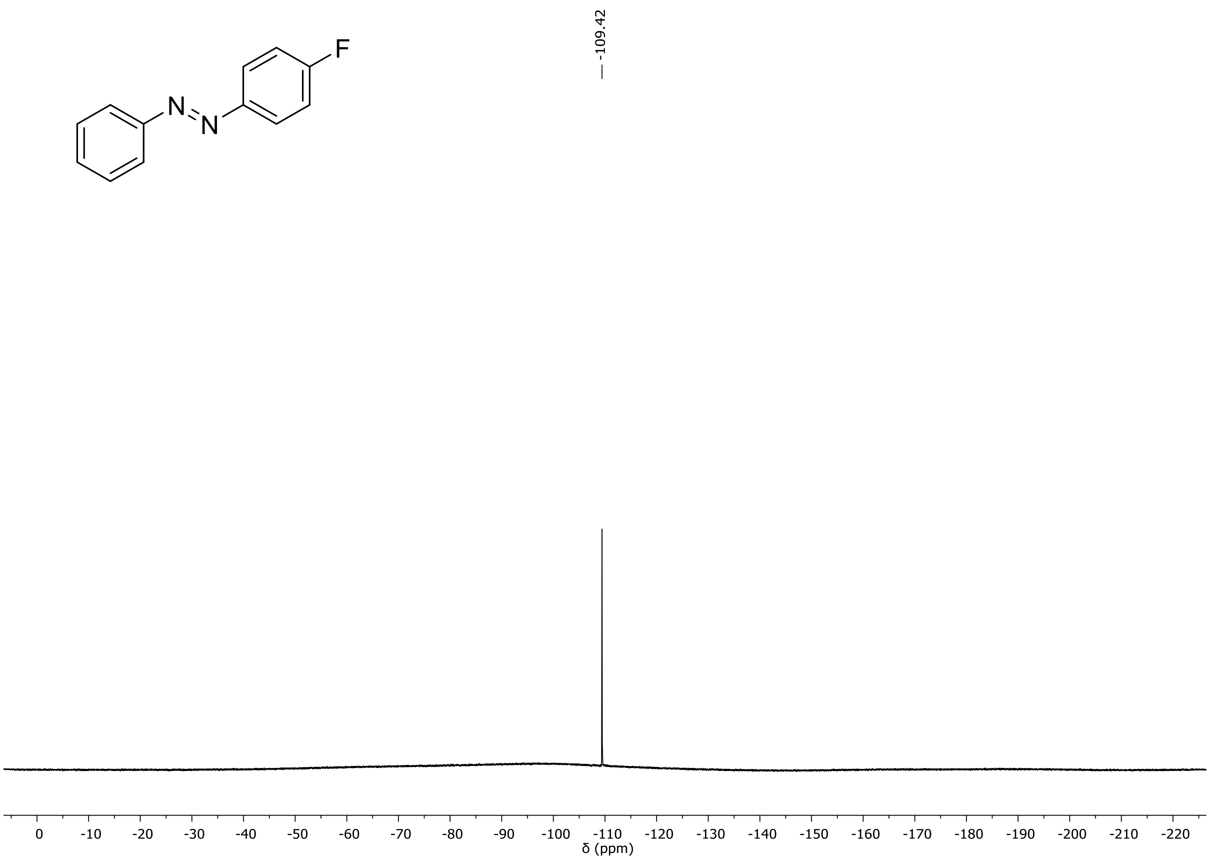

Figure S7. ^{19}F NMR of 4-fluoroazobenzene in $CDCl_3$ at 25 °C.

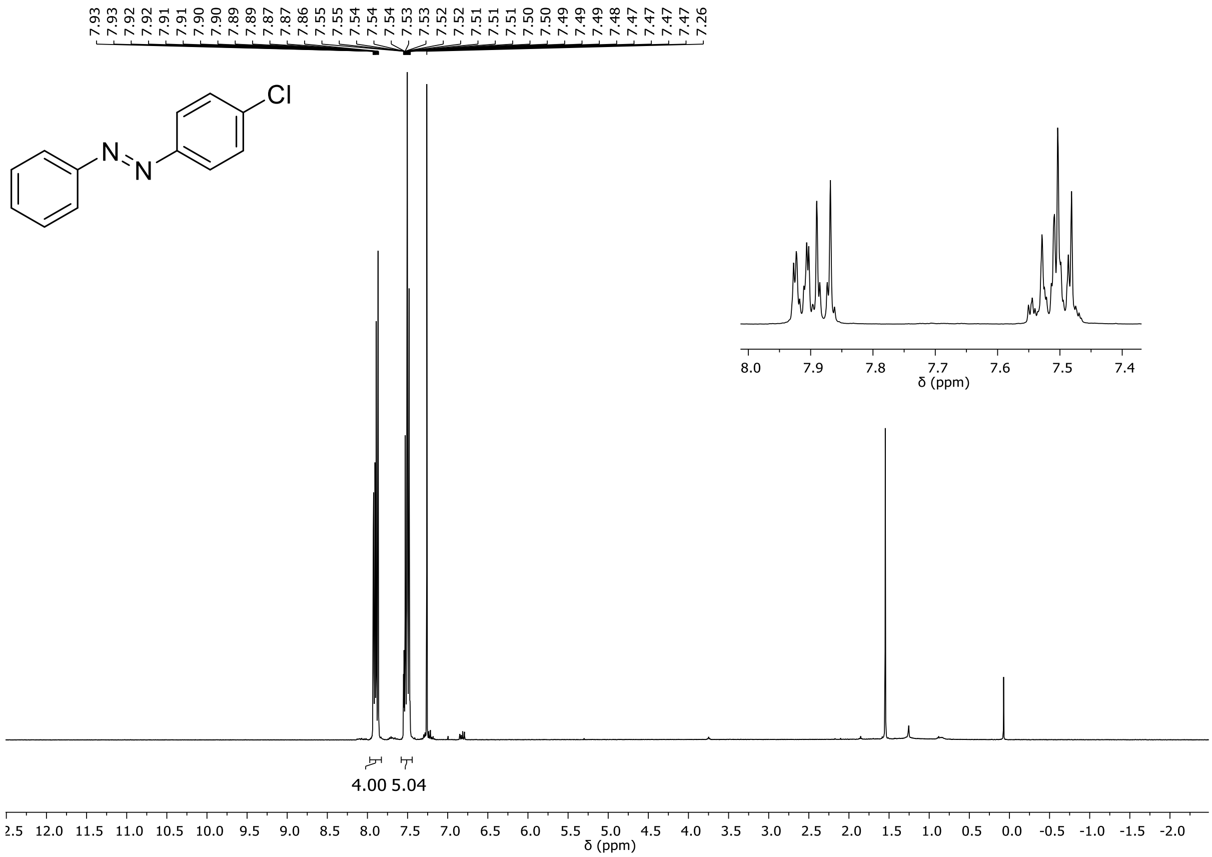

Figure S8. ^{1}H NMR of 4-chloroazobenzene in $CDCl_3$ at 25 °C. Small amounts of the *Z*-isomer and grease are not peaked.

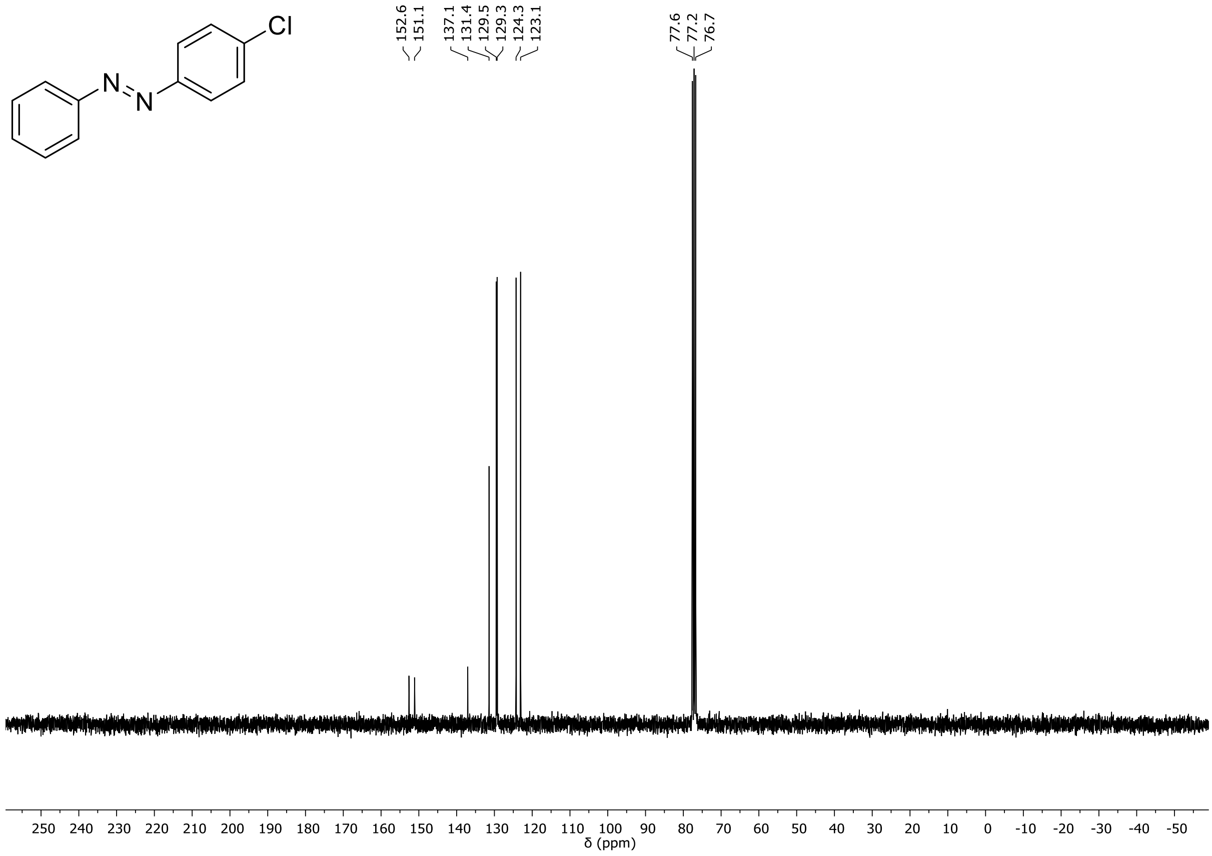

Figure S9. $^{13}C\{^{1}H\}$ NMR of 4-chloroazobenzene in $CDCl_3$ at 25 °C.

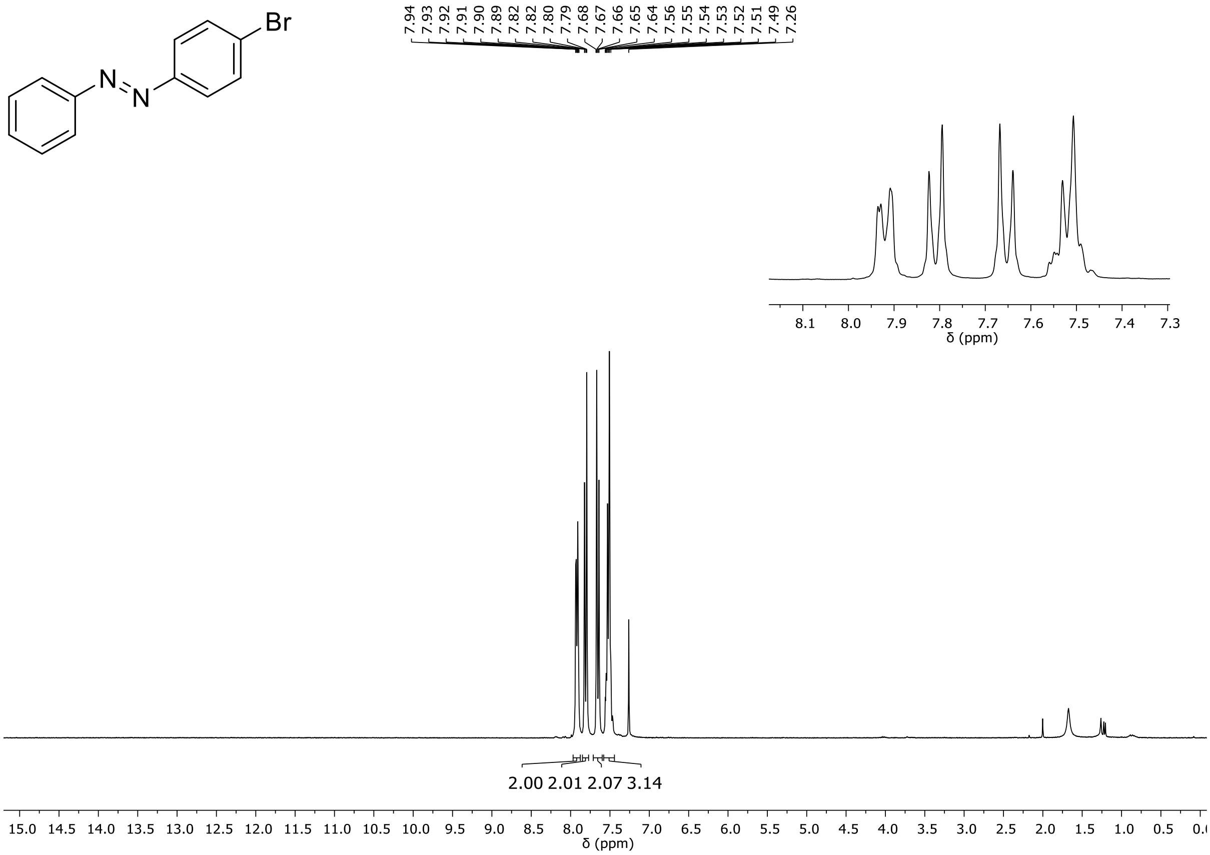

Figure S10. ^{1}H NMR of 4-bromoazobenzene in $CDCl_3$ at 25 °C.

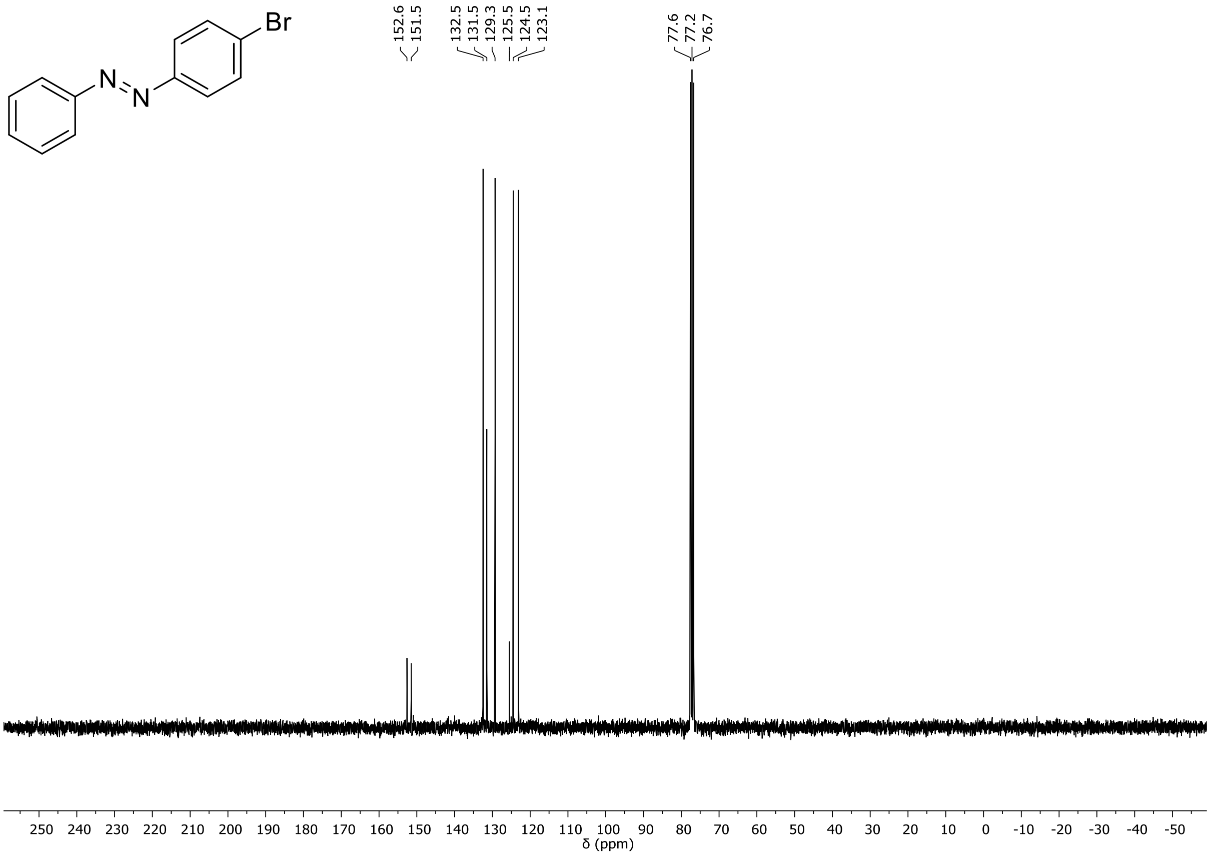

Figure S11. $^{13}C\{^{1}H\}$ NMR of 4-bromoazobenzene in $CDCl_3$ at 25 °C.

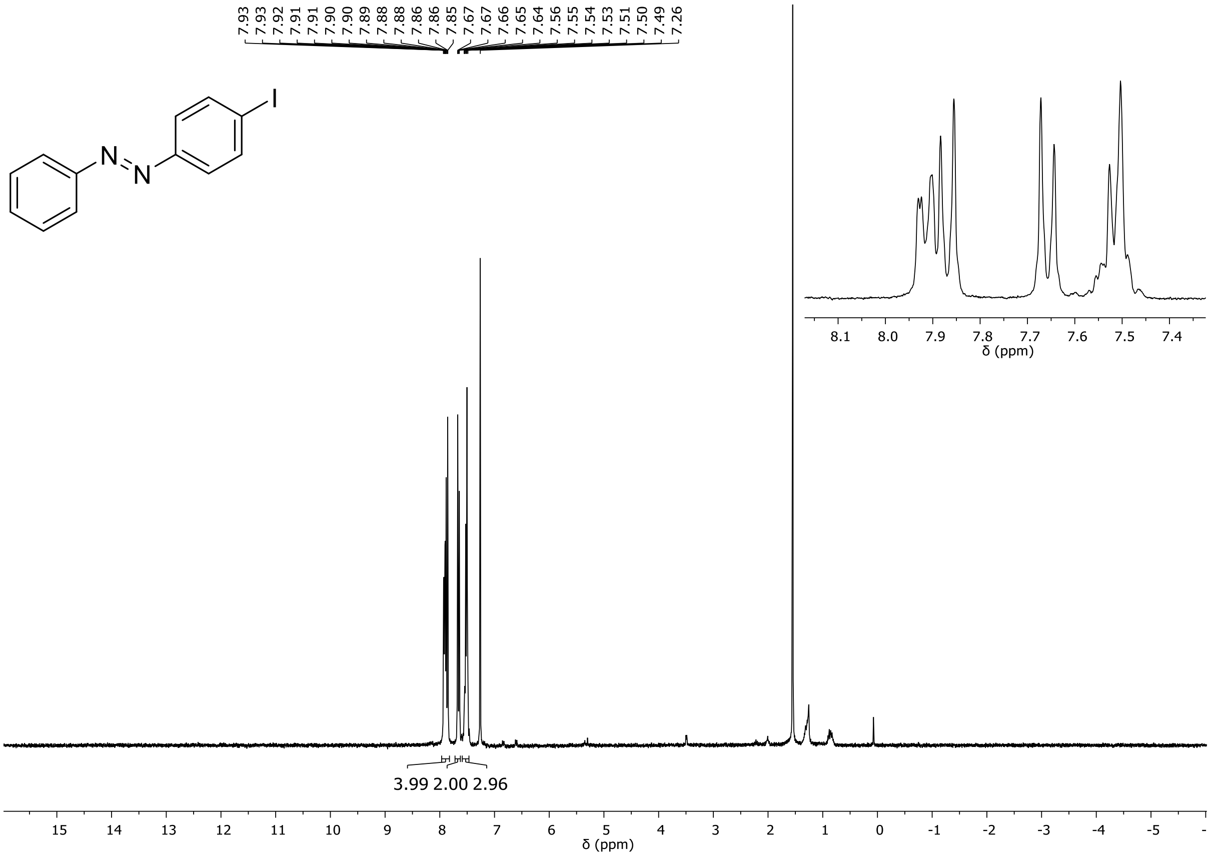

Figure S12. ^{1}H NMR of 4-iodoazobenzene in $CDCl_3$ at 25 °C.

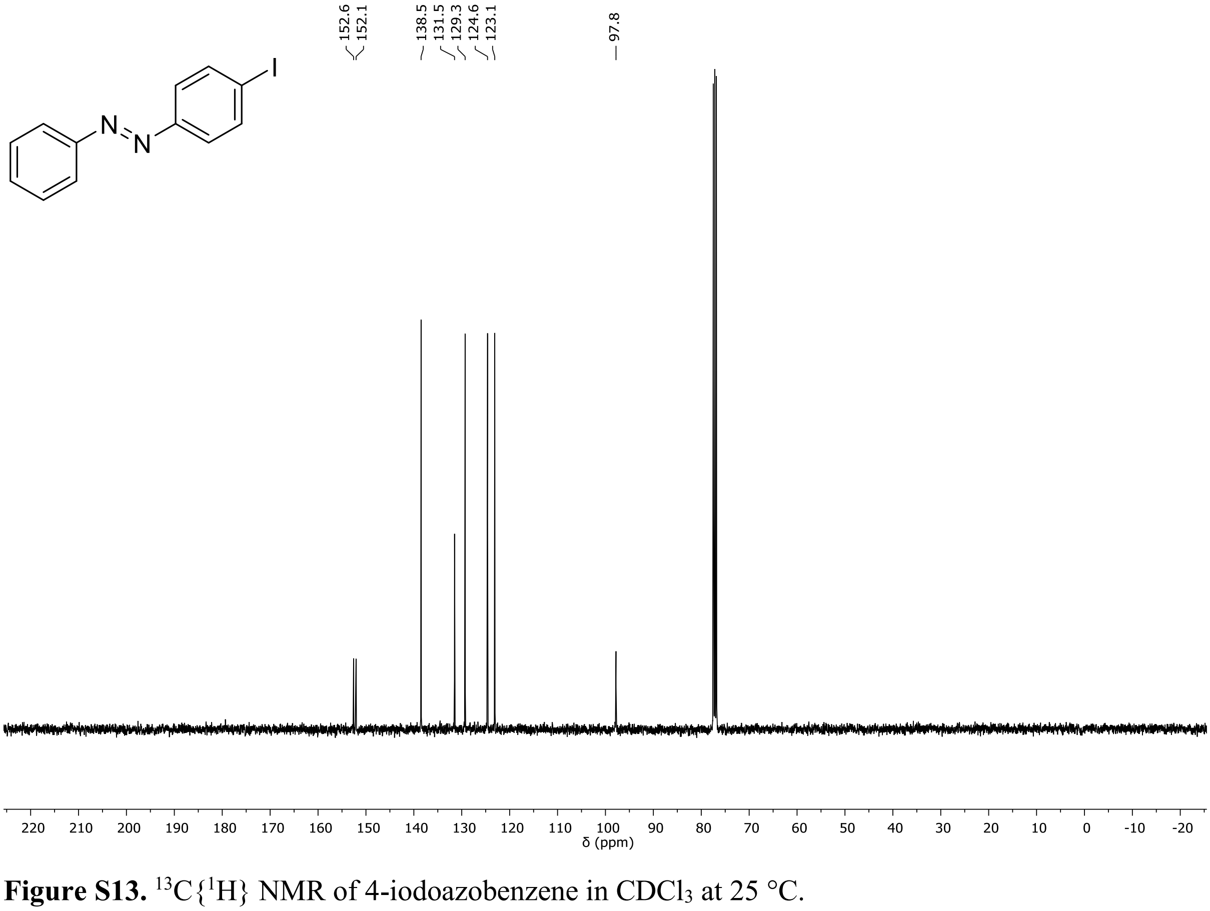

Figure S13. $^{13}C\{^1H\}$ NMR of 4-iodoazobenzene in $CDCl_3$ at 25 °C.

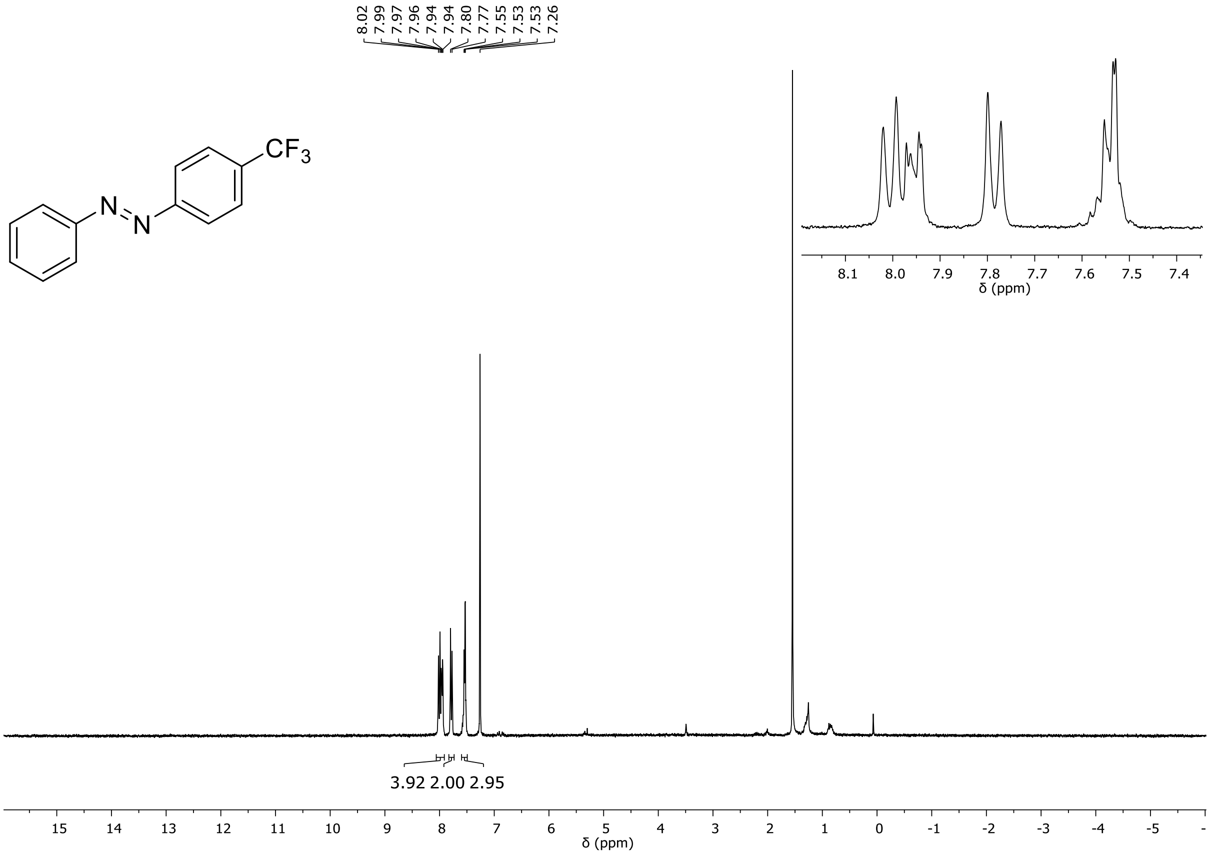

Figure S14. ^{1}H NMR of 4-(trifluoromethyl)azobenzene in $CDCl_3$ at 25 °C.

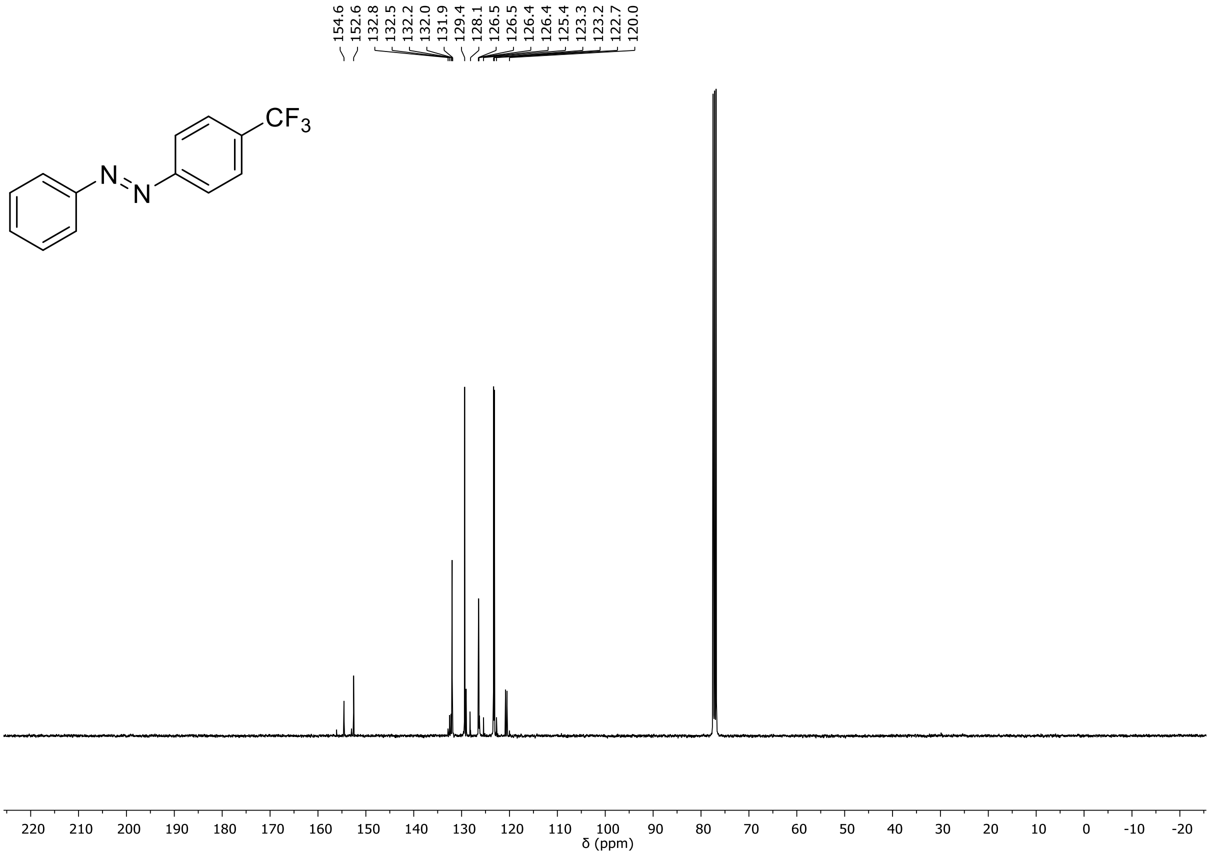

Figure S15. $^{13}C\{^{1}H\}$ NMR of 4-(trifluoromethyl)azobenzene in $CDCl_3$ at 25 °C. Fluorine-couplings are visible throughout the aromatic region.

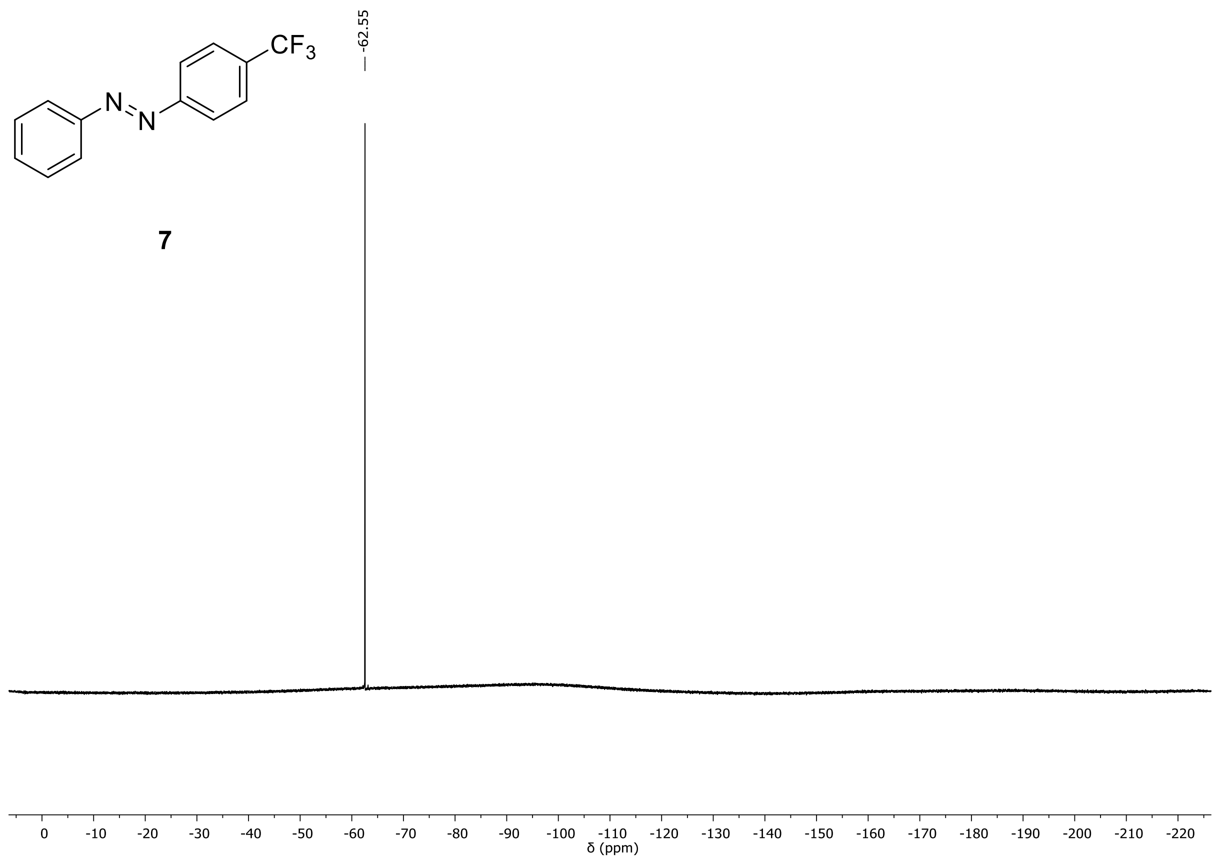

Figure S16. ^{19}F NMR of 4-(trifluoromethyl)azobenzene in $CDCl_3$ at 25 °C.

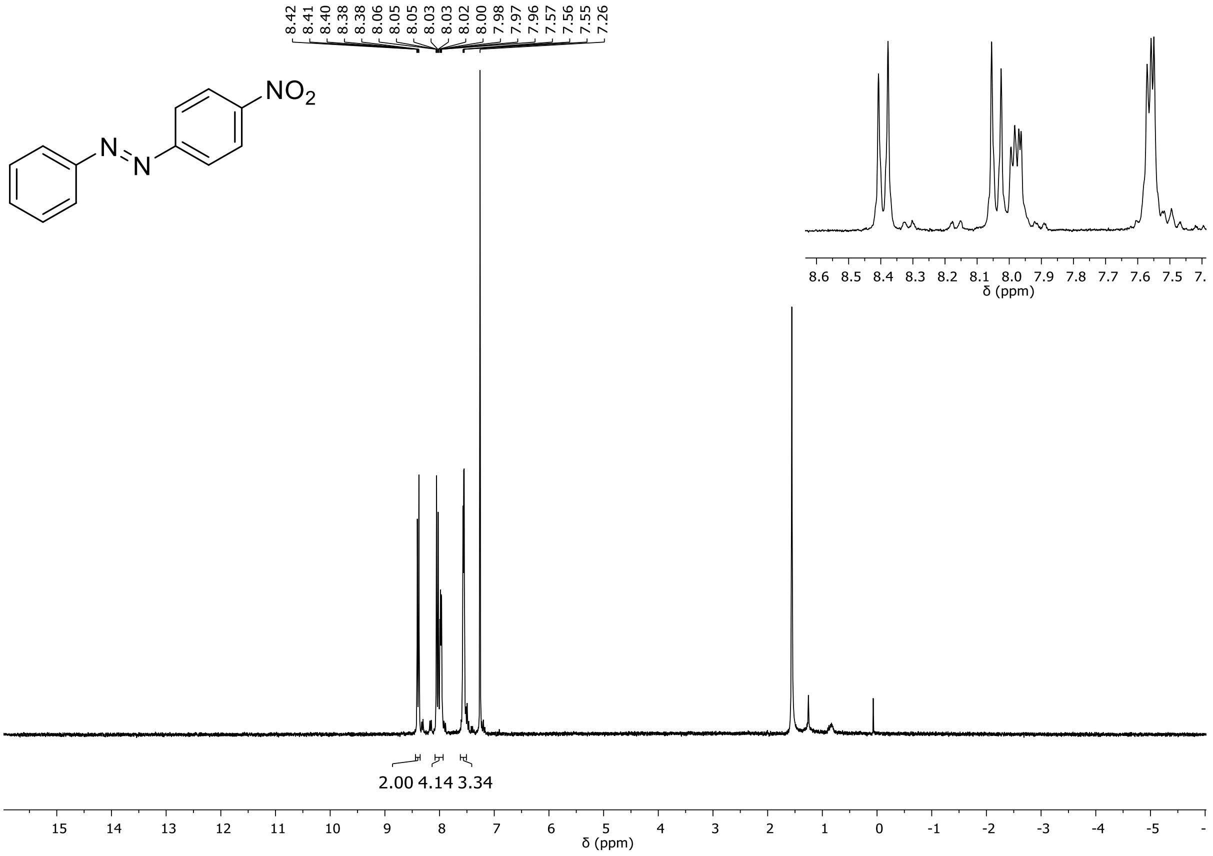

Figure S17. ^{1}H NMR of 4-nitroazobenzene in $CDCl_3$ at 25 °C. Small amounts of the *Z*-isomer and grease are not peaked.

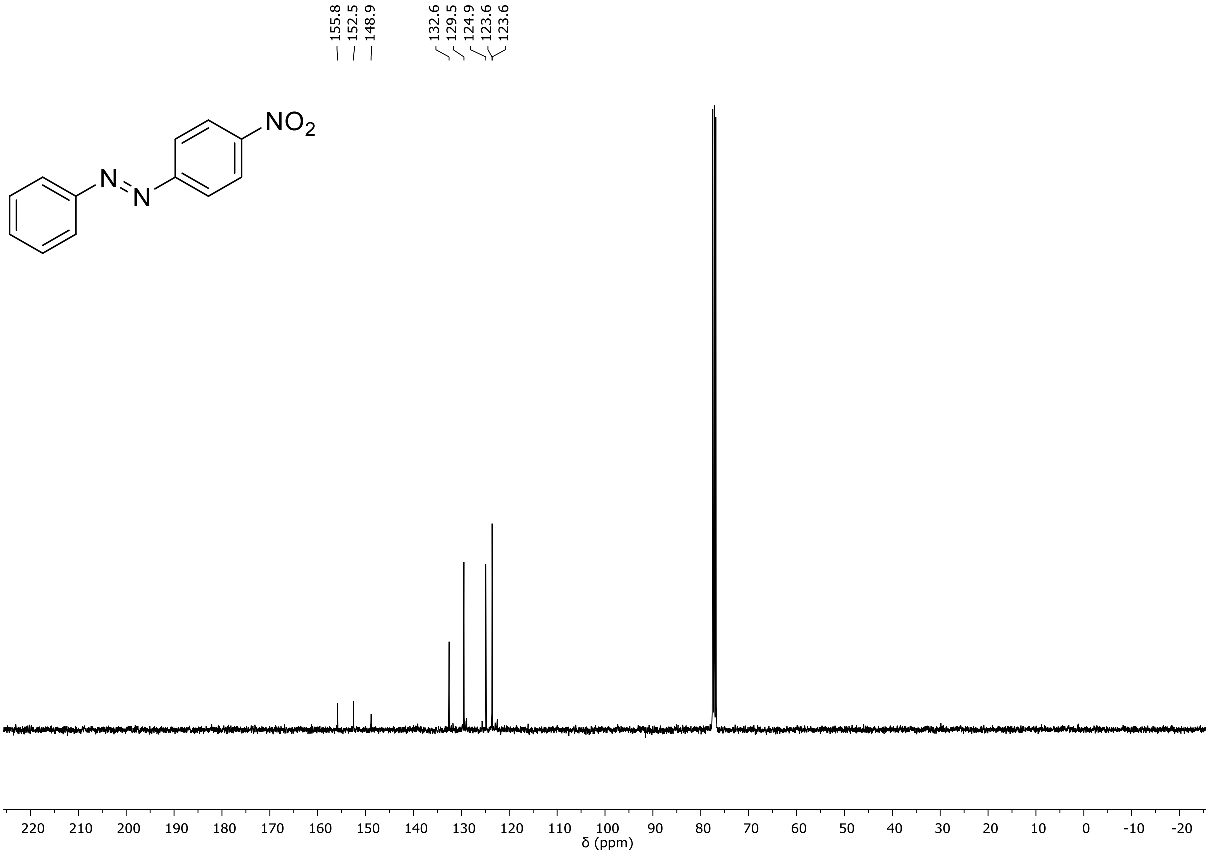

Figure S18. $^{13}C\{^{1}H\}$ NMR of 4-nitroazobenzene in $CDCl_3$ at 25 °C.

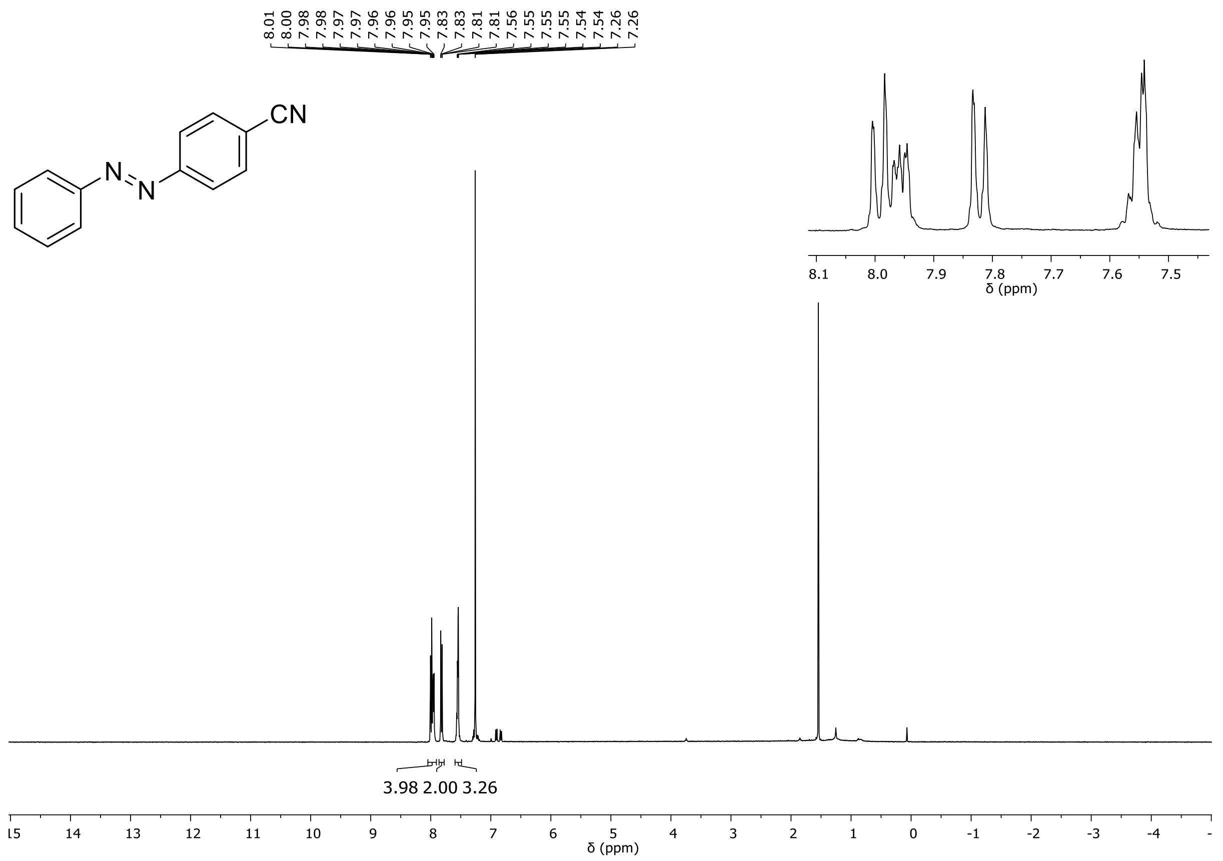

Figure S19. ^{1}H NMR of 4-cyanoazobenzene in $CDCl_3$ at 25 °C. Small amounts of the *Z*-isomer and grease are not peaked.

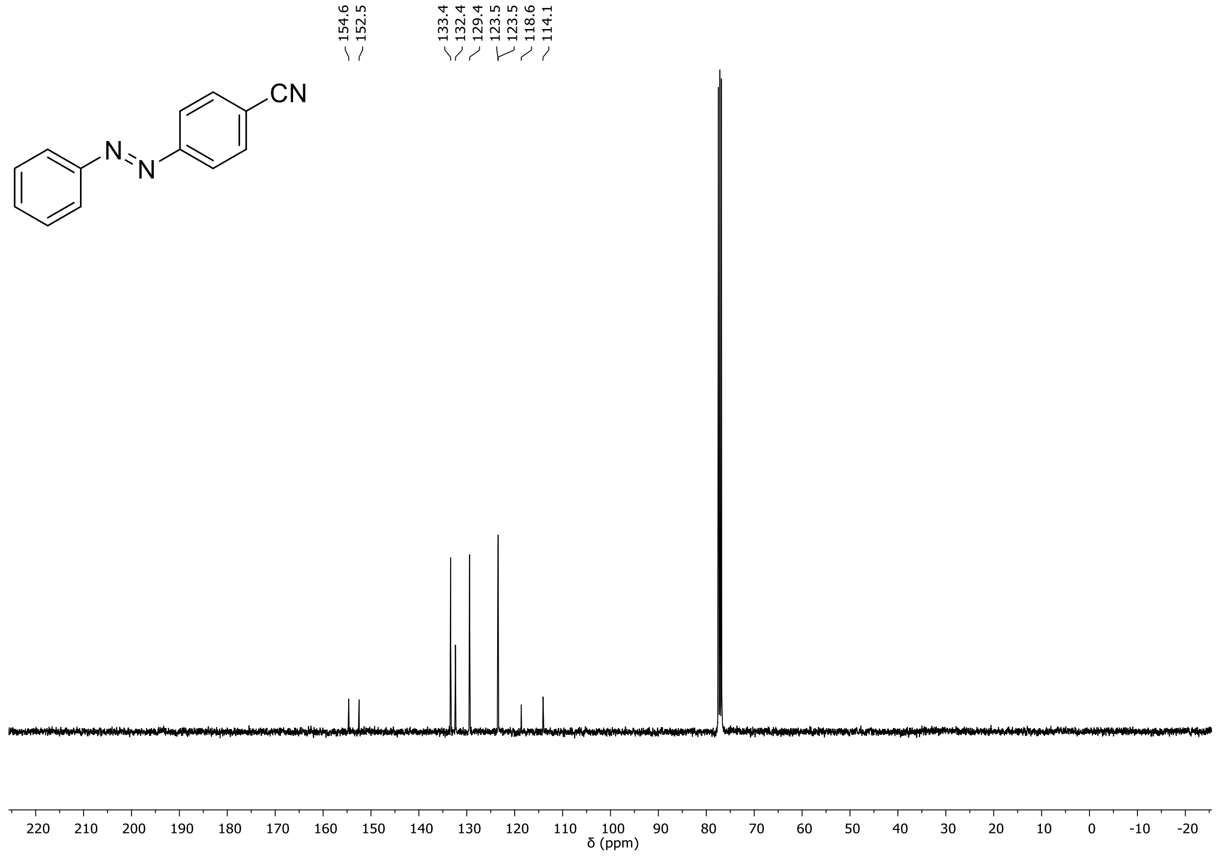

Figure S20. ^{13}C{^{1}H} NMR of 4-cyanoazobenzene in $CDCl_3$ at 25 °C.

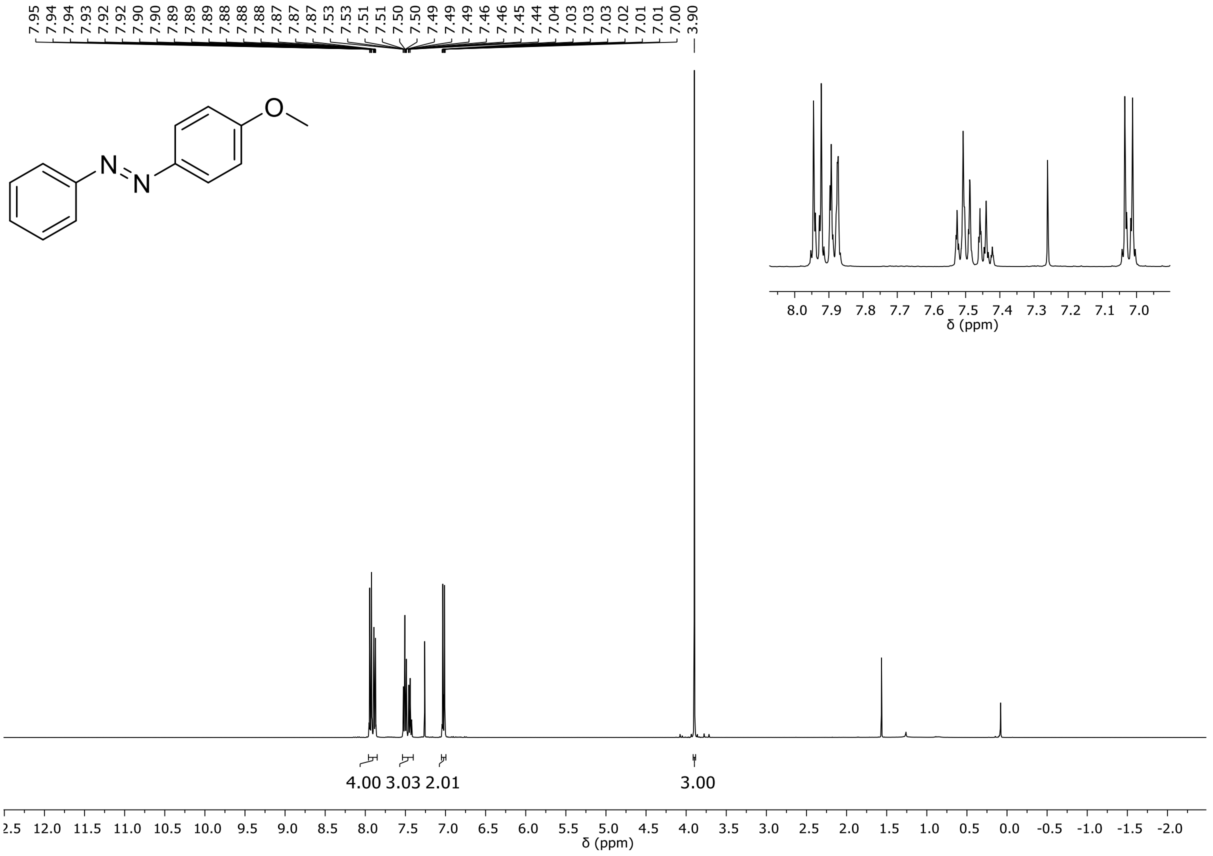

Figure S21. ^{1}H NMR of 4-methoxyazobenzene in $CDCl_3$ at 25 °C. Small amounts of grease are not peaked.

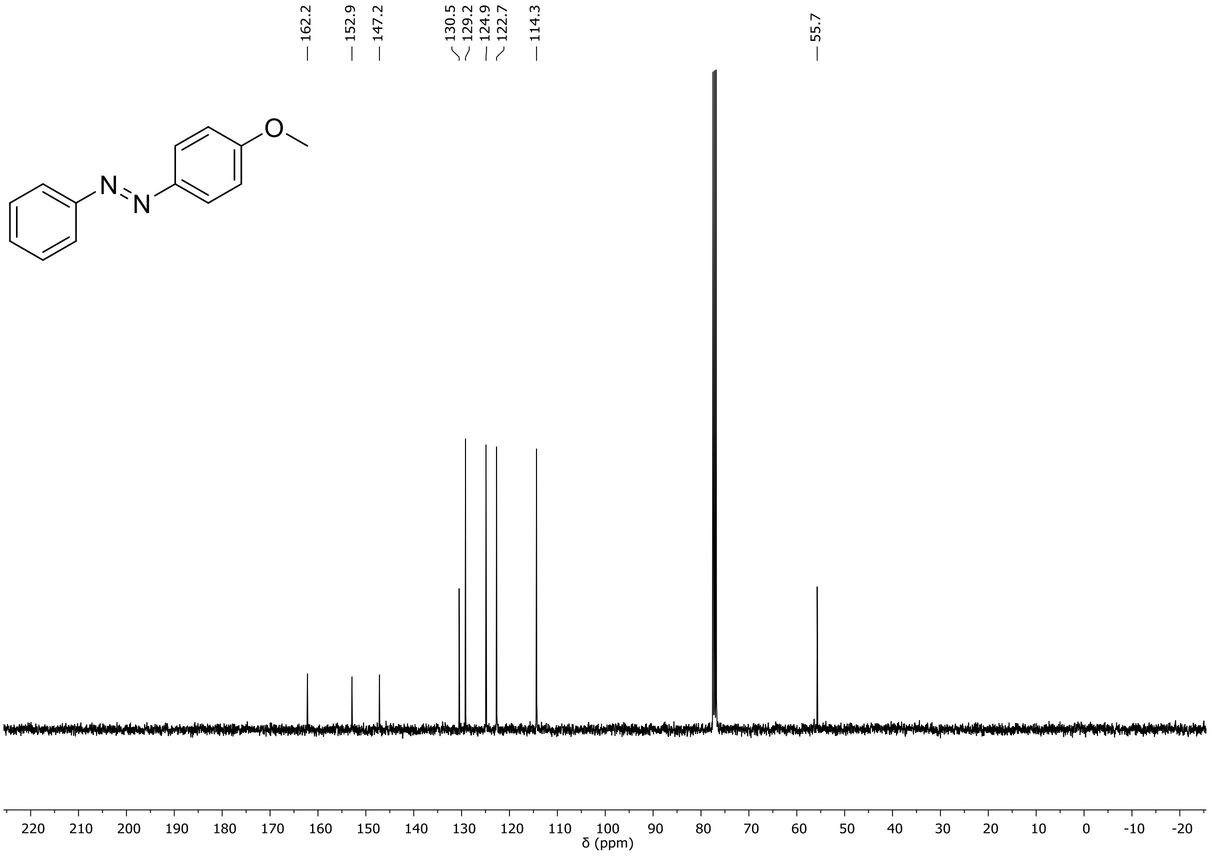

Figure S22. $^{13}C\{^{1}H\}$ NMR of 4-methoxyazobenzene in $CDCl_3$ at 25 °C.

3 Thermal Activation Analysis

3.1 Eyring Equation and Eyring Analysis

The Eyring equation (Eq. 3.1) is used to correlate the rate constants determined in Section 4 to the activation parameters.

$$k = \frac{\gamma\, k_B\, T}{h}\, e^{\frac{-\Delta G^{\ddagger}}{R\,T}} \tag{3.1}$$

By substituting Eq. 3.2 in Eq. 3.1, the most common form (Eq. 3.3) is obtained.

$$\Delta G^{\ddagger} = \Delta H^{\ddagger} - T\Delta S^{\ddagger} \tag{3.2}$$

$$k = \frac{\gamma\, k_B\, T}{h}\, e^{-\frac{\Delta H^{\ddagger}}{RT}+\frac{\Delta S^{\ddagger}}{R}} \tag{3.3}$$

Where k is the rate constant, γ the transmission coefficient, k_B the Boltzmann constant, T the temperature (in Kelvin), h the Planck constant, $\Delta G^{\ddagger}$ the Gibbs free energy of activation, $\Delta H^{\ddagger}$ the enthalpy of activation, $\Delta S^{\ddagger}$ the entropy of activation and R the gas constant.

The basis of the Eyring analysis is the Eyring equation (Eq. 3.3), which relates the reaction rate constant to the activation parameters. Most useful in its linearized form (Eq. 3.4), it enables the separation of the Gibbs free energy and determines the entropy of activation ($\Delta S^{\ddagger}$) and the enthalpy of activation ($\Delta H^{\ddagger}$). We assumed the transmission coefficient to be equal to 1.

$$ln\frac{k}{T} = \frac{-\Delta H^{\ddagger}}{RT} + ln\frac{k_B}{h} + \frac{\Delta S^{\ddagger}}{R} \tag{3.4}$$

Eyring plots (Section 5) are constructed by plotting $\ln\frac{k}{T}$ versus 1/T, where the rate constants are determined experimentally (Section 4). This allows us to perform linear regression on the obtained data to determine the enthalpy of activation from the negative slope $-\Delta H^{\ddagger}/R$ and the entropy of activation from the y-intercept $\ln(k_B/h)$ $\Delta S^{\ddagger}/R$ (Eq. 3.4). The obtained activation parameters and the corresponding values at 25 °C of the Gibbs free energy of activation, half-life and lifetimes are listed in Table 1 in the manuscript.

3.2 Rate Constants

A 20 mL solution of an azobenzene with A = ~1.0±0.2 was prepared. Three fluorescence cuvettes were filled up to the screw thread and closed. The three cuvettes were irradiated while stirring for 5 to 10 minutes. The cuvettes were placed in the multicell holder and equilibrated at the selected temperature in the 30-90 °C range (30, 39.5, 49.1, 58.6, 68.0, 77.6, 86.9 °C) while stirring for 2 minutes. Afterwards, the exponential decay at a fixed wavelength was recorded. The samples measured under argon were sparged with a positive argon stream for 15 minutes.

A home-built Python script was used to fit and plot all the exponential decays of the azobenzenes (see Section 4) to the following exponential function in Eq. 3.5 in which τ represents the lifetime, the inverse of the rate constant k.

$$y = A\, e^{\left(\frac{-x}{\tau}\right)} + y_0 \tag{3.5}$$

For each temperature, the kinetic constant was derived by taking the inverse of τ. The mean kinetic constants for each temperature, along with their associated errors, were then determined and used in the Eyring Analysis.

3.3 Error Analysis

3.3.1 Errors on the Rate Constant

The primary source of error in k is experimental errors. The UV-Vis absorption may be influenced by factors such as baseline drift or poor signal-to-noise ratios. These effects can be partially corrected for by performing a baseline correction on the spectra and ensuring constant reaction conditions (temperature).

To improve the overall quality of the exponential fit, the exponential process should be continued until it levels off, i.e., reaches a plateau.

Errors in the working temperature are minimized by measuring the temperature over a prolonged time period at the set temperature using an external thermocouple. To compensate for the time required for the sample to thermalize in the Cary5000, the initial data points at selected temperatures were excluded, for temperatures of 70, 80 and 90 °C, the first 60 data points, and for temperatures of 30, 40, 50 and 60 °C, the first 40 data points. Exclusion of these data points is based on the thermocouple measurements.

3.3.2 Errors on the Enthalpy and Entropy of Activation

The errors in the enthalpy and entropy are derived from plotting the experimentally determined rates against temperature and subsequently fitting them to the Eyring equation in its linearized form. Hence, the error in the temperature is relevant; inaccuracy in the measurement will plot erroneous datapoints in the 1/T values and will ultimately lead to poor fits. Therefore, the working temperatures were determined with an external thermocouple (measured temperatures: 30, 39.5, 49.1, 58.6, 68.0, 77.6 and 86.9 °C). For simplicity, we have reported the temperatures as 30, 40, 50, 60, 70, 80, and 90 °C, but for the plot, the exact measured temperatures were used. Errors can be further minimized by allowing the sample ample time to equilibrate. The remaining errors in the enthalpy and entropy result from the linear regression performed on the linearized Eyring equation (Eq. 3.4), where they are obtained as the fitting parameters. Linear regression and its error analysis are well established and are discussed in the next section.

3.3.3 Simple Linear Regression

For simple linear regression, the mathematical method of least-squares fitting is used to determine the best-fitting line through the dataset. The sum of the squared differences between the observed values y_i and the predicted values $\hat{y}_i$ generated by the linear model (Eq. 3.6) is minimized.

$$y = ax + b \tag{3.6}$$

Given the slope a and y-intercept b, the optimal values of a and b are determined by calculating the residuals (e_i) for each data point (Eq. 3.7).

$$e_i = y_i - \hat{y}_i \tag{3.7}$$

The residuals are squared and summed to obtain the residual sum of squares (RSS), where n is the total number of observations. The coefficients a and b are adjusted to minimize the RSS, providing the best-fitting line.

$$RSS = \sum_{i=1}^{n} (y_i - \hat{y}_i)^2 \tag{3.8}$$

The goodness of fit is assessed by analyzing the standard error of regression (Eq. 3.9) and the coefficient of determination, R^2 (Eq. 3.11).

$$s = \sqrt{\frac{\sum_{i=1}^{n} (y_i - \hat{y}_i)^2}{n-k}} = \sqrt{\frac{\sum_{i=1}^{n} (y_i - \hat{y}_i)^2}{n-2}} = \sqrt{\frac{RSS}{n-2}} \tag{3.9}$$

In Eq. 3.9, y_i is the observed value for the ith observation, $\hat{y}_i$ is the predicted value for the ith observation, n is the total number of observations, and n-k are the residual degrees of freedom, accounting for the number of estimated parameters k (k = 2, intercept and slope). This is almost identical to the root mean square error (RMSE), Eq. 3.10, in which there is no adjustment for the reduced degrees of freedom.

$$RMSE = \sqrt{\frac{\sum_{i=1}^{n} (y_i - \hat{y}_i)^2}{n}} = \sqrt{\frac{RSS}{n}} = \sqrt{MSE} \tag{3.10}$$

Which leads to the equation for R^2

$$R^2 = 1 - \frac{\sum_{i=1}^{n} (y_i - \hat{y}_i)^2}{\sum_{i=1}^{n} (y_i - \bar{y})^2} = 1 - \frac{RSS}{TSS} \tag{3.11}$$

Where ȳ is the mean of the observed values, and $\sum_{i=1}^{n}(y_i - \bar{y})^2$, is the total sum of squares (TSS). s gives a measure of the typical size of the prediction error, whereas R^2 indicates how well the model fits the data.

The standard errors (SEs) for the parameters a and b are given by equations 3.12 and 3.13.

$$SE_a = \frac{s}{\sqrt{\sum_{i=1}^{n}(x_i - \bar{x})^2}} = \sqrt{\frac{1}{n-2} - \frac{\sum_{i=1}^{n}(y_i - \hat{y}_i)^2}{\sum_{i=1}^{n}(x_i - \bar{x})^2}} \tag{3.12}$$

$$SE_b = s\sqrt{\frac{1}{n}\frac{x^2}{\sum_{i=1}^{n}(x_i - \bar{x})^2}} = \sqrt{\frac{\sum_{i=1}^{n}(y_i - \hat{y}_i)^2}{n-2}}\sqrt{\frac{1}{n}\frac{x^2}{\sum_{i=1}^{n}(x_i - \bar{x})^2}} \tag{3.13}$$

In these expressions, s is the standard error of the regression (Eq. 3.9), x_i is the observed value for the ith observation of the independent variable, x is their mean, y_i is the observed value for the ith observation of the dependent variable, $\hat{y}_i$ is the predicted value for the i^{th}observation of the dependent variable, n is the total number of observations, and MSE is the mean square error.

In the Eyring analysis using the linearized Eyring equation (Eq. 3.4), the parameters a and b in the formula y = ax + b are substituted by a = $-\Delta H^{\ddagger}/R$, x = 1/T and b = $\Delta S^{\ddagger}/R$. Therefore, the outcomes of Eq. 3.12 and 3.13 need to be multiplied by R to obtain the errors on $\Delta H^{\ddagger}$ and $\Delta S^{\ddagger}$.

3.3.4 The error in the Fitting Parameter and the Gibbs free Energy

The errors on the fitting parameters can be obtained by taking the square root of the diagonal elements in the variance-covariance matrix from the fitting (Eq. 3.14–16).

$$\sigma_a = \sqrt{variance[1,1]} \tag{3.14}$$

$$\sigma_b = \sqrt{variance[0,0]} \tag{3.15}$$

$$\sigma_{\Delta H \Delta S} = \sigma_{\Delta H} \sigma_{\Delta S} \rho_{\Delta H \Delta S} \tag{3.16}$$

And these are propagated to calculate the error on the fitting parameters by multiplying by R. (Eq. 3.17 & 3.18).

$$\sigma_{\Delta H^{\ddagger}} = \sigma_a * R \tag{3.17}$$

$$\sigma_{\Delta S^{\ddagger}} = \sigma_b * R \tag{3.18}$$

When the Gibbs free energy is calculated from the enthalpy and entropy of activation according to Eq. 3.2, the errors obtained for $\Delta H^{\ddagger}$ and $\Delta S^{\ddagger}$ directly propagate into the error in $\Delta G^{\ddagger}$ as a function of those parameters. It follows from its standard formula that the standard deviation σ on $\Delta H^{\ddagger}$ and $\Delta S^{\ddagger}$ propagate according to Eq. 3.19.

$$\sigma_{\Delta G} = \sqrt{\sigma_{\Delta G}^2 + T^2 \sigma_{\Delta S}^2 - 2T\sigma_{\Delta H \Delta S}} \tag{3.19}$$

Where $\sigma_{\Delta H \Delta S}$ is the covariance between the entropy and enthalpy of activation (Eq. 3.16), where ρ = 1 because $\Delta H^{\ddagger}$ and $\Delta S^{\ddagger}$ perfectly correlate.

4 Thermal Kinetics

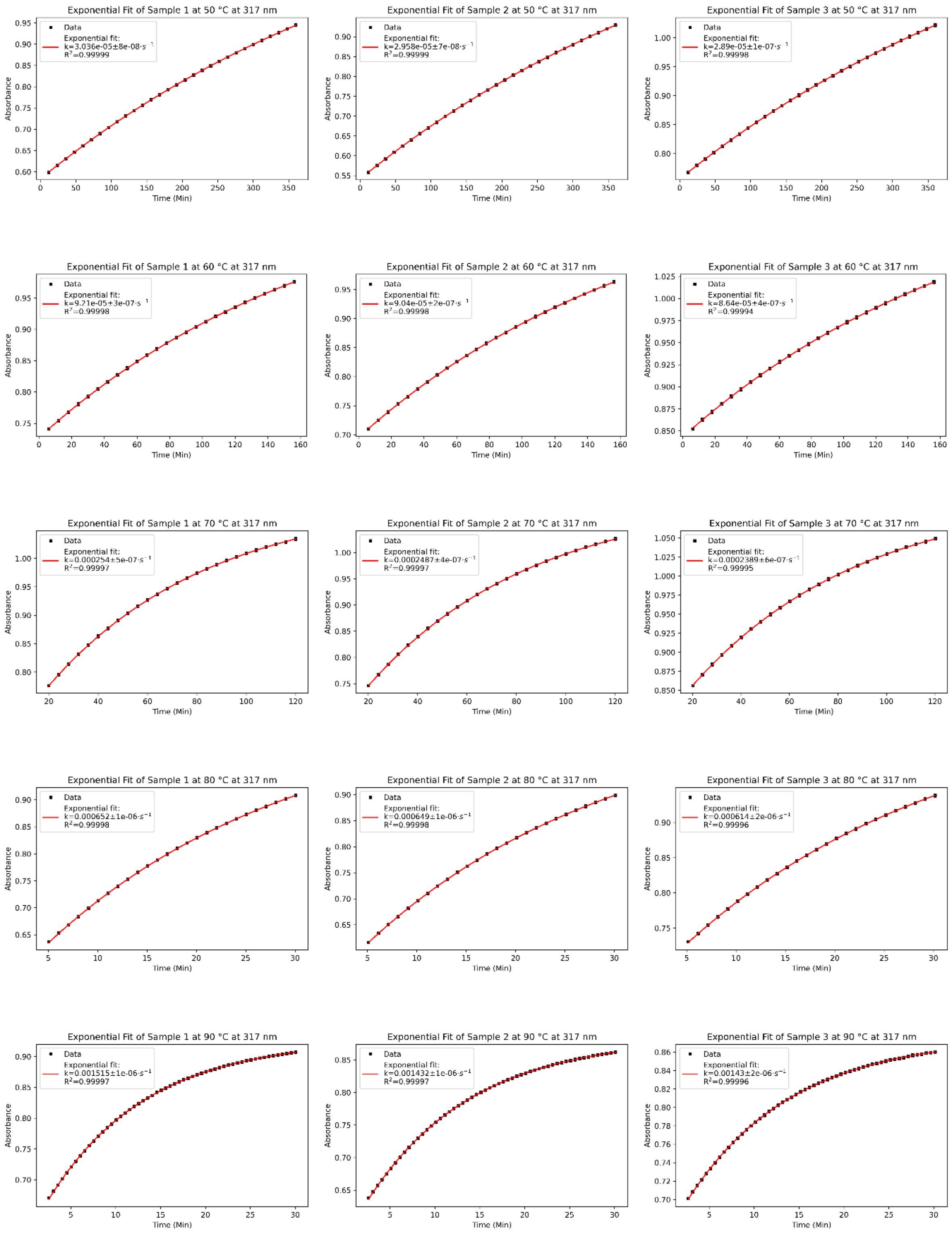

Figure S23. The kinetic traces were recorded at λ = 317 nm in triplicate for azobenzene at temperatures of 50, 60, 70, 80, and 90 °C. Irradiated with a 340 nm LED.

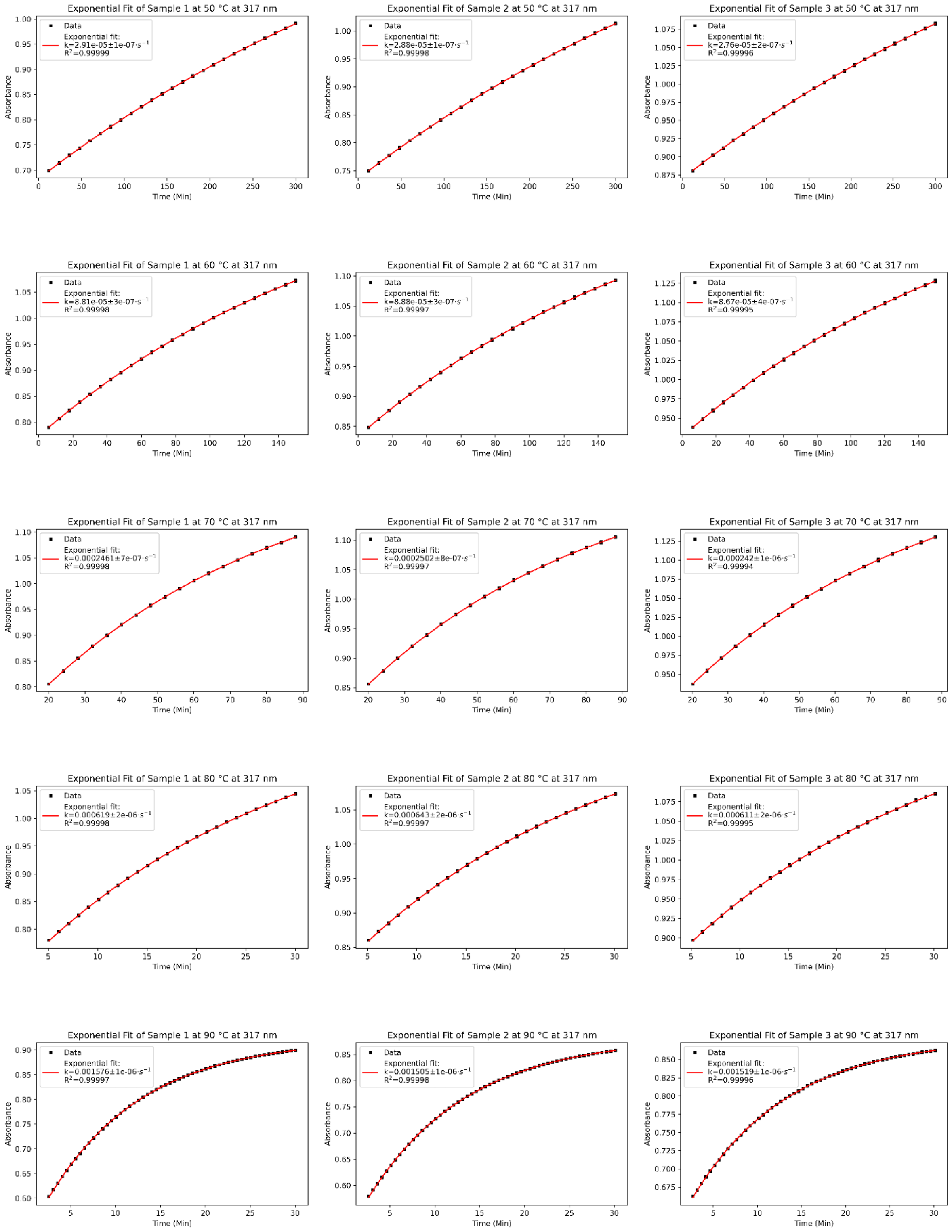

Figure S24. The kinetic traces were recorded at λ = 317 nm in triplicate for azobenzene under argon at temperatures of 50, 60, 70, 80, and 90 °C. Irradiated with a 340 nm LED.

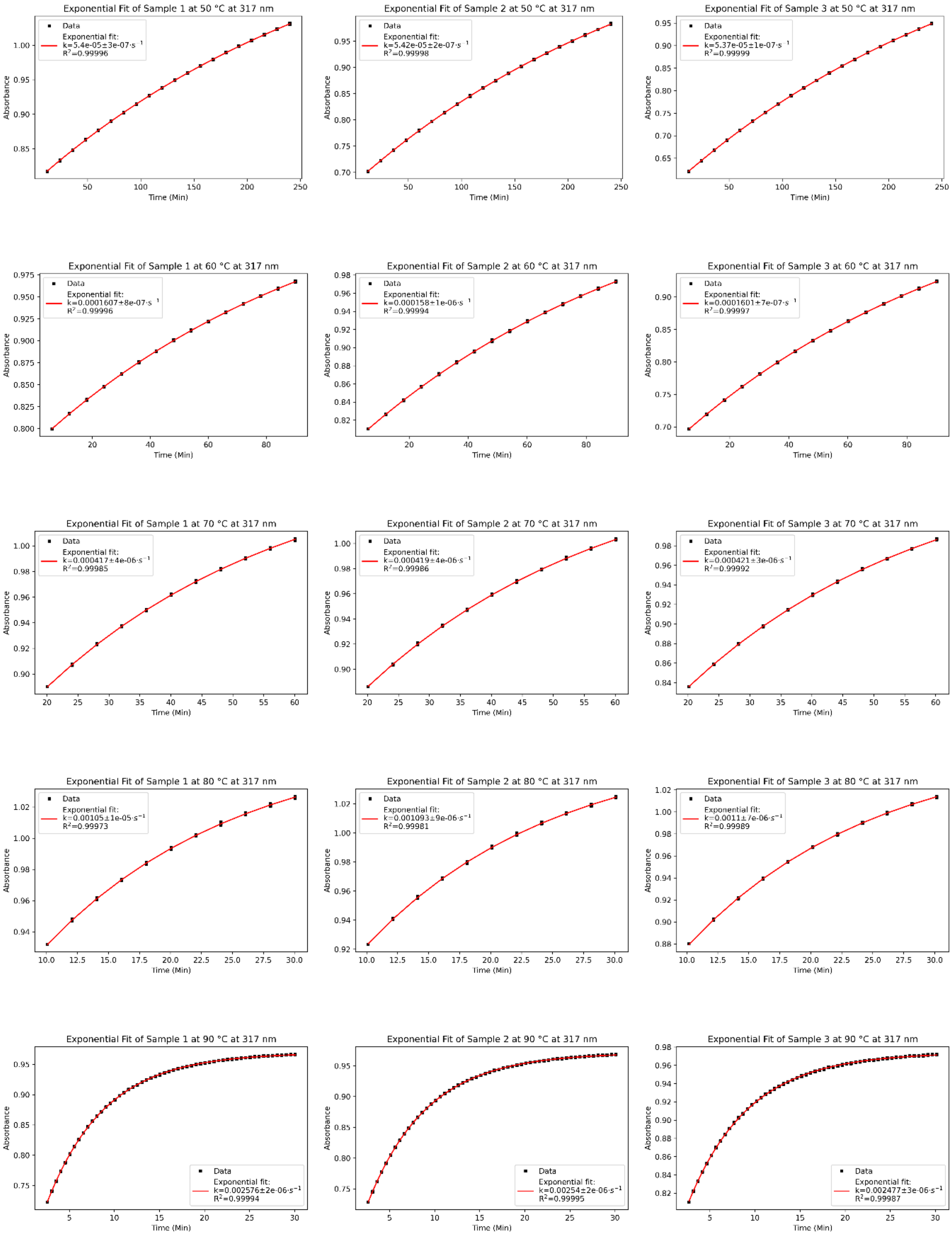

Figure S25. The kinetic traces were recorded at λ = 317 nm in triplicate for 4-(*tert*-butyl)azobenzene at temperatures of 50, 60, 70, 80, and 90 °C. Irradiated with a 340 nm LED.

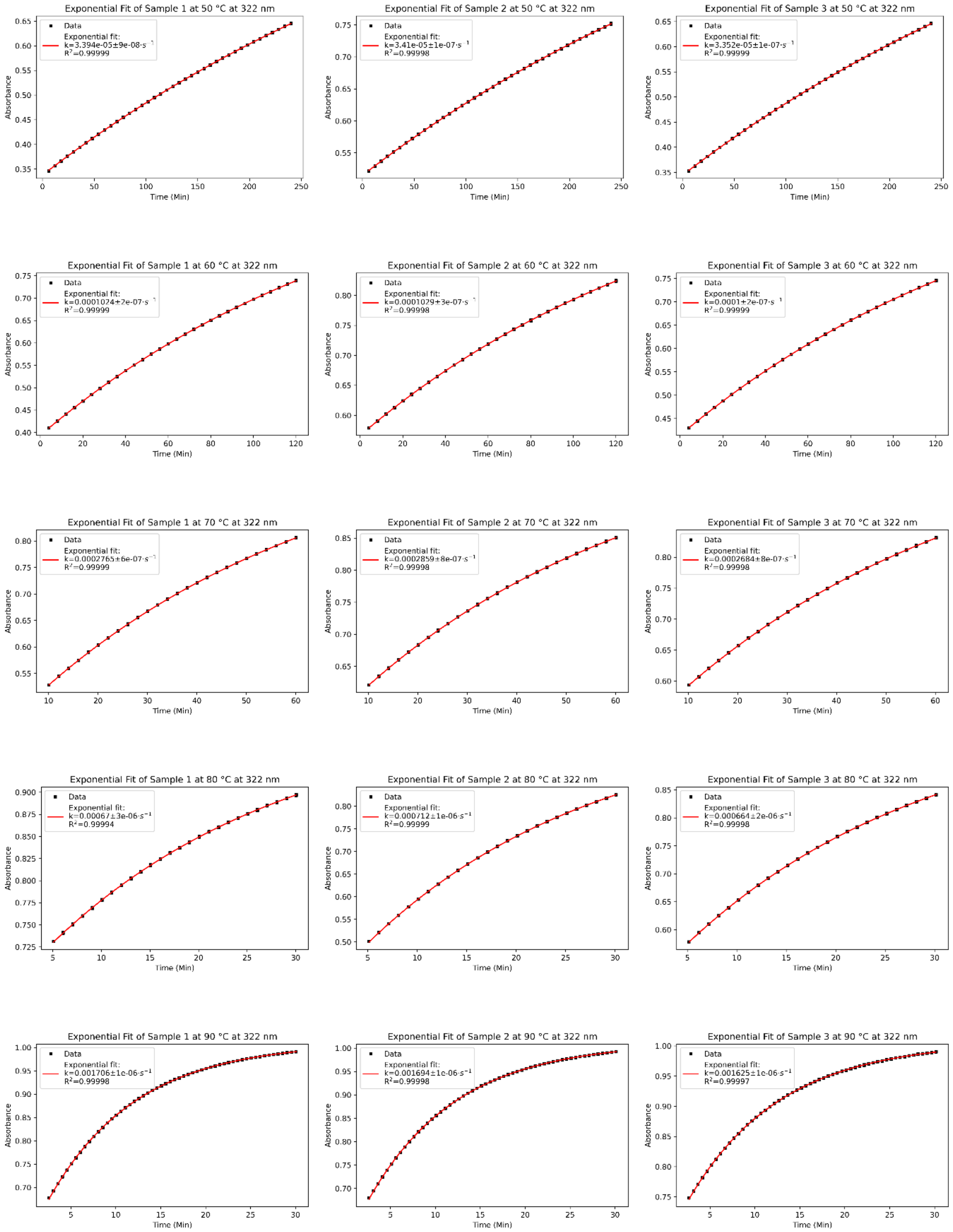

Figure S26. The kinetic traces were recorded at λ = 322 nm in triplicate for 4-fluoroazobenzene at temperatures of 50, 60, 70, 80, and 90 °C. Irradiated with a 340 nm LED.

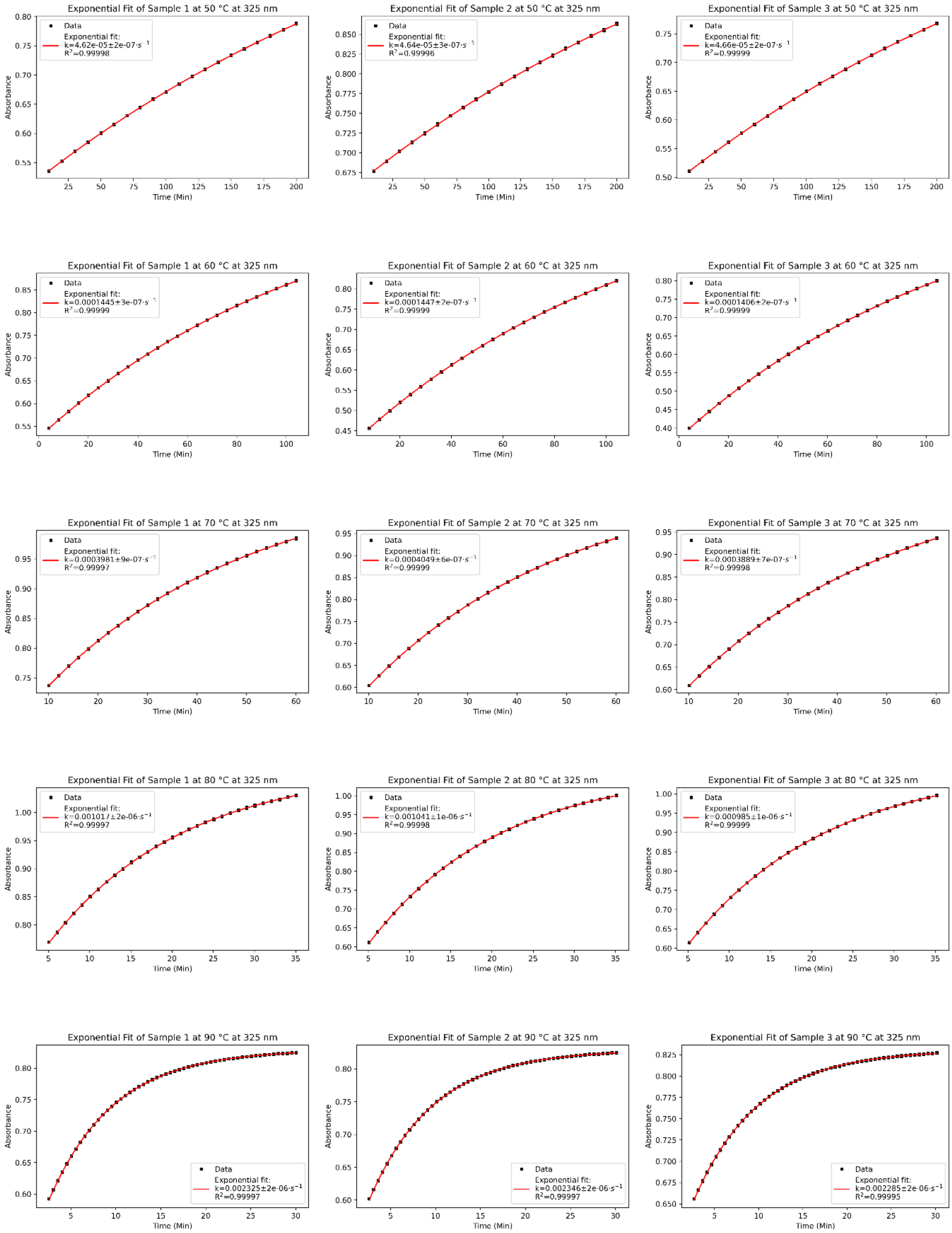

Figure S27. The kinetic traces were recorded at λ = 325 nm in triplicate for 4-chloroazobenzene at temperatures of 50, 60, 70, 80, and 90 °C. Irradiated with a 340 nm LED.

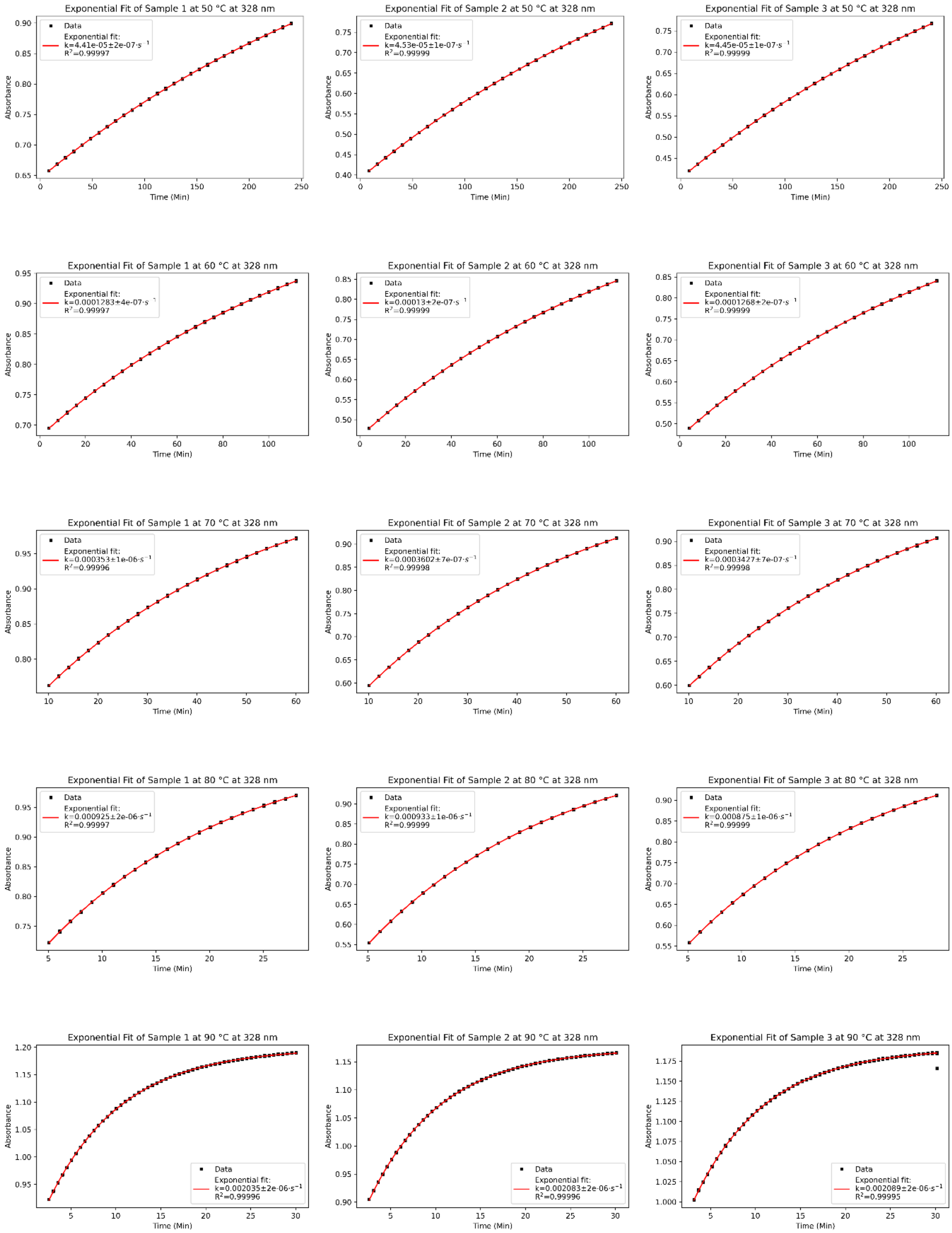

Figure S28. The kinetic traces were recorded at λ = 328 nm in triplicate for 4-bromoazobenzene at temperatures of 50, 60, 70, 80, and 90 °C. Irradiated with a 340 nm LED.

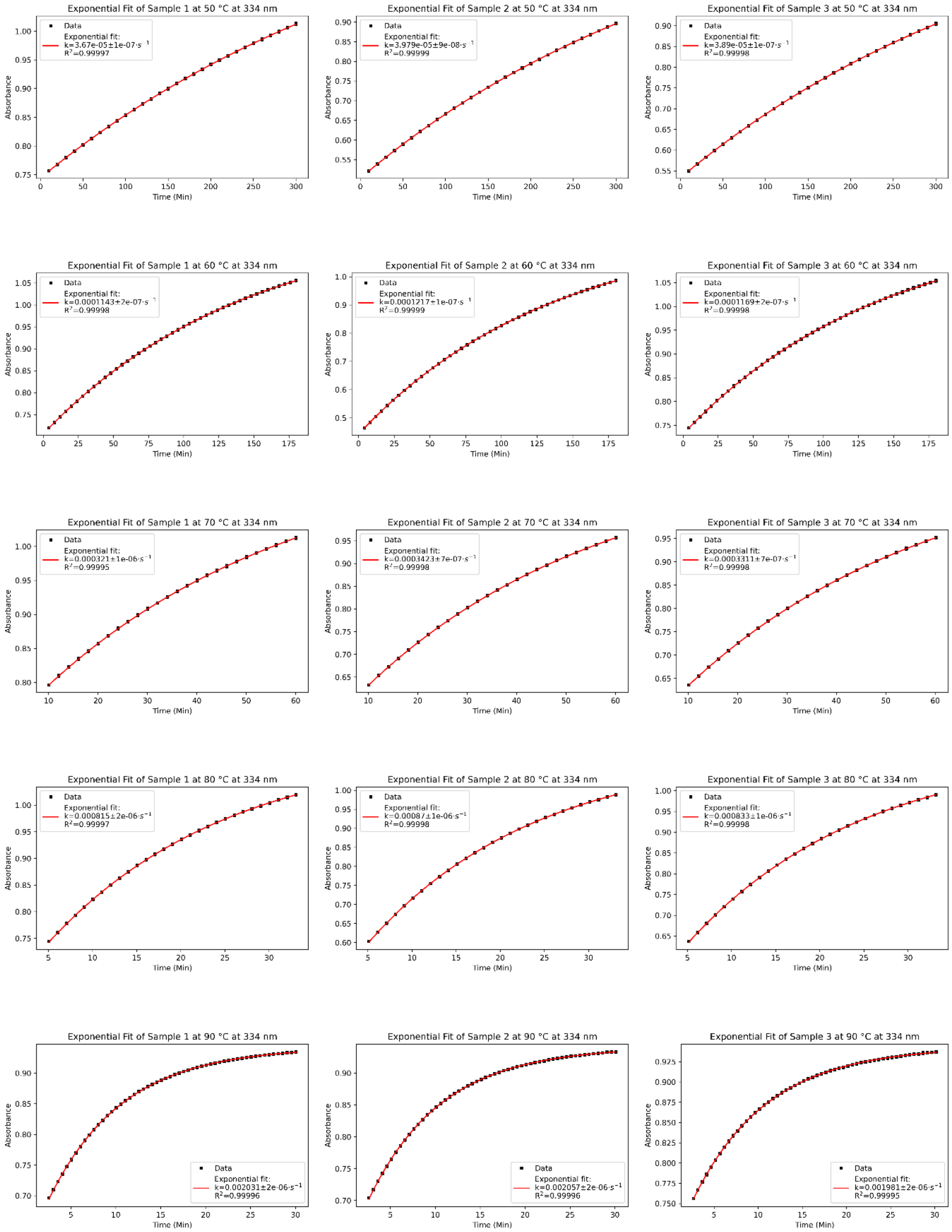

Figure S29. The kinetic traces were recorded at λ = 334 nm in triplicate for 4-iodoazobenzene at temperatures of 50, 60, 70, 80, and 90 °C. Irradiated with a 340 nm LED.

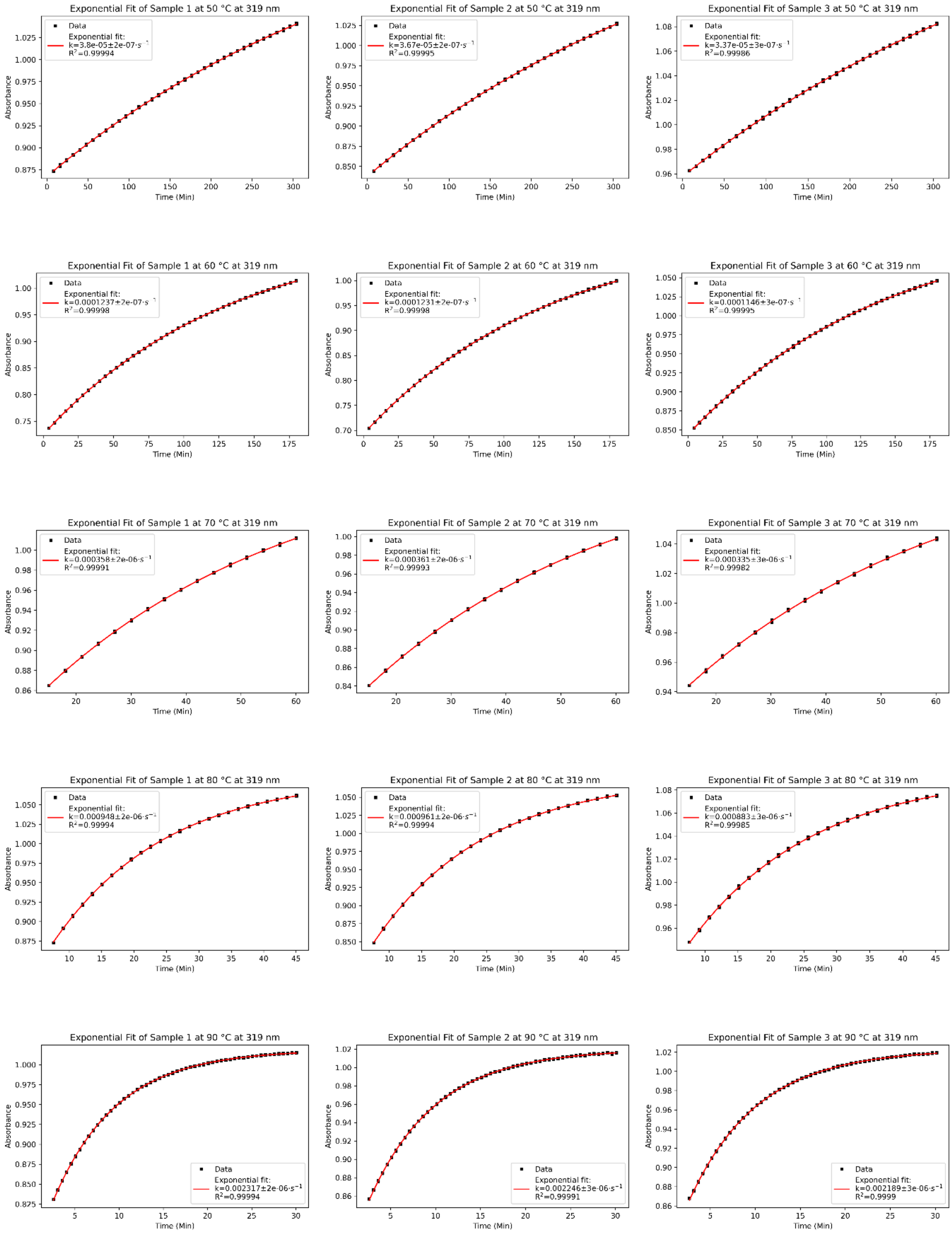

Figure S30. The kinetic traces were recorded at λ = 319 nm in triplicate for 4-(trifluoromethyl)azobenzene at temperatures of 50, 60, 70, 80, and 90 °C. Irradiated with a 340 nm LED.

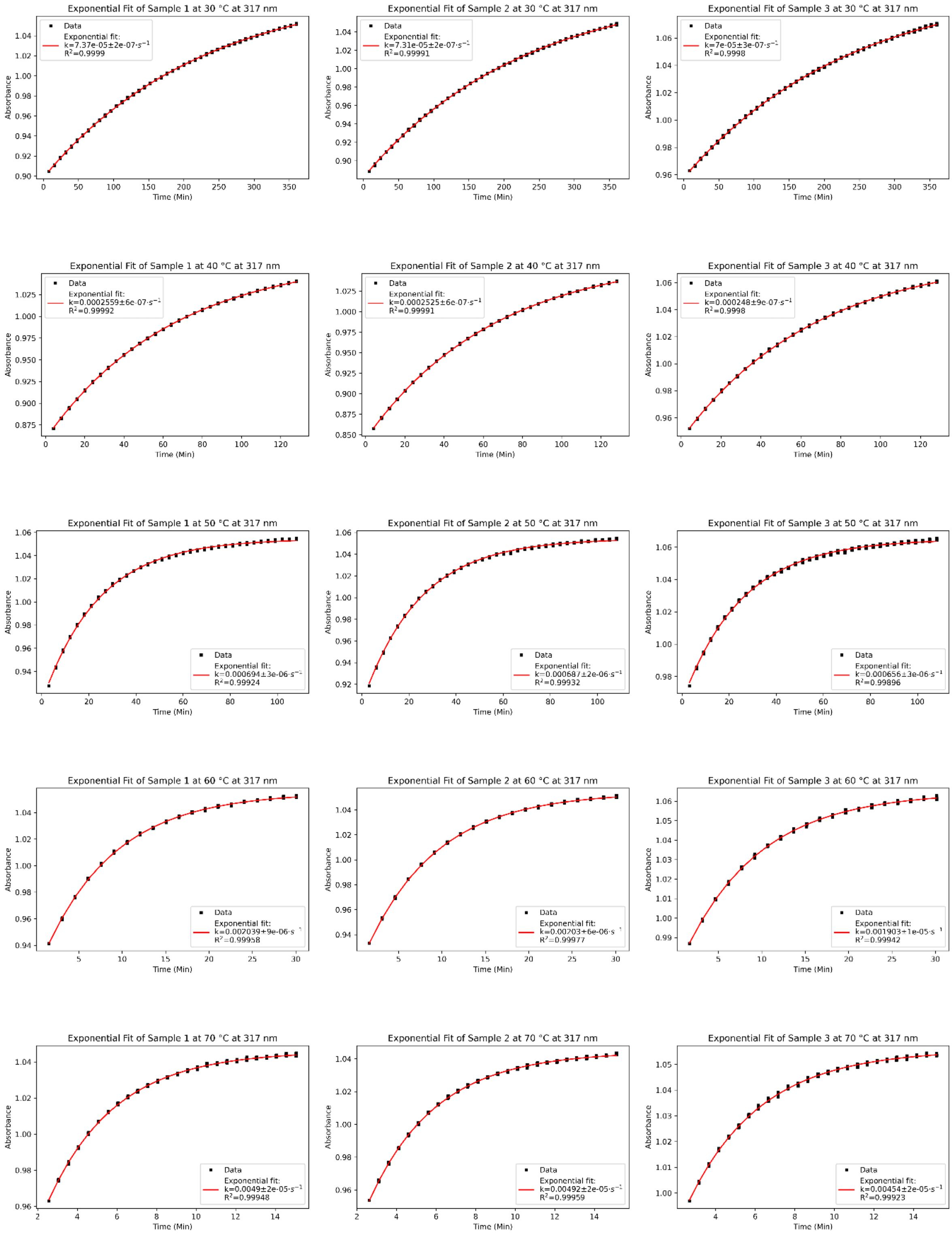

Figure S31. The kinetic traces were recorded at λ = 317 nm in triplicate for 4-nitroazobenzene at temperatures of 30, 40, 50, 60, and 70 °C. Irradiated with a 340 nm LED.

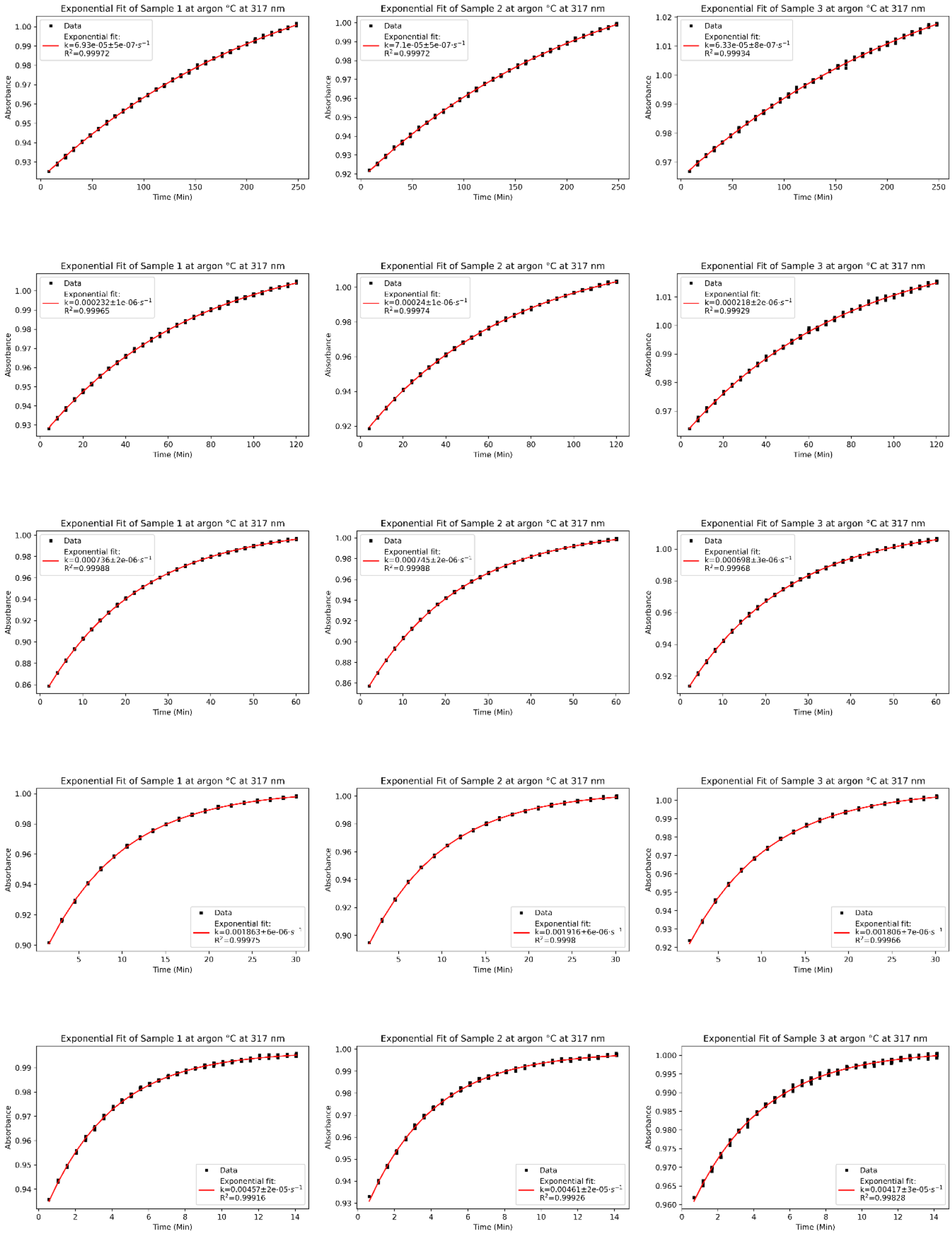

Figure S32. The kinetic traces were recorded at λ = 317 nm in triplicate for 4-nitroazobenzene under argon at temperatures of 30, 40, 50, 60, and 70 °C. Irradiated with a 340 nm LED.

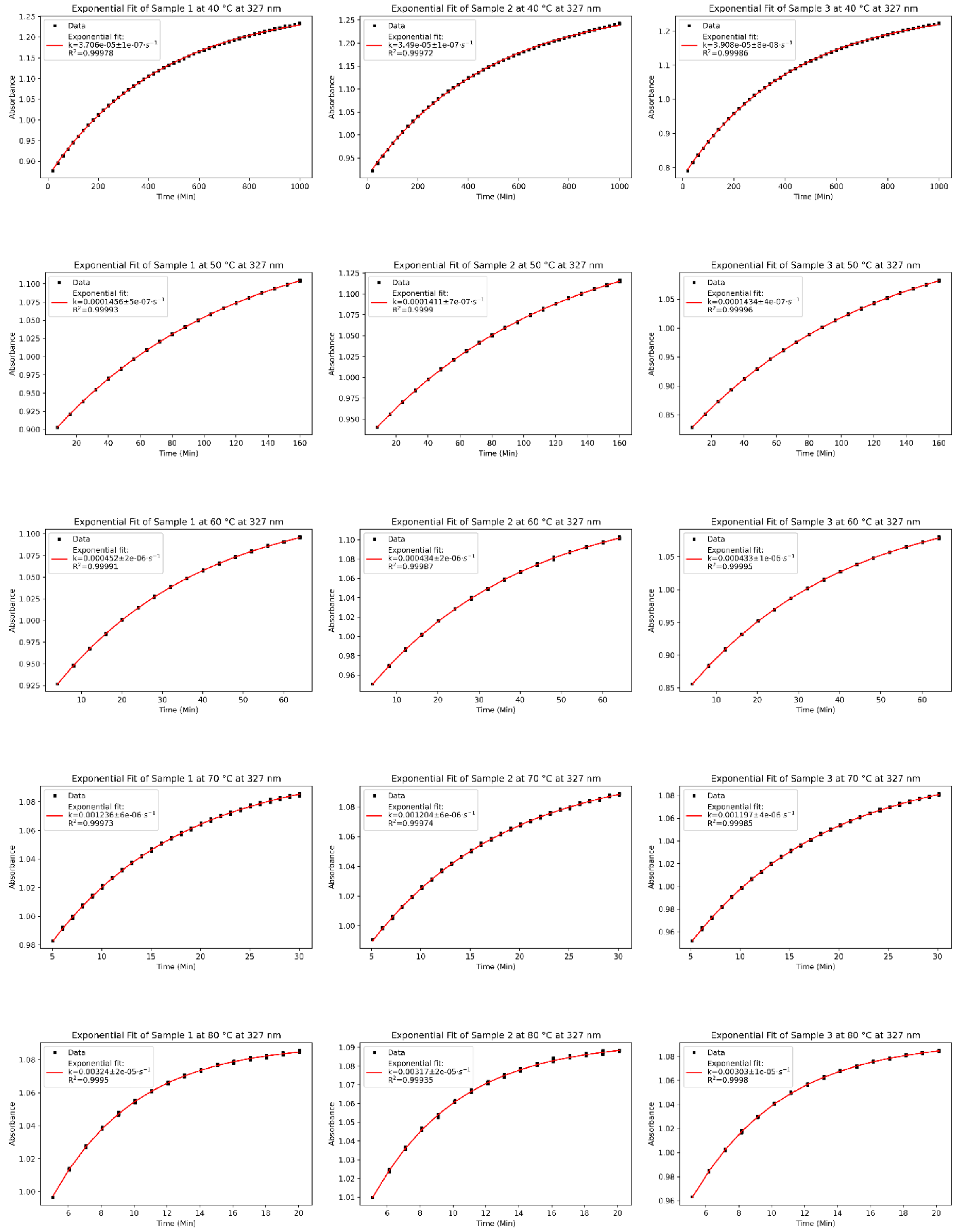

Figure S33. The kinetic traces were recorded at λ = 327 nm in triplicate for 4-cyanoazobenzene at temperatures of 40, 50, 60, 70, and 80 °C. Irradiated with a 340 nm LED.

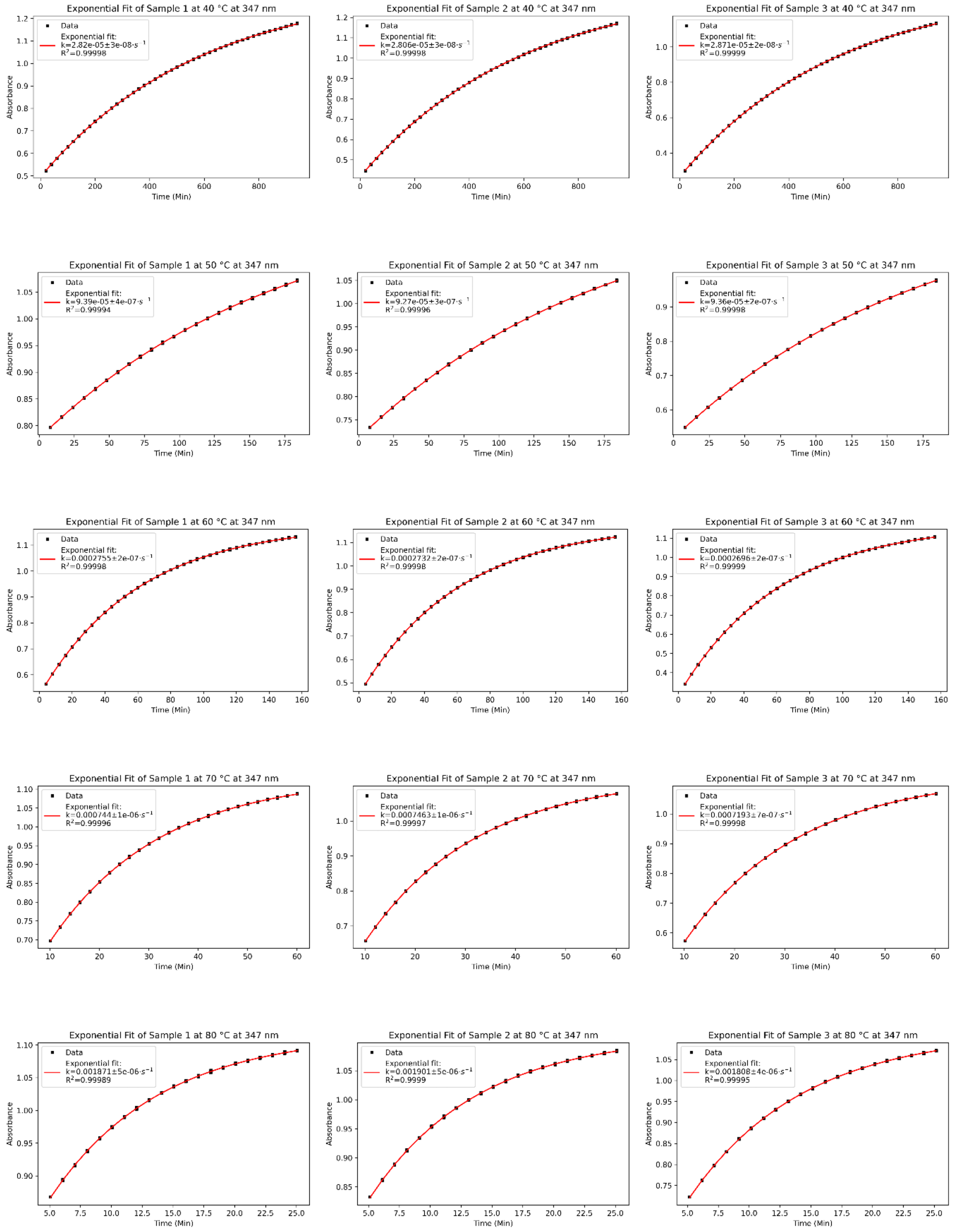

Figure S34. The kinetic traces were recorded at λ = 347 nm in triplicate for 4-methoxyazobenzene at temperatures of 40, 50, 60, 70, and 80 °C. Irradiated with a 340 nm LED.

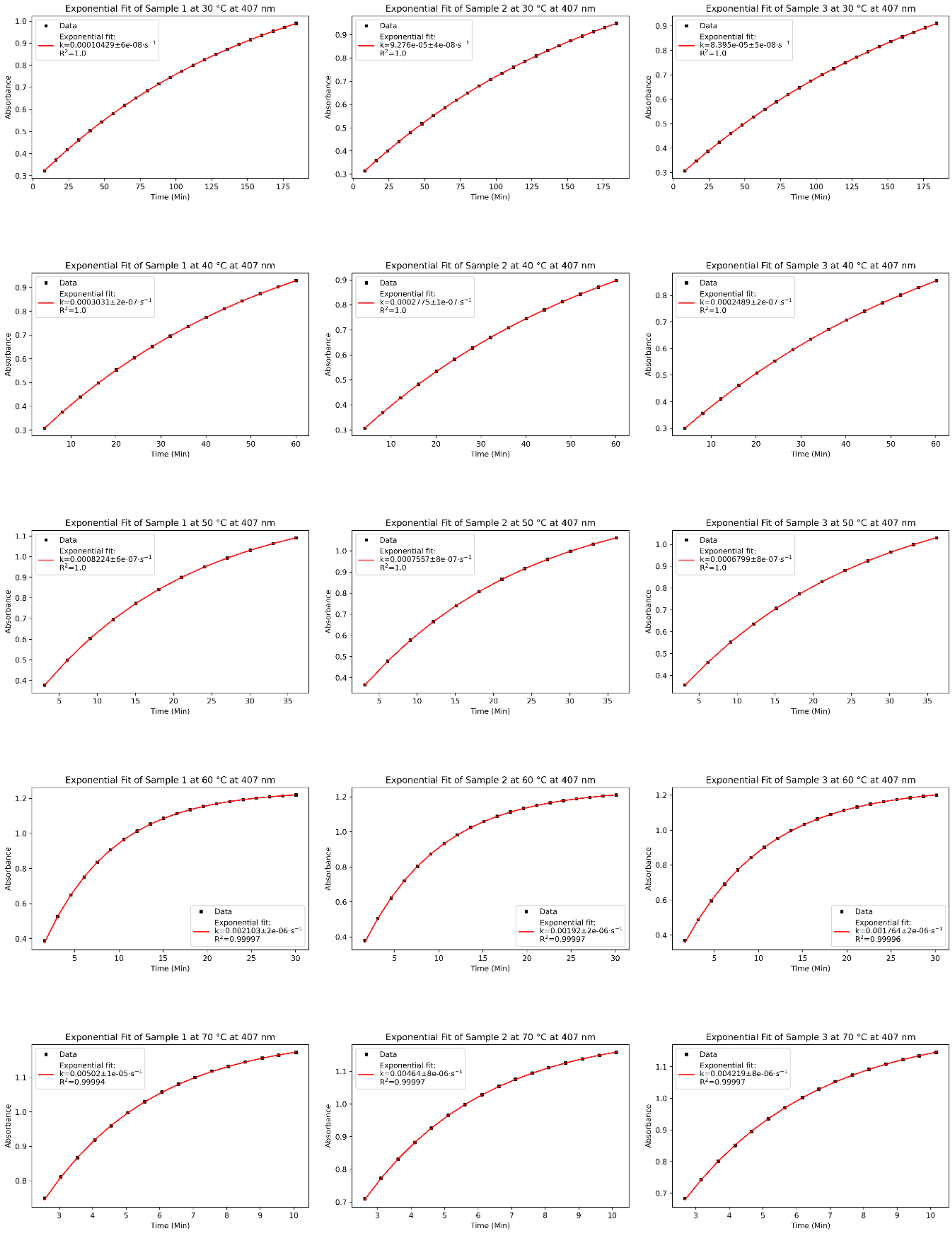

Figure S35. The kinetic traces were recorded at λ = 407 nm in triplicate for 4-(dimethylamino)azobenzene at temperatures of 30, 40, 50, 60 and 70 °C. Irradiated with a 395 nm LED.

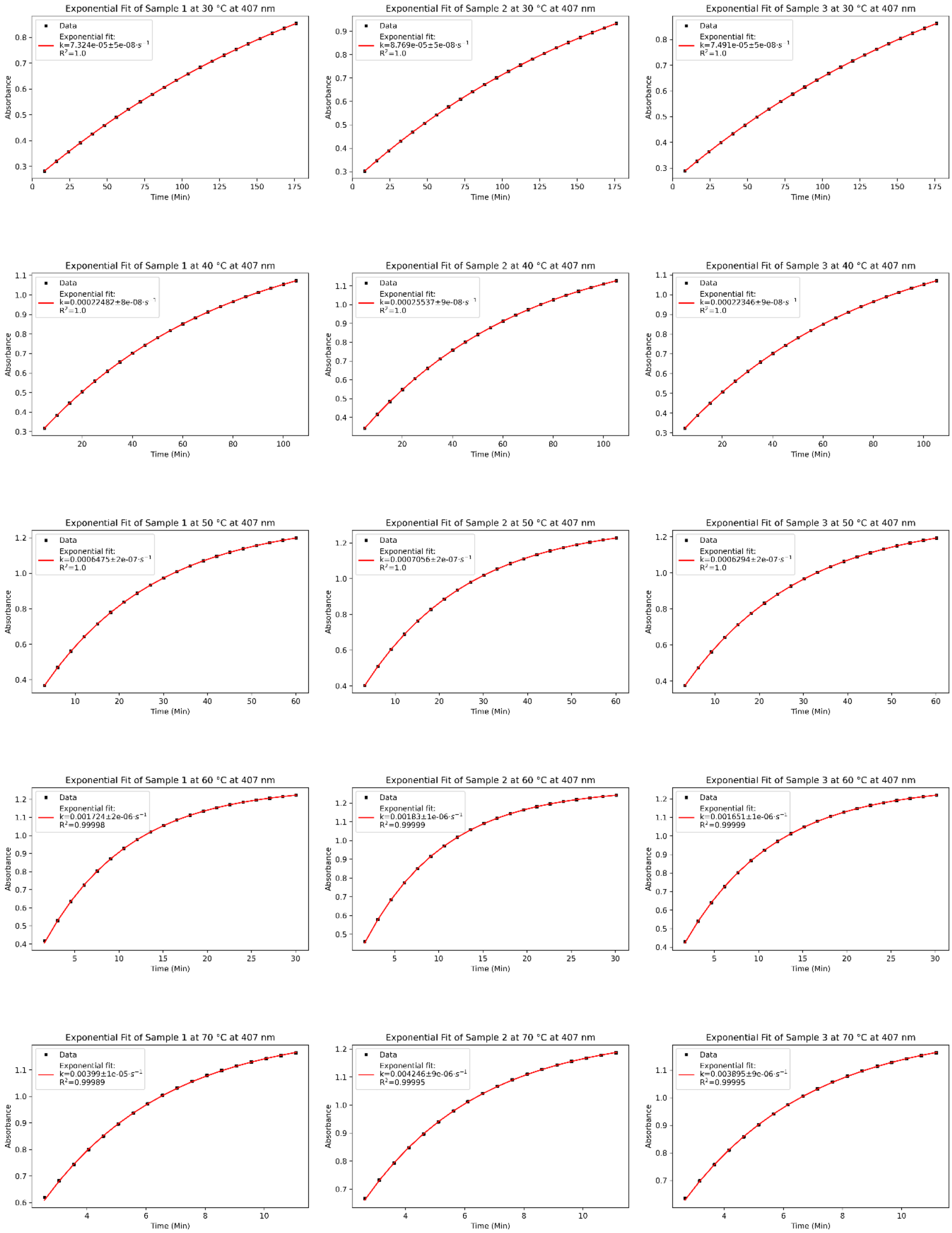

Figure S36. The kinetic traces were recorded at λ = 407 nm in triplicate for 4-(dimethylamino)azobenzene under argon at temperatures of 30, 40, 50, 60 and 70 °C. Irradiated with a 395 nm LED.

5 Eyring Plots

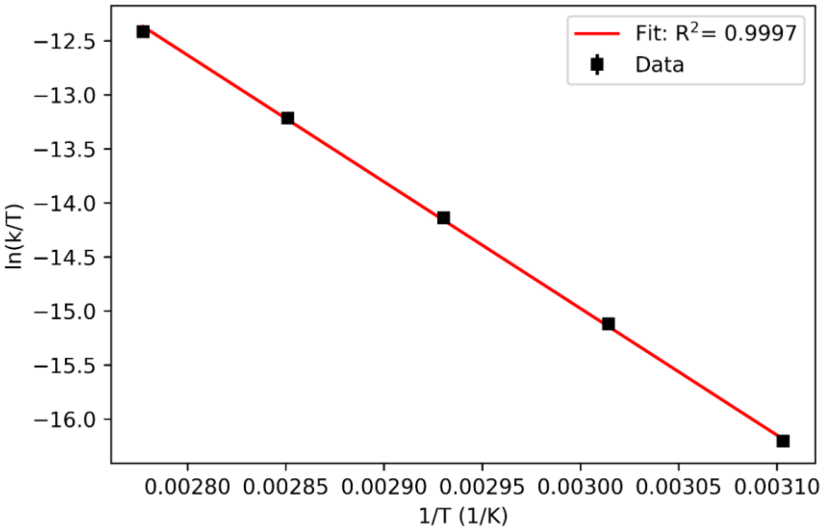

Figure S37. Eyring plot for azobenzene.

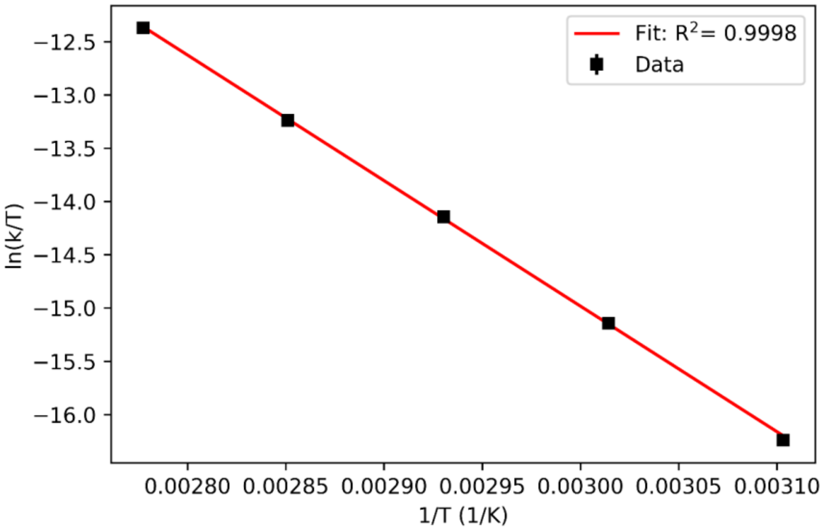

Figure S38. Eyring plot for azobenzene under argon.

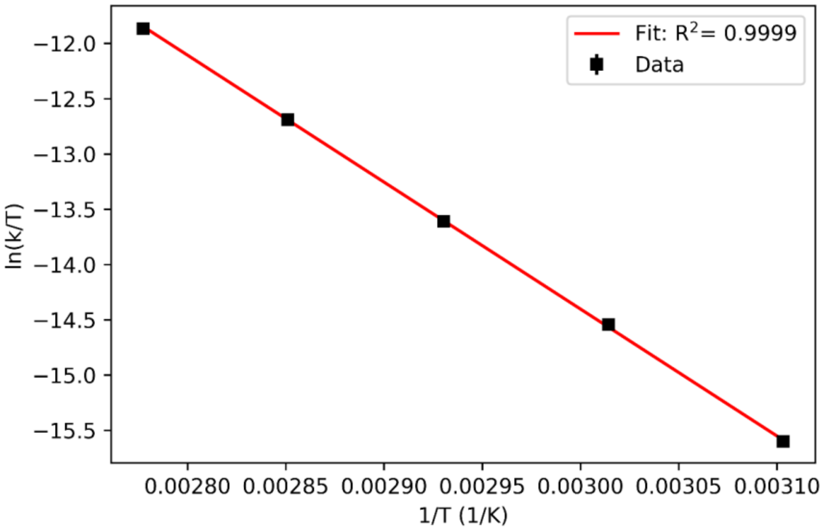

Figure S39. Eyring plot for 4-(*tert*-butyl)azobenzene.

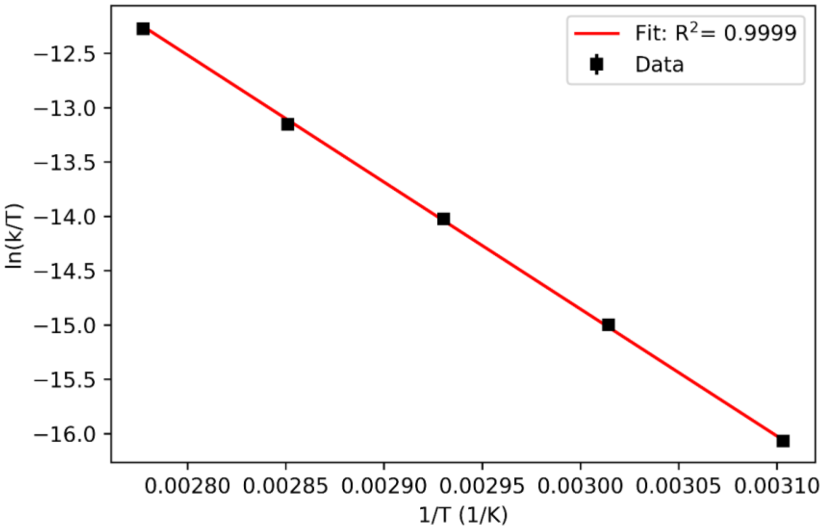

Figure S40. Eyring plot for 4-fluoroazobenzene.

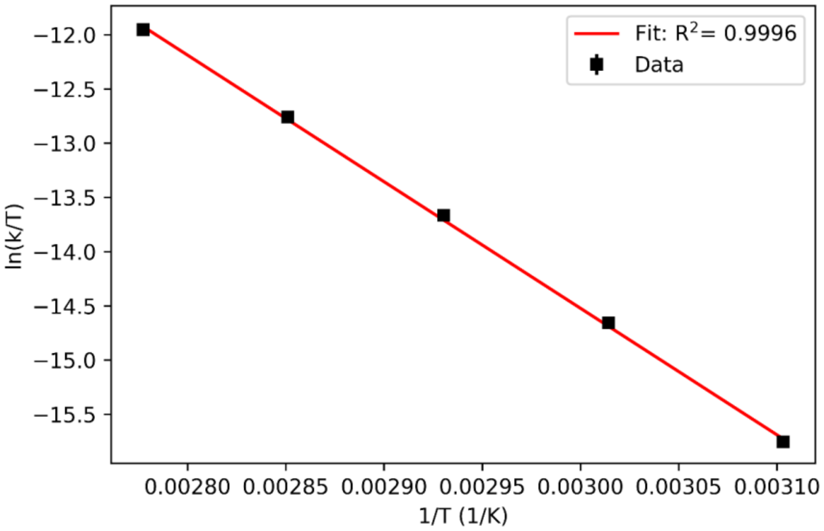

Figure S41. Eyring plot for 4-chloroazobenzene.

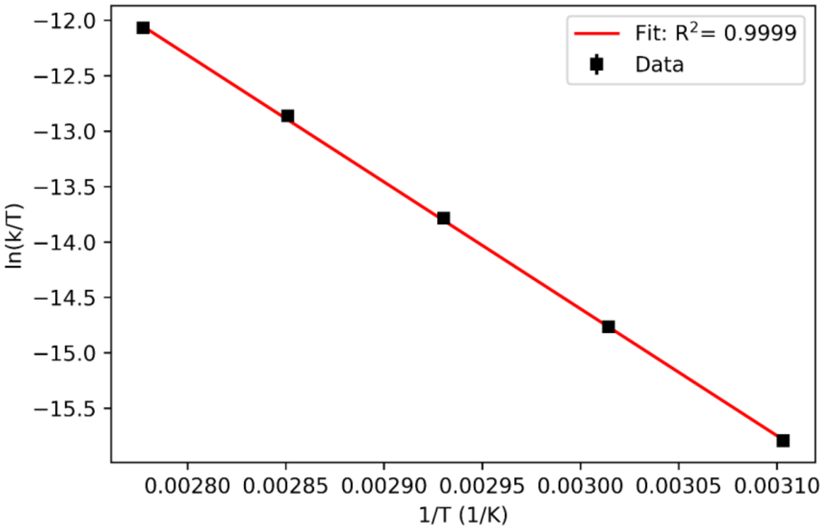

Figure S42. Eyring plot for 4-bromoazobenzene.

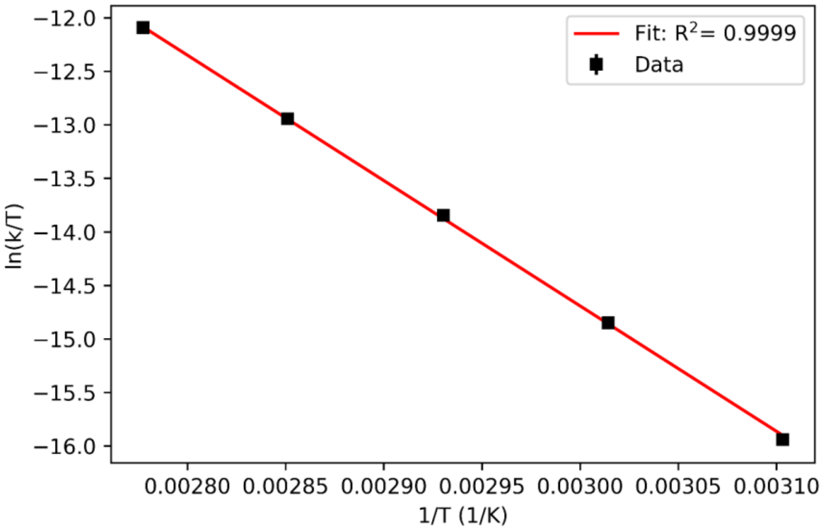

Figure S43. Eyring plot for 4-iodoazobenzene.

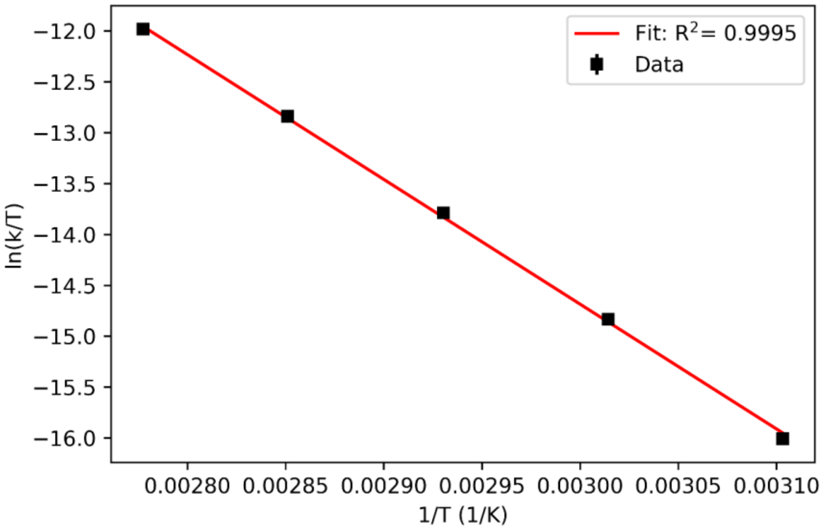

Figure S44. Eyring plot for 4-(trifluoromethyl)azobenzene.

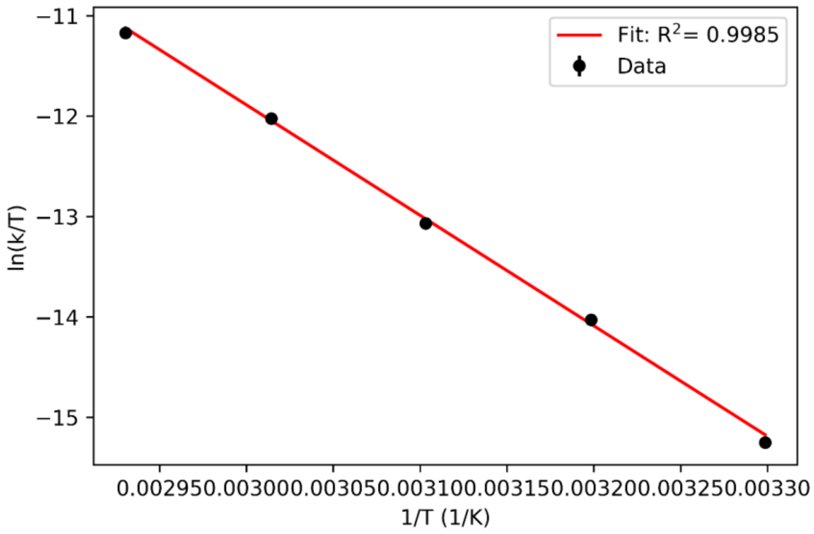

Figure S45. Eyring plot for 4-nitroazobenzene.

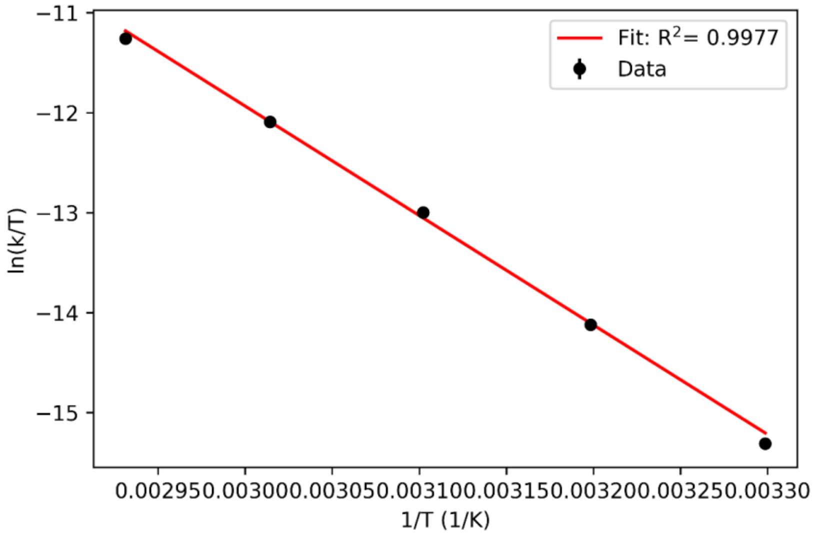

Figure S46. Eyring plot for 4-nitroazobenzene under argon.

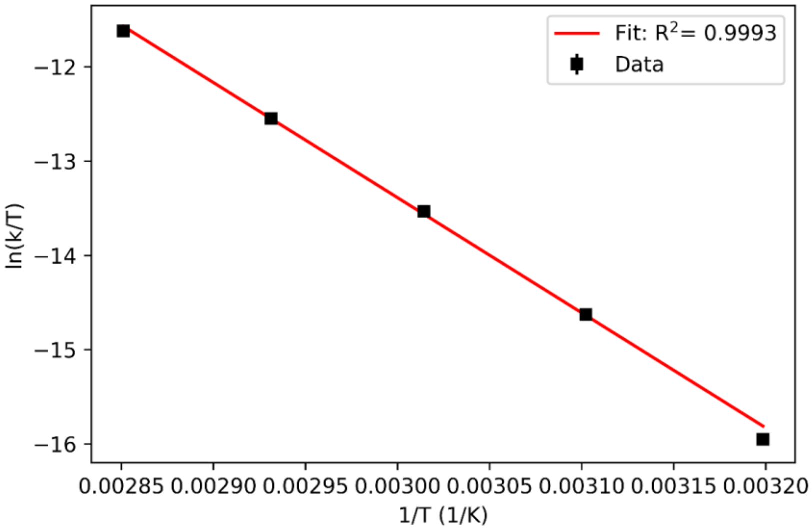

Figure S47. Eyring plot for 4-cyanoazobenzene.

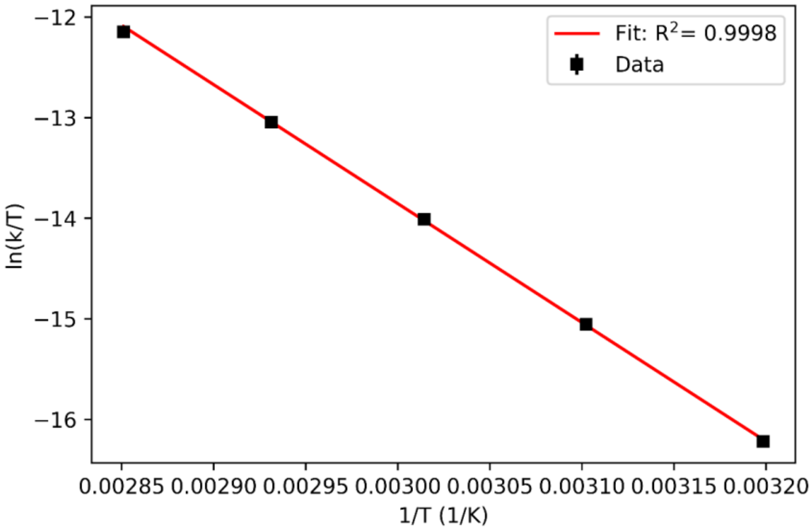

Figure S48. Eyring plot for 4-methoxyazobenzene.

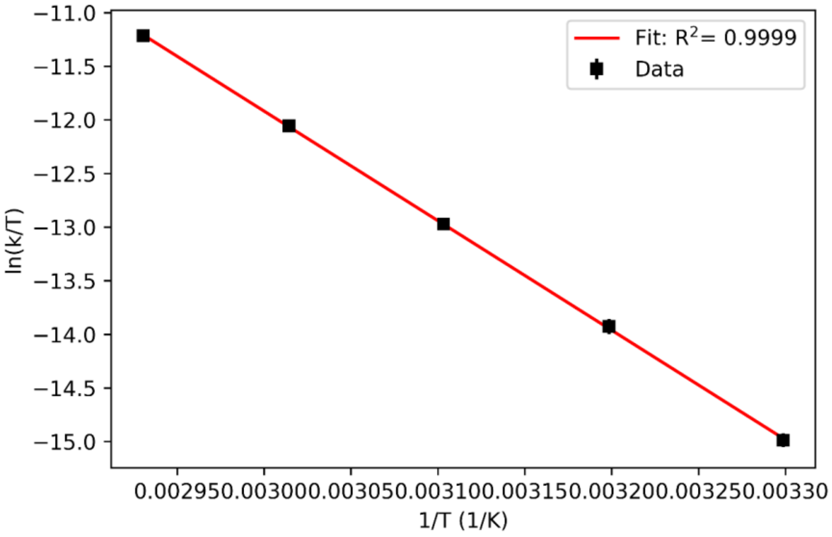

Figure S49. Eyring plot for 4-(dimethylamino)azobenzene.

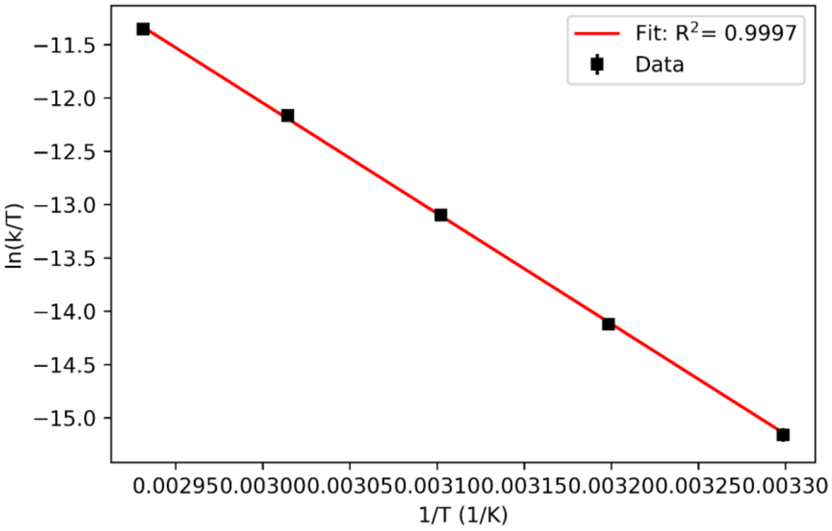

Figure S50. Eyring plot for 4-(dimethylamino)azobenzene under argon.

6 Hammett and Activation Parameters Plots

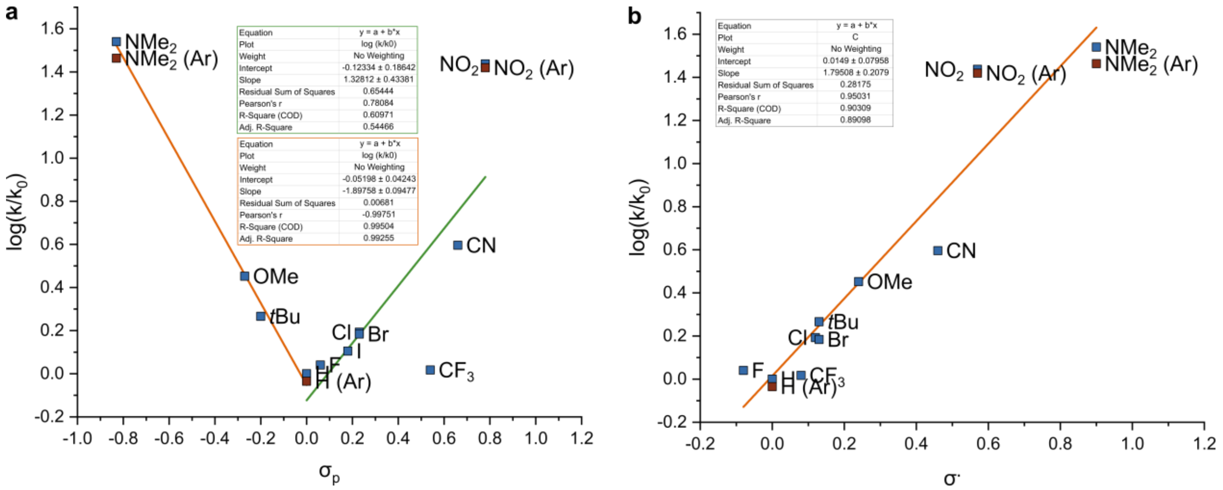

Figure S51. a. Hammett plot for the rates of mono-*para*-azobenzenes, with unsubstituted azobenzene as reference, against the tabulated σ_p Hammett-Taft parameter (CF_3 and NO_2 were not used in the linear fit).[4] The data obtained under argon are excluded from the fit. **b.** Hammett plot for the rates of mono-*para*-azobenzenes, with unsubstituted azobenzene as reference, against the tabulated σ· Creary parameter.[5] The data obtained under argon are excluded from the fit.

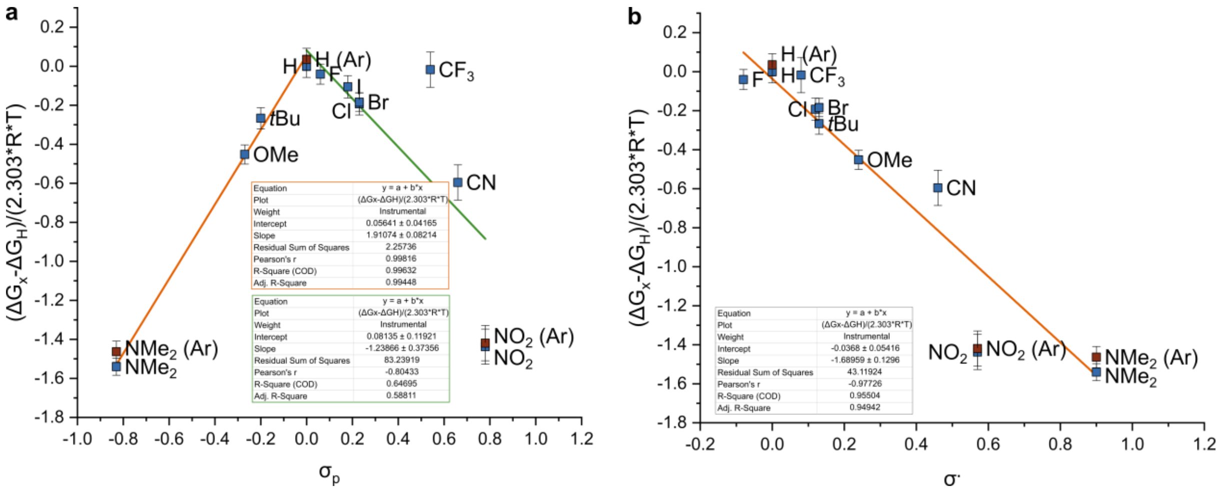

Figure S52. a. Plot of the Gibbs free energy of mono-*para*-azobenzenes, with unsubstituted azobenzene as reference, against the tabulated Hammett-Taft σ_p parameter (CF_3 and NO_2 were not used in the linear fit). The data obtained under argon are excluded from the fit. **b.** Plot of the Gibbs free energy of mono-*para*-azobenzenes, with unsubstituted azobenzene as reference, against the tabulated σ· Creary parameter. The data obtained under argon are excluded from the fit.

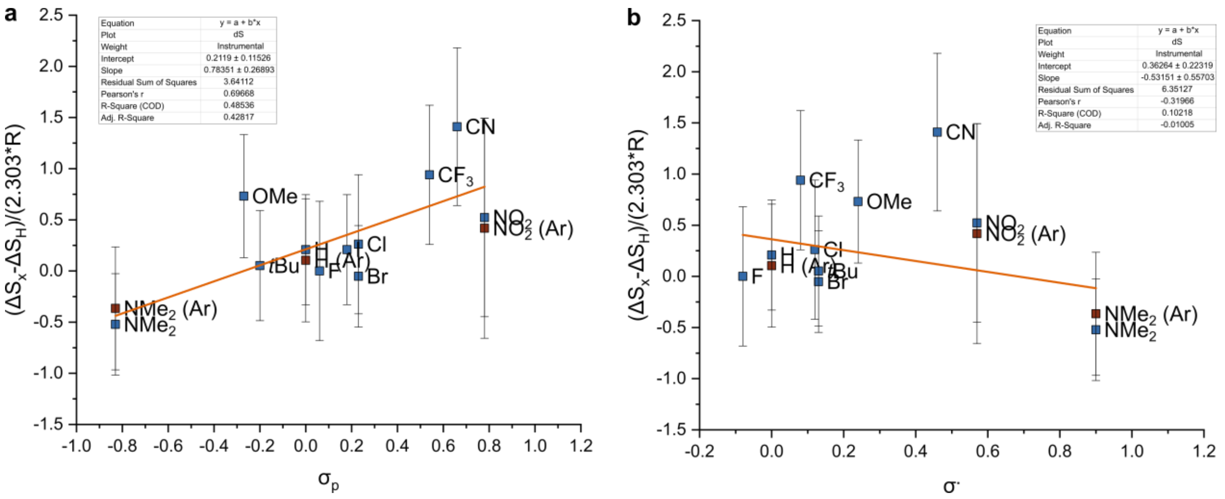

Figure S53. a. Plot of the entropy of activation of mono-*para*-azobenzenes, with unsubstituted azobenzene as reference, against the tabulated Hammett-Taft σ_p parameter. The data obtained under argon are excluded from the fit. **b.** Plot of the enthalpy of activation of mono-*para*-azobenzenes, with unsubstituted azobenzene as reference, against the tabulated σ· Creary parameter. The data obtained under argon are excluded from the fit. The linear fits shown have limited quantitative significance due to the intrinsic covariance between activation enthalpies and entropies obtained from Eyring analysis. They are included solely to illustrate qualitative trends. Notably, $\Delta H^{\ddagger}$ and $\Delta S^{\ddagger}$ display similar substituent-dependent variations, consistent with strong enthalpy–entropy compensation across the series (see following figure).

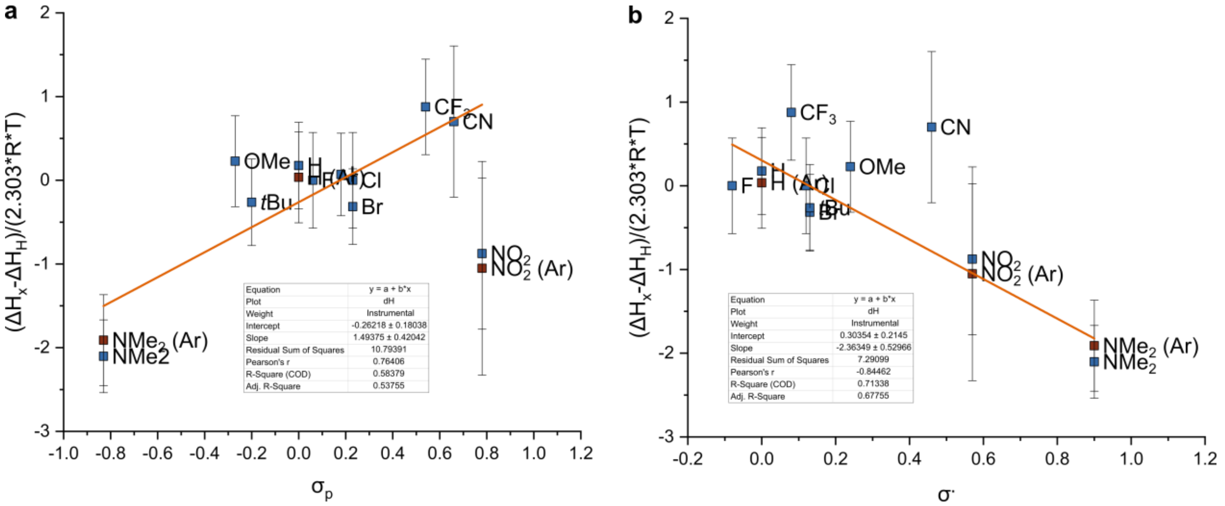

Figure S54. a. Plot of the enthalpy of activation of mono-*para*-azobenzenes, with unsubstituted azobenzene as reference, against the tabulated Hammett-Taft parameter σ_p. The data obtained under argon are excluded from the fit. **b.** Plot of the entropy of activation of mono-*para*-azobenzenes, with unsubstituted azobenzene as reference, against the tabulated σ· Creary parameter. The data obtained under argon are excluded from the fit. The linear fits shown have limited quantitative significance due to the intrinsic covariance between activation enthalpies and entropies obtained from Eyring analysis. They are included solely to illustrate qualitative trends. Notably, $\Delta H^{\ddagger}$ and $\Delta S^{\ddagger}$ display similar substituent-dependent variations, consistent with strong enthalpy–entropy compensation across the series (see previous figure).

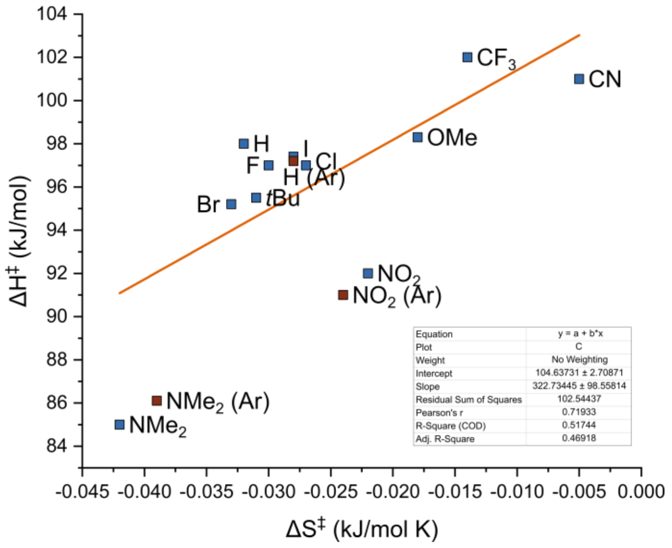

Figure S55. Plot of the change in entropy of activation against the change in enthalpy of activation across the series of mono-*para*-substituted azobenzenes. The data obtained under argon are excluded from the fit. For non-adiabatic multistate processes, the activation parameters obtained from Eyring analysis are effective (apparent) quantities. The transmission coefficient (γ < 1) enters the rate expression as a prefactor and is absorbed into the apparent activation entropy when a simple adiabatic Eyring form is used. As a result, $\Delta S^{\ddagger}$ is expected to show additional substituent-dependent scatter, even when the underlying mechanism and the effective free energies of activation are uniform. Consequently, the absence of a strong linear correlation between $\Delta H^{\ddagger}$ and $\Delta S^{\ddagger}$ or with substituent constants (the two previous figures) is not mechanistically meaningful and does not contradict a single reaction pathway.

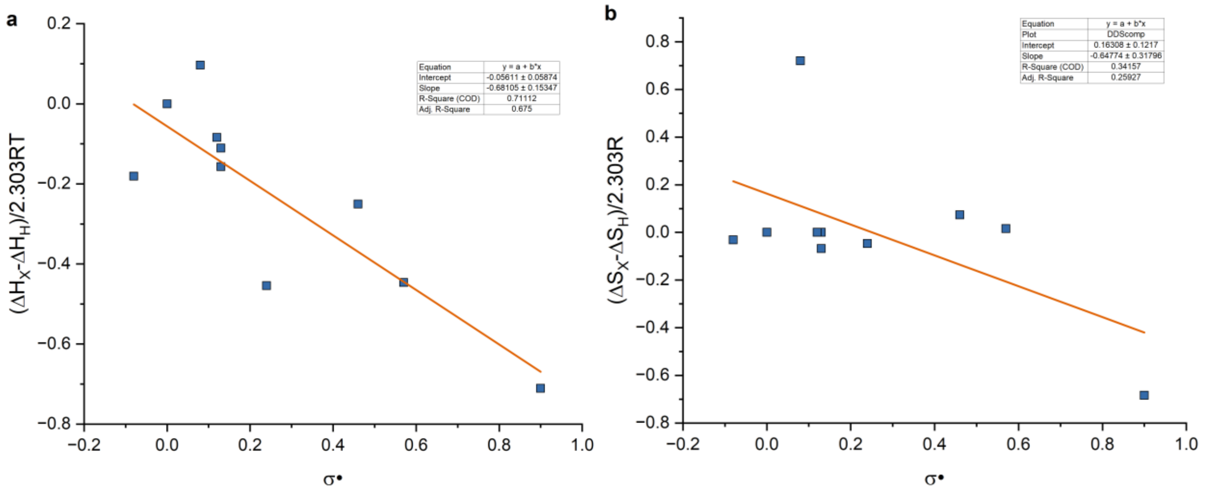

Figure S56. Correlation of computed relative activation enthalpies ($\Delta\Delta H^{\ddagger}$, **a**) and activation entropies ($\Delta\Delta S^{\ddagger}$, **b**) at the XMS-CASPT2 level with the Creary $\sigma\cdot$ substituent parameter. $\Delta\Delta H^{\ddagger}$ displays a systematic dependence on $\sigma\cdot$, whereas $\Delta\Delta S^{\ddagger}$ values cluster around a common negative value, with the apparent linear fit driven mainly by a small number of outliers. This behavior reflects the weak substituent sensitivity of the apparent activation entropy in a non-adiabatic multistate process and the experimental trend obtained in the previous Figures.

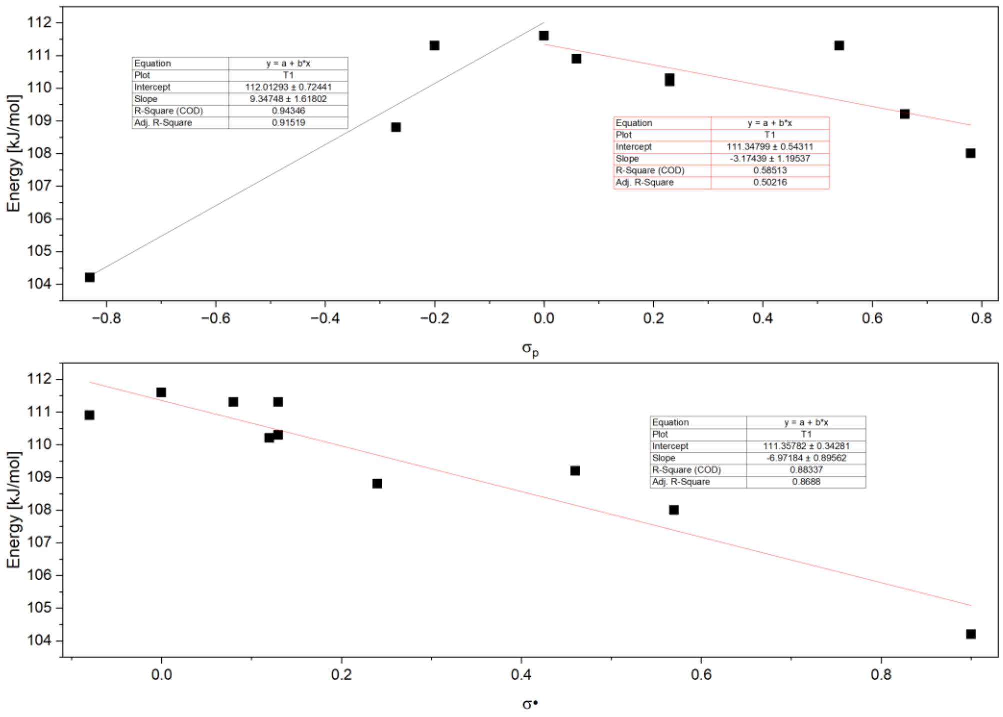

Figure S57. Top: Plot of the triplet energies (T_1, BH&HLYP/def2-TZVP, calculated using the *Z*-form as reference) of mono-*para*-azobenzenes, against the tabulated Hammett-Taft σ_p parameter. **b.** Plot for the triplet energies (T_1, BH&HLYP/def2-TZVP, calculated using the *Z*-form as reference) of mono-*para*-azobenzenes, against the tabulated σ· Creary parameter.

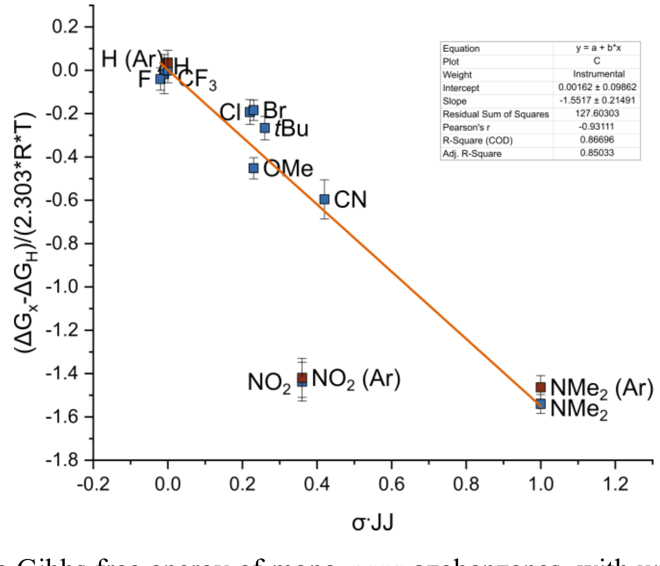

Figure S58. Plot of the Gibbs free energy of mono-*para*-azobenzenes, with unsubstituted azobenzene as reference, against the tabulated σ· JJ parameter.[6] The data obtained under argon are excluded from the fit.

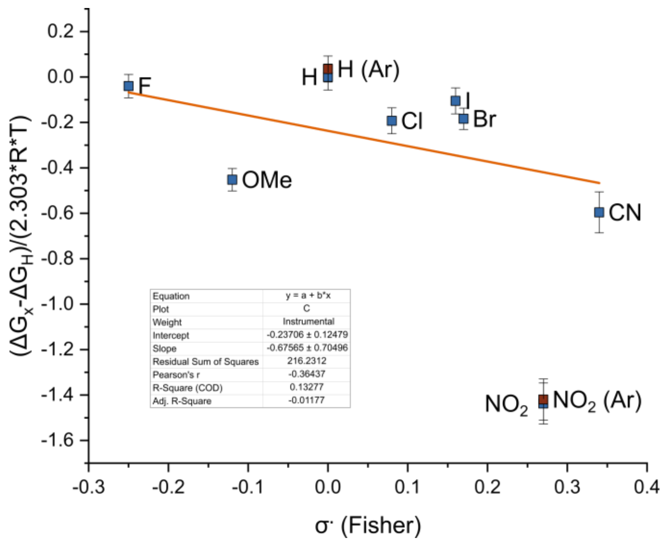

Figure S59. Plot of the Gibbs free energy of mono-*para*-azobenzenes, with unsubstituted azobenzene as reference, against the tabulated σ· (Fisher).[7,8] The data obtained under argon are excluded from the fit.

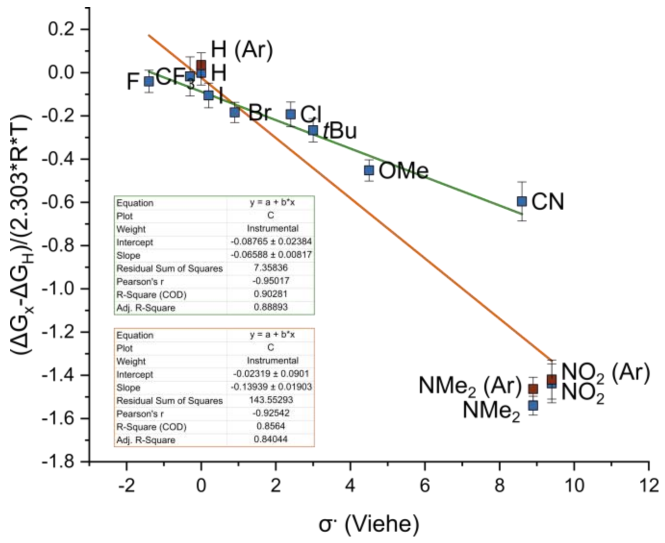

Figure S60. Plot for the Gibbs free energy of mono-*para*-azobenzenes, with unsubstituted azobenzene as reference, against the tabulated σ· (Viehe) parameter.[9] The data obtained under argon are excluded from the fit.

7 Exner Plots

Exner, or isokinetic, plots were obtained by plotting the logarithm of the kinetic constants obtained at a given temperature against those obtained at a different temperature. Suppose the temperature difference is significant enough (> 10K), and given that the plot is linear, it is possible to infer that the series follows the same type of entropy-enthalpy compensation along the entire series. Indirectly, it advocates for the presence of a single mechanism that is followed by all compounds in the series. This isokinetic relationship relates two experimentally determined rates (k_1, k_2) measured at two distinct temperatures ($T_2 > T_1$) according to equation 7.1.[10]

$$\log k_2 = a + b \log k_1 \tag{7.1}$$

Where the slope b is related to the isokinetic temperature, β, according to equation 7.2.

$$\beta = T_1 T_2 \frac{1 - b}{T_1 - bT_2} \tag{7.2}$$

Using 298 K and 333 K for T_1 and T_2 and 1.13 for b (see Figure S64), we were able to determine the isokinetic temperature to be 165 K. In every Exner plot that we constructed, we observed b > 1, meaning that there is compensation for enthalpy-entropy along the substituent series.

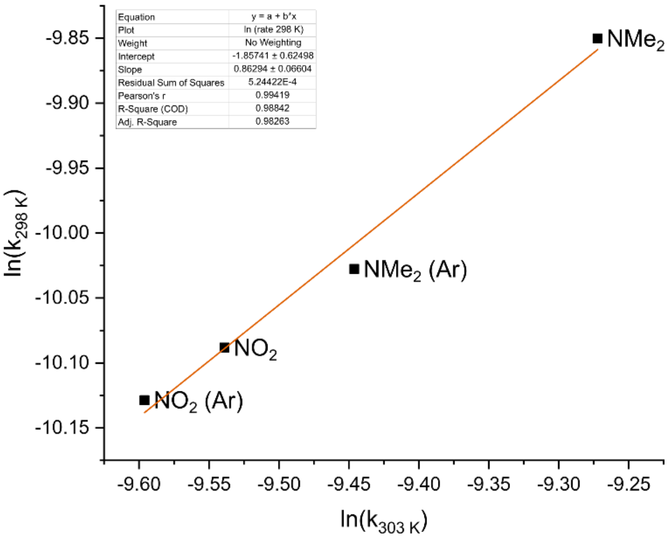

Figure S61. Exner Plot of the thermal relaxation rate of mono-*para*-azobenzenes at 298 K against the rate at 303 K.

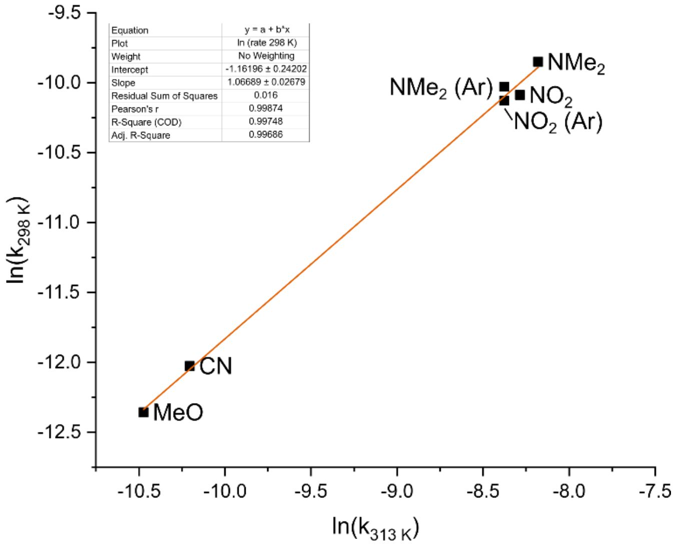

Figure S62. Exner Plot of the thermal relaxation rate of mono-*para*-azobenzenes at 298 K against the rate at 313 K.

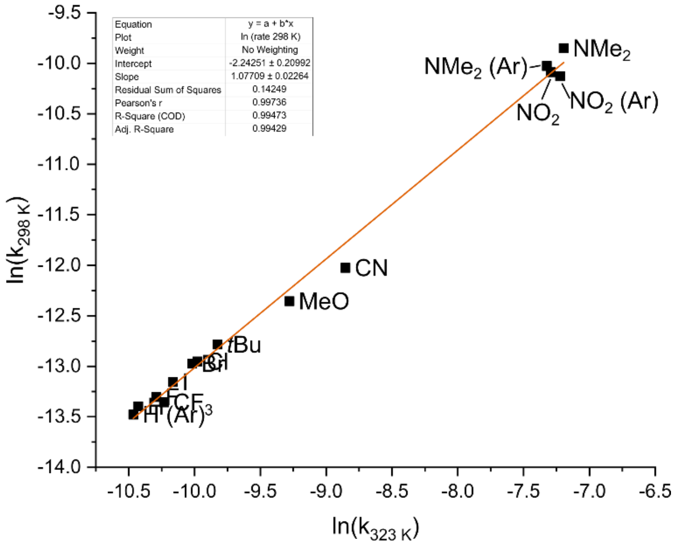

Figure S63. Exner Plot of the thermal relaxation rate of mono-*para*-azobenzenes at 298 K against the rate at 323 K.

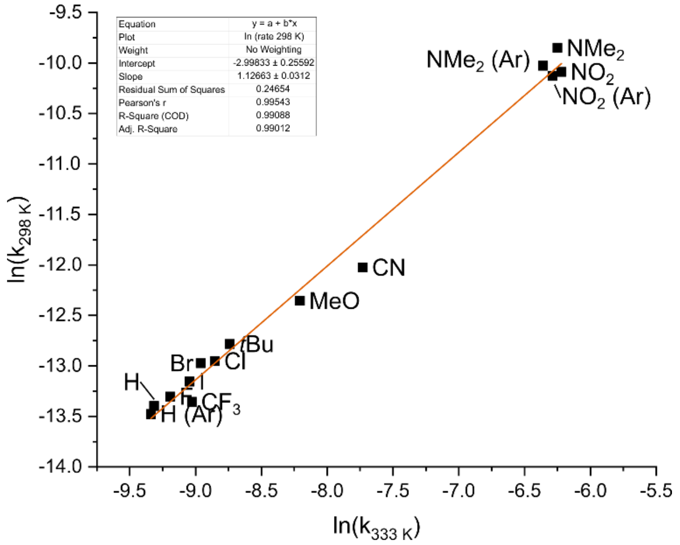

Figure S64. Exner Plot of the thermal relaxation rate of mono-*para*-azobenzenes at 298 K against the rate at 333 K.

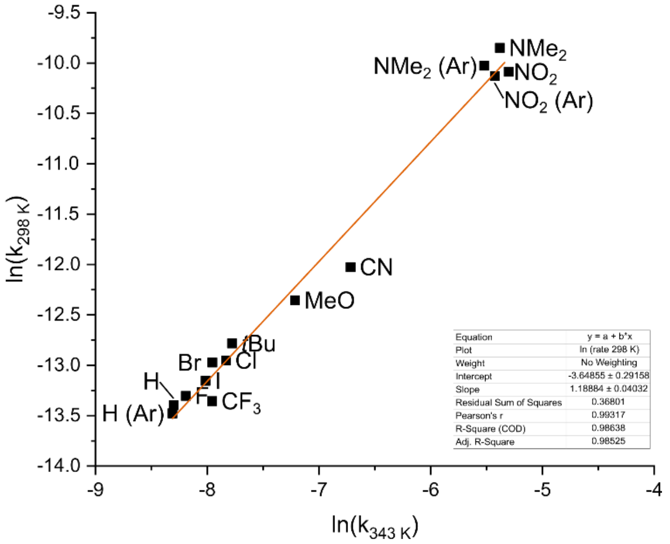

Figure S65. Exner Plot of the thermal relaxation rate of mono-*para*-azobenzenes at 298 K against the rate at 343 K.

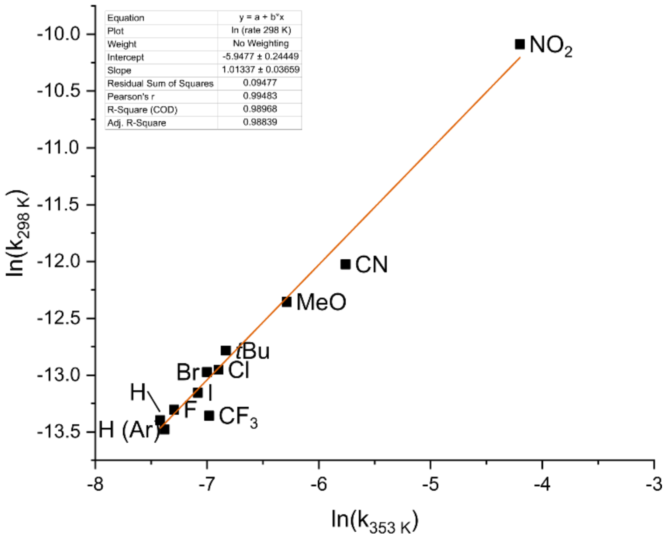

Figure S66. Exner Plot of the thermal relaxation rate of mono-*para*-azobenzenes at 298 K against the rate at 353 K.

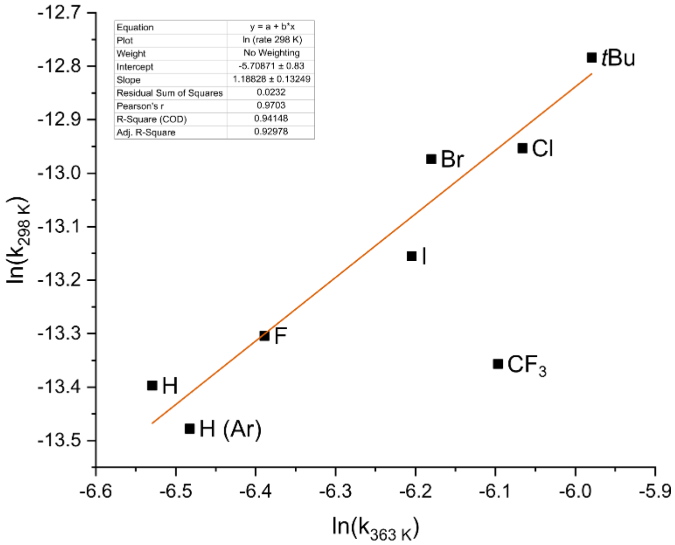

Figure S67. Exner Plot of the thermal relaxation rate of mono-*para*-azobenzenes at 298 K against the rate at 363 K.

8 Computational Analysis

8.1 General Details for Optimizations

All geometry optimizations at the DFT level were performed using ORCA 6.0.1[11,12] program package with the following combinations of density functionals and basis sets: PBE0-D4[13]/def2-TZVP[14,15], B97-3c[16], ωB97X-3c[17] or r^2SCAN-3c.[18] Geometry optimizations at the complete active space self-consistent field (CASSCF) level, specifically CASSCF(14,12)/ANO-S-VDZP averaging the three lower singlet states,[19–21] were performed in OpenMOLCAS 23.10.[21] ωB97X-3c was selected to be the most cost-efficient method to provide geometries for further energy corrections based on a benchmark described in Section 8.2. Further details on CASSCF are provided in Section 8.3.2. Depending on the structure type, different approaches were applied during optimization; please refer to the corresponding subsections below.

With the optimized structures in hand, single-point energies were calculated with mixed-reference spin flip[22] (MRSF) TDDFT (as implemented in OpenQP[23] program package) at BH&HLYP[24–27]/def2-TZVP or ωB97X-D4[28–30]/def2-TZVP levels (in the gas phase) as well as with spin flip[31] (SF) DFT at BH&HLYP-D4/def2-TZVP level in the gas phase and with toluene as implicit solvent (using the SMD[32] implicit solvation method). Additional single points where computed at the DLPNO-(U)CCSD(T)/aug-cc-pVTZ as implemented in ORCA 6.0.1[11] and (XMS-)CASPT2(14,12)/ANO-RCC-VTZP (for the majority of substituents) and (XMS-)CASPT2(16,14)/ANO-RCC-VTZP (for NO_2 and CN; see the corresponding section on the CASSCF//CASPT2 calculation details).

All optimized geometries are available at the following DOI: 10.6084/m9.figshare.30444422.

All energies are provided in a separate Excel spreadsheet in the Electronic Supplementary Information.

8.1.1 Ground State Minima Optimization

Ground state minima (*Z*-isomers) were calculated at the corresponding level of theory with a regular restricted closed-shell Kohn-Sham reference function, followed by the frequency calculation (no imaginary vibration modes found).

8.1.2 Triplet State Minima Optimization

T_1 state minima were calculated at the ωB97X-3c and MRSF-BH&HLYP/def2-TZVP levels of theory with an unrestricted Kohn-Sham reference function. MRSF-TDDFT was employed following the default settings implemented in OpenQP. The optimization for MRSF-TDDFT used ORCA as the external optimizer for OpenQP, using an in-house script.[33]

8.1.3 Transition State for the Inversion Mechanism

Transition states for inversion (TSInv) were calculated at the corresponding level of theory with an unrestricted Kohn-Sham reference function. Starting geometries were obtained at xTB/GFN2 level of theory (with Fermi smearing applied, T = 1500 K) with the help of Pysisyphus program package;[34] initial Hessian matrices were calculated with ORCA 6.0.1 at xTB/GFN2 level of theory (with Fermi smearing, T = 1500 K). The TS nature of the obtained structures was confirmed with frequency analysis (a single imaginary frequency observed). For all substituted azobenzenes, a separate inversion transition state was identified for each nitrogen atom of the double bond (Inv1 and Inv2). In the final rate constant calculation, the two inversion pathways were considered separately. Then the rate constants for the two inversions were summed up to yield the final inversion rate constant (ΣInv).

8.1.4 Transition State for the Adiabatic Rotation Mechanism

Transition states for *adiabatic* rotation (TSRot) were calculated at the corresponding level of theory with unrestricted Kohn-Sham reference function within Broken Symmetry (BS) formalism (%scf BrokenSym 1,1) to take into account the breaking of the π-bond appropriately. Geometry optimization was followed by the frequency calculation (a single imaginary vibrational mode was found). Starting geometries xTB/GFN2 level of theory (with Fermi smearing applied, T = 1500 K) with the help of pysisyphus program package;[34] initial Hessian matrices were calculated with ORCA 6.0.1 at xTB/GFN2 level of theory (with Fermi smearing, T = 1500 K). The TS nature of the obtained structures was confirmed with frequency analysis (a single imaginary frequency observed).

8.1.5 Non-adiabatic Rotation Mechanism

The multireference character of the S_0 potential energy surface (PES) in the region close to the Minimum Energy Crossing Points between S_0 and T_1 (MECP, with MECP1 the crossing point closer to the *Z*-isomer of the corresponding azobenzene, and MECP2 the one on the opposite side of the transition state of rotation), arises from the proximity of the S_1 state PES. Due to the multi-configurational nature of the wavefunction in that region, single-reference methods like conventional DFT usually fail even to find an appropriate MECP geometry if run with default settings, predicting C-N=N-C dihedral for azobenzene MECP1 (closer to the *Z*-isomer on the reaction coordinate) to be ~61° (instead of ~71°, reported in different references [35,36]).

To accurately recover the geometry of the MECPs, we optimized these geometries with ORCA 6.0.1 at a corresponding DFT level of theory within Broken Symmetry formalism (%scf BrokenSym 1,1) for the S_0 state and with an unrestricted Kohn-Sham open shell reference function for the T_1 state. Alternatively, optimization was performed using OpenMOLCAS 23.10 at the CASSCF(14,12) level for H, NO_2, and NMe_2 substituted azobenzenes.

8.1.6 On the Significance of the Conformational Space in the Study

For *tert*-butyl, trifluoromethyl, and methoxy-substituted azobenzenes, two conformers can be found for the *Z*-isomer as well as for most transition states (some conformers for inversion transition states become degenerate or nearly degenerate due to the changes in the symmetry along the reaction coordinate; in such cases, a single geometry for such a TS was combined with two conformers of the ground state). In the current study, we performed all analyses separately for such pairs of conformers. The difference in the final values of the effective Gibbs energy of activation rarely exceeded 1 kJ/mol, which is within the accuracy of the computational methods employed; thus, the conformer to consider was selected arbitrarily.

8.2 On the Choice of DFT Method for Optimizations

For the optimization method used, we tested a variety of functionals (PBE0/def2-TZVP, r^{2}SCAN-3c, B97-3c, and ωB97X-3c) using toluene as implicit solvent (SMD), with three different substituents (H, NMe_2 and NO_2, to cover the extremes of the electron effects) at the (BS-)DFT level as described above. We compared the complete set of structures (*Z*-, MECPs, all TSs) *vs.* geometries optimized at the CASSCF(14,12)/ANO-S-VDZP level to assess the feasibility of using DFT structures for the substituent series; geometrical RMSD was used as the quality metric. The largest displacements were due to the different pyramidalization of the amino group in NMe_2-azobenzene.

The best results were obtained with the ωB97X-3c functional, with an RMSD of 0.1836 Å.

We therefore decided to use ωB97X-3c as the functional to provide both geometrical and thermal energy corrections. We should mention that all DFT geometries studied are comparable in quality.

The complete RMSD data are provided in the following Tables.

Table S1. RMSD of the azobenzenes geometries optimized via ωB97X-3c vs CASSCF(14,12)/ANO-S-VDZP. The average RMSD is 0.18363.

Substituent	Z	MECP1	MECP2	TSInv	TSInv2	TSRot
H	0.042799	0.041093	0.070417	0.326182	[a]	0.041699
NMe2	0.489310	0.180372	0.110981	0.500241	0.357382	0.149992
NO2	0.059050	0.053284	0.091794	0.136241	0.430713	0.040094

[a] TSInv and TSInv2 are degenerate in azobenzene.

Table S2. RMSD of the azobenzenes geometries optimized via r^2SCAN-3c vs CASSCF(14,12)/ANO-S-VDZP. The average RMSD is 0.19861.

Substituent	Z	MECP1	MECP2	TSInv	TSInv2	TSRot
H	0.18097	0.06714	0.11105	0.29088	[a]	0.06858
NMe2	0.51167	0.13691	0.10381	0.15825	0.37957	0.11853
NO2	0.23347	0.09051	0.14391	0.14799	0.54554	0.08764

[a] TSInv and TSInv2 are degenerate in azobenzene.

Table S3. RMSD of the azobenzenes geometries optimized via b97-3c vs CASSCF(14,12)/ANO-S-VDZP. The average RMSD is 0.22873.

Substituent	Z	MECP1	MECP2	TSInv	TSInv2	TSRot
H	0.18899	0.09515	0.12732	0.31420	[a]	0.09347
NMe2	0.52005	0.16365	0.12515	0.29661	0.37181	0.15386
NO2	0.24764	0.13022	0.15178	0.13478	0.63860	0.13510

[a] TSInv and TSInv2 are degenerate in azobenzene.

Table S4. RMSD of the azobenzenes geometries optimized via PBE0-D4/def2-TZVP vs CASSCF(14,12)/ANO-S-VDZP. The average RMSD is 0.19076.

Substituent	Z	MECP1	MECP2	TSInv	TSInv2	TSRot
H	0.13148	0.06149	0.10314	0.32310	[a]	0.04751
NMe2	0.50745	0.17026	0.11693	0.30995	0.36924	0.14769
NO2	0.17643	0.08532	0.13396	0.12888	0.36499	0.06505

[a] TSInv and TSInv2 are degenerate in azobenzene.

8.3 Specific Details on the Methods Employed

8.3.1 Coupled Cluster Calculations

Coupled cluster calculations were performed in ORCA 6.0.1 within domain-based local pair natural orbital coupled cluster singles, doubles and perturbative triples (DLPNO-CCSD(T))[37–45] approach with the aug-cc-pVTZ basis set (with auxiliary aug-cc-pVTZ/C and aug-cc-pVTZ/C/JK bases) and with the full iterative (DLPNO-MP2) treatment of the MP2 guess, triggered by “TightPNO” and “%MDCI UseFullLMP2Guess” keywords. For all structures, an unrestricted Kohn-Sham reference function was used ((U)CCSD(T)); wavefunction stability

was performed in all calculations before performing the coupled cluster run. (BS)-DFT ωB97X-3c optimized structures were used for the DLPNO-(U)CCSD(T)//aug-cc-pVTZ correction (see Section 8.2).

8.3.2 CASSCF/CASPT2 Calculations

All complete active space self-consistent field (CASSCF) and second-order multiconfigurational perturbation theory (CASPT2) calculations were performed in OpenMOLCAS 23.10,[21] averaging the lowest three states of the same multiplicity (SA3). (BS)-DFT ωB97X-3c optimized structures were used for the CASPT2//CASSCF correction (see Section 8.2). To choose the active space, we followed the recommendations from Cembran *et al***.**[46], suggesting (14,12) active space with five π, five π* orbitals, and two N lone pairs. For NO_2 and CN, we also included one π and one π* orbital of the substituent. For substituents containing extra lone pairs (halogens, OMe, NMe_2), we attempted to include one lone pair of the substituent, but could not locate a stable active space with such a set of orbitals.

To obtain the desired active space, we first used a small ANO-S-VDZP basis set. Subsequently, we expanded it with the EXPBAS tool in OpenMOLCAS to a large relativistic ANO-RCC-VTZP basis set. An exception is *p*-I-substituted azobenzene, for which we used ANO-RCC-VTZP from the outset due to the nature of the substituent.

After the stable active spaces were found, all-states extended multi-state second-order multiconfigurational perturbation theory (XMS-CASPT2) was applied to calculate the energies of the first three singlet and triplet excited states. No IPEA shift was used. Different imaginary shift values (0.0, 0.1, 0.2, 0.3) were tested for both single-state CASPT2 and XMS-CASPT2 for *p*-NO2-azobenzene (nitroazobenzene was chosen as the test case because it is among the most problematic molecules for both DFT and CCSD(T) methods in terms of the competition between NA-Rot and Inv mechanisms, see Section Table S16). Setting an imaginary shift of 0.1 was sufficient to avoid the intruder-state problem (see Table S5 and Table S6).

Table S5. Reference weights for each state with different imaginary shifts at the XMS-CASPT2(16,14)/ANO-RCC-VTZP level for nitrozobenzene at different geometries obtained at the ωB97X-3c/SMD(toluene) level of theory.

	**MEP1**				**MEP2**			
IMAG	0	0.1	0.2	0.3	0	0.1	0.2	0.3
S_0	0.5497	0.5513	0.5551	0.5607	0.5479	0.5510	0.5551	0.5609
S_1	0.5319	0.5481	0.5523	0.5583	0.5464	0.5494	0.5534	0.5592
S_2	0.4627	0.5461	0.5509	0.5573	0.5278	0.5470	0.5517	0.5580
T_1	0.4800	0.5488	0.5532	0.5591	0.4064	0.5494	0.5537	0.5597
T_2	0.4605	0.5397	0.5468	0.5544	0.2054	0.5426	0.5486	0.5558
T_3	0.3200	0.5375	0.5457	0.5538	0.2836	0.5379	0.5460	0.5542

	**TSInv**				**TSInv2**			
IMAG	0	0.1	0.2	0.3	0	0.1	0.2	0.3
S_0	0.5494	0.5509	0.5548	0.5605	0.5496	0.5518	0.5556	0.5613
S_1	0.5370	0.5485	0.5528	0.5589	0.5502	0.5516	0.5553	0.5609
S_2	0.5213	0.5426	0.5492	0.5565	0.5420	0.5471	0.5513	0.5574
T_1	0.5366	0.5453	0.5498	0.5561	0.5034	0.5440	0.5493	0.5559
T_2	0.5440	0.5527	0.5575	0.5635	0.5319	0.5485	0.5546	0.5611
T_3	0.0254	0.5496	0.5542	0.5605	0.4082	0.5377	0.5466	0.5548

	**TSRot**				**Z**			
IMAG	0	0.1	0.2	0.3	0	0.1	0.2	0.3
S_0	0.5484	0.5503	0.5543	0.5601	0.5546	0.5556	0.5588	0.5638
S_1	0.5443	0.5475	0.5517	0.5578	0.4605	0.5478	0.5524	0.5584
S_2	0.4792	0.5469	0.5516	0.5578	0.0217	0.5359	0.5448	0.5532
T_1	0.5398	0.5490	0.5536	0.5595	[a]	0.5400	0.5472	0.5549
T_2	0.2598	0.5392	0.5468	0.5547	[a]	0.5488	0.5549	0.5614
T_3	0.1168	0.5379	0.5462	0.5542	[a]	0.5388	0.5480	0.5557

[a] The calculation suffered from PT2 convergence issues.

Table S6. Reference weights for each state with different imaginary shifts at the CASPT2(16,14)/ANO-RCC-VTZP level for nitrozobenzene at different geometries obtained at the ωB97X-3c/SMD(toluene) level of theory.

	**MEP1**				**MEP2**			
IMAG	0	0.1	0.2	0.3	0	0.1	0.2	0.3
S_0	0.5532	0.5545	0.5580	0.5634	0.5528	0.5546	0.5583	0.5637
S_1	0.5468	0.5490	0.5529	0.5588	0.5482	0.5502	0.5540	0.5597
S_2	0.5204	0.5481	0.5524	0.5585	0.5477	0.5499	0.5539	0.5598
T_1	0.5401	0.5505	0.5550	0.5608	0.5435	0.5510	0.5554	0.5612
T_2	0.4654	0.5396	0.5465	0.5542	0.4984	0.5440	0.5497	0.5567
T_3	0.0046	0.5380	0.5458	0.5539	0.2786	0.5383	0.5461	0.5541
	**TSInv**				**TSInv2**			
IMAG	0	0.1	0.2	0.3	0	0.1	0.2	0.3
S_0	0.5553	0.5565	0.5599	0.5652	0.5568	0.5579	0.5612	0.5662
S_1	0.5435	0.5503	0.5544	0.5603	0.5503	0.5517	0.5554	0.5610
S_2	0.5409	0.5445	0.5502	0.5573	0.5529	0.5541	0.5574	0.5627
T_1	0.5443	0.5481	0.5526	0.5586	0.5433	0.5480	0.5527	0.5589
T_2	0.5530	0.5571	0.5615	0.5671	0.5468	0.5514	0.5573	0.5639
T_3	0.5474	0.5507	0.5550	0.5612	0.5317	0.5427	0.5497	0.5573
	**TSRot**				**Z**			
IMAG	0	0.1	0.2	0.3	0	0.1	0.2	0.3
S_0	0.5499	0.5519	0.5558	0.5615	0.5581	0.5591	0.5621	0.5670
S_1	0.5471	0.5492	0.5532	0.5590	0.5113	0.5495	0.5536	0.5593
S_2	0.5421	0.5489	0.5531	0.5590	0.4333	0.5390	0.5459	0.5536
T_1	0.5430	0.5507	0.5553	0.5611	0.4881	0.5471	0.5519	0.5581
T_2	0.1252	0.5389	0.5464	0.5542	0.5461	0.5515	0.5575	0.5640
T_3	0.4448	0.5399	0.5470	0.5548	0.0022	0.5407	0.5486	0.5568

Single-state CASPT2 energies were evaluated for comparison; the difference in the results of the single- and multi-state CASPT2 is within 0.2 kJ/mol for *Z* and TSInv, up to 1.7 kJ/mol for MECPs, with the most significant deviation for TSRot, where XMS-CASPT2 shows consistently higher energies (for 5.3–14.1 kJ/mol, depending on the substituent).

Table S7. Energy difference (kJ/mol) between CASPT2 and XMS-CASPT2 results (green: higher XMS-CASPT2)

	**Z**	**MEP1**	**MEP2**	**TSInv**	**TSInv2**	**TSRot**
H	-0.09	-0.79	2.25	-0.12	-0.12	-5.43
Br	-0.09	-0.86	2.20	-0.14	-0.10	-6.03
CF_3	-0.07	-0.87	0.68	-0.19	-0.04	-6.72
CN	-0.09	-1.05	1.84	-0.25	-0.03	-9.08
Cl	-0.10	-0.91	1.90	-0.13	-0.10	-5.93
F	-0.11	-1.11	1.71	-0.08	-0.13	-5.33
I	-0.09	-0.93	2.11	-0.12	-0.09	-6.28
NMe_2	-0.38	-4.25	-0.11	0.17	-0.27	-14.11
NO_2	-0.07	-1.27	1.99	0.00	0.03	-11.10
OMe	-0.20	-2.72	0.14	-0.04	-0.18	-7.77
tBu	-0.10	-1.50	1.42	-0.09	-0.13	-6.02
**RMSD**	0.15	1.76	1.55	0.14	0.13	7.91

Figures of the active spaces are provided in Section 8.8.

8.3.3 SF-TDDFT and MRSF-TDDFT

Single-point energy refinements along the thermal corrections were carried out with spin-flip DFT (SF-DFT) and mixed-reference spin-flip TDDFT (MRSF-TDDFT) to obtain a balanced description in the potential energy surface (PES) region where the electronic structure departs from a single closed-shell determinant (notably near the MECP and the rotational TS). SF-based formalisms are specifically designed to treat such near-degeneracy and open-shell singlet character, and have been adopted and benchmarked for azobenzene thermal isomerization/ISC models in the context of the Bombarelli group's high-throughput/ML workflow, typically using BH&HLYP to mitigate spin-contamination artifacts.[47] Accordingly, we employed BH&HLYP-D4/def2-TZVP for SF-TDDFT single points and explicitly monitored $\langle S^2 \rangle$ to ensure consistent state assignment, using the $\langle S^2 \rangle$ diagnostics to select the appropriate singlet energies in strongly multireference regions (MECPs and TSRot, with the $\langle S^2 \rangle$ value always below 1.2). By using the ORCA implementation, it was also possible to correct the single point with solvent (toluene) using the implicit SMD method.

MRSF-TDDFT was used as a complementary, modern SF framework that enhances the robustness of SF-TDDFT in challenging situations (e.g., reduced sensitivity to spin contamination) and has been benchmarked across organic photochemical landscapes and thermal barriers of azo compounds.[48]

We therefore computed MRSF-TDDFT single points with BH&HLYP and ωB97X-D4 (both with def2-TZVP as basis set). We validated the electronic character by orbital/state tracking for unsubstituted azobenzene, confirming that across the isomerization, the original S_0 remains the ground state and S_1 consistently corresponds to the expected $n \rightarrow \pi^*$ excitation.

8.3.4 Comparison of Electronic Energies Obtained with Different Methods

We compared the electronic activation energies of the different paths for the thermal *Z→E* isomerization of azobenzene (TSInv, TSRot, and the two MECPs) obtained with varying levels of theory with two literature reference datasets (Kaupp *et al.*[35] and Wu *et al.*[36]). Except for broken-symmetry DFT, all methods place the minimum-energy crossing points (MECP1 and MECP2) below both inversion and rotational transition states. Multireference perturbative approaches (XMS-CASPT2 and CASPT2) yield MECP energies (MECP1 ca. 87–88 kJ/mol, MECP2 *ca.* 79–81 kJ/mol) close to the reference from Wu (as one would expect considering a single-state CASPT2 was used in the reference) and well below the corresponding TSs, consistent with stabilization of near-degenerate open-shell singlet character along rotation. Spin-flip DFT at the BH&HLYP/def2-TZVP level reproduces the inversion barrier in excellent agreement with both references and yields MECP energies closely matching the theoretical best estimate values from Kaupp *et al.*. Solvation corrections (SMD) have only a minor quantitative effect and do not alter the mechanistic ranking. Mixed-reference spin-flip TDDFT further confirms the preference for the non-adiabatic rotational pathway, but exhibits a marked functional dependence: BH&HLYP systematically lowers all barriers, whereas ωB97X-D4 yields MECP2 energies in near-quantitative agreement with Wu *et al.*, but substantially overestimates the rotational TS. In contrast, DLPNO-(U)CCSD(T) strongly overestimates the rotational barrier but agrees with the MECPs, reflecting the known limitations of single-reference methods in regions of pronounced multireference character. While this result does not affect the mechanistic interpretation for unsubstituted azobenzene, where adiabatic rotation is not kinetically competitive, it suggests that caution is warranted when applying single-reference coupled-cluster approaches to substituted systems in which rotational pathways may

be energetically lowered. Finally, broken-symmetry DFT (ωB97X-3c) has the opposite effect: it significantly stabilizes the rotational and MECP regions, thereby artificially lowering TSRot and enabling partial competition from the adiabatic rotational pathway, as expected from symmetry-breaking stabilization of diradicaloid configurations. These results demonstrate that any method that adequately describes the open-shell singlet and near-degenerate electronic structures provides a consistent mechanistic ranking of the relevant stationary points in the thermal isomerization of azobenzene. While absolute barriers remain method-dependent, the relative ordering of adiabatic transition states and MECPs is preserved across multireference and spin-flip approaches, supporting their general applicability for modeling the thermal back-reaction and computing the thermal activation energies.

Table S8. Comparison of the electronic energy barriers between Z-azobenzene and different relevant points on the thermal isomerization coordinate (in kJ/mol) at various levels of theory. The reference values reported in Kaupp *et al.*[35] and Wu *et al.*[36] are also shown.

	Kaupp*et al.*	Wu *et al.*	ωB97X-3c/SMD	XMS-CASPT2[a]	CASPT2[a]	DLPNO-(U)CCSD(T)[b]	SF-BH&HLYP-D4[c]	SF-BH&HLYP-D4/SMD[c]	MRSF-BH&HLYP[c]	MRSF-ωB97X-D4[c]
TSInv	130	128	121	116	116	132	131	134	113	119
TSRot	127	117	88	111	106	158	134	132	121	150
MECP1	106	92	83	88	87	106	106	105	87	99
MECP2	96	88	73	79	81	97	94	92	73	88

[a] Active space (14,12), basis set ANO-RCC-VTZP

[b] Basis set aug-cc-pVTZ

[c] Basis set def2-TZVP

8.4 Wentzel-Kramers-Brillouin (WKB) theory

The non-adiabatic rotation mechanism (NA-Rot) requires consideration within Wentzel-Kramers-Brillouin (WKB) theory, which was previously applied in the case of azobenzenes, for instance, by Reimann *et al.*,[35] Axelrod *et al.*,[47] Singer and Schlögl *et al.*[49] Analogously, we applied the WKB theory in the current study, following the expressions:

$$k_{NARot} = \left(\frac{1}{k_{ISC1}} + \frac{1}{k_{ISC2}}\right)^{-1} = \frac{k_{ISC1} \cdot k_{ISC2}}{k_{ISC1} + k_{ISC2}} \tag{8.1}$$

$$k_{ISC}(T, V_{SO}) = \frac{k_B T}{h} \gamma(T, V_{SO}) e^{\left(-\frac{\Delta G_{MECP}}{RT}\right)} = \frac{\pi^{\frac{3}{2}} \beta}{2h\sqrt{\varepsilon_0 / k_B T}} \left[1 + \frac{1}{2} e^{\left(\frac{1}{12\beta^2 (k_B T \varepsilon_0)^3}\right)}\right] e^{\left(-\frac{\Delta G_{MECP}}{RT}\right)} \tag{8.2}$$

$$\beta = \frac{4 V_{SO}^{\frac{3}{2}}}{\hbar} \left(\frac{\mu}{\bar{F} \Delta F}\right)^{\frac{1}{2}} \tag{8.3}$$

$$\varepsilon_0 = \frac{\Delta F}{2\bar{F}V_{SO}} \tag{8.4}$$

$$\Delta F = \left|\mathbf{F}_{T_1} - \mathbf{F}_{S_0}\right| \tag{8.5}$$

$$\bar{F} = \left|\sum_{j=1}^{N}\sum_{n=1}^{3}\left(\mathbf{F}_{S_0}\right)_{jn}\left(\mathbf{F}_{T_1}\right)_{jn}\right|^{\frac{1}{2}} \tag{8.6}$$

$$\mu = \left(\frac{1}{|\Delta F|^2}\sum_{j=1}^{N}\sum_{n=1}^{3}\Delta F_{jn}^2 m_n^{-1}\right)^{-1} \tag{8.7}$$

With the optimized MECP geometry in hand, we calculated the gradients $\mathbf{F}_{S_0}$ and $\mathbf{F}_{T_1}$. Electronic energies at MECP geometries were calculated at SF-DFT BH&HLYP-D4/def2-TZVP level in gas phase and with toluene within the SMD model, as well as at MRSF-DFT BH&HLYP/def2-TZVP and MRSF-DFT ωB97X-D4/def2-TZVP level (both in gas phase due to the absence of a solvent model in relevant version of OpenQP) DLPNO-(U)CCSD(T) and CASPT2. The corrected single-point calculations yielded some S_0/T_1 energy gaps, as shown in the following tables.

Table S9. S_0/T_1 average gaps for MECP1 and MECP2, obtained at different level of theories on ωB97X-3c/SMD(toluene) optimized geometries.

Method	MECP1			MECP2		
	**Gap range** [kJ/mol]	**Average** [kJ/mol]	**Median** [kJ/mol]	**Gap range** [kJ/mol]	**Average** [kJ/mol]	**Median** [kJ/mol]
SF BH&HLYP-D4/def2-TZVP	13.7 - 18.7	16.1	16	15.9 - 21.5	18.8	18.6
SF BH&HLYP-D4/def2-TZVP/SMD(toluene)	15.6 - 20.3	17.6	17.5	16.9 - 20.8	18.5	18.2
MRSF BH&HLYP/def2-TZVP	13.8 - 18.4	16.2	17.1	10.4 - 17.7	14.3	14.3
MRSF ωB97X-D4/def2-TZVP/	25.4 - 30.5	27.9	29.3	20.3 - 29.2	25.2	25.6
DLPNO-(U)CCSD(T)/aug-cc-pVTZ	11.6 - 17.9	14.1	13.5	9.2 - 15.7	12.0	11.9
CASPT2[a]/ANO-RCC-VTZP	0.1 - 6.0	3.4	3.6	1.7 - 8.5	5.6	6.1
XMS-CASPT2[a]/ANO-RCC-VTZP	0.8 - 7.8	3.3	2.8	0.8 - 6.6	2.8	2.0

[a] The active space chosen was (14,12), apart from NO_2 and CN, where a larger (16,14) was employed.

For WKB calculations, electronic energies of MECPs were calculated as an average between electronic energies of S_0 and T_1 states within a given method. The quality of the MECP geometries obtained is reflected by the small gaps obtained at the multireference levels of CASPT2 and XMS-CASPT2 (more on the choice of the DFT optimization level can be found in Section 8.2).

8.5 Calculation of the Spin-Orbit Coupling Matrix Elements

An essential part of the calculations within the WKB theory is the spin-orbit coupling matrix elements (SOCME, V_{SO}). We calculated these values at the XMS-CASPT2/ANO-RCC-VTZP//ωB97X-3c/SMD(toluene) level of theory using the Complete Active Space Interaction Approach (CASSI)[50] (see Table S10), which in the case of unsubstituted azobenzene resulted in values of 23.4 and 25.3 cm^{-1}, in accordance with the values reported in the literature: 18.6 and 23.1 cm^{-1} by Reimann *et al.*, at multireference configurational interaction/complete active space self-consistent field (MRCI/CASSCF) level,[35] 22.1 and 22.8 cm^{-1} by Martins *et al.*, at the n-electron valence state perturbation theory (NEVPT2/CASSCF) level,[36] 19.8 and 22.8 by Cembran *et al.* at the CASSCF level.[46] The values reported in Table S10 were used for computing the intersystem crossing rates (see Section 8.6).

We would like to add that neither the substitution pattern nor the choice of basis set seemed to modify significantly the values obtained (please refer to Table S11 for the results obtained from the smaller ANO-S-VDZP basis set). In particular, the average RMSD between the SOCME values computed with the ANO-RCC-VTZP and ANO-S-VDZP is 0.89 cm^{-1}.

Finally, we calculated the SOCME for each MECP at TDA(3 states)-PBE0-D4/def2-TZVP level. The obtained values, ranging from 25.0 to 29.5 cm^{-1}, are overestimated relative to the values we computed at the multireference XMS-CASPT2 level and to those reported in the literature; nonetheless, the method provides values that are close to the higher-level theory, enough to be considered for use with more complex molecules than monosubstituted azobenzenes.

Table S10. SOCME obtained at the XMS-CASPT2/ANO-RCC-VTZP//ωB97X-3c/SMD(toluene) level.

Substituent	MECP1 [cm^{-1}]	MECP2 [cm^{-1}]
Br	23.0	25.0
CF_3	24.0	26.3
CN	23.7	25.9
Cl	23.1	25.1
F	22.8	24.7
H	23.4	25.3
I	22.9	24.8
NMe_2	20.3	22.6
NO_2	24.3	26.6
OMe	21.4	23.6
*t*Bu	22.9	24.8

Table S11. SOCME obtained at the XMS-CASPT2/ANO-S-VDZP//ωB97X-3c/SMD(toluene) level.

Substituent	MECP1 [cm^{-1}]	MECP2 [cm^{-1}]
Br	22.1	24.2
CF_3	23.0	25.5
CN	22.9	25.2
Cl	21.9	24.2
F	22.0	23.8
H	22.6	24.5
I	22.1	24.2
NMe_2	19.4	21.7
NO_2	23.5	25.9
OMe	20.5	22.7
*t*Bu	22.0	23.9

Table S12. SOCME obtained at the TDA-PBE0-D4/def2-TZVP//ωB97X-3c/SMD(toluene) level.

Substituent	MECP1 [cm^{-1}]	MECP2 [cm^{-1}]
Br	25.5	26.3
CF_3	28.4	29.5
CN	28.0	29.2
Cl	27.2	28.2
F	27.7	28.6
H	28.2	29.1
I	-[a]	-[a]
NMe_2	25.0	25.8
NO_2	28.2	29.2
OMe	26.7	27.5
*t*Bu	27.8	28.7

[a] The value was not computed

8.6 Thermal Corrections to the Electronic Energy

In order to determine the thermal corrections to the non-adiabatic rotational path it is necessary to utilize the so-called *effective Hessian* at the MECP, which is provided directly from the MECP optimization and frequency analysis from ORCA (using the %SurfCrossNumFreq keyword).

To simulate the temperature dependence of the calculated Gibbs free energies, we applied the qRRHO model[51] with an in-house Python script *qrrho.py*, provided separately in the electronic supplementary materials. The correctness of the script was demonstrated by comparing energies, calculated by qrrho.py at elevated temperatures, against similar data calculated natively by ORCA 6.0.1. The obtained values fit within 2–3 kJ/mol, which we believe is within the accuracy of the applied methods.

Intersystem crossing rates were calculated at each MECP geometry as described in Section 8.4, using an in-house script, *kisc.py*, provided separately in the electronic supplementary materials.

To obtain the final values of thermal activation energies, a rate constant for each of the considered mechanisms was calculated in the range of temperatures from 298.15 K to 373 K, as given by the transition state theory (for adiabatic rotation and inversion) as:

$$k_{Rot,Inv}(T) = \frac{k_B T}{h} e^{\left(-\frac{\Delta G^{\ddagger}(T)}{RT}\right)}$$

or within WKB as described above in section 8.4. Gibbs free energy was scaled using an in-house script for quasi-rigid-rotor harmonic-oscillator model analysis based on the list of normal modes. To perform Eyring analysis of thus-obtained temperature-dependent rate constants (k_{Rot}, k_{Inv1}, k_{Inv2}, k_{NARot}, and total rate constant k_{tot} taken as the sum of individual k's), each of them was linearized in the coordinates "ln(k_i/T) – 1/T", representing the equation:

$$ln\frac{k}{T} = -\frac{\Delta H}{R} \cdot \frac{1}{T} + \left(\frac{\Delta S}{R} + ln\frac{k_B}{h}\right)$$

The intercept and slope of the line are determined using the least-squares method. Then, the effective enthalpy and entropy of activation ($\Delta H^{\ddagger}$ and $\Delta S^{\ddagger}$, respectively) can be found as:

$$\Delta H^{\ddagger} = -R \cdot slope$$

$$\Delta S^{\ddagger} = R \cdot \left(intercept - ln \frac{k_B}{h} \right)$$

Finally, the effective, total, Gibbs free energy of activation is derived as $\Delta G^{\ddagger} = \Delta H^{\ddagger} - T \cdot \Delta S^{\ddagger}$ and then used for further analysis.

The calculated branching ratios (see Table S16) are highly consistent across all methods that properly account for open-shell singlet and near-degenerate electronic structure, with the thermal back-isomerization proceeding almost exclusively via the non-adiabatic rotational pathway through the MECPs. Spin-flip and multireference approaches predict NARot to account for more than 97–99% of the reactive flux for nearly all substituents. Conventional DFT (ωB97X-3c) is a clear outlier, substantially overestimating adiabatic rotation. Most substituents show negligible method dependence; however, the NO_2 derivative exhibits a noticeably larger and strongly method-dependent inversion contribution, likely reflecting enhanced electronic perturbation and increased sensitivity to the treatment of near-degeneracy effects. Despite this, non-adiabatic rotation remains the dominant pathway for NO_2 across all methods beyond conventional DFT.

It is important to note that within all the applied methods (all used SF-DFT, MRSF-TDDFT combinations as well (U)CCSD(T) and (XMS-)CASPT2), the obtained total $\Delta S^{\ddagger}$ was negative, as listed in Table S13. The only method in which NARot was not the sole or predominant path was pure DFT (see Table S16); in this case, the absolute value of the activation entropy diminished, approaching 0. In all cases apart from pure DFT, CF_3 showed a lower entropy of activation value (halved) compared to the average within the method.

Table S13. $\Delta S^{\ddagger}$ [J/(mol K)] calculated on the ωB97X-3c/SMD(toluene) level geometries with energy corrections at different levels of theory. All possible pathways are considered and averaged.

**Substituent**	ωB97X-3c/ SMD(toluene)	MRSF-BH&HLYP/ def2-TZVP	MRSF-ωB97X-D4/ def2-TZVP	SF-BH&HLYP-D4/ def2-TZVP	SF-BH&HLYP-D4/ def2-TZVP/SMD	DLPNO-(U)CCSD(T)/ aug-cc-pVTZ	CASPT2[a]/ ANO-RCC-VTZP	XMS-CASPT2[a]/ ANO-RCC-VTZP
Br	-6.4	-29.2	-29.4	-29.2	-29.3	-29.7	-29.8	-29.6
CF3	-4.0	-13.3	-11.3	-13.3	-14.0	-14.4	-15.8	-15.8
Cl	-6.1	-29.3	-29.5	-29.3	-29.4	-29.9	-29.6	-29.6
CN	-6.9	-27.2	-23.4	-27.4	-28.4	-28.5	-27.6	-28.2
F	-5.5	-29.7	-29.6	-29.6	-29.7	-30.2	-30.6	-30.2
H	-5.6	-29.0	-28.9	-29.0	-29.1	-29.6	-30.1	-29.6
I	-	-	-	-	-	-	-32.4	-30.2
NMe_2	-3.9	-42.8	-41.4	-42.2	-42.7	-42.9	-32.7	-42.7
NO_2	-6.7	-18.2	-14.9	-20.5	-23.9	-22.0	-28.8	-29.3
OMe	-4.3	-30.0	-29.8	-29.8	-29.9	-30.5	-30.2	-30.5
tBu	-6.4	-28.8	-29.7	-29.2	-29.1	-30.4	-32.7	-30.9

[a] The active space chosen was (14,12), apart from NO_2 and CN, where a larger (16,14) was employed.

Table S14. ΔG‡ [kJ/mol] calculated on the ωB97X-3c/SMD(toluene) level geometries with energy corrections at different levels of theory. All possible pathways are considered and averaged.

Substituent	ωB97X-3c/ SMD(toluene)	MRSF-BH&HLYP/ def2-TZVP	MRSF-ωB97X-D4/ def2-TZVP	SF-BH&HLYP-D4/ def2-TZVP	SF-BH&HLYP-D4/ def2-TZVP/SMD	DLPNO- (U)CCSD(T)/ aug-cc-pVTZ	CASPT2[a]/ ANO-RCC-VTZP	XMS-CASPT2[a]/ ANO-RCC-VTZP
Br	72.7	64.8	77.3	84.5	83.2	84.6	65.2	66.0
CF3	74.7	73.2	85.0	92.3	90.6	92.6	70.6	71.3
Cl	72.5	65.0	76.9	84.2	82.9	84.5	65.4	66.1
CN	72.3	64.8	77.4	83.5	81.8	83.8	65.1	65.6
F	72.9	65.6	78.5	85.1	83.6	85.6	64.2	65.4
H	74.0	66.0	78.2	85.6	83.9	85.4	65.7	66.6
I	-	-	-	-	-	-	63.0	65.3
NMe_2	67.6	58.0	70.5	75.8	70.3	78.9	60.7	58.6
NO_2	72.9	69.6	81.4	86.2	83.0	89.4	63.3	64.2
OMe	70.8	64.8	78.7	84.3	81.7	84.6	61.5	63.7
tBu	72.0	66.0	76.6	84.9	82.7	84.4	63.1	65.3

[a] The active space chosen was (14,12), apart from NO_2 and CN, where a larger (16,14) was employed.

Table S15. ΔG‡ [kJ/mol] calculated on the ωB97X-3c/SMD(toluene) level geometries with energy corrections at different levels of theory. All possible pathways are considered and averaged.

Substituent	ωB97X-3c/ SMD(toluene)	MRSF-BH&HLYP/ def2-TZVP	MRSF-ωB97X-D4/ def2-TZVP	SF-BH&HLYP-D4/ def2-TZVP	SF-BH&HLYP-D4/ def2-TZVP/SMD	DLPNO- (U)CCSD(T)/ aug-cc-pVTZ	CASPT2[a]/ ANO-RCC-VTZP	XMS-CASPT2[a]/ ANO-RCC-VTZP
Br	61.4	64.8	77.2	84.4	83.2	84.6	64.4	65.8
CF3	69.5	72.8	83.0	91.8	90.4	92.1	70.2	71.0
Cl	61.1	65.0	76.7	84.1	82.9	84.5	64.5	65.9
CN	61.1	63.7	74.0	82.4	81.3	83.1	63.0	64.6
F	61.2	65.5	78.3	85.0	83.5	85.6	63.7	65.2
H	62.4	66.0	77.9	85.5	83.9	85.4	65.0	66.4
I	-	-	-	-	-	-	62.2	65.1
NMe_2	48.8	57.7	69.3	75.2	70.0	78.6	53.8	58.2
NO_2	61.7	63.6	73.5	81.4	80.1	85.2	62.1	64.0
OMe	58.7	64.7	78.3	84.1	81.5	84.6	60.8	63.6
tBu	60.5	66.0	76.5	84.8	82.7	84.4	62.4	65.2

[a] The active space chosen was (14,12), apart from NO_2 and CN, where a larger (16,14) was employed.

Table S16. Percentage of the computed thermal pathways (ΣInv as a sum of Inv1 and Inv2 contributions, Rot and NARot) calculated on the ωB97X-3c/SMD(toluene) level geometries with energy corrections at different levels of theory.

		ωB97X-3c/SMD	MRSF-BH&HLYP[a]	MRSF-ωB97X-D4[a]	SF-BH&HLYP-D4[a]	SF-BH&&HLYP-D4/SMD[a]	DLPNO(U)CCSD(T)[b]	CASPT2[c]	XMS-CASPT2[c]
Br	ΣInv	0.00	0.01	0.08	0.02	0.00	0.01	0.00	0.01
	Rot	64.64	0.00	0.00	0.02	0.03	0.00	0.79	0.10
	NA-Rot	35.36	99.99	99.92	99.96	99.97	99.99	99.21	99.90
CF3	ΣInv	0.00	0.37	2.76	0.52	0.11	0.38	0.13	0.17
	Rot	40.73	0.00	0.00	0.00	0.01	0.00	0.17	0.02
	NA-Rot	59.26	99.63	97.24	99.48	99.88	99.62	99.70	99.82
Cl	ΣInv	0.00	0.01	0.08	0.01	0.00	0.00	0.00	0.00
	Rot	65.96	0.00	0.00	0.03	0.03	0.00	0.88	0.11
	NA-Rot	34.04	99.99	99.92	99.96	99.97	100.00	99.12	99.88
CN	ΣInv	0.02	1.35	6.38	1.23	0.44	0.66	0.64	0.87
	Rot	60.64	0.00	0.00	0.01	0.01	0.00	1.73	0.07
	NA-Rot	39.34	98.65	93.62	98.76	99.55	99.34	97.62	99.06
F	ΣInv	0.00	0.01	0.13	0.03	0.00	0.00	0.00	0.00
	Rot	68.40	0.00	0.00	0.03	0.03	0.00	0.48	0.08
	NA-Rot	31.60	99.99	99.87	99.94	99.96	100.00	99.52	99.91
H	ΣInv	0.00	0.01	0.16	0.03	0.00	0.02	0.00	0.01
	Rot	65.37	0.00	0.00	0.02	0.02	0.00	0.61	0.09
	NA-Rot	34.63	99.99	99.84	99.95	99.97	99.98	99.38	99.90
I	ΣInv							0.01	0.01
	Rot							0.72	0.09
	NA-Rot							99.27	99.90
NMe_2	ΣInv	0.00	0.06	0.69	0.07	0.00	0.09	0.00	0.02
	Rot	85.12	0.03	0.00	0.23	0.17	0.00	9.33	0.18
	NA-Rot	14.88	99.91	99.31	99.70	99.83	99.91	90.67	99.80
NO_2	ΣInv	0.19	15.08	23.88	10.45	5.00	8.34	0.02	0.04
	Rot	58.92	0.00	0.00	0.01	0.01	0.00	1.33	0.02
	NA-Rot	40.89	84.91	76.12	89.54	94.99	91.66	98.65	99.94
OMe	ΣInv	0.00	0.02	0.25	0.04	0.00	0.03	0.00	0.00
	Rot	74.80	0.01	0.00	0.09	0.11	0.00	0.63	0.08
	NA-Rot	25.20	99.98	99.75	99.86	99.89	99.97	99.37	99.92
tBu	ΣInv	0.00	0.01	0.04	0.01	0.00	0.01	0.00	0.00
	Rot	62.61	0.00	0.00	0.02	0.03	0.00	0.55	0.08
	NA-Rot	37.39	99.99	99.96	99.96	99.97	99.99	99.44	99.91

[a] Basis set def2-TZVP

[b] Basis set aug-cc-pVTZ.

[c] The active space chosen was (14,12), apart from NO_2 and CN, where a larger (16,14) was employed. Basis set: ANO-RCC- VTZP.

8.6.1 Error Analysis

For a given method m, with N available substituents, we use the formula of the Mean Absolute Error (MAE):

$$\text{MAE}_m = \frac{1}{N}\sum_{i=1}^{N} \mid \Delta G^{\ddagger}_{m,i} - \Delta G^{\ddagger}_{\text{exp},i} \mid$$

with $\Delta G^{\ddagger}_{m,i}$ the computed activation energy for the ith substituent computed with method m, and $\Delta G^{\ddagger}_{\text{exp},i}$ the experimental activation energy for the same substituent.

We also used the following formula to obtain the Root Mean Square Error of the computed activation Free energies with respect to experiment:

$$\text{RMSE}_m = \sqrt{\frac{1}{N}\sum_{i=1}^{N}\left(\Delta G^{\ddagger}_{m,i} - \Delta G^{\ddagger}_{\text{exp},i}\right)^2}$$

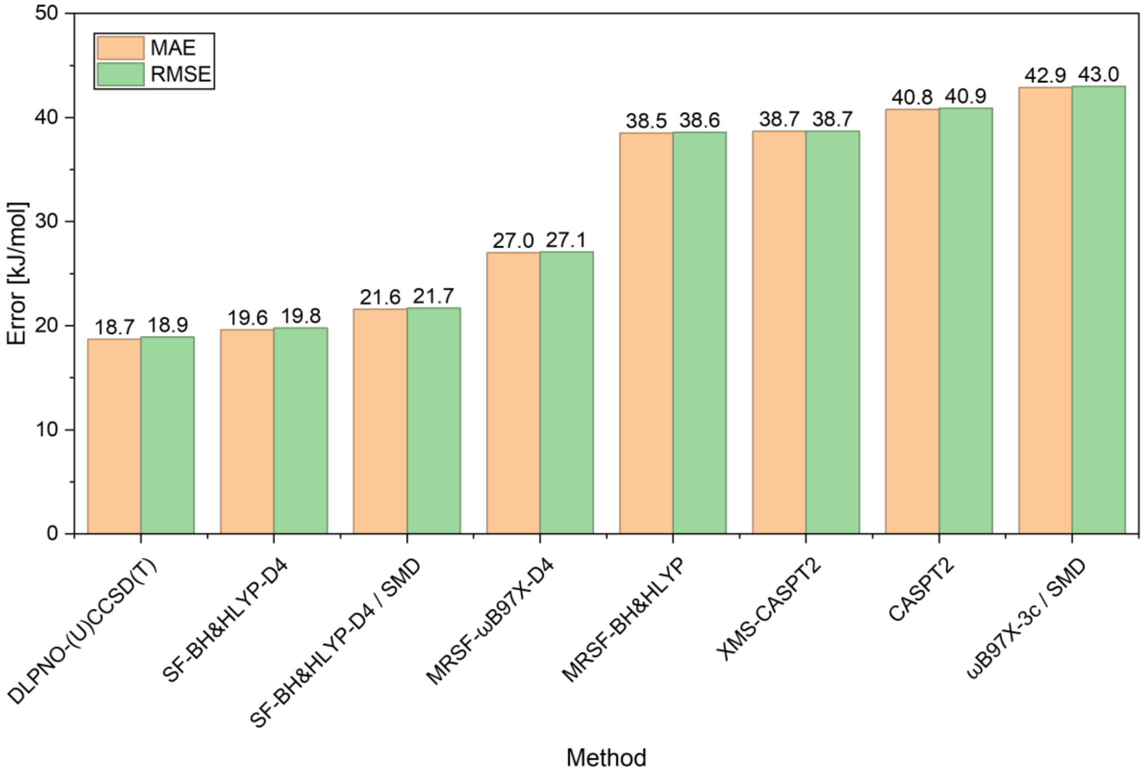

Figure S68. MAE and RMSE of the computed activation Free energies with respect to experimental ones.

The MAE measures the average deviation from the experimental data. At the same time, the RMSE weights larger errors more heavily and is often used to assess both bias and scatter relative to experimental reference values. We found that all methods systematically underestimate the experimental activation energies. From the calculations, three methods can be seen as comparably good: DLPNO-(U)CCSD(T) and SF-BH&HLYP-D4 in gas phase and SMD, yield MAEs of approximately 19–22 kJ/mol and comparable RMSE values. The remaining approaches show progressively larger deviations from experiment. MRSF-ωB97X-

D4 exhibits intermediate performance, with MAE and RMSE values around 27 kJ/mol. This reflects an improved description of near-degenerate electronic structure relative to conventional DFT, but also highlights a sensitivity to the underlying exchange-correlation functional, which in this case appears to compress the barrier heights. MRSF-BH&HLYP performs significantly worse, with MAEs approaching 40 kJ/mol, consistent with an overall overstabilization of open-shell singlet character and an excessive lowering of the potential energy surface in the transition-state and crossing regions. Similar underestimation magnitudes are observed for CASPT2 and XMS-CASPT2, yielding MAEs of approximately 39–41 kJ/mol. This behavior is consistent with the known tendency of CASPT2-based methods (particularly when a fixed active space is employed) to compress absolute barrier heights, despite providing a qualitatively correct description of the electronic structure and mechanistic pathways. Finally, the ωB97X-3c/SMD protocol exhibits the largest deviations from experiment (MAE > 40 kJ/mol), reflecting the combined limitations of a small basis set and a broken-symmetry DFT description of activation energies in systems with significant multireference character.

Nevertheless, the similarities between the two errors hold for all the methods, indicating that the computed activation energies deviate from experiment in a largely systematic fashion rather than being dominated by isolated outliers.

Despite quantitative differences in absolute activation energies, all methods that explicitly account for open-shell singlet and near-degenerate electronic structures yield a qualitatively consistent mechanistic picture of thermal back-isomerization. In particular, these approaches systematically identify access to the minimum-energy crossing seam as energetically preferred over purely adiabatic rotation, with inversion remaining a minor pathway across the substituent series. Even methods that substantially underestimate absolute barriers, such as CASPT2, XMS-CASPT2, and MRSF-based approaches, preserve the relative ordering of the relevant stationary points and predict comparable branching trends. Conversely, deviations from this mechanistic consensus are primarily associated with (BS-)DFT, which tend to overstabilize symmetry-broken rotational configurations. This overall agreement indicates that, while absolute barrier heights are method-dependent (as expected), the qualitative mechanistic conclusions are robust across a broad range of multireference and spin-flip electronic-structure frameworks.

8.7 Computed LFER Plots

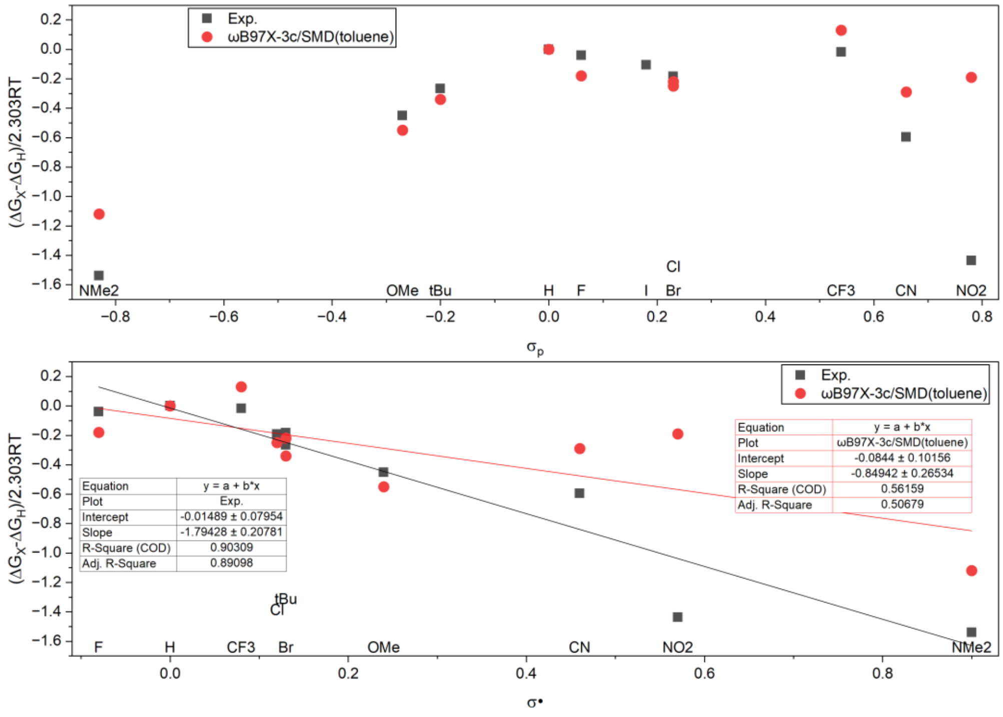

Figure S69. Computed *vs*. experimental LFER at the ωB97X-3c/SMD(toluene) level of theory plotted *vs.* the Hammett σ_p (top) and Creary σ· (bottom) parameter.

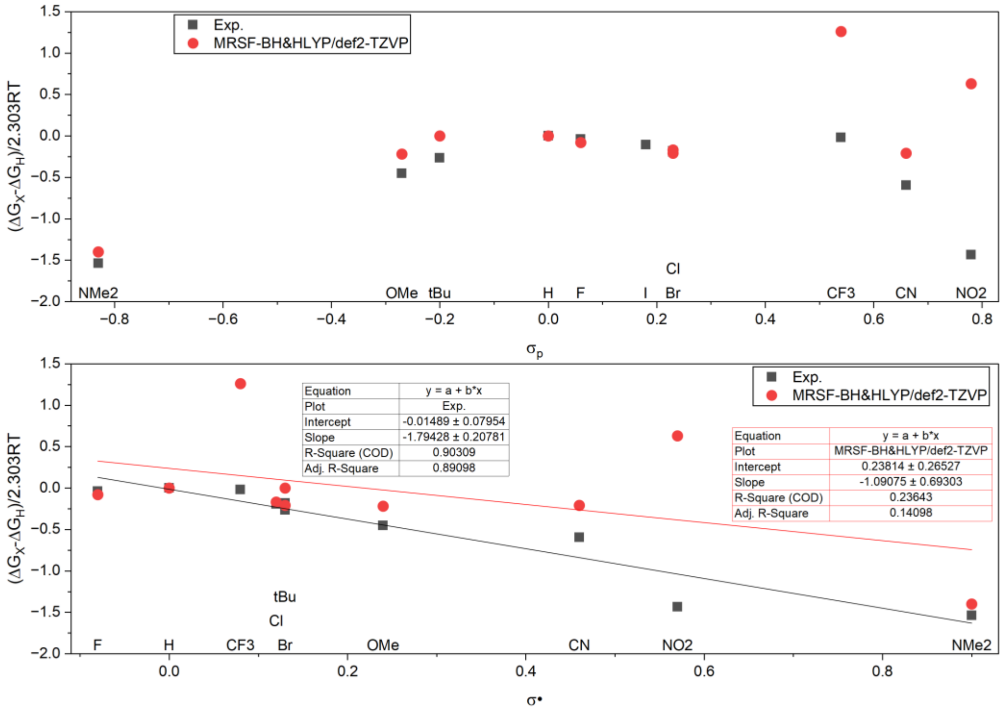

Figure S70. Computed *vs*. experimental LFER at the MRSF-BH&HLYP/def2-TZVP//ωB97X-3c/SMD(toluene) level of theory plotted *vs.* the Hammett σ_p (top) and Creary σ· (bottom) parameter.

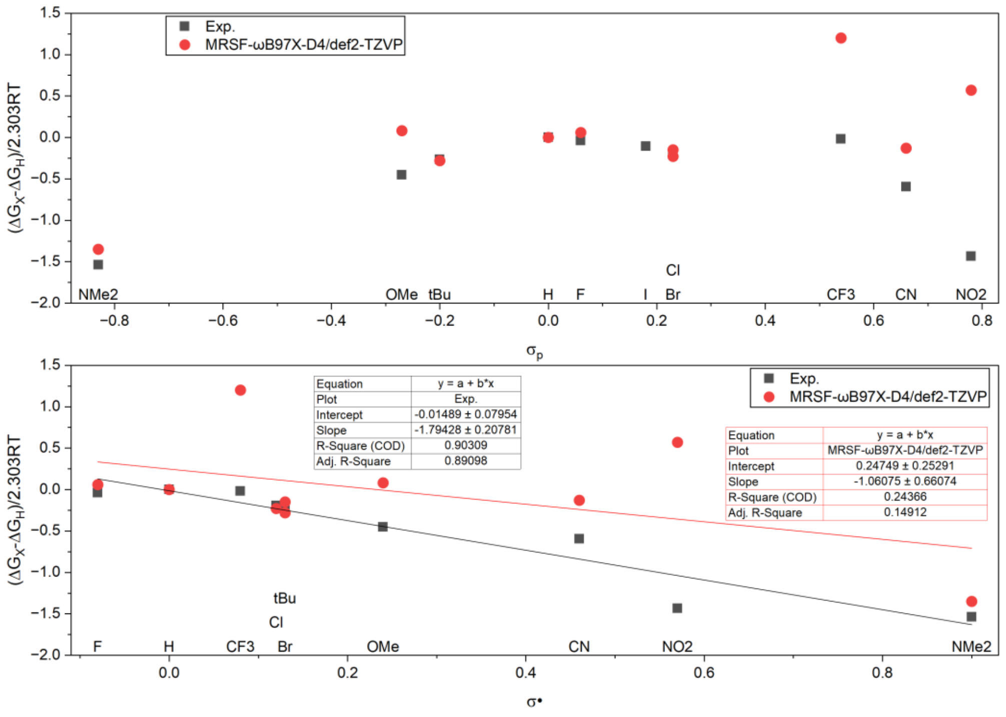

Figure S71. Computed *vs*. experimental LFER at the MRSF-ωB97X-D4/def2-TZVP//ωB97X-3c/SMD(toluene) level of theory plotted *vs.* the Hammett σ_p (top) and Creary σ· (bottom) parameter.

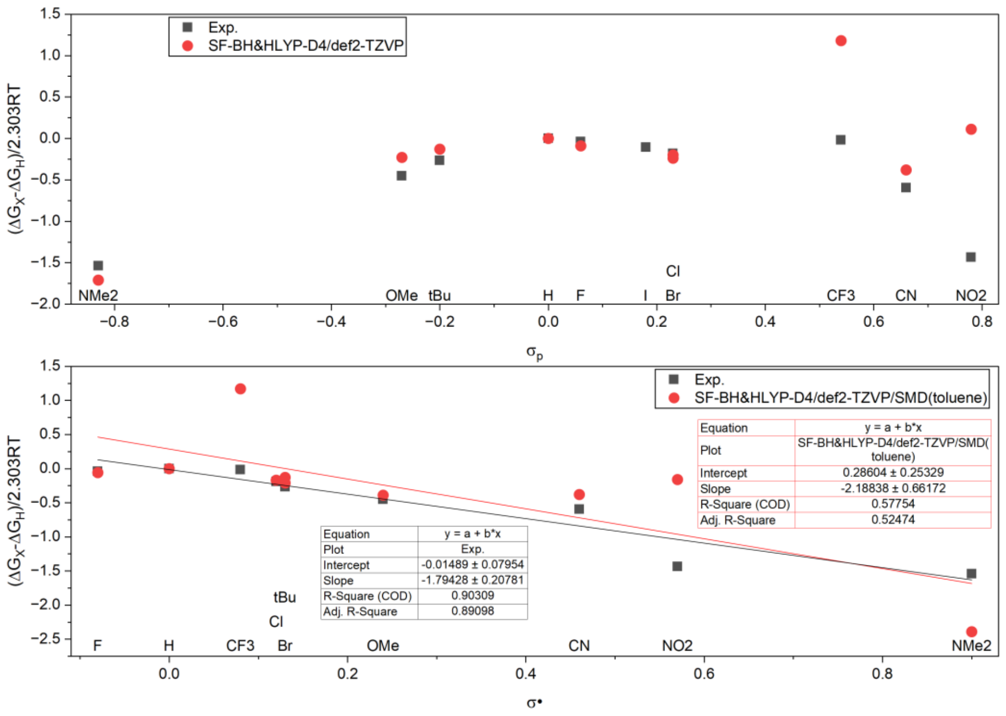

Figure S72. Computed *vs*. experimental LFER at the SF-BH&HLYP-D4/def2-TZVP//ωB97X-3c/SMD(toluene) level of theory plotted *vs.* the Hammett σ_p (top) and Creary σ· (bottom) parameter.

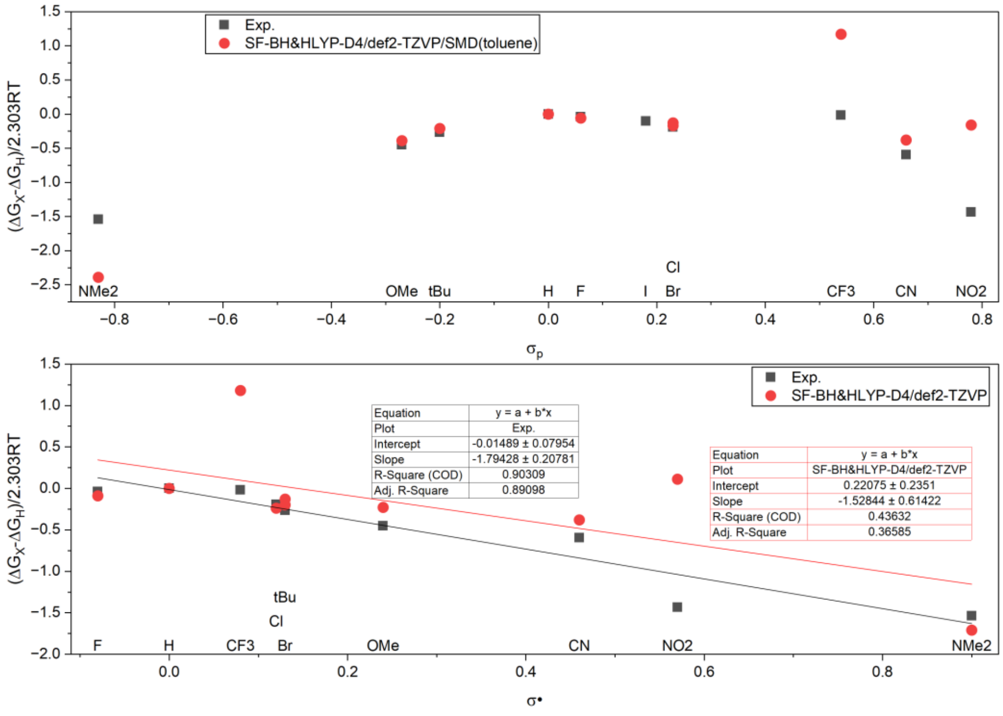

Figure S73. Computed *vs*. experimental LFER at the SF-BH&HLYP-D4/def2-TZVP/SMD(toluene) //ωB97X-3c/SMD(toluene) level of theory plotted *vs*. the Hammett σ_p (top) and Creary σ· (bottom) parameter.

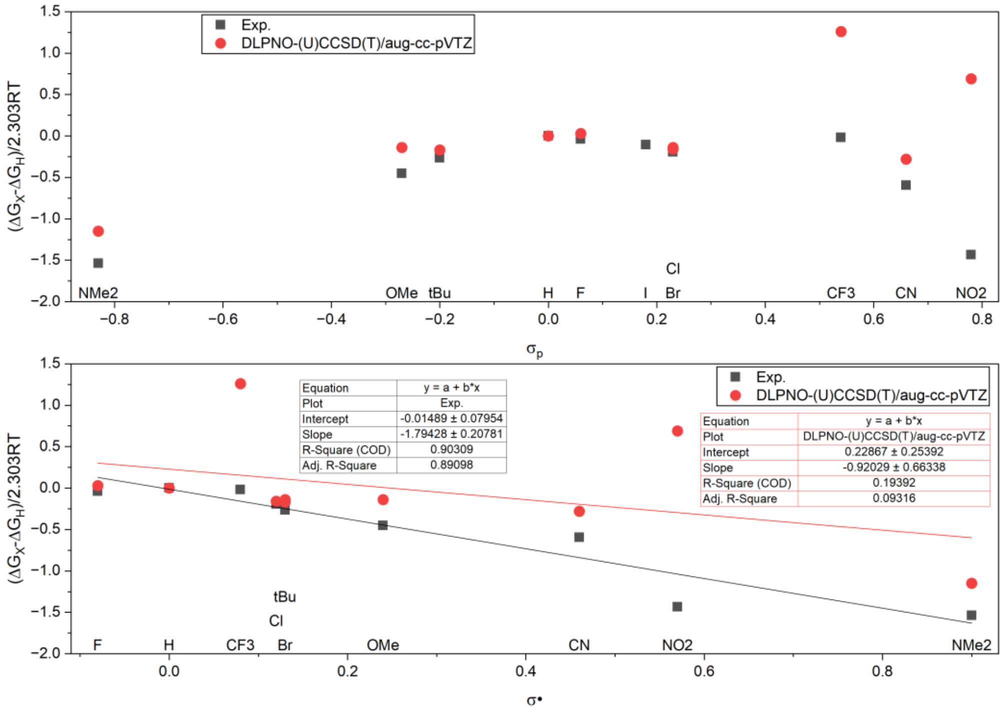

Figure S74. Computed *vs*. experimental LFER at the DLPNO-(U)CCSD(T)/aug-cc-pVTZ//ωB97X-3c/SMD(toluene) level of theory plotted *vs*. the Hammett σ_p (top) and Creary σ· (bottom) parameter.

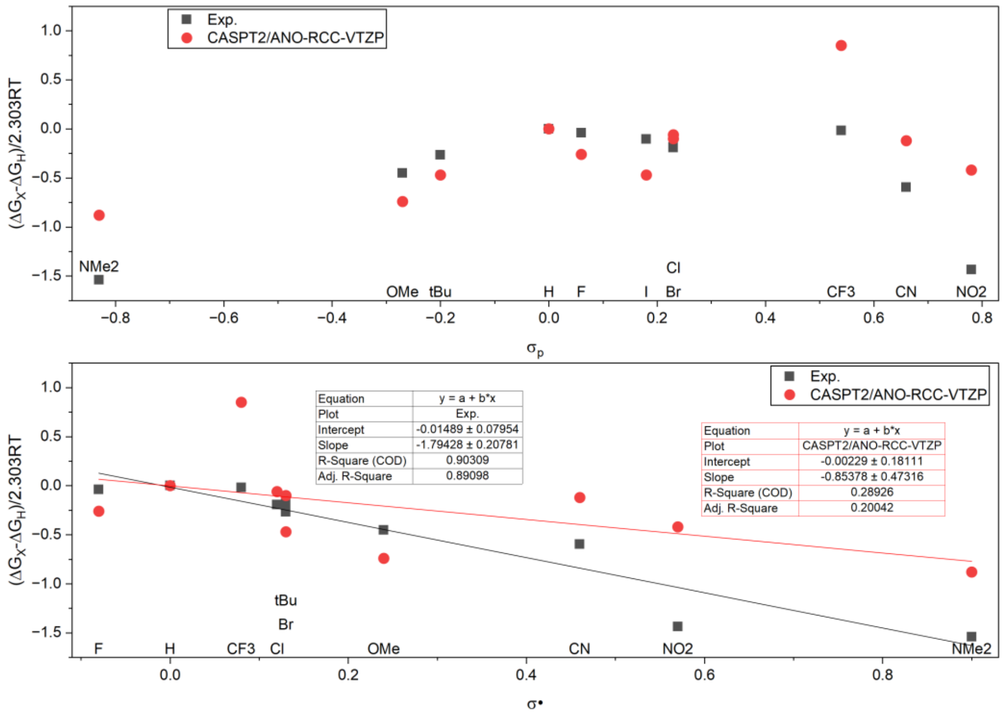

Figure S75. Computed *vs*. experimental LFER at the CASPT2/ANO-RCC-VTZP//ωB97X-3c/SMD(toluene) level of theory plotted *vs*. the Hammett σ_p (top) and Creary σ· (bottom) parameter.

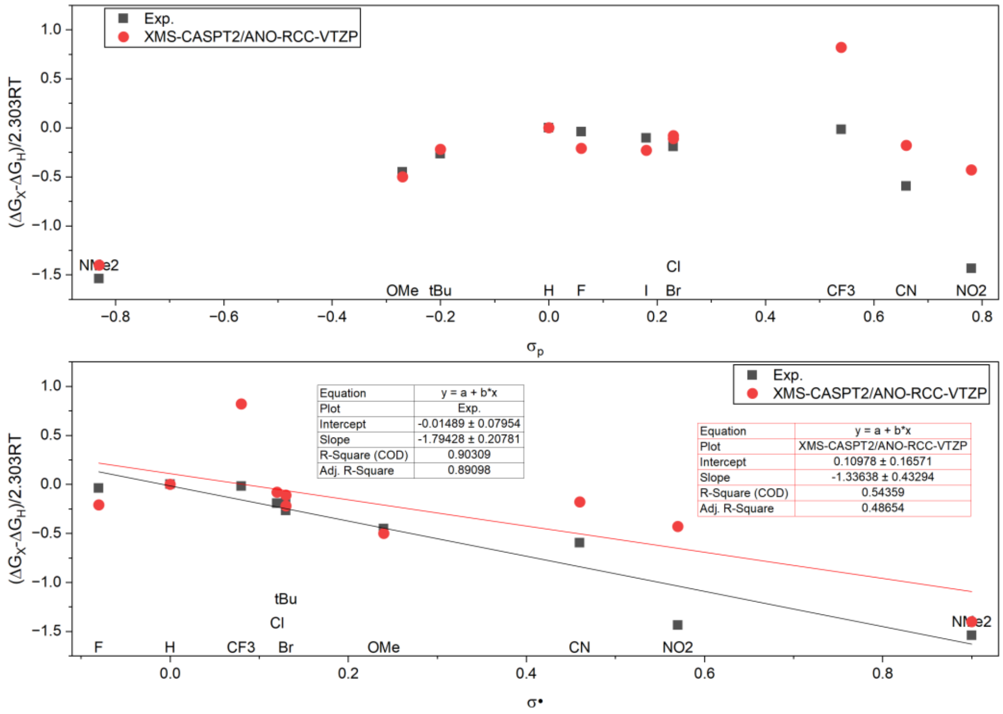

Figure S76. Computed *vs*. experimental LFER at the XMS-CASPT2/ANO-RCC-VTZP//ωB97X-3c/SMD(toluene) level of theory plotted *vs*. the Hammett σ_p (top) and Creary σ· (bottom) parameter.

The previous Figures compare the experimental and computed LFER values as a function of substituent parameters, using both Hammett σ_p and Creary $\sigma\cdot$ parameters. As expected for a process involving substantial electronic reorganization and partial diradical character, the experimental data show only weak correlation with Hammett σ_p, whereas a significantly improved linearity is obtained when Creary $\sigma\cdot$ is employed. Consequently, the latter was used for quantitative comparison. Among the tested methods, SF-BH&HLYP-D4 yields the highest formal correlation coefficient with experiment (R^2 *ca.* 0.57), reflecting its ability to reproduce the overall monotonic trend across the substituent series (see also Section 8.6.1). However, closer inspection of the Creary plots reveals that the central portion of the dataset partly drives this correlation, while deviations become more pronounced for strongly electron-withdrawing substituents. In contrast, XMS-CASPT2, despite exhibiting a slightly lower R^2 value (ca. 0.54), provides a more balanced qualitative description across the full electronic range, particularly in the electron-withdrawing region, where several other methods either compress the response or deviate in sign or magnitude. This distinction highlights that, for LFER analysis, the correlation coefficient alone does not fully capture the quality of agreement, as methods with similar or even higher R^2 may still exhibit systematic distortions in specific substituent classes. Notably, the R^2 values obtained here are within the range reported in previous computational LFER studies of photochemical and non-adiabatic processes (*e.g.*, R^2 = 0.31 in related work by González and co-workers[52]), underscoring the intrinsic difficulty of reproducing substituent sensitivities in systems governed by near-degenerate electronic structure. Overall, these plots indicate that XMS-CASPT2 offers a more physically consistent representation of substituent effects across the entire electronic spectrum, whereas SF-BH&HLYP-D4 provides a competitive but less uniform description, particularly when strongly electron-withdrawing groups are considered.

8.8 Active Spaces

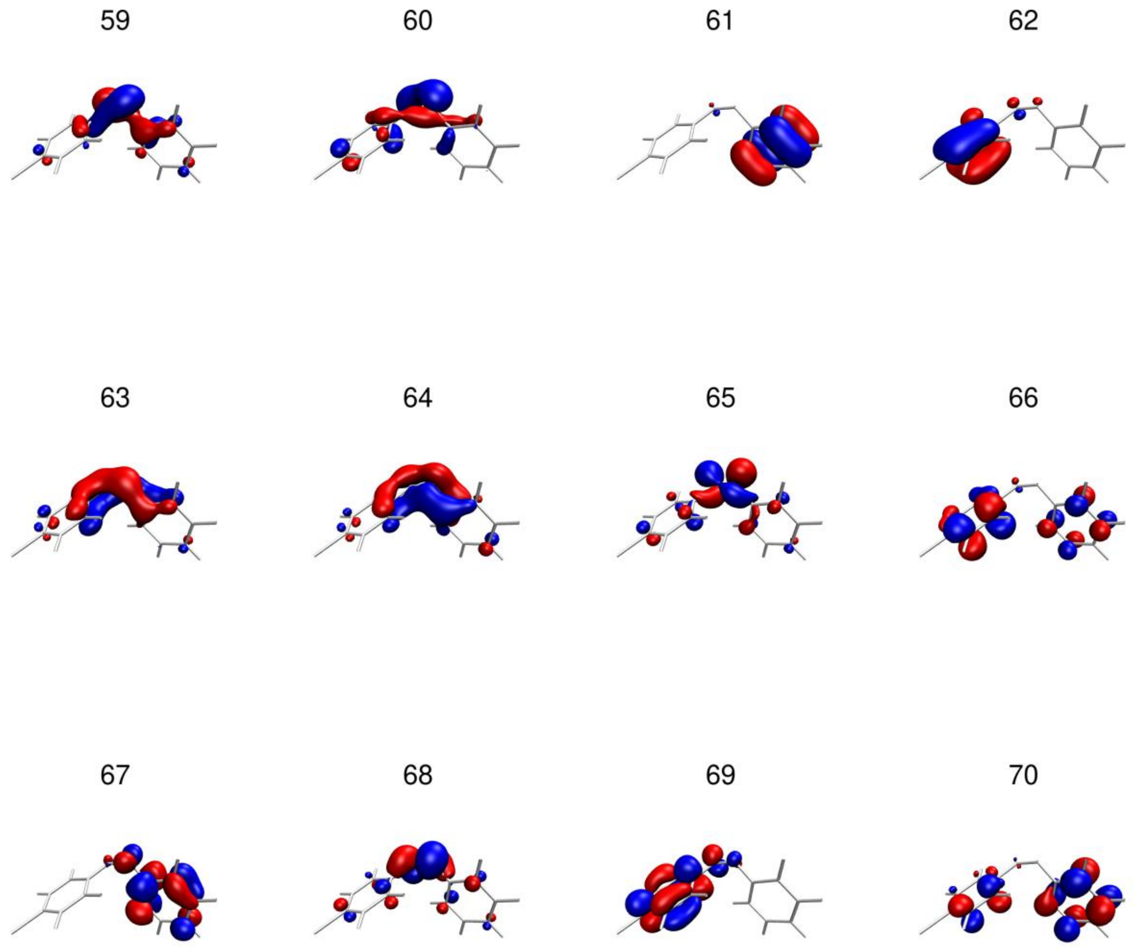

Figure S77. Active space of the MECP1 of bromoazobenzene at the CASSCF/ANO-RCC-VTZP//ωB97X-3c/SMD(toluene) level of theory. The digits represent the orbital number.

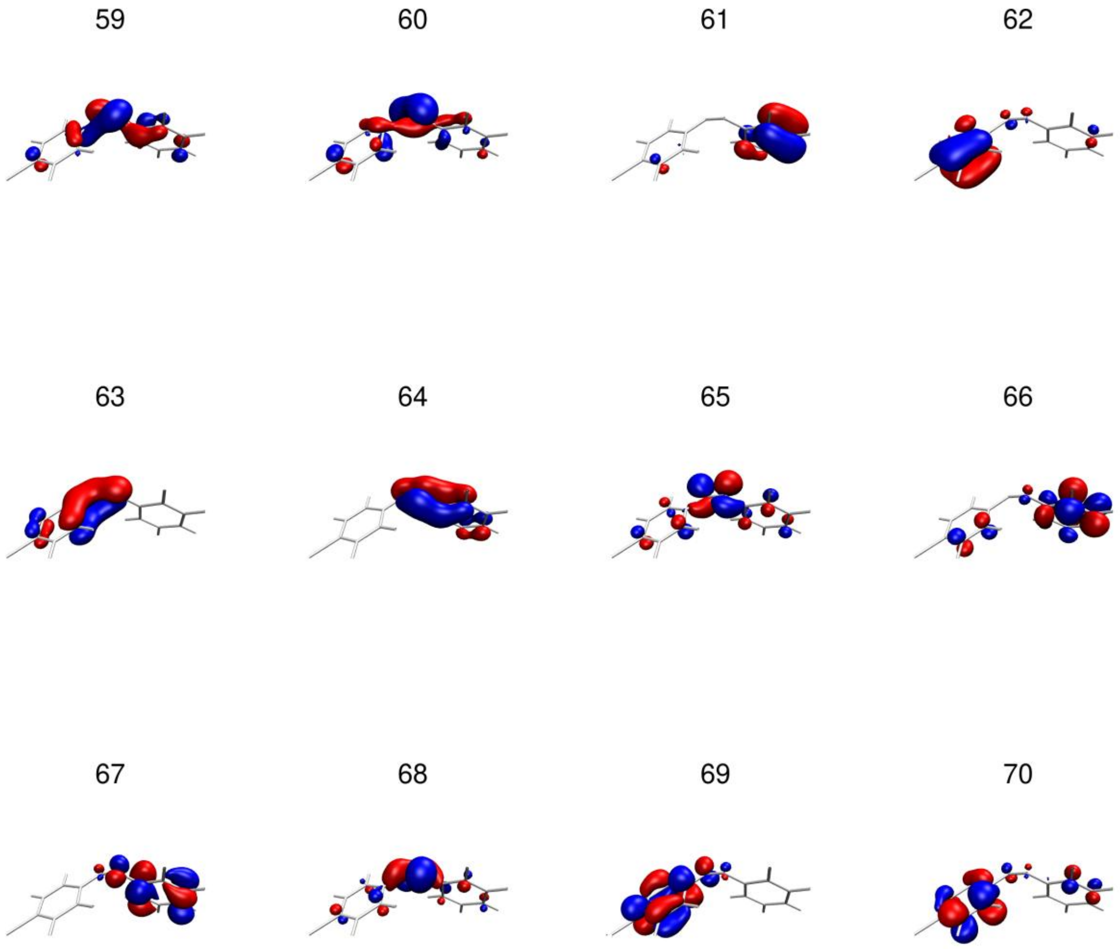

Figure S78. Active space of the MECP2 of bromoazobenzene at the CASSCF/ANO-RCC-VTZP//ωB97X-3c/SMD(toluene) level of theory. The digits represent the orbital number.

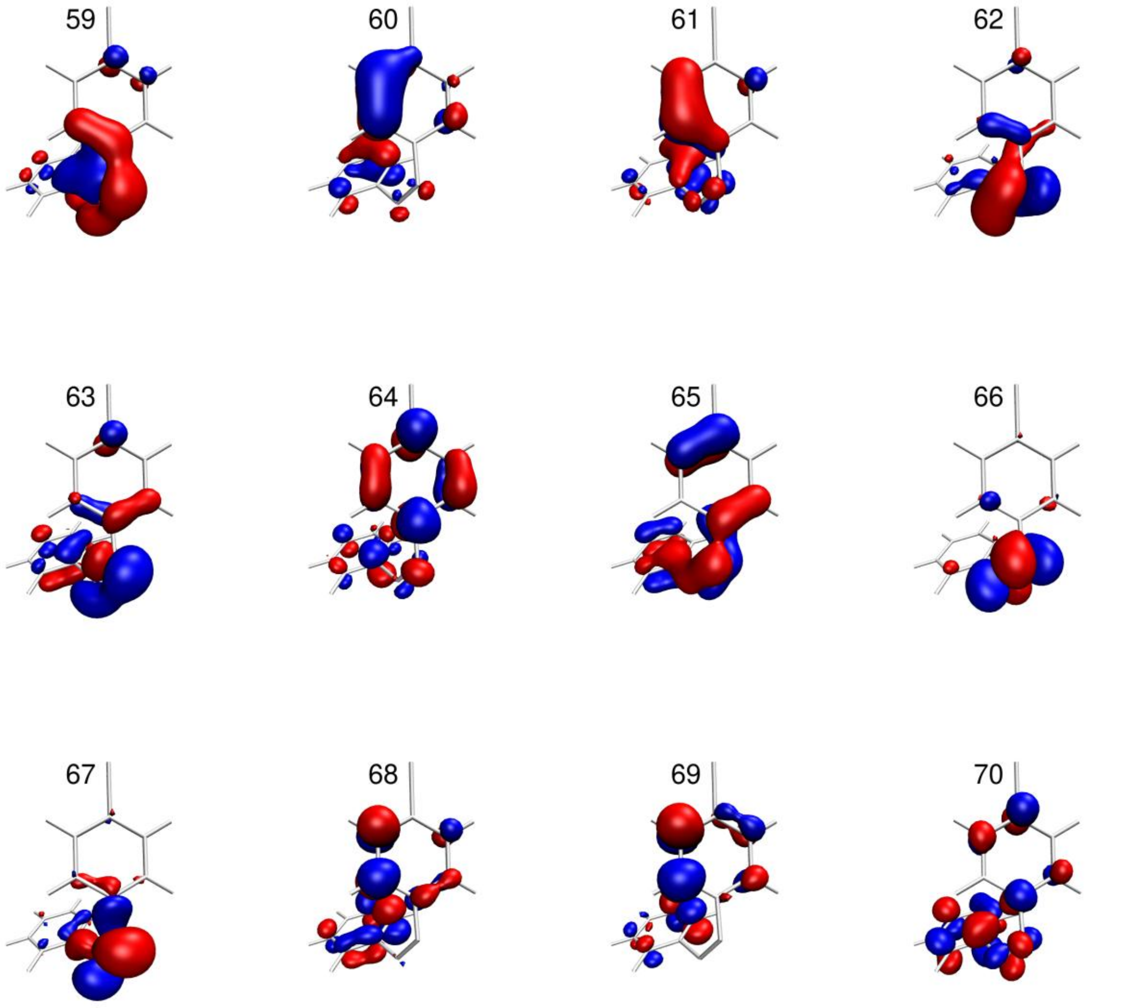

Figure S79. Active space of the Z-form of bromoazobenzene at the CASSCF/ANO-RCC-VTZP//ωB97X-3c/SMD(toluene) level of theory. The digits represent the orbital number.

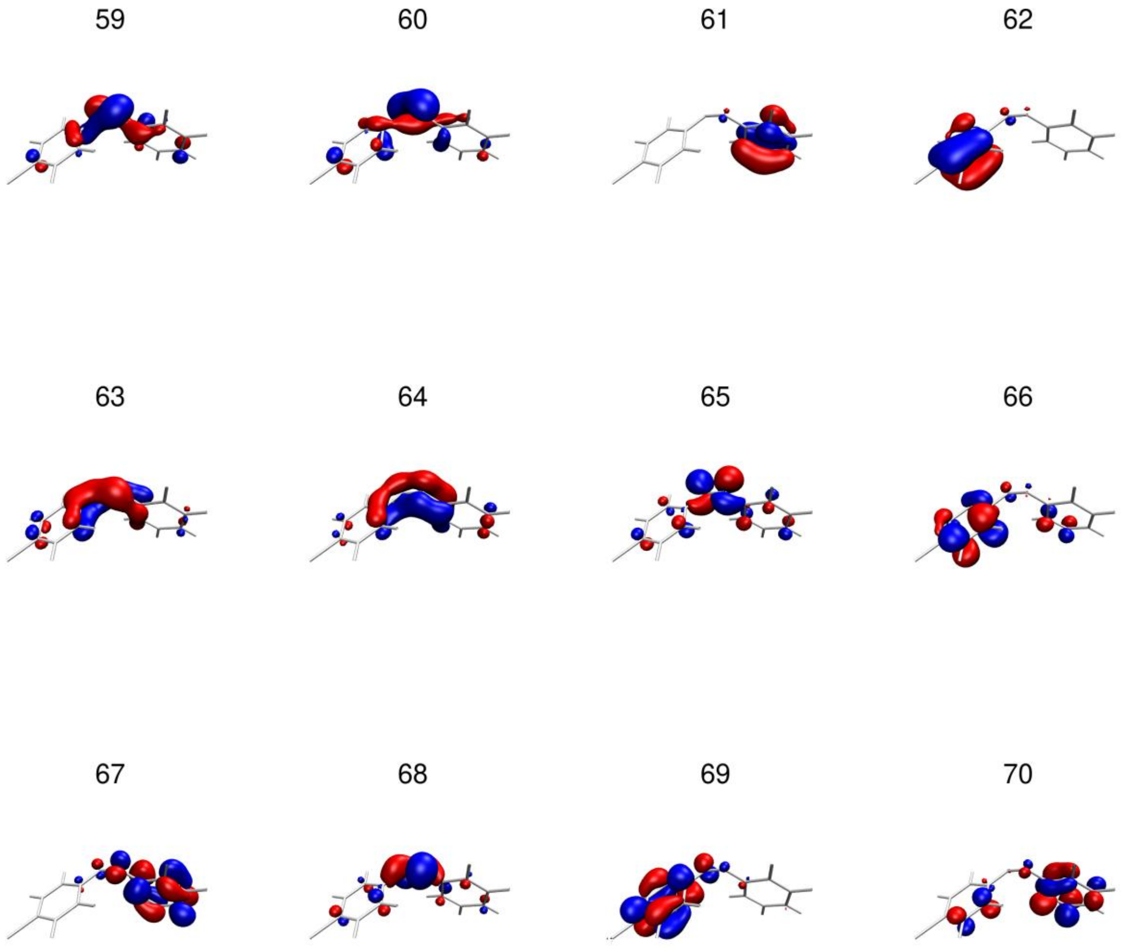

Figure S80. Active space of the T_1 minimum of bromoazobenzene at the CASSCF/ANO-RCC-VTZP//ωB97X-3c/SMD(toluene) level of theory. The digits represent the orbital number.

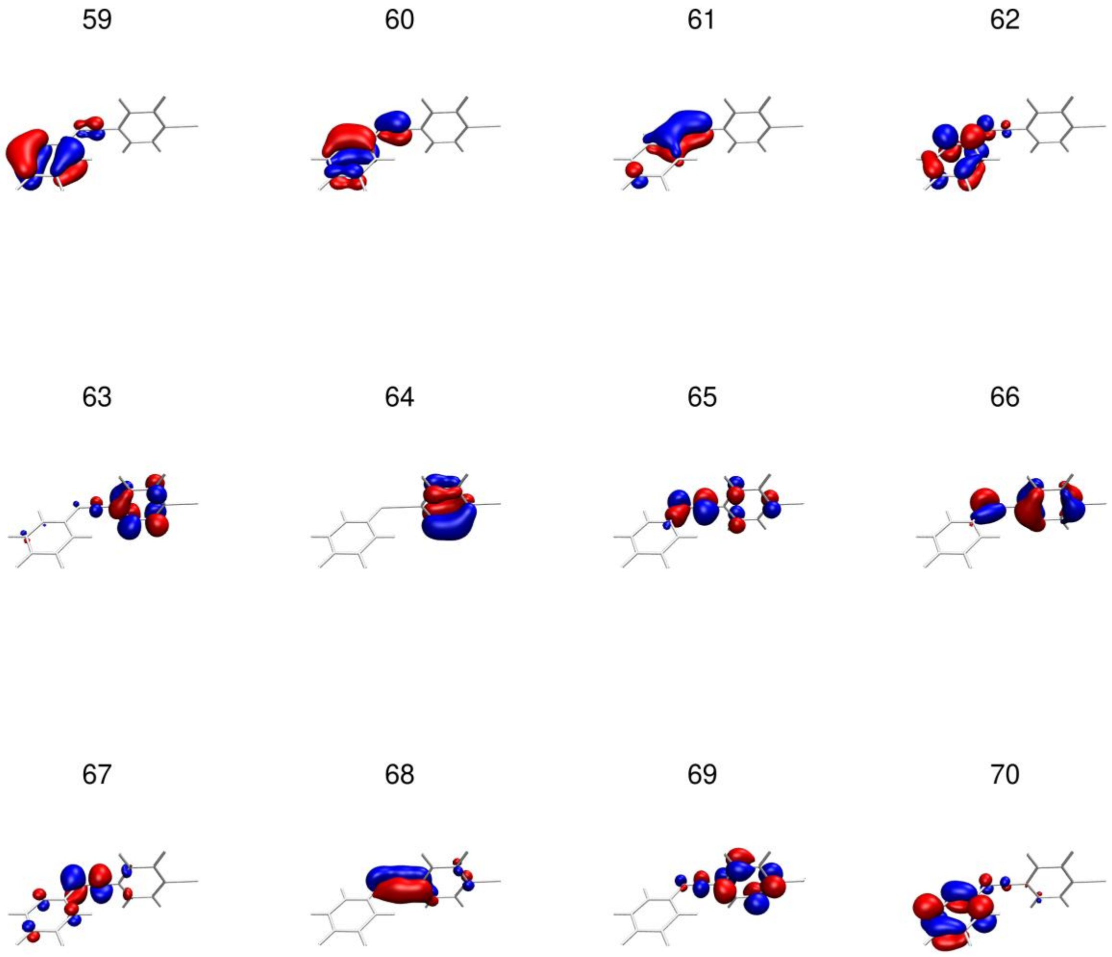

Figure S81. Active space of the TSInv1 of bromoazobenzene at the CASSCF/ANO-RCC-VTZP//ωB97X-3c/SMD(toluene) level of theory. The digits represent the orbital number.

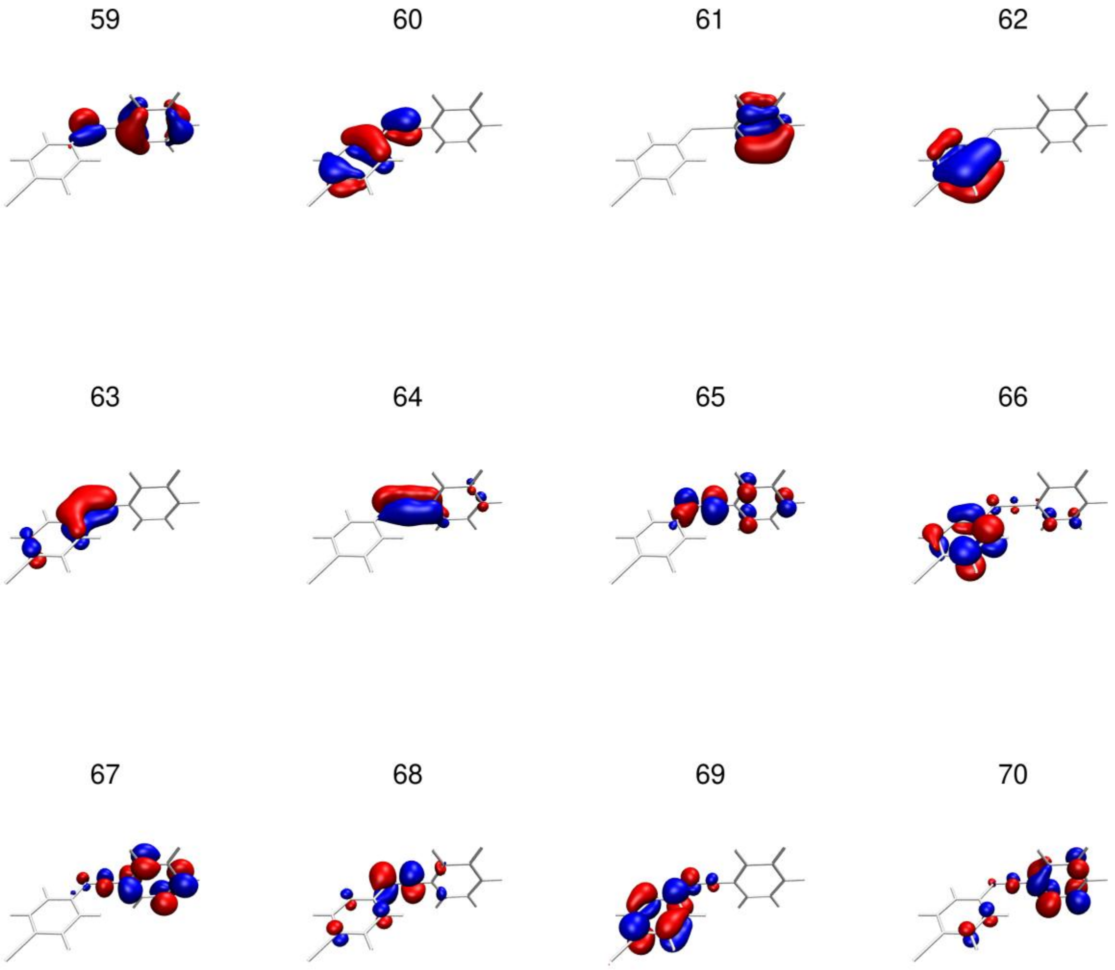

Figure S82. Active space of the TSInv2 of bromoazobenzene at the CASSCF/ANO-RCC-VTZP//ωB97X-3c/SMD(toluene) level of theory. The digits represent the orbital number.

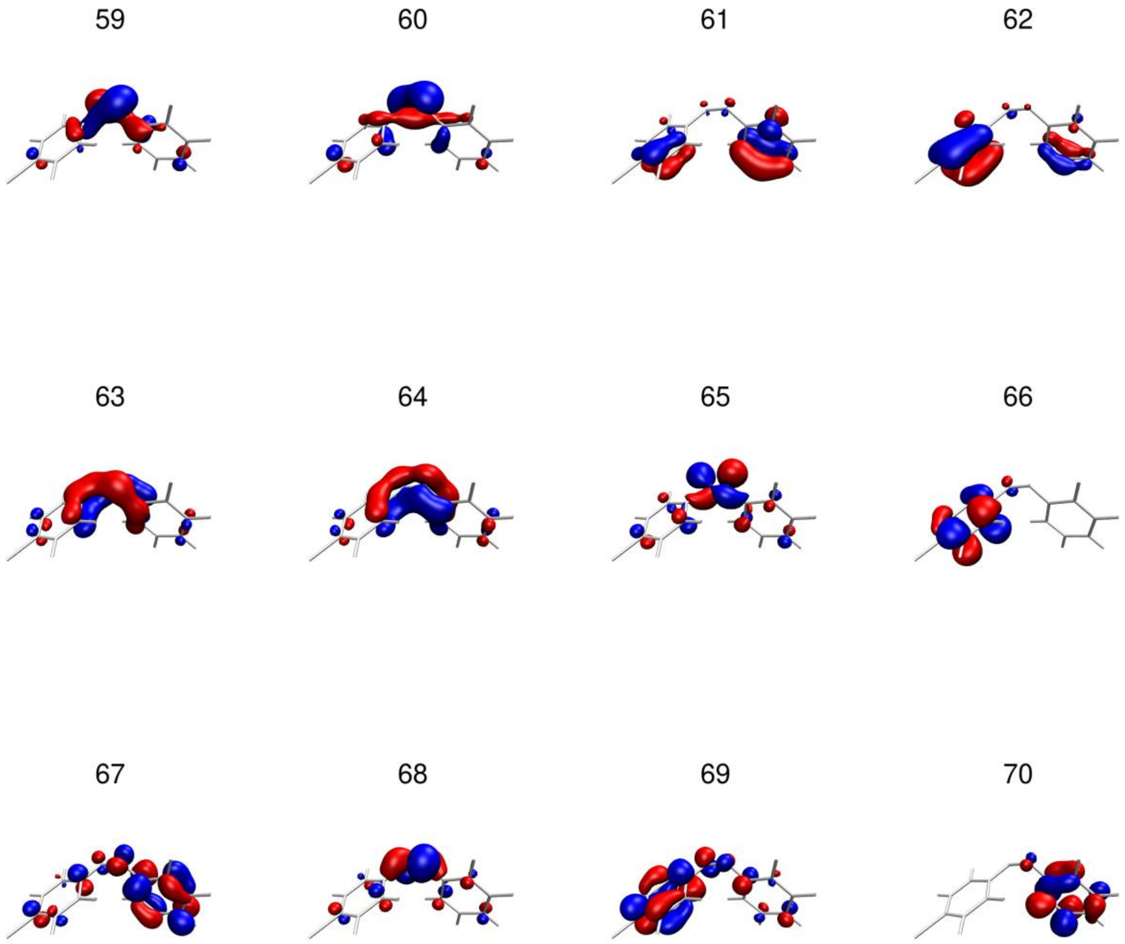

Figure S83. Active space of the TSRot of bromoazobenzene at the CASSCF/ANO-RCC-VTZP//ωB97X-3c/SMD(toluene) level of theory. The digits represent the orbital number.

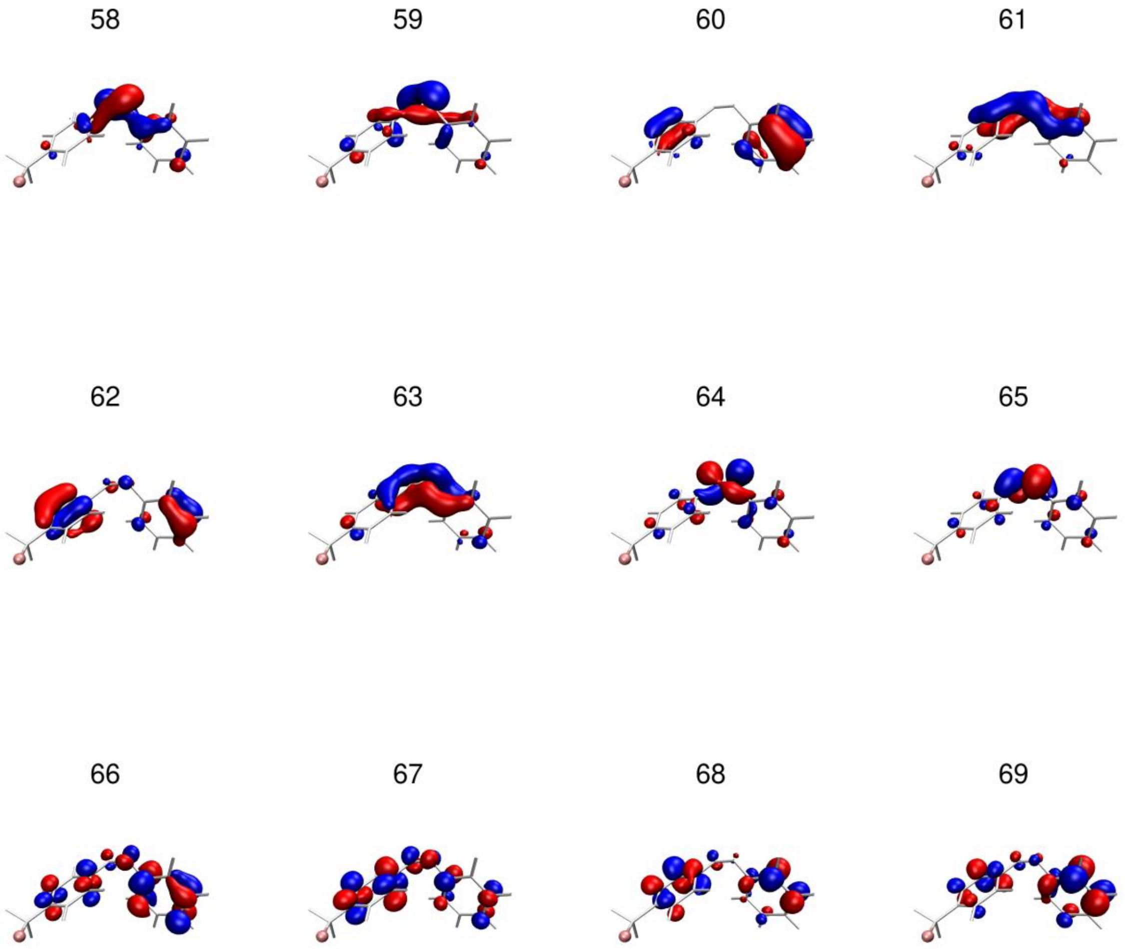

Figure S84. Active space of the MECP1 of CF_3-azobenzene at the CASSCF/ANO-RCC-VTZP//ωB97X-3c/SMD(toluene) level of theory. The digits represent the orbital number.

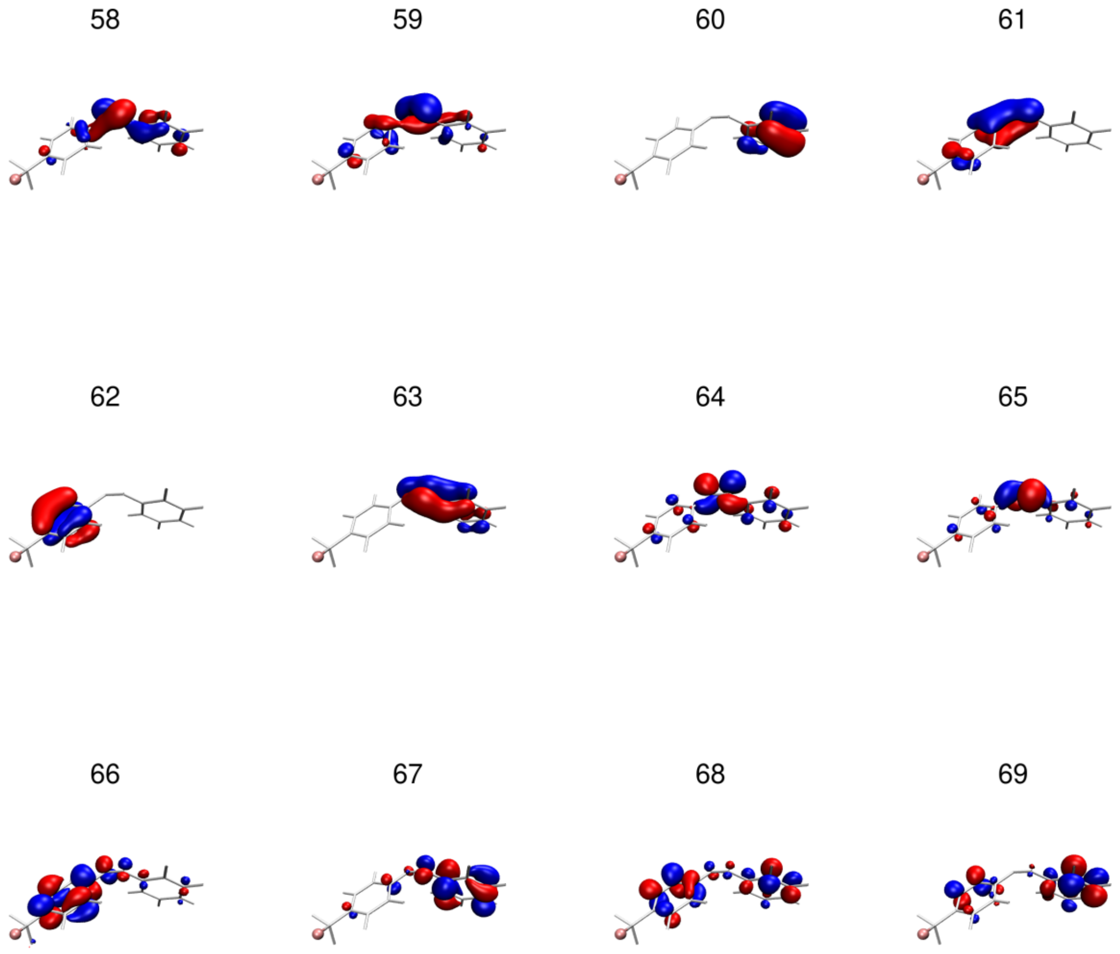

Figure S85. Active space of the MECP2 of CF_3-azobenzene at the CASSCF/ANO-RCC-VTZP//ωB97X-3c/SMD(toluene) level of theory. The digits represent the orbital number.

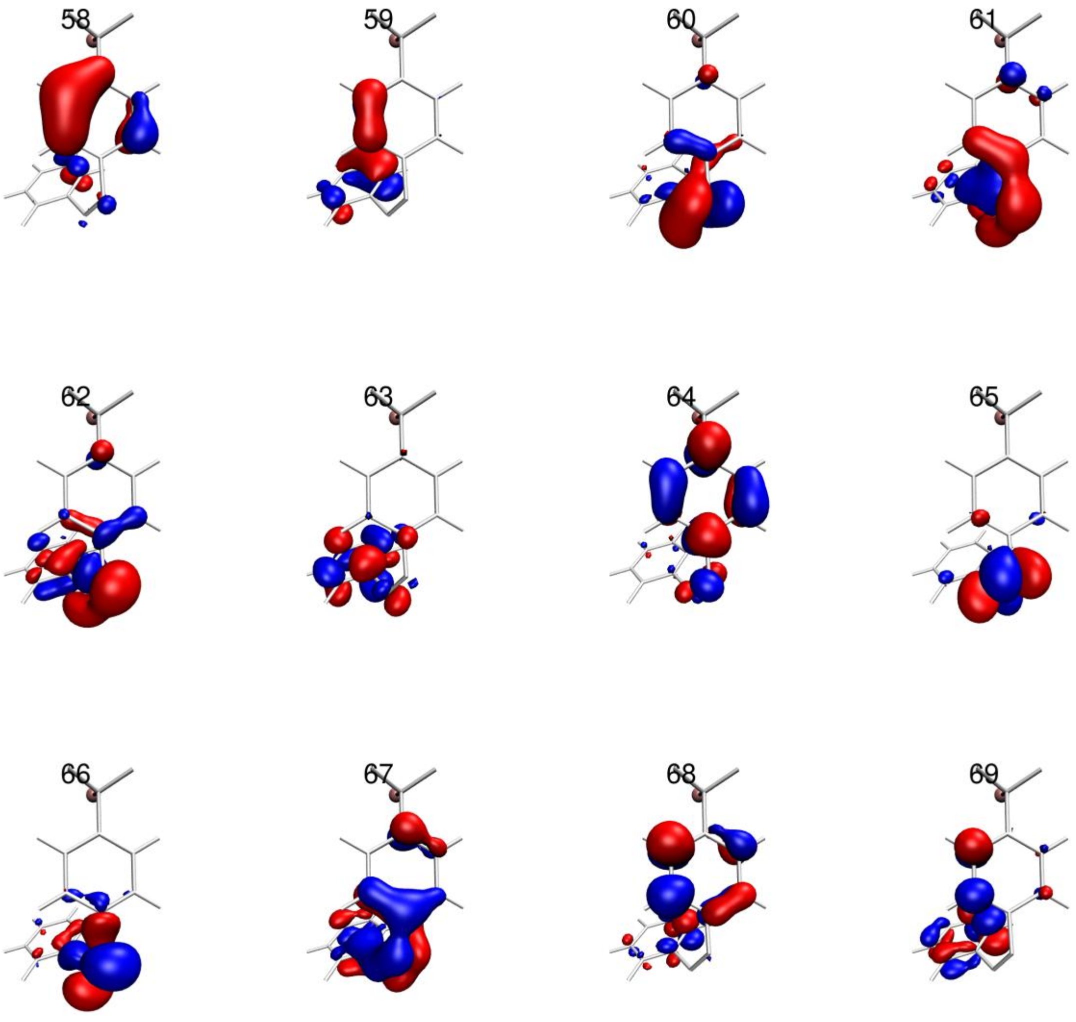

Figure S86. Active space of the Z-form of CF_3-azobenzene at the CASSCF/ANO-RCC-VTZP//ωB97X-3c/SMD(toluene) level of theory. The digits represent the orbital number.

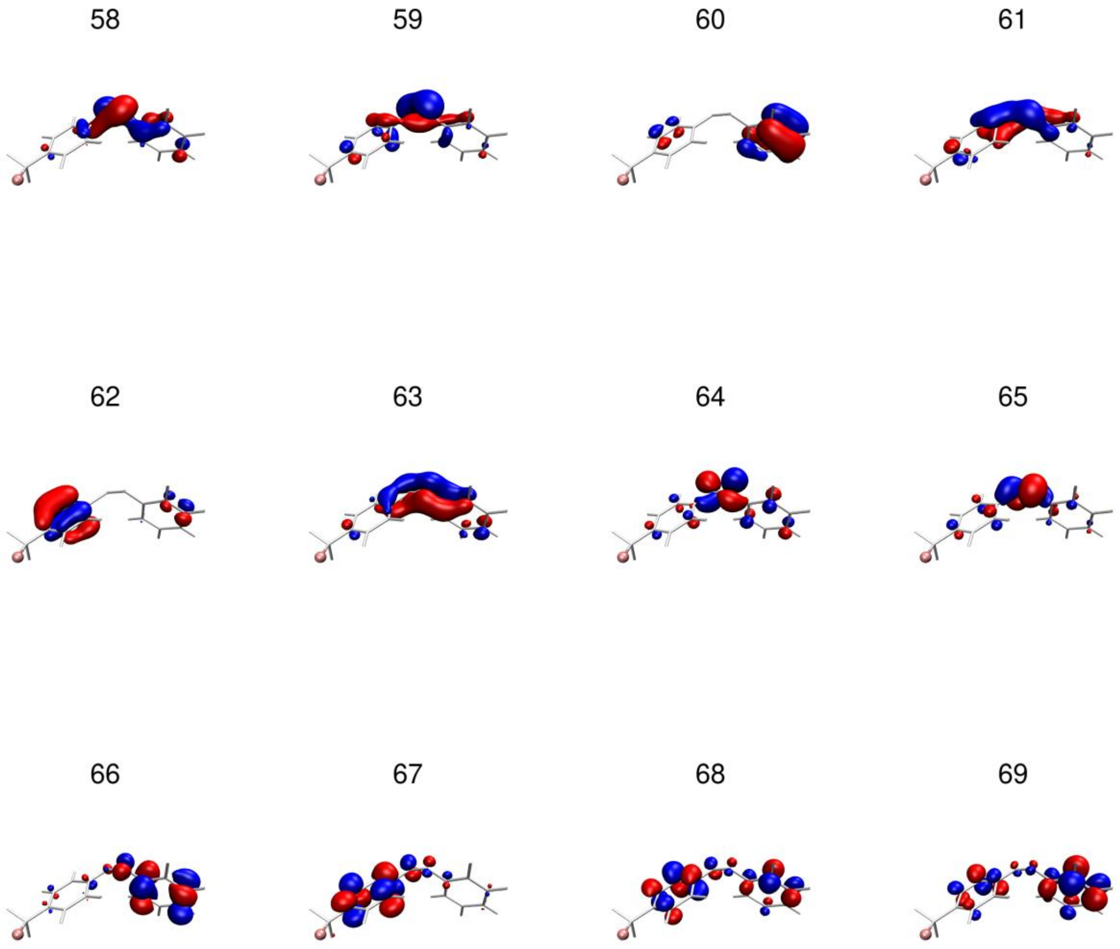

Figure S87. Active space of the T_1 minimum of CF_3-azobenzene at the CASSCF/ANO-RCC-VTZP//ωB97X-3c/SMD(toluene) level of theory. The digits represent the orbital number.

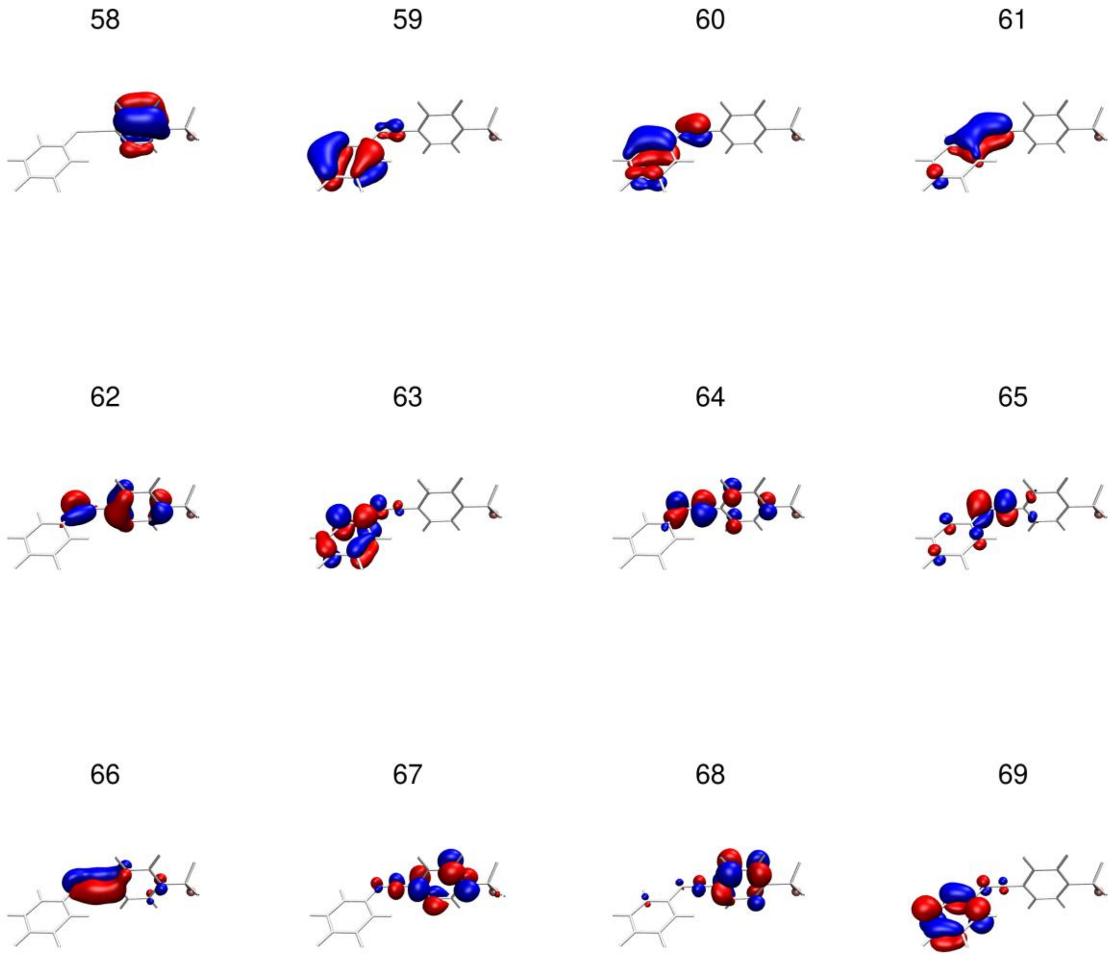

Figure S88. Active space of the TSInv1 of CF_3-azobenzene at the CASSCF/ANO-RCC-VTZP//ωB97X-3c/SMD(toluene) level of theory. The digits represent the orbital number.

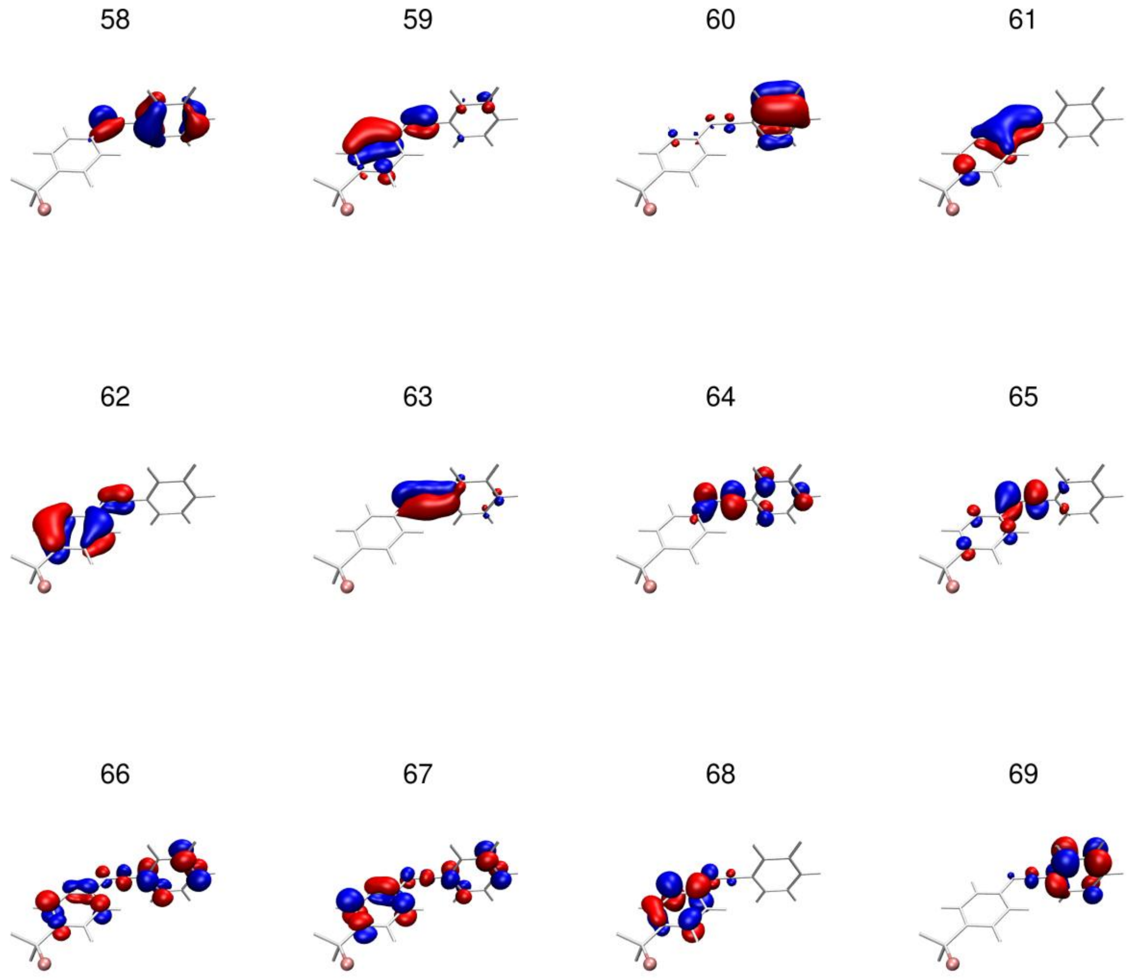

Figure S89. Active space of the TSInv2 of CF_3-azobenzene at the CASSCF/ANO-RCC-VTZP//ωB97X-3c/SMD(toluene) level of theory. The digits represent the orbital number.

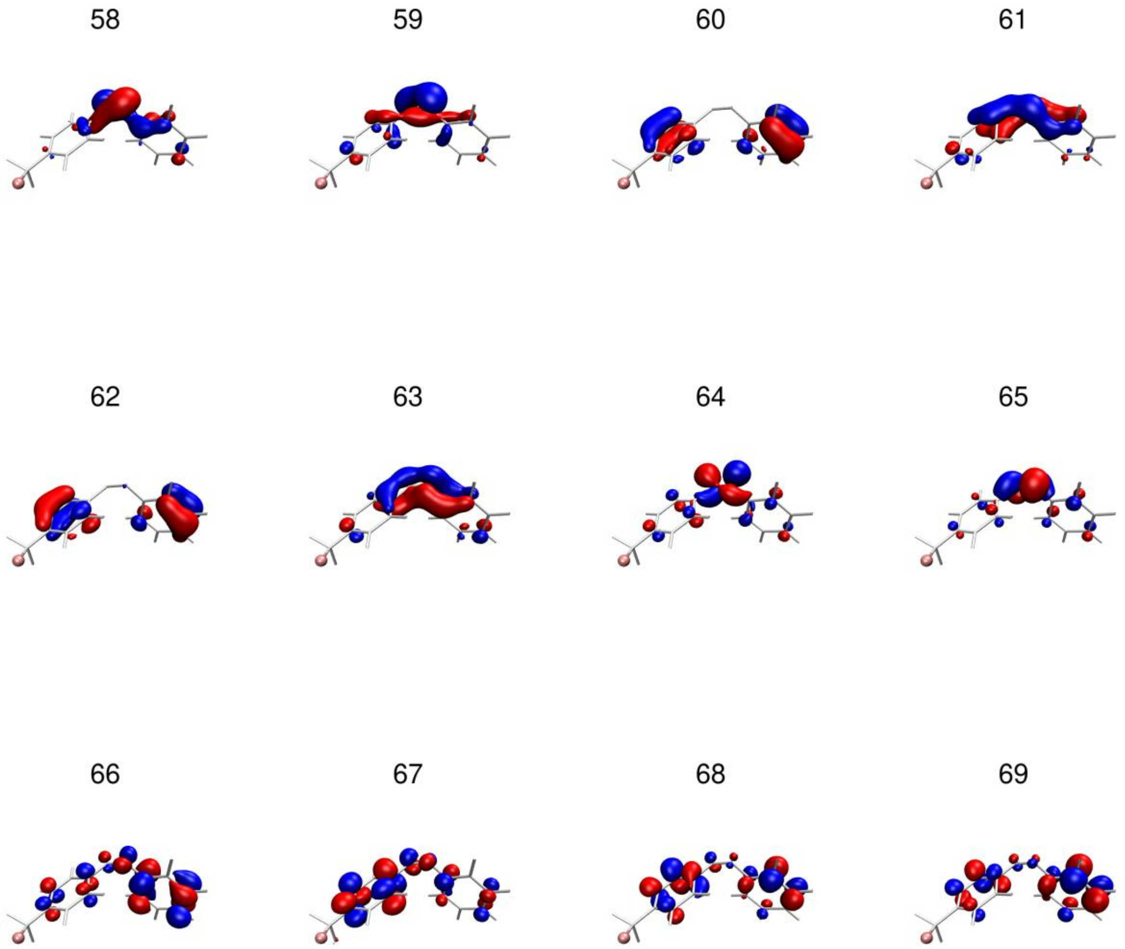

Figure S90. Active space of the TSRot of CF_3-azobenzene at the CASSCF/ANO-RCC-VTZP//ωB97X-3c/SMD(toluene) level of theory. The digits represent the orbital number.

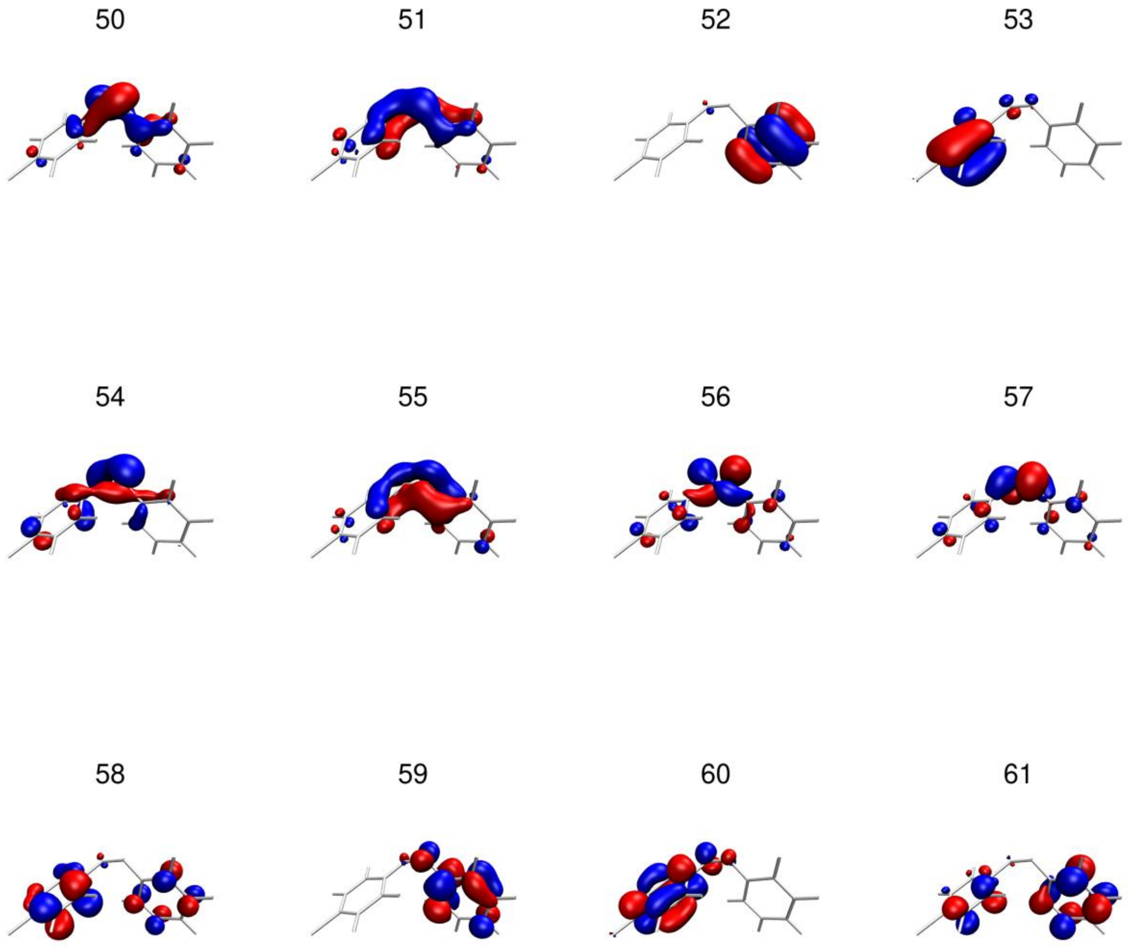

Figure S91. Active space of the MECP1 of chloroazobenzene at the CASSCF/ANO-RCC-VTZP//ωB97X-3c/SMD(toluene) level of theory. The digits represent the orbital number.

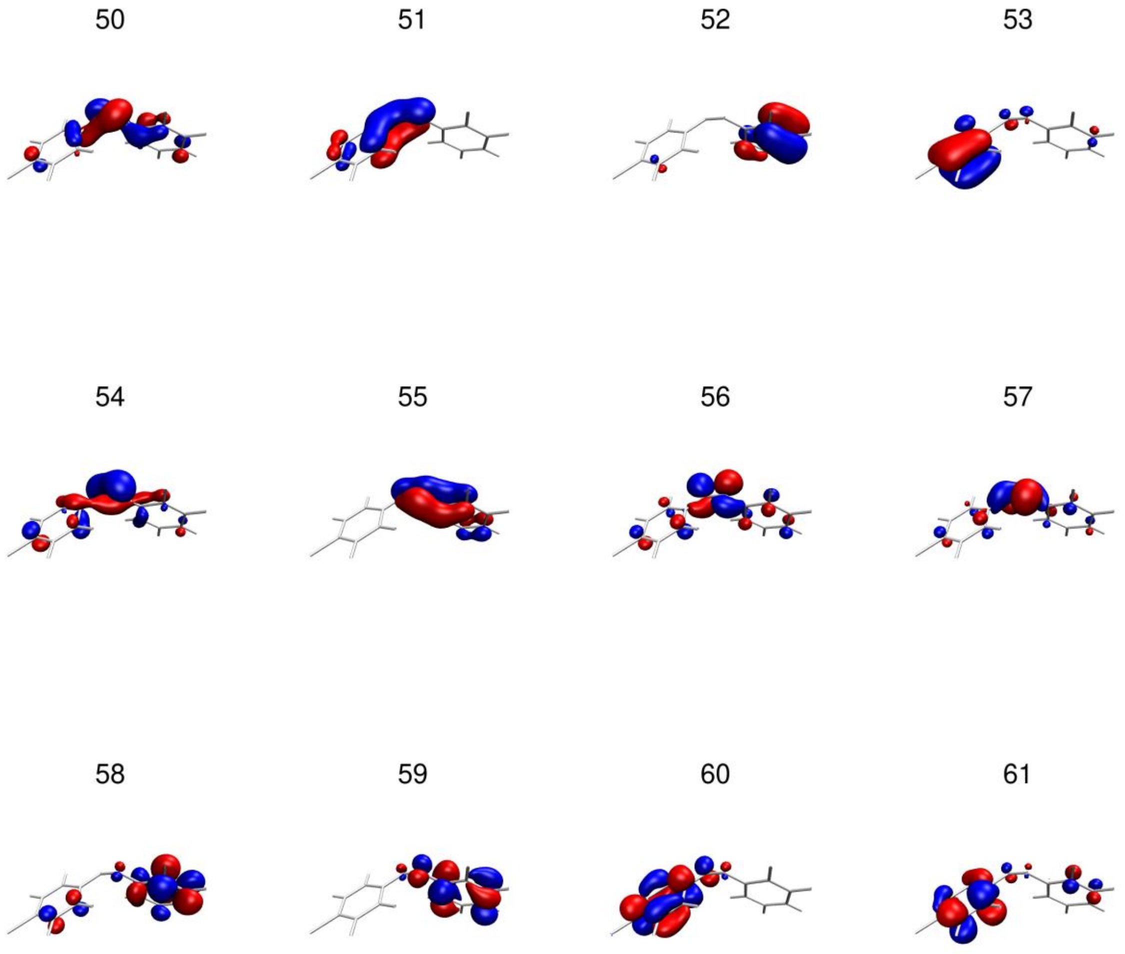

Figure S92. Active space of the MECP2 of chloroazobenzene at the CASSCF/ANO-RCC-VTZP//ωB97X-3c/SMD(toluene) level of theory. The digits represent the orbital number.

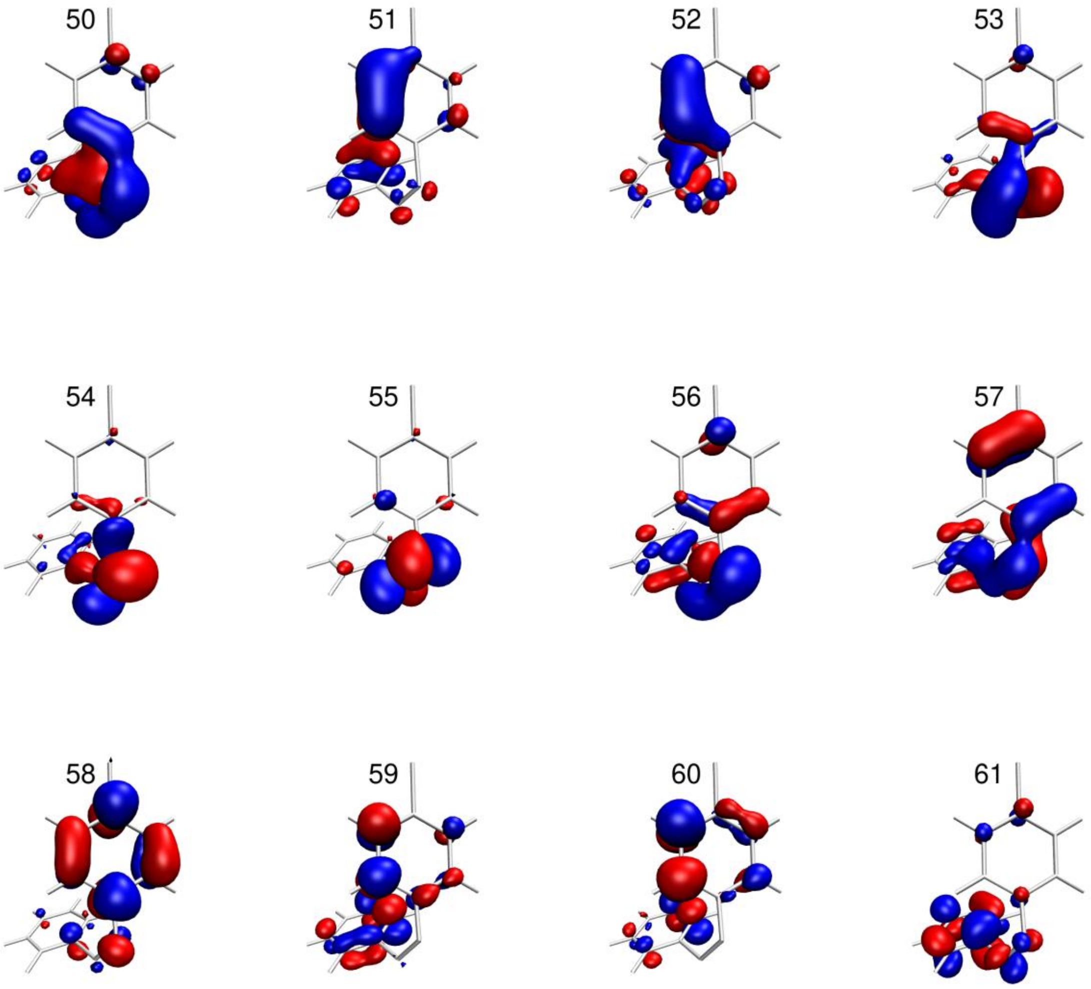

Figure S93. Active space of the Z-form of chloroazobenzene at the CASSCF/ANO-RCC-VTZP//ωB97X-3c/SMD(toluene) level of theory. The digits represent the orbital number.

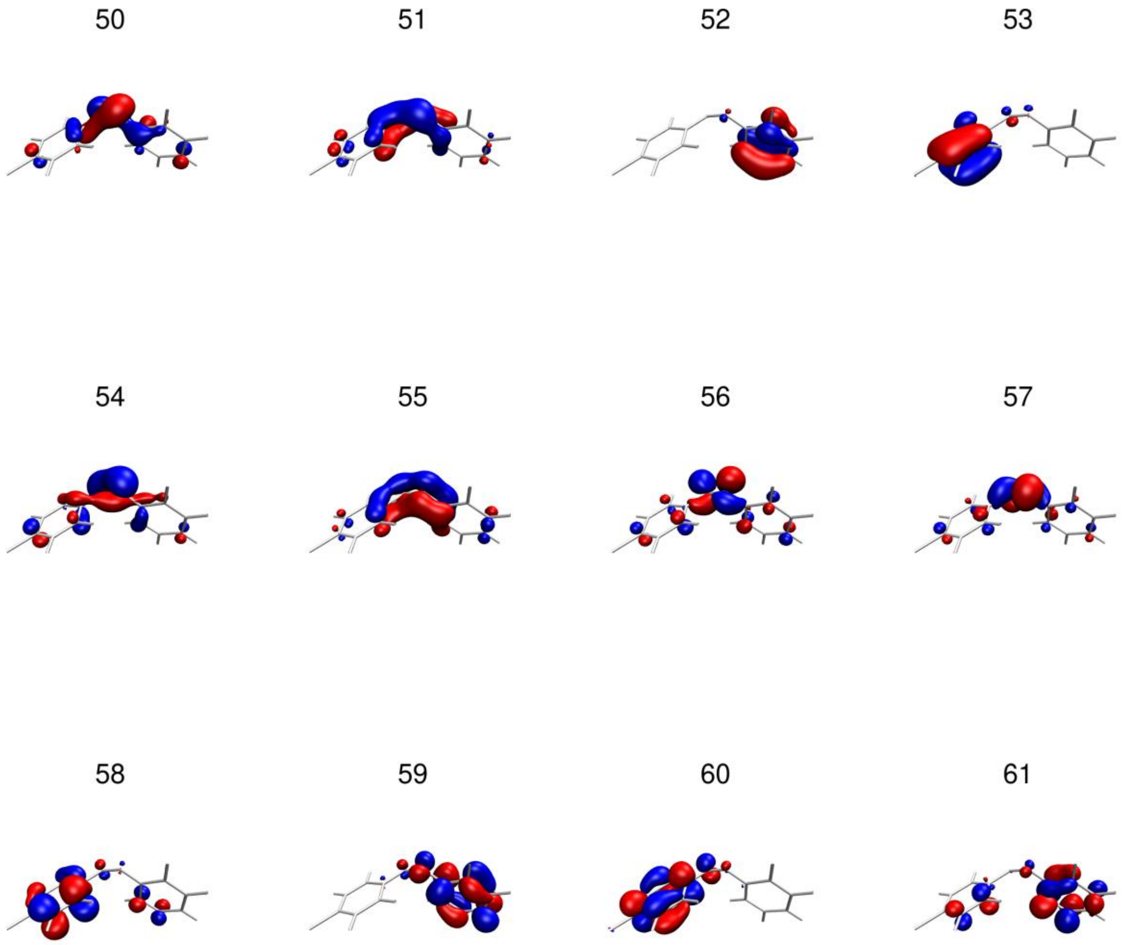

Figure S94. Active space of the T_1 minimum of chloroazobenzene at the CASSCF/ANO-RCC-VTZP//ωB97X-3c/SMD(toluene) level of theory. The digits represent the orbital number.

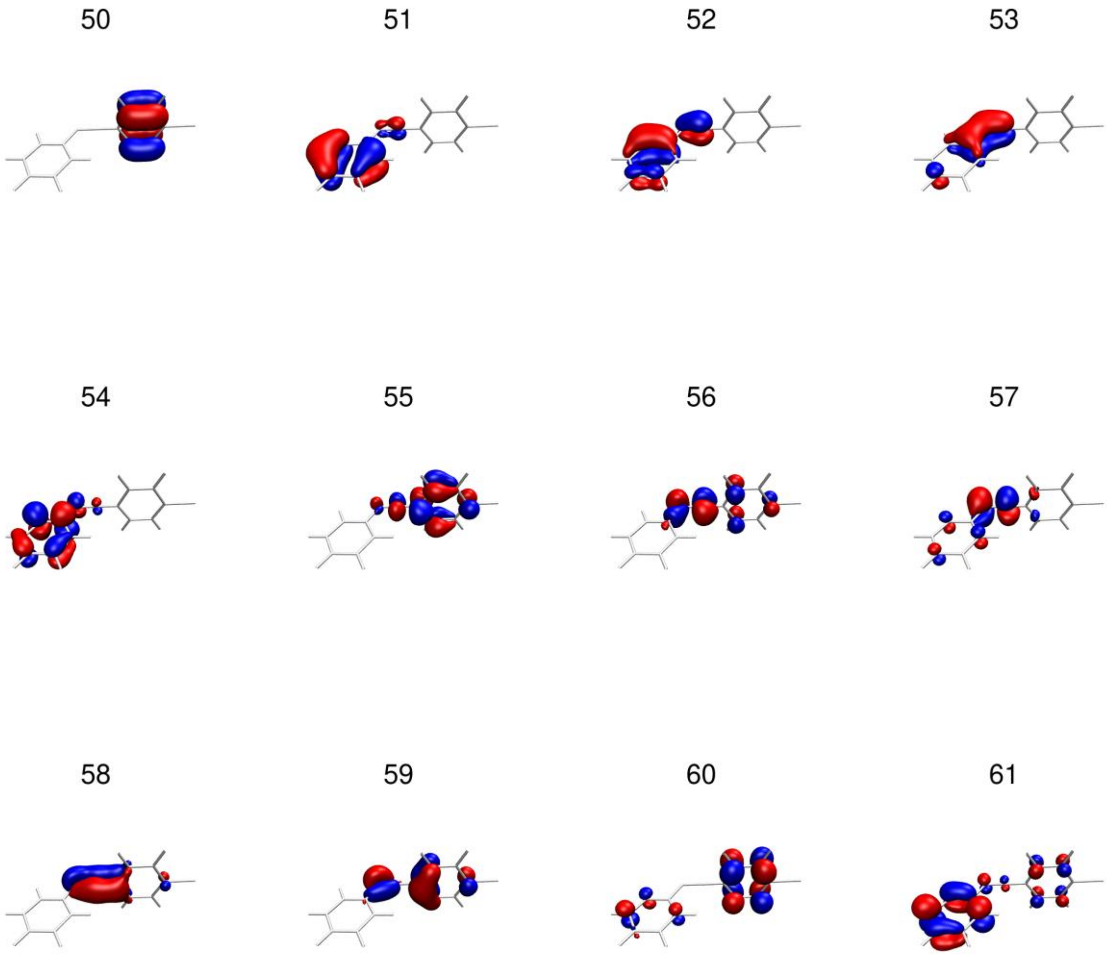

Figure S95. Active space of the TSInv1 of chloroazobenzene at the CASSCF/ANO-RCC-VTZP//ωB97X-3c/SMD(toluene) level of theory. The digits represent the orbital number.

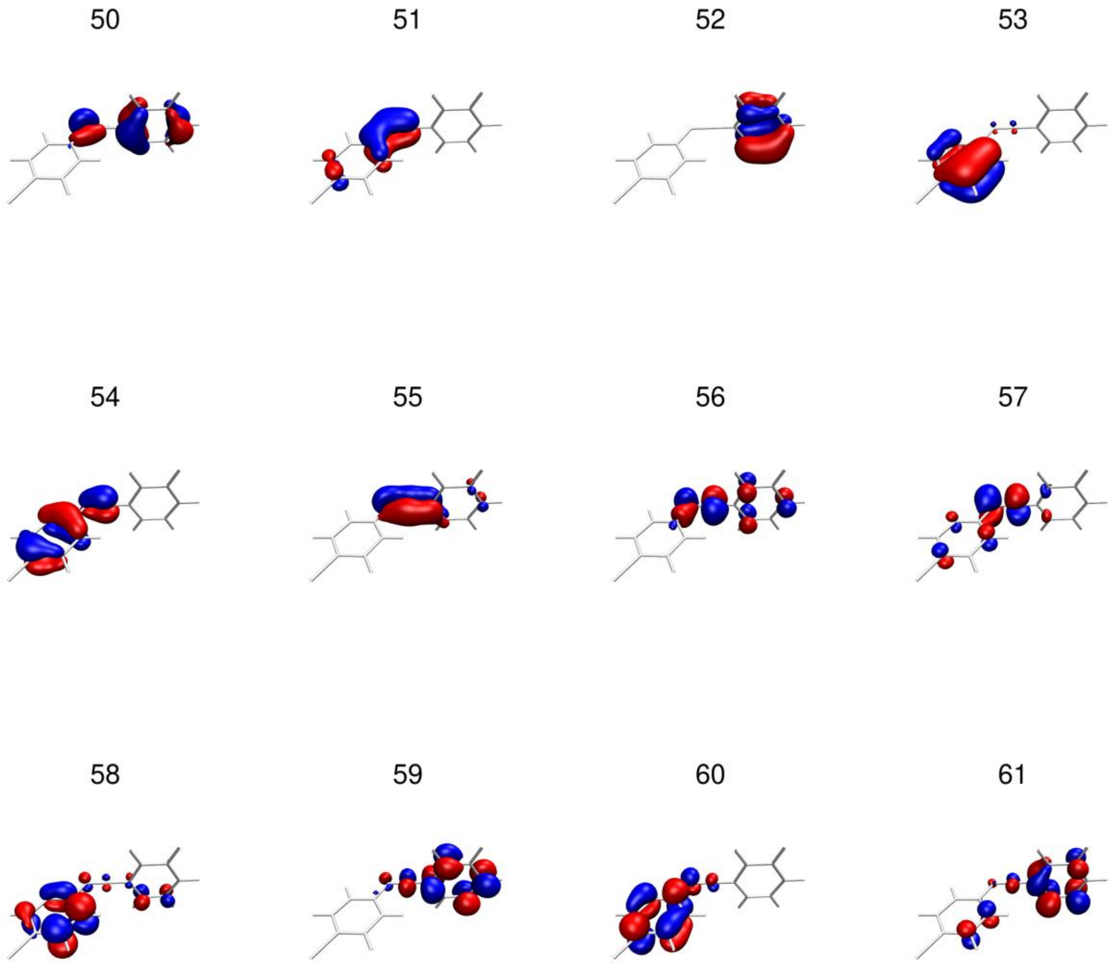

Figure S96. Active space of the TSInv2 of chloroazobenzene at the CASSCF/ANO-RCC-VTZP//ωB97X-3c/SMD(toluene) level of theory. The digits represent the orbital number.

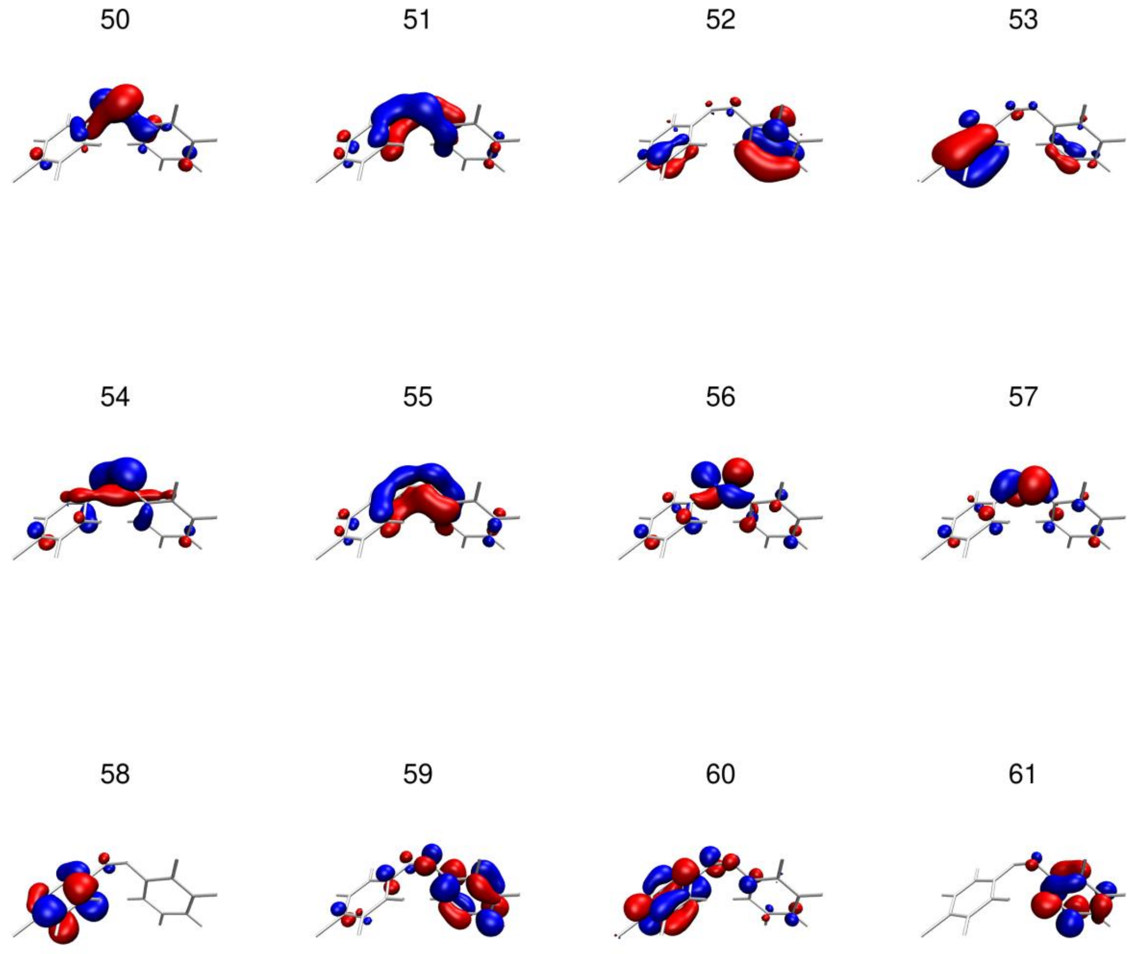

Figure S97. Active space of the TSRot of chloroazobenzene at the CASSCF/ANO-RCC-VTZP//ωB97X-3c/SMD(toluene) level of theory. The digits represent the orbital number.

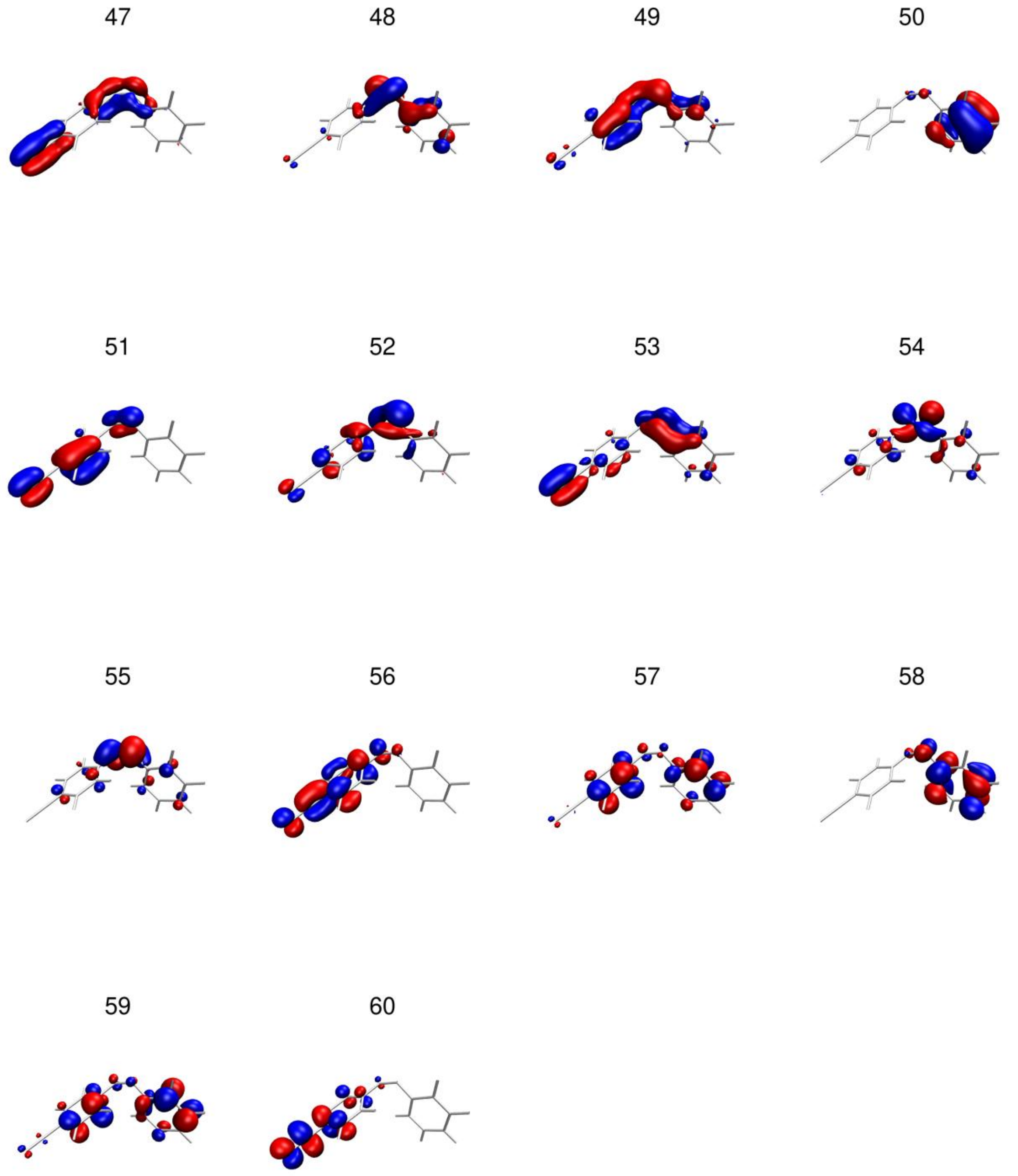

Figure S98. Active space of the MECP1 of CN-azobenzene at the CASSCF/ANO-RCC-VTZP//ωB97X-3c/SMD(toluene) level of theory. The digits represent the orbital number.

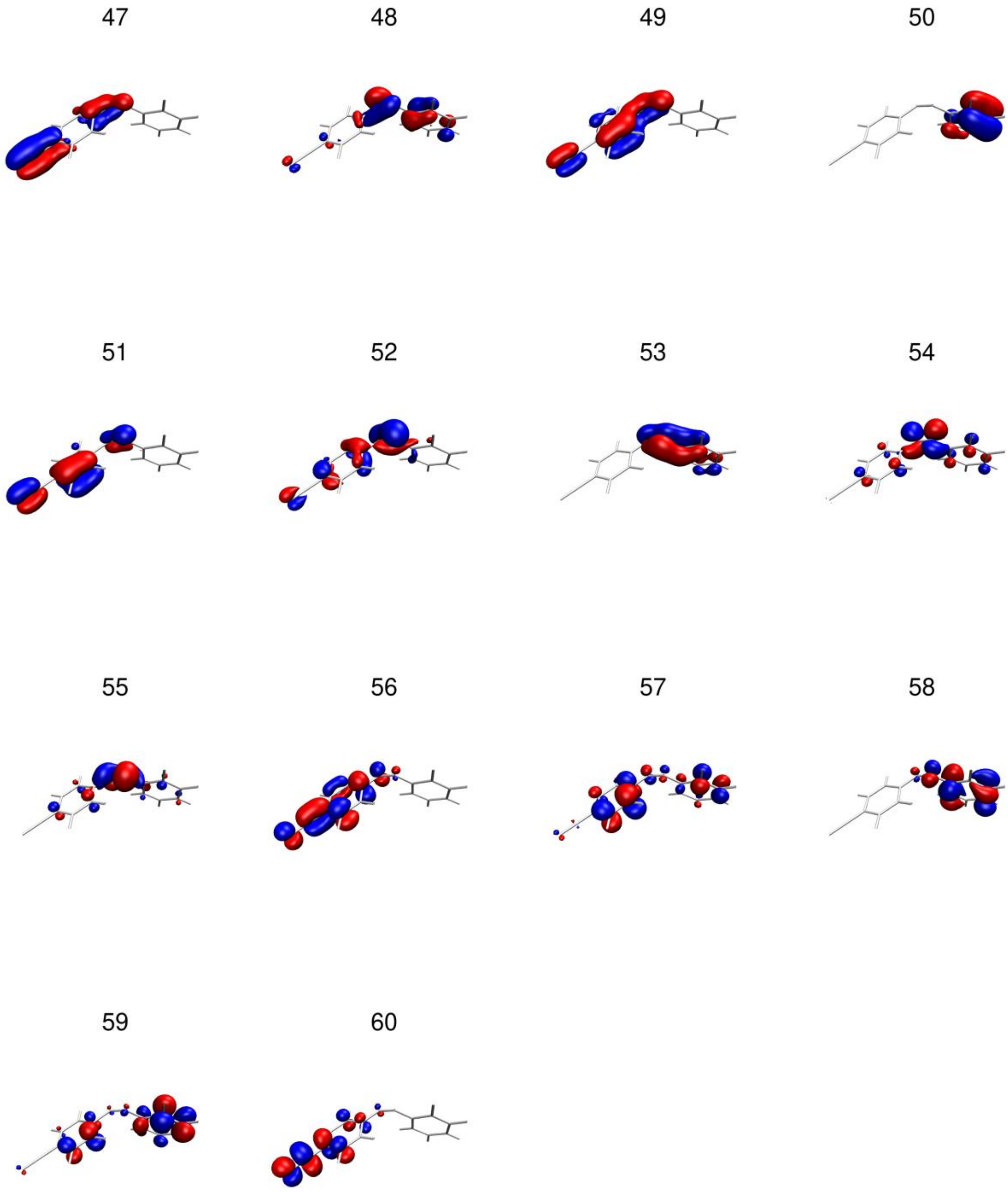

Figure S99. Active space of the MECP2 of CN-azobenzene at the CASSCF/ANO-RCC-VTZP//ωB97X-3c/SMD(toluene) level of theory. The digits represent the orbital number.

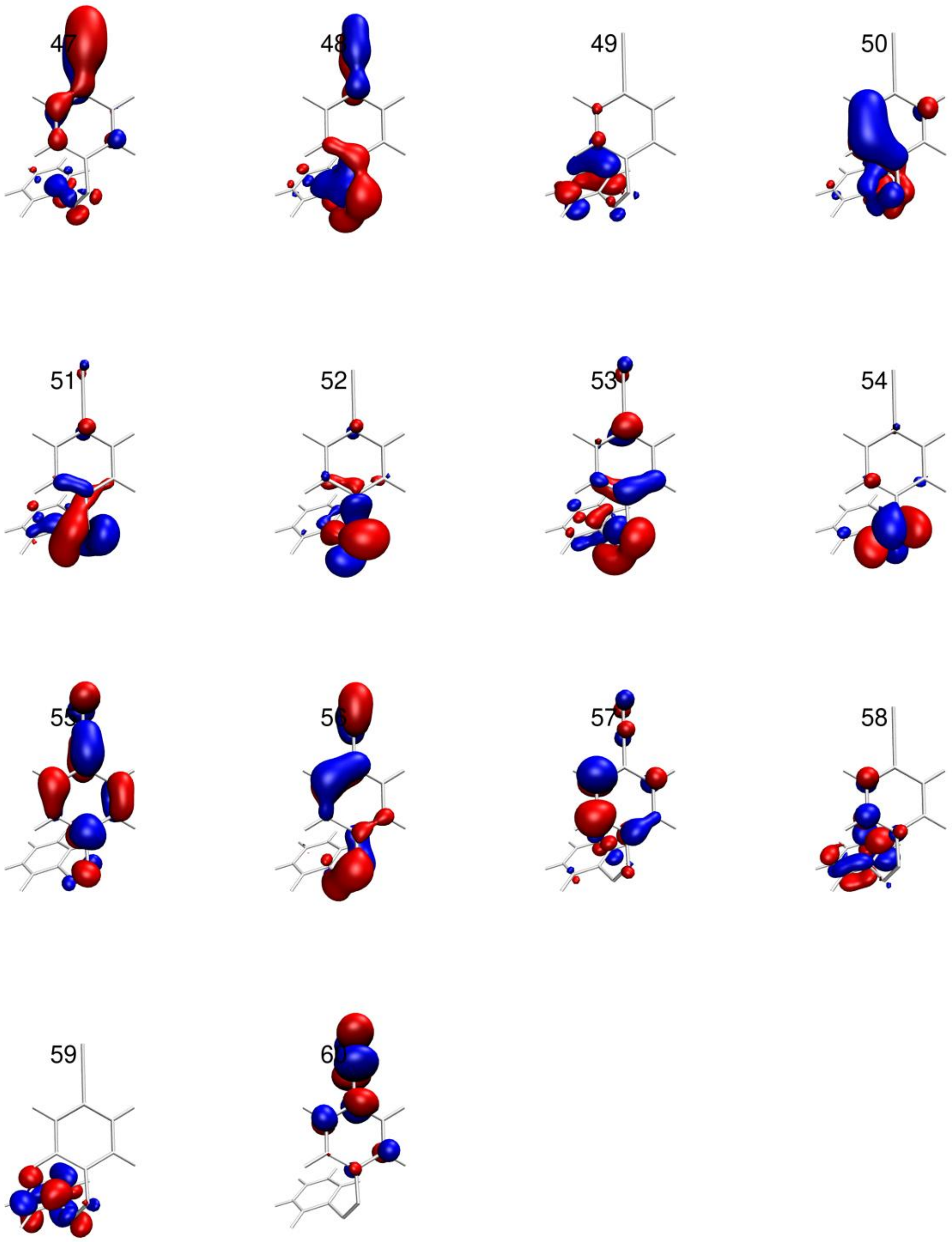

Figure S100. Active space of the Z-form of CN-azobenzene at the CASSCF/ANO-RCC-VTZP//ωB97X-3c/SMD(toluene) level of theory. The digits represent the orbital number.

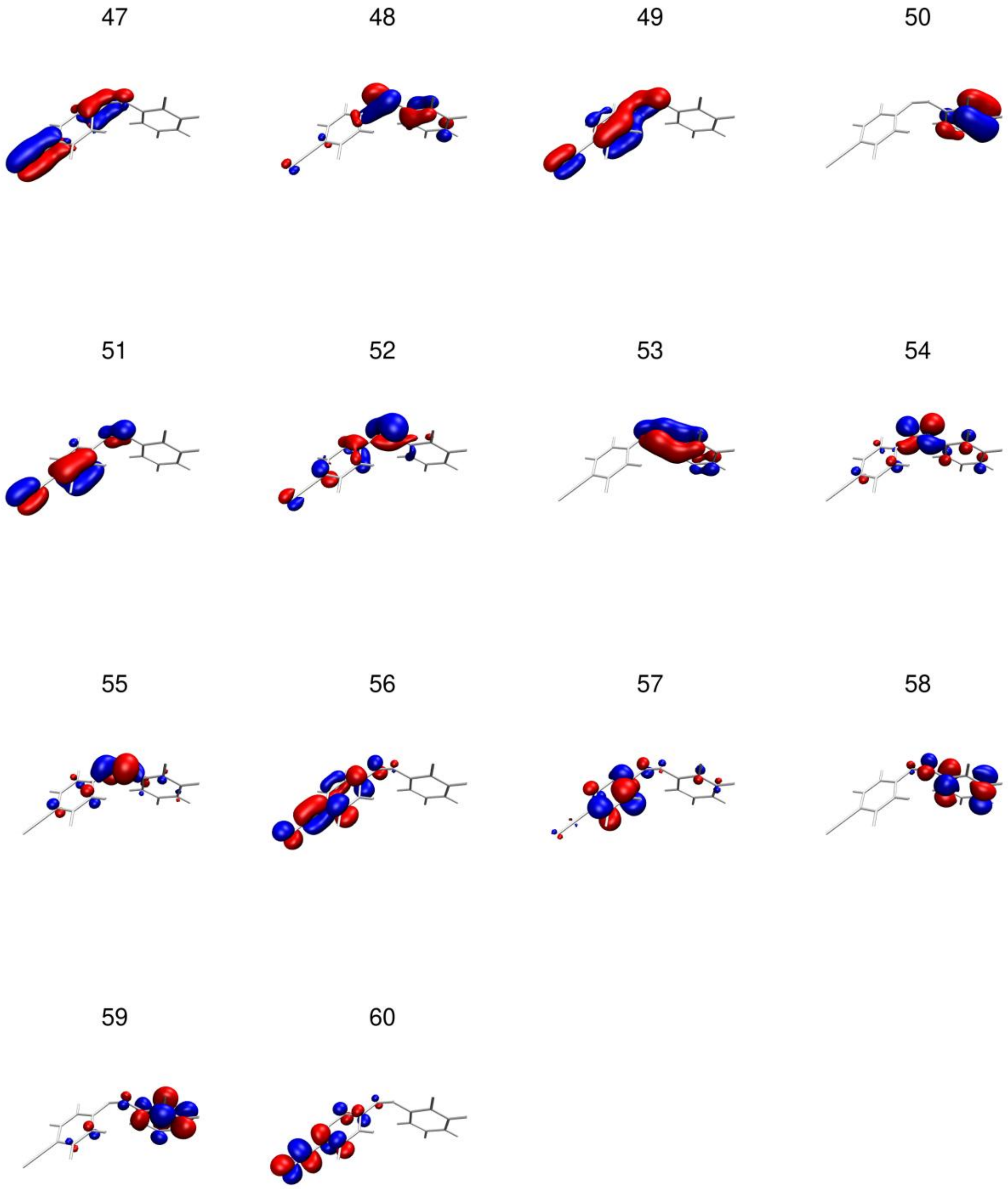

Figure S101. Active space of the T_1 minimum of CN-azobenzene at the CASSCF/ANO-RCC-VTZP//ωB97X-3c/SMD(toluene) level of theory. The digits represent the orbital number.

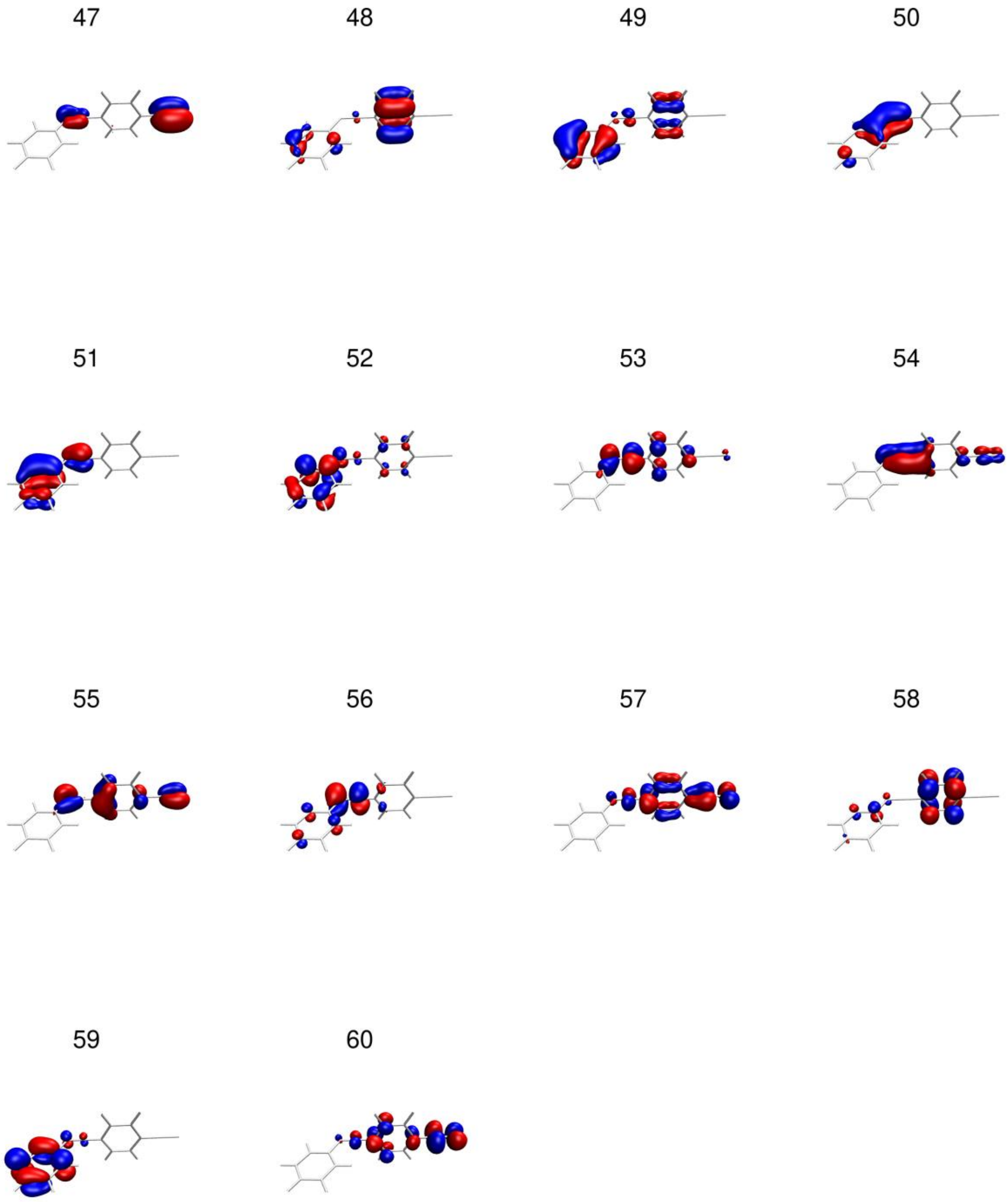

Figure S102. Active space of the TSInv1 of CN-azobenzene at the CASSCF/ANO-RCC-VTZP//ωB97X-3c/SMD(toluene) level of theory. The digits represent the orbital number.

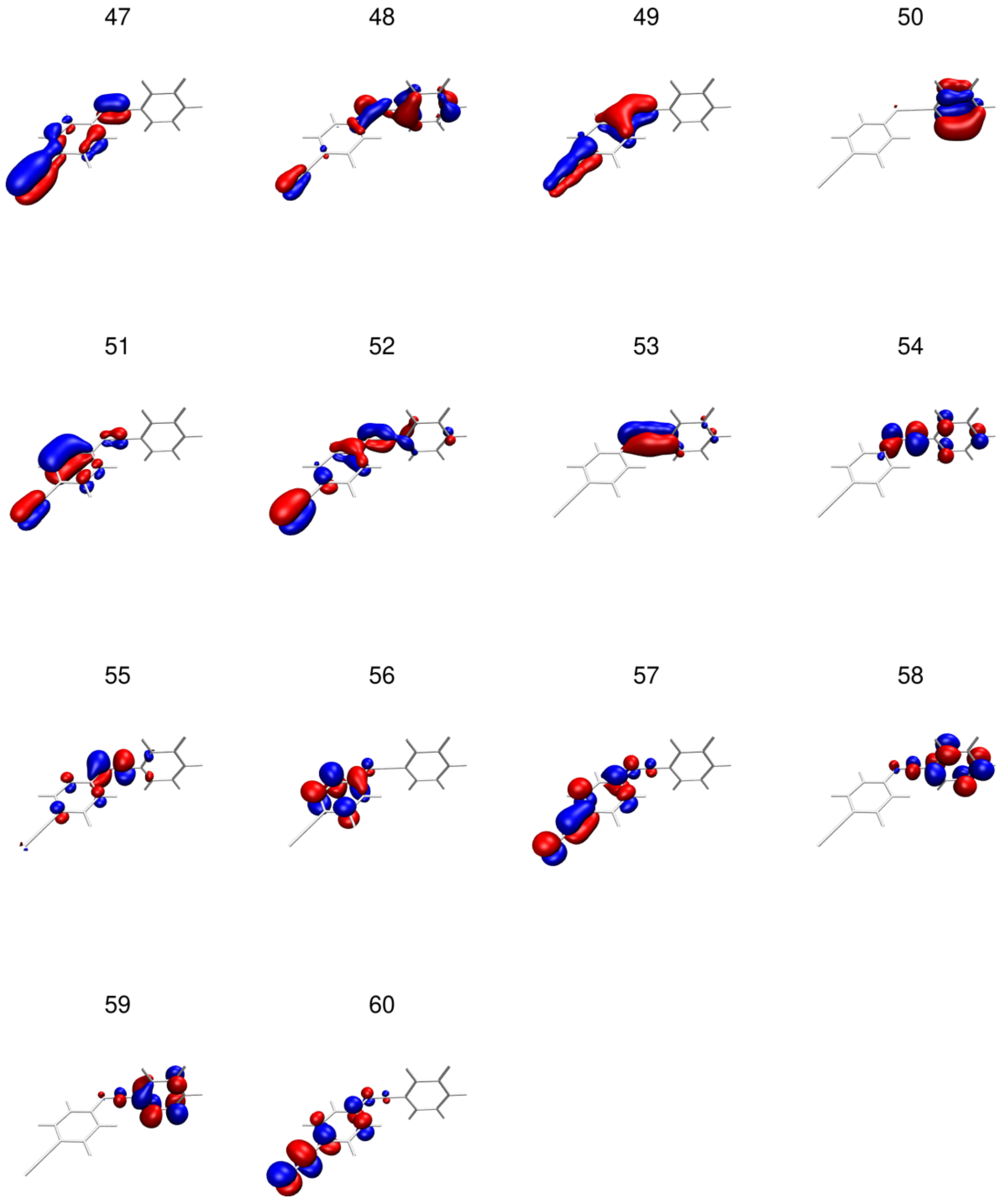

Figure S103. Active space of the TSInv2 of CN-azobenzene at the CASSCF/ANO-RCC-VTZP//ωB97X-3c/SMD(toluene) level of theory. The digits represent the orbital number.

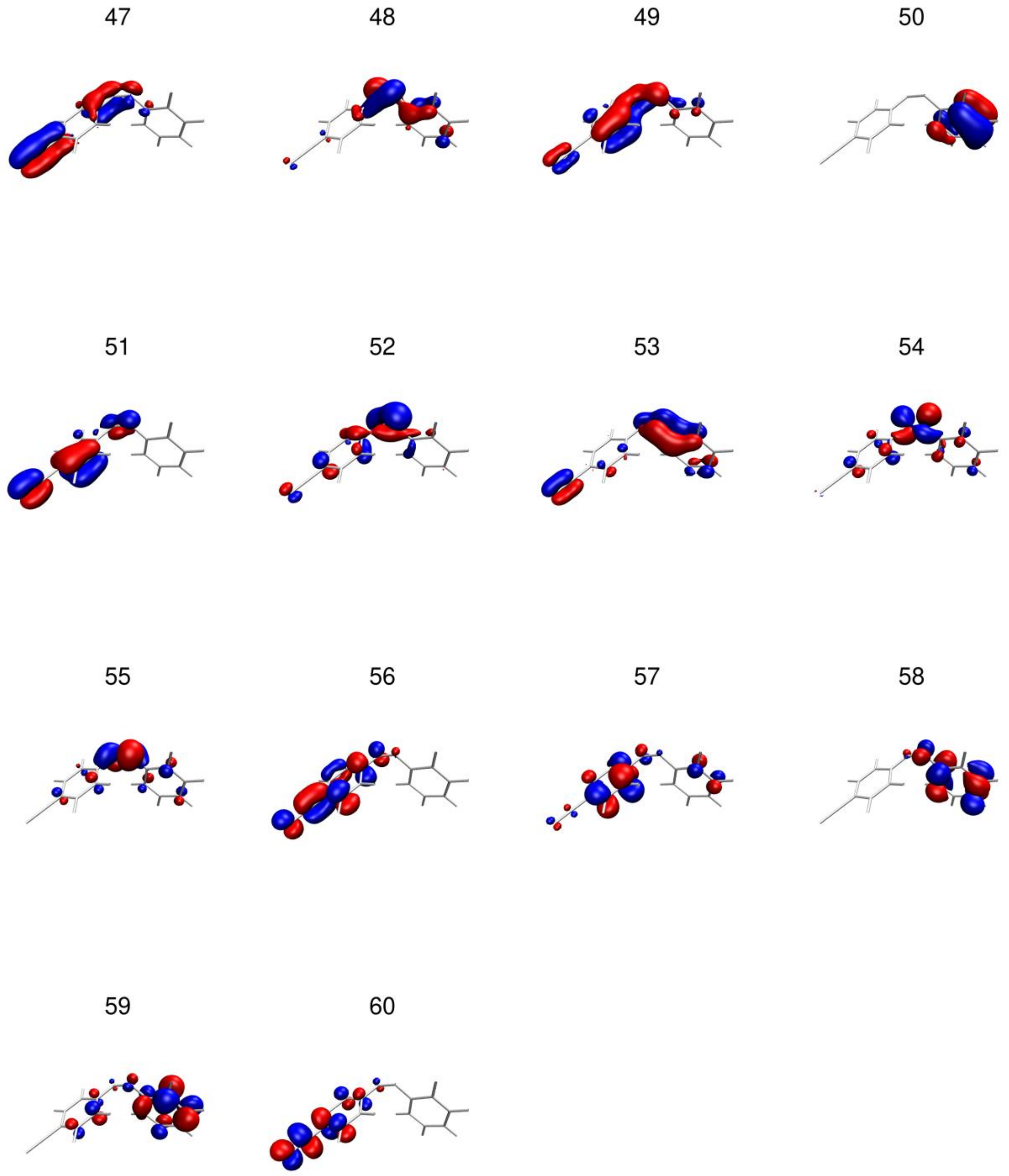

Figure S104. Active space of the TSRot of CN-azobenzene at the CASSCF/ANO-RCC-VTZP//ωB97X-3c/SMD(toluene) level of theory. The digits represent the orbital number.

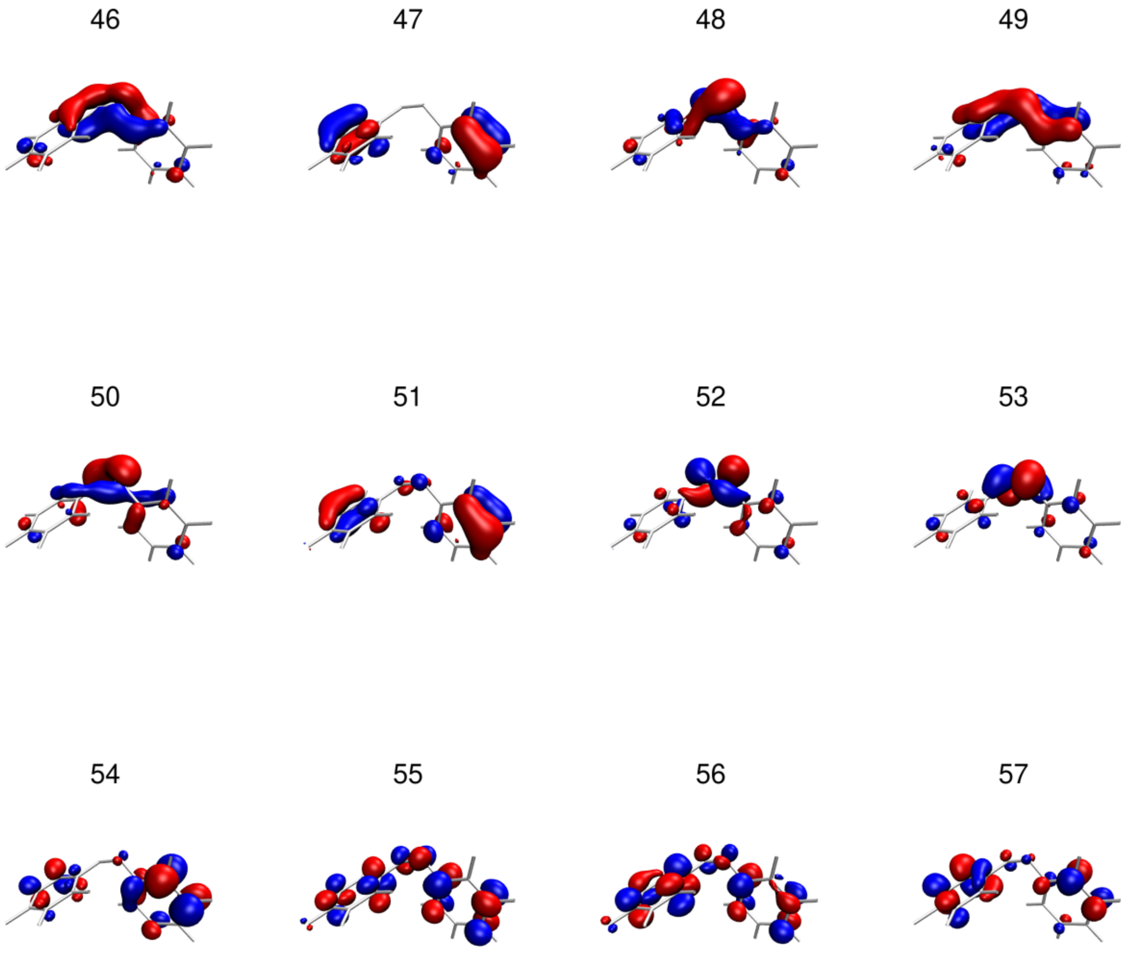

Figure S105. Active space of the MECP1 of fluoroazobenzene at the CASSCF/ANO-RCC-VTZP//ωB97X-3c/SMD(toluene) level of theory. The digits represent the orbital number.

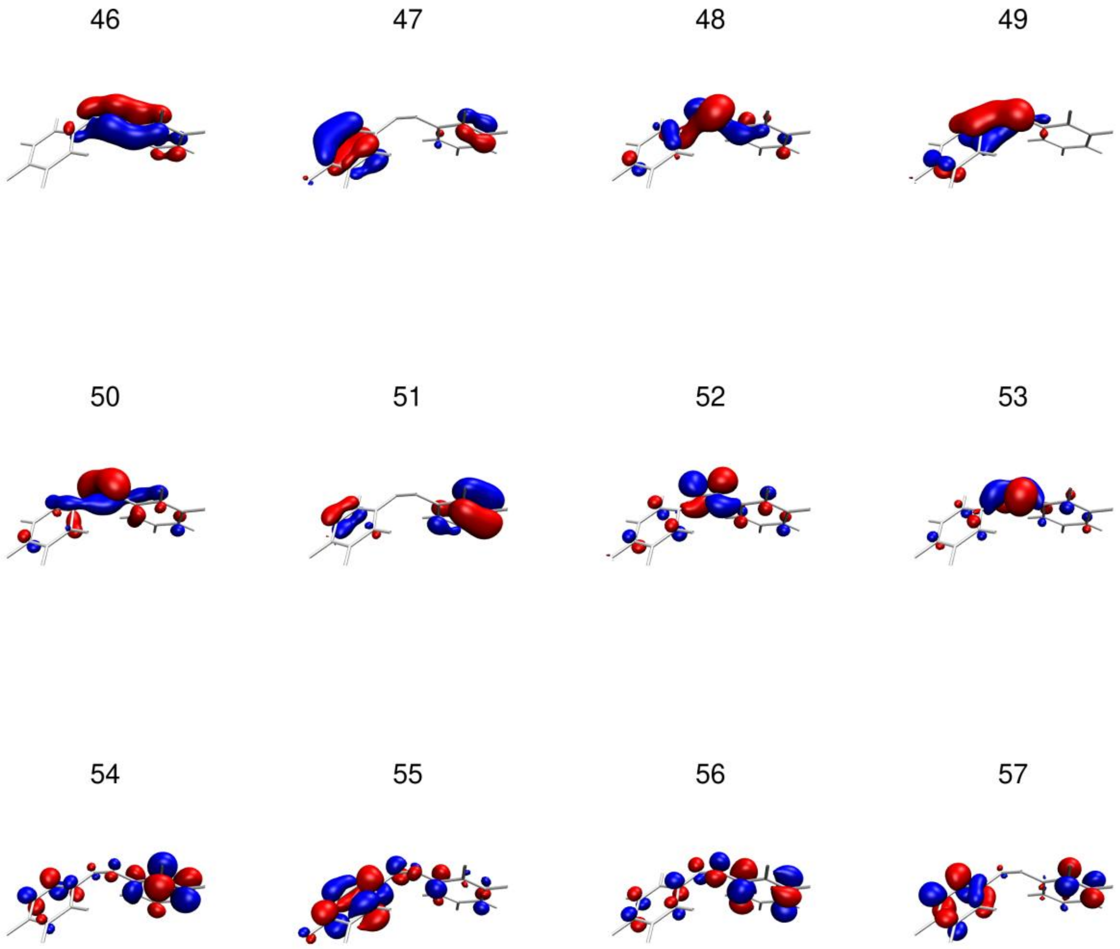

Figure S106. Active space of the MECP2 of fluoroazobenzene at the CASSCF/ANO-RCC-VTZP//ωB97X-3c/SMD(toluene) level of theory. The digits represent the orbital number.

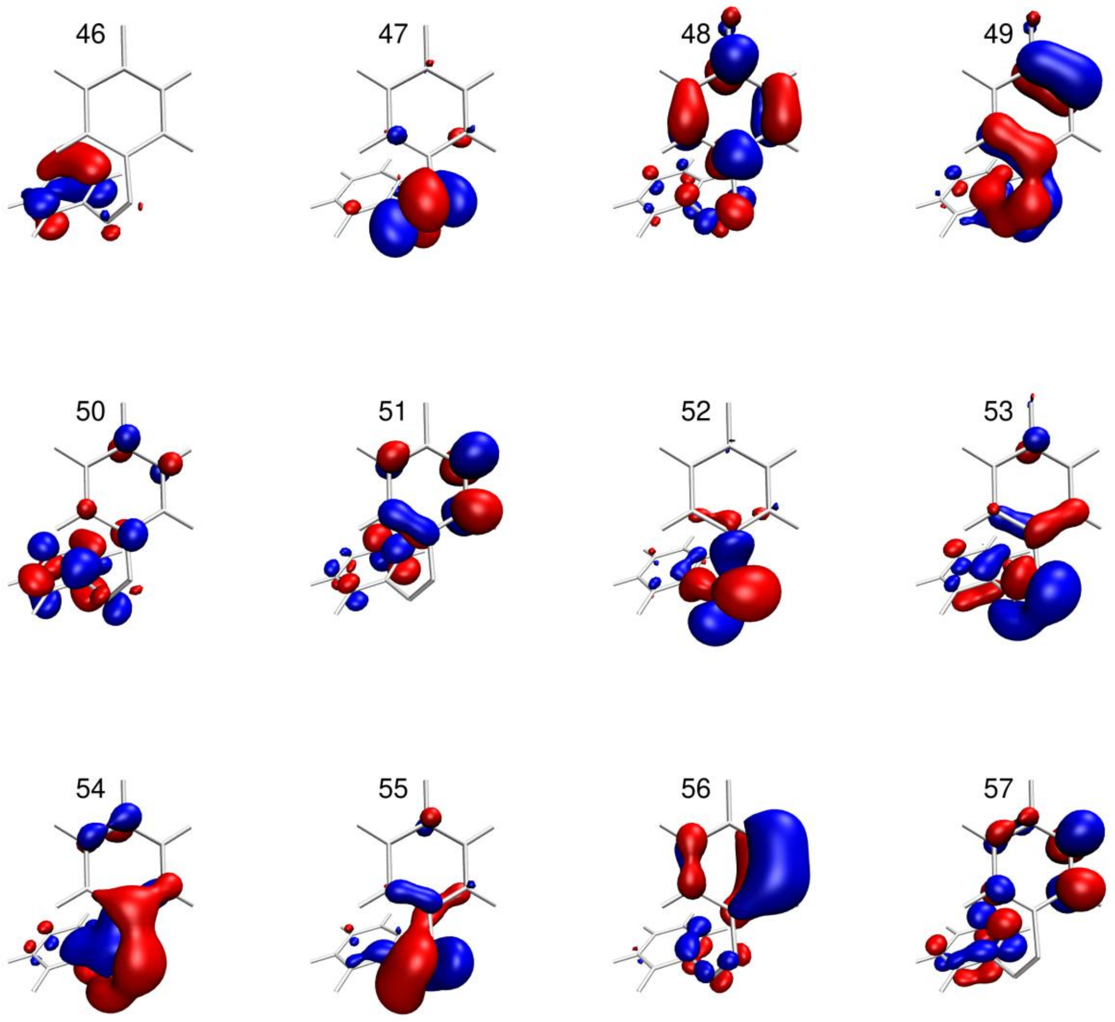

Figure S107. Active space of the Z-form of fluoroazobenzene at the CASSCF/ANO-RCC-VTZP//ωB97X-3c/SMD(toluene) level of theory. The digits represent the orbital number.

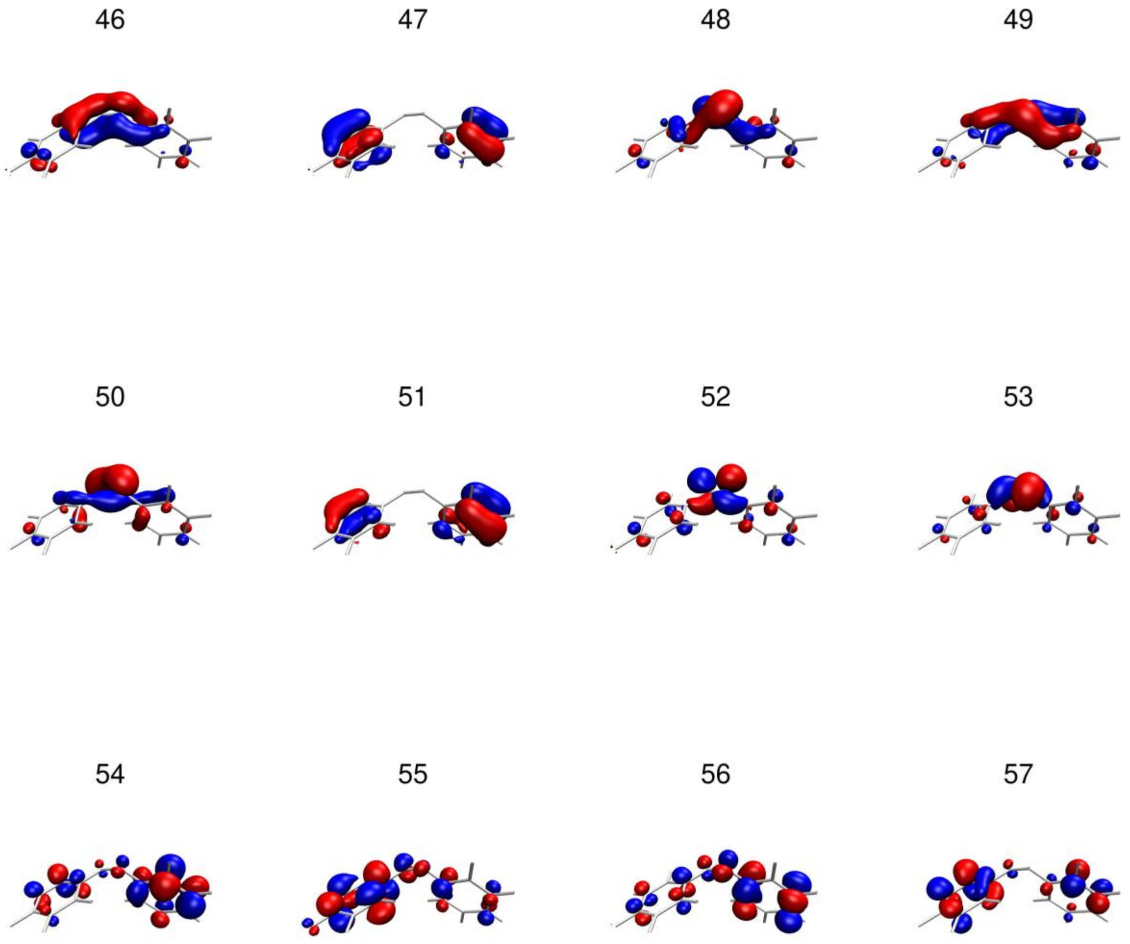

Figure S108. Active space of the T_1 minimum of fluoroazobenzene at the CASSCF/ANO-RCC-VTZP//ωB97X-3c/SMD(toluene) level of theory. The digits represent the orbital number.

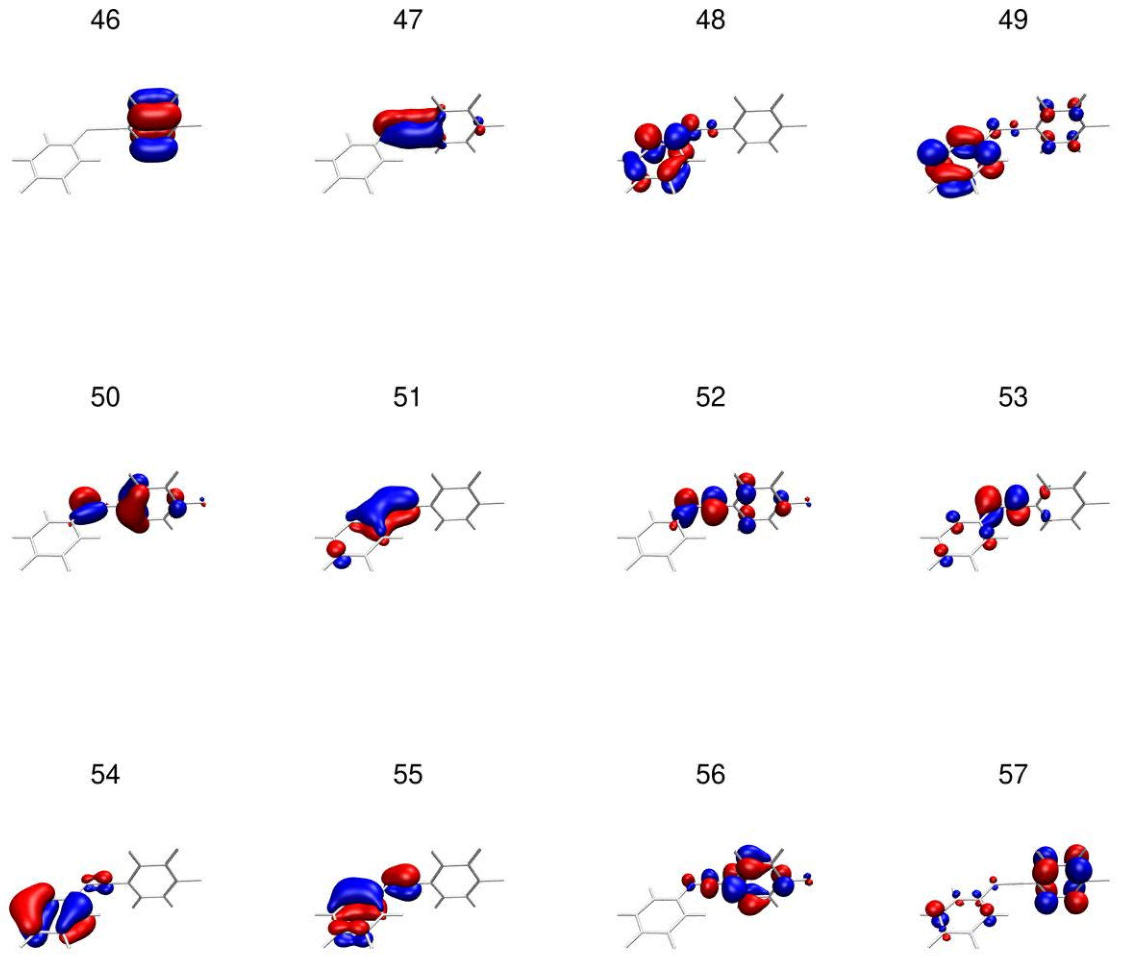

Figure S109. Active space of the TSInv1 of fluoroazobenzene at the CASSCF/ANO-RCC-VTZP//ωB97X-3c/SMD(toluene) level of theory. The digits represent the orbital number.

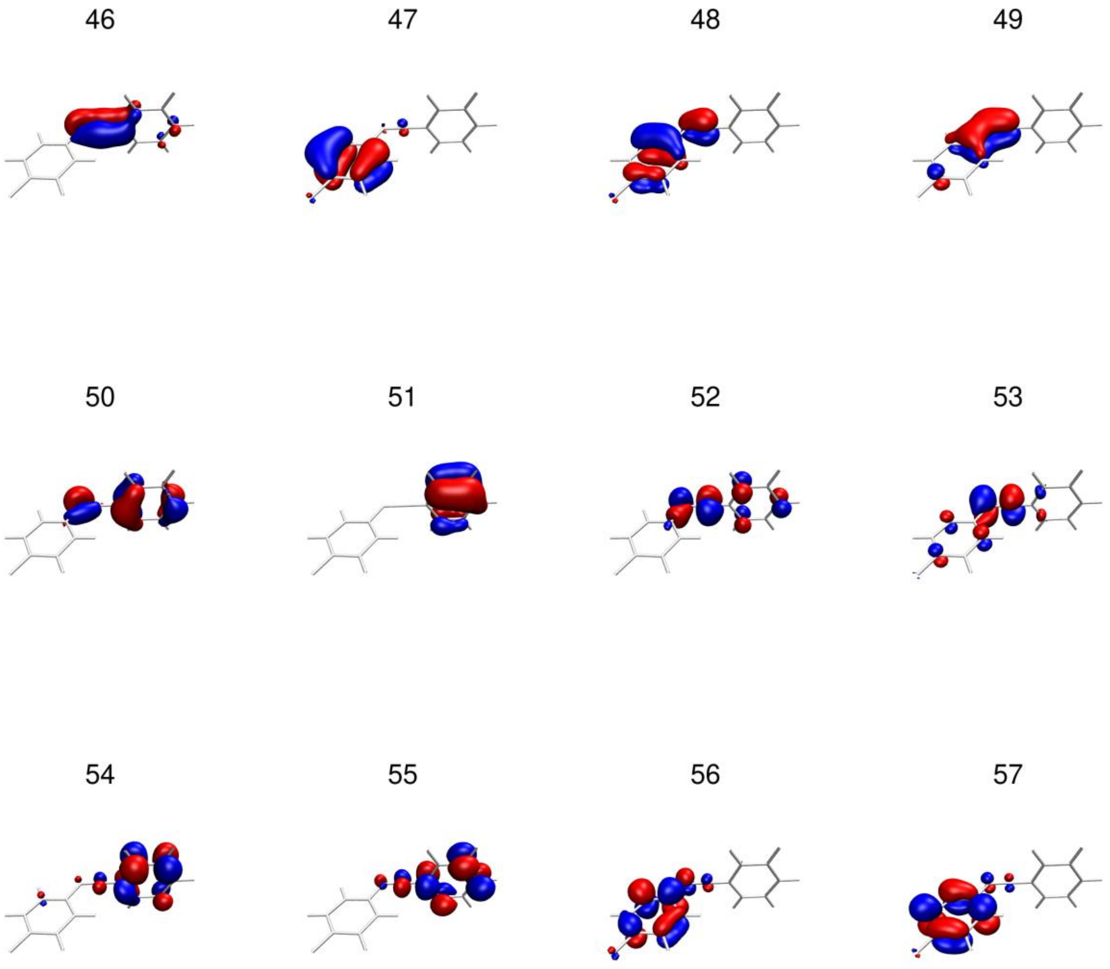

Figure S110. Active space of the TSInv2 of fluoroazobenzene at the CASSCF/ANO-RCC-VTZP//ωB97X-3c/SMD(toluene) level of theory. The digits represent the orbital number.

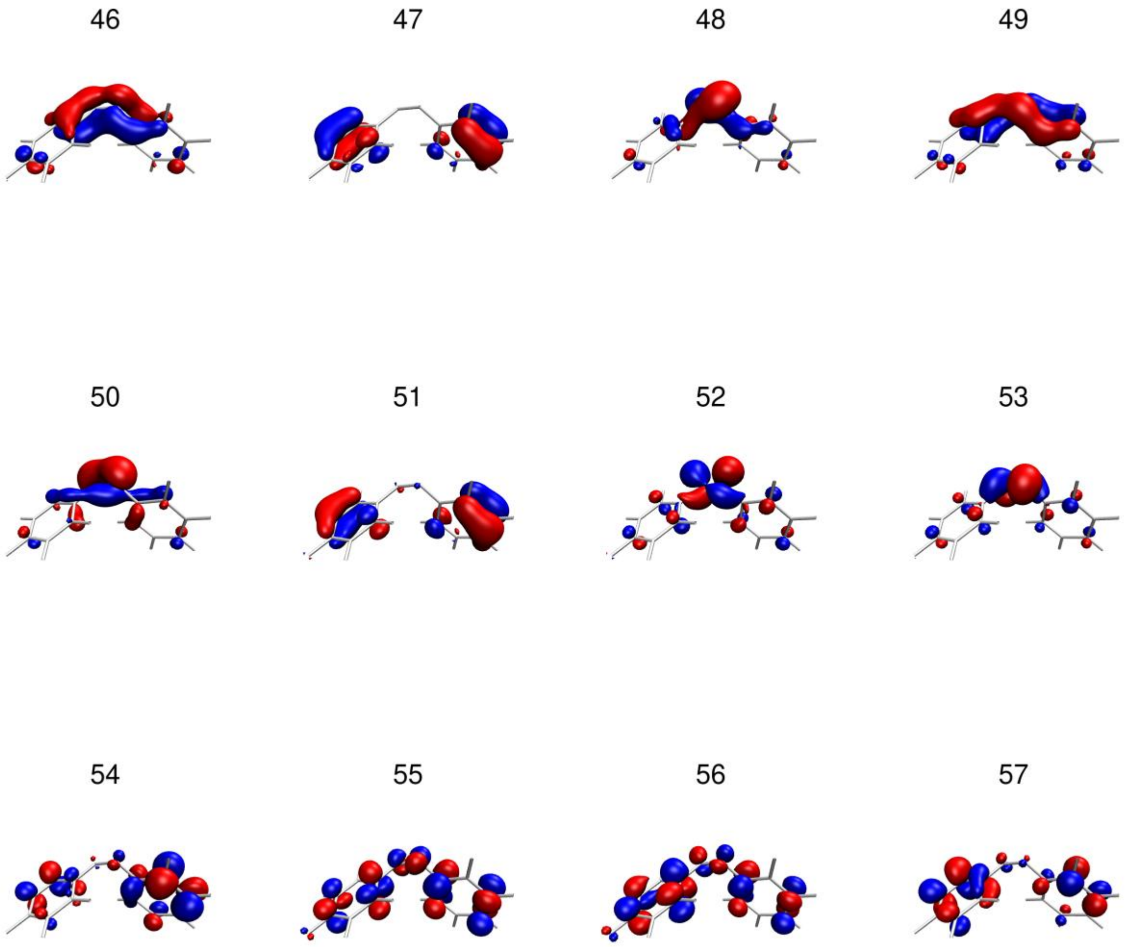

Figure S111. Active space of the TSRot of fluoroazobenzene at the CASSCF/ANO-RCC-VTZP//ωB97X-3c/SMD(toluene) level of theory. The digits represent the orbital number.

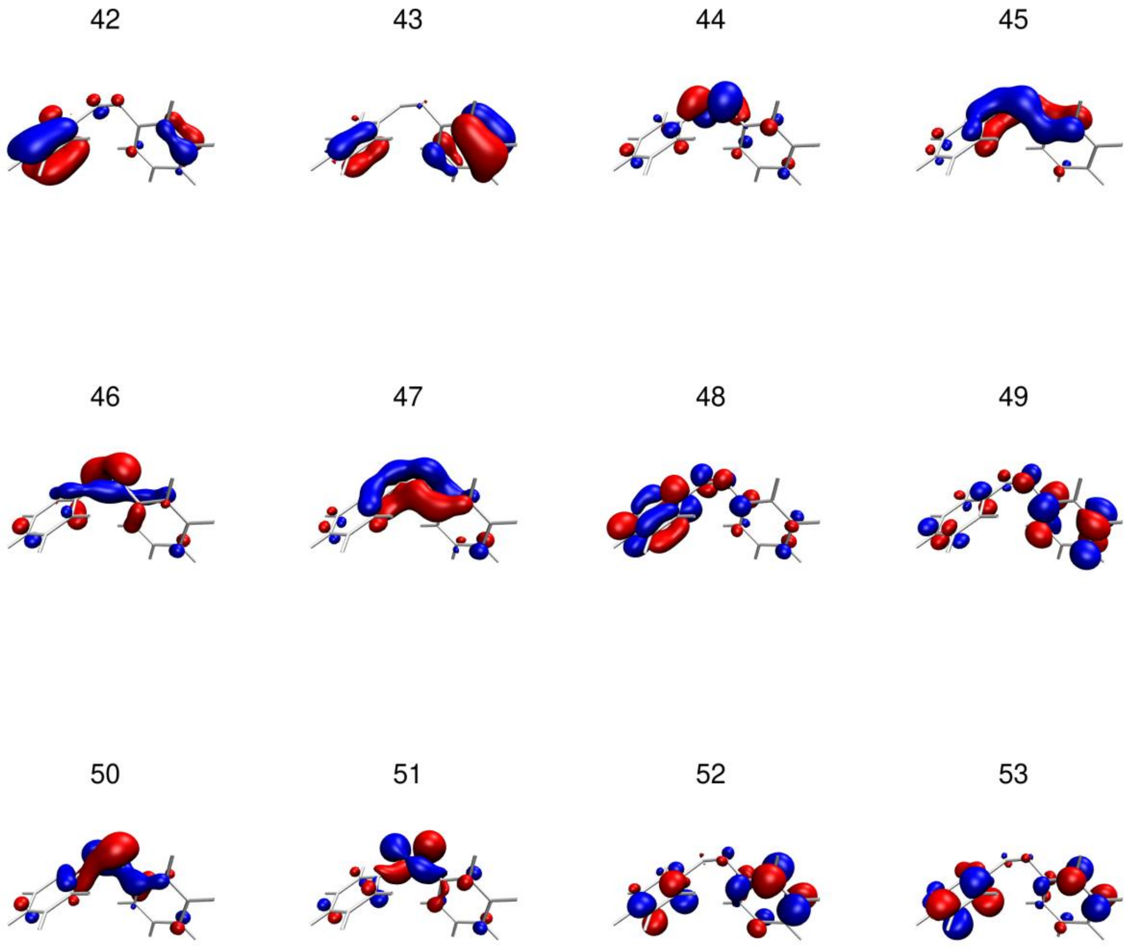

Figure S112. Active space of the MECP1 of azobenzene at the CASSCF/ANO-RCC-VTZP//ωB97X-3c/SMD(toluene) level of theory. The digits represent the orbital number.

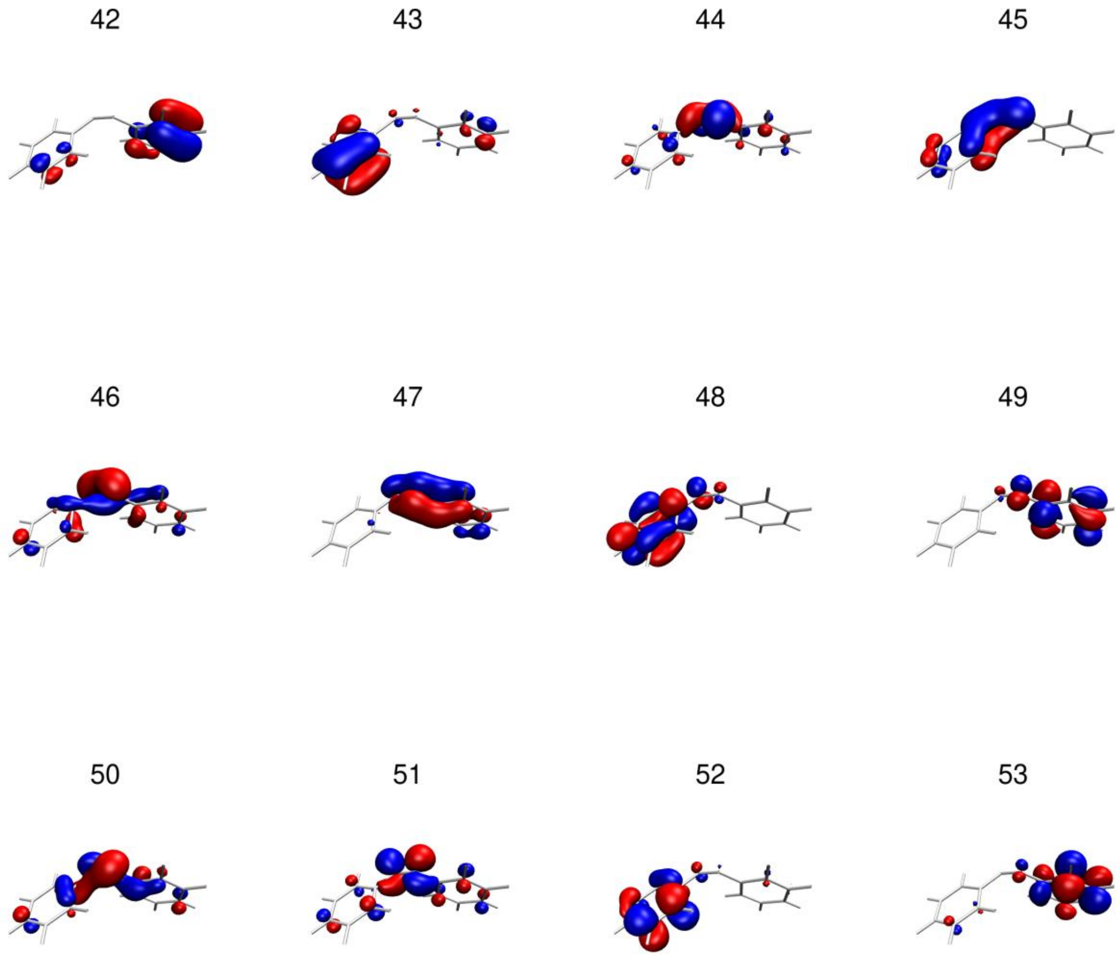

Figure S113. Active space of the MECP2 of azobenzene at the CASSCF/ANO-RCC-VTZP//ωB97X-3c/SMD(toluene) level of theory. The digits represent the orbital number.

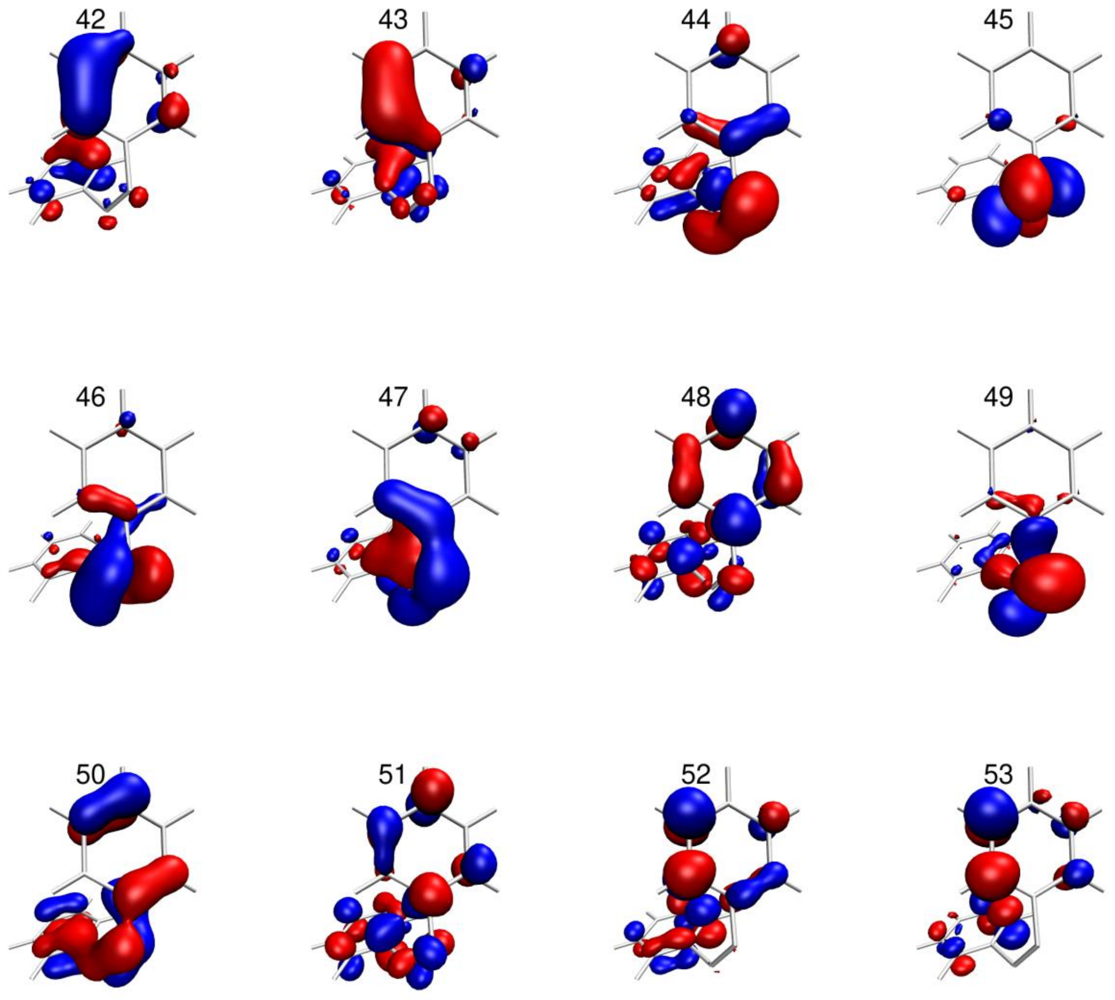

Figure S114. Active space of the Z-form of azobenzene at the CASSCF/ANO-RCC-VTZP//ωB97X-3c/SMD(toluene) level of theory. The digits represent the orbital number.

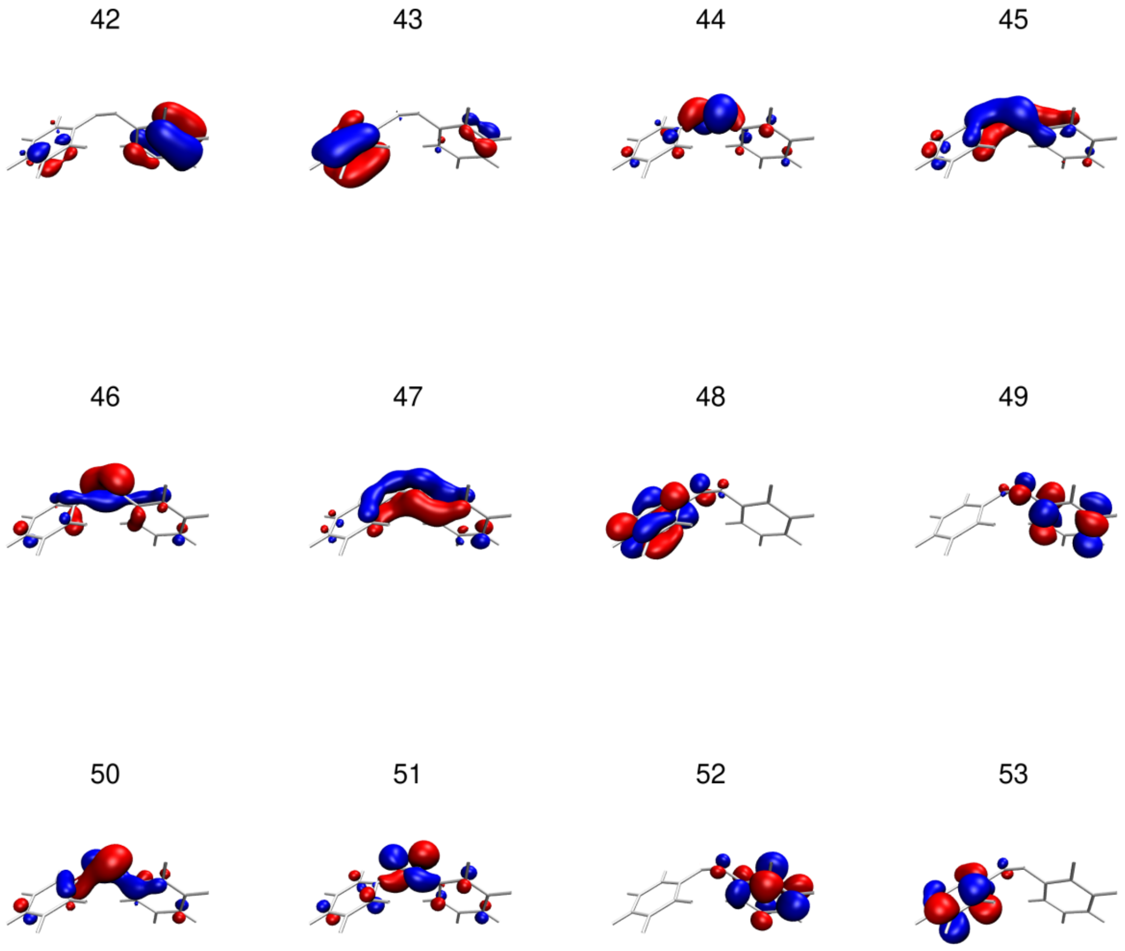

Figure S115. Active space of the T_1 minimum of azobenzene at the CASSCF/ANO-RCC-VTZP//ωB97X-3c/SMD(toluene) level of theory. The digits represent the orbital number.

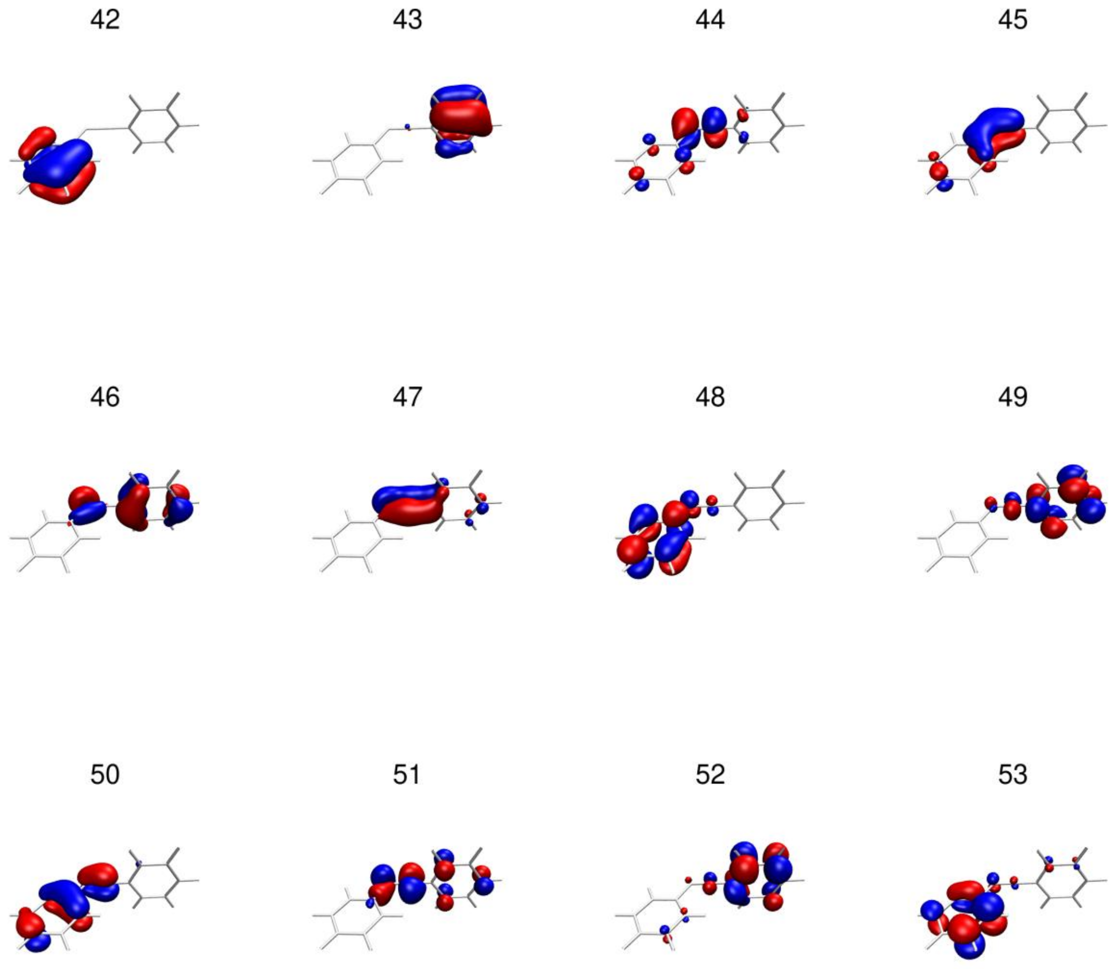

Figure S116. Active space of the TSInv1 of azobenzene at the CASSCF/ANO-RCC-VTZP//ωB97X-3c/SMD(toluene) level of theory. The digits represent the orbital number.

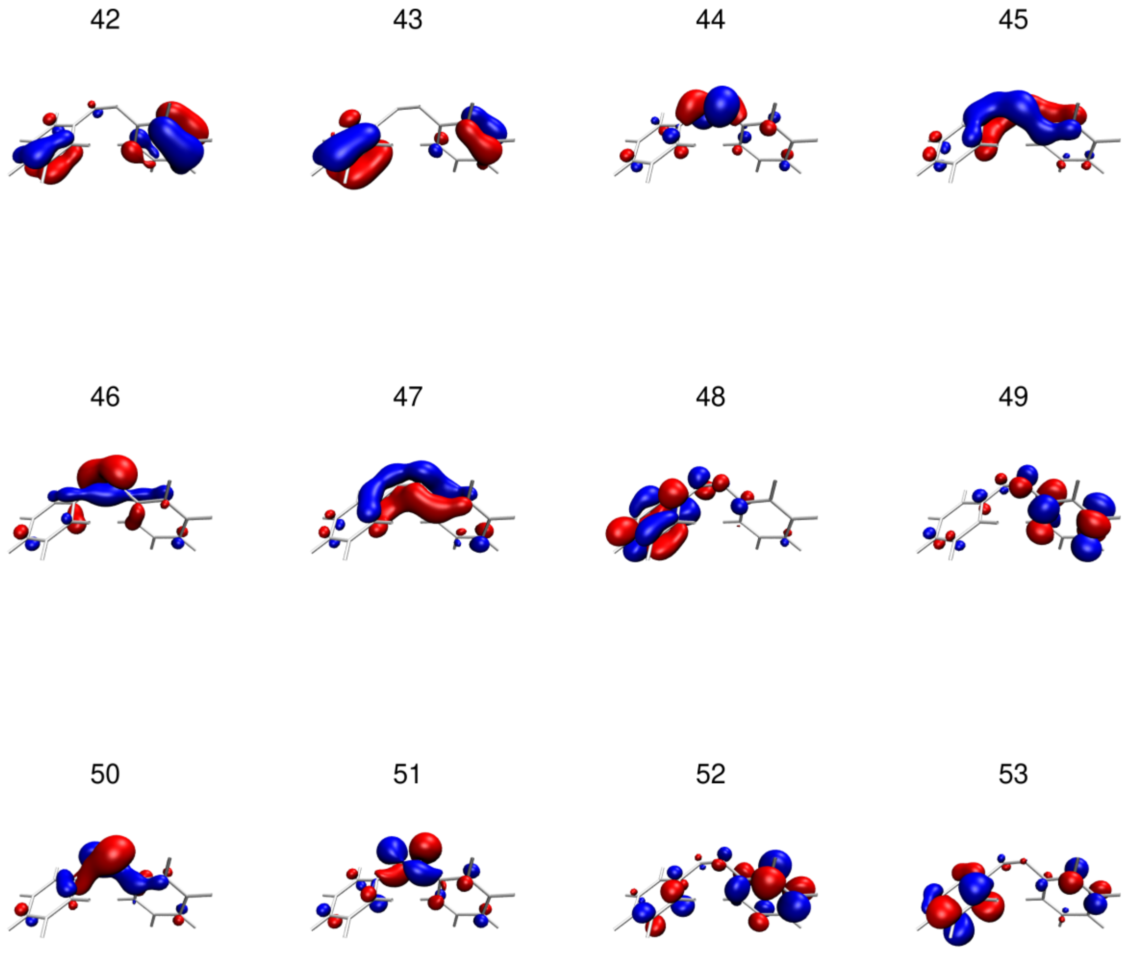

Figure S117. Active space of the TSRot of azobenzene at the CASSCF/ANO-RCC-VTZP//ωB97X-3c/SMD(toluene) level of theory. The digits represent the orbital number.

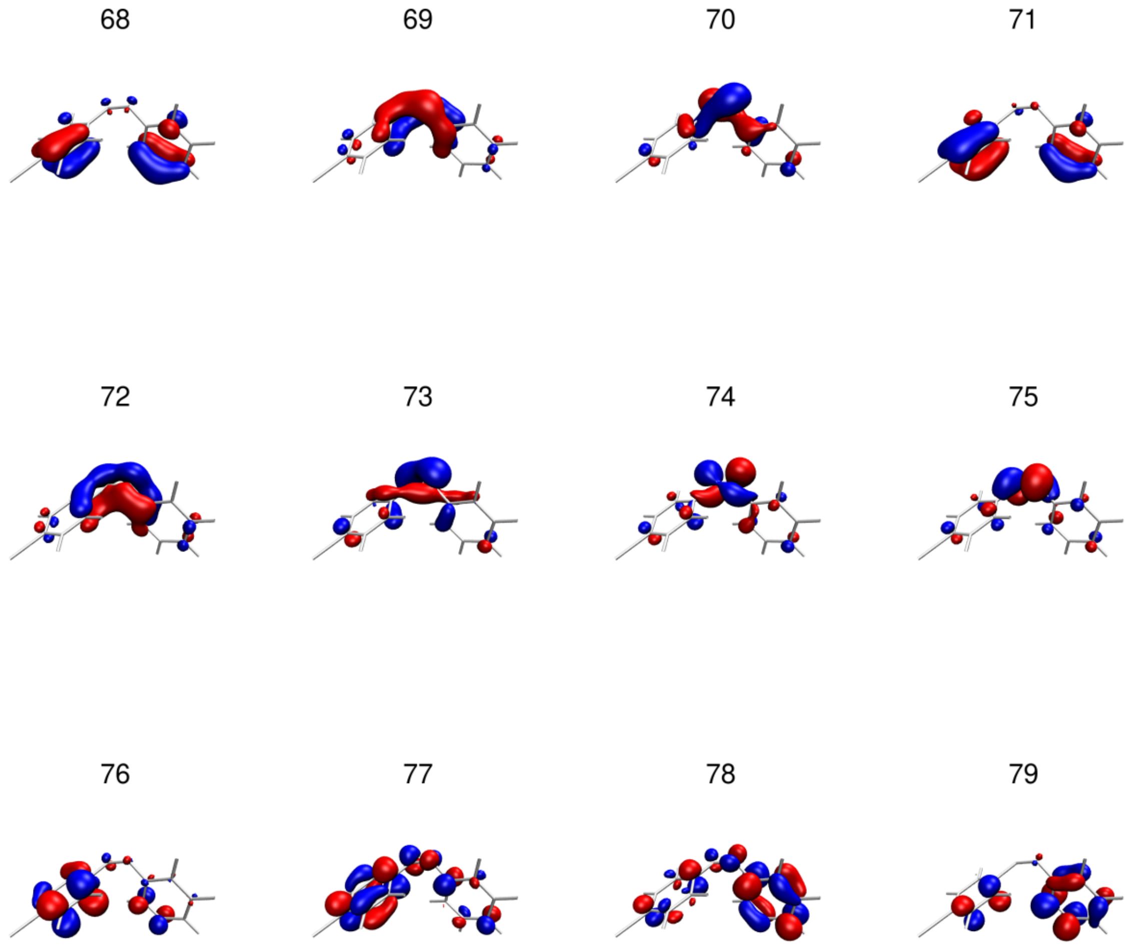

Figure S118. Active space of the MECP1 of iodoazobenzene at the CASSCF/ANO-RCC-VTZP//ωB97X-3c/SMD(toluene) level of theory. The digits represent the orbital number.

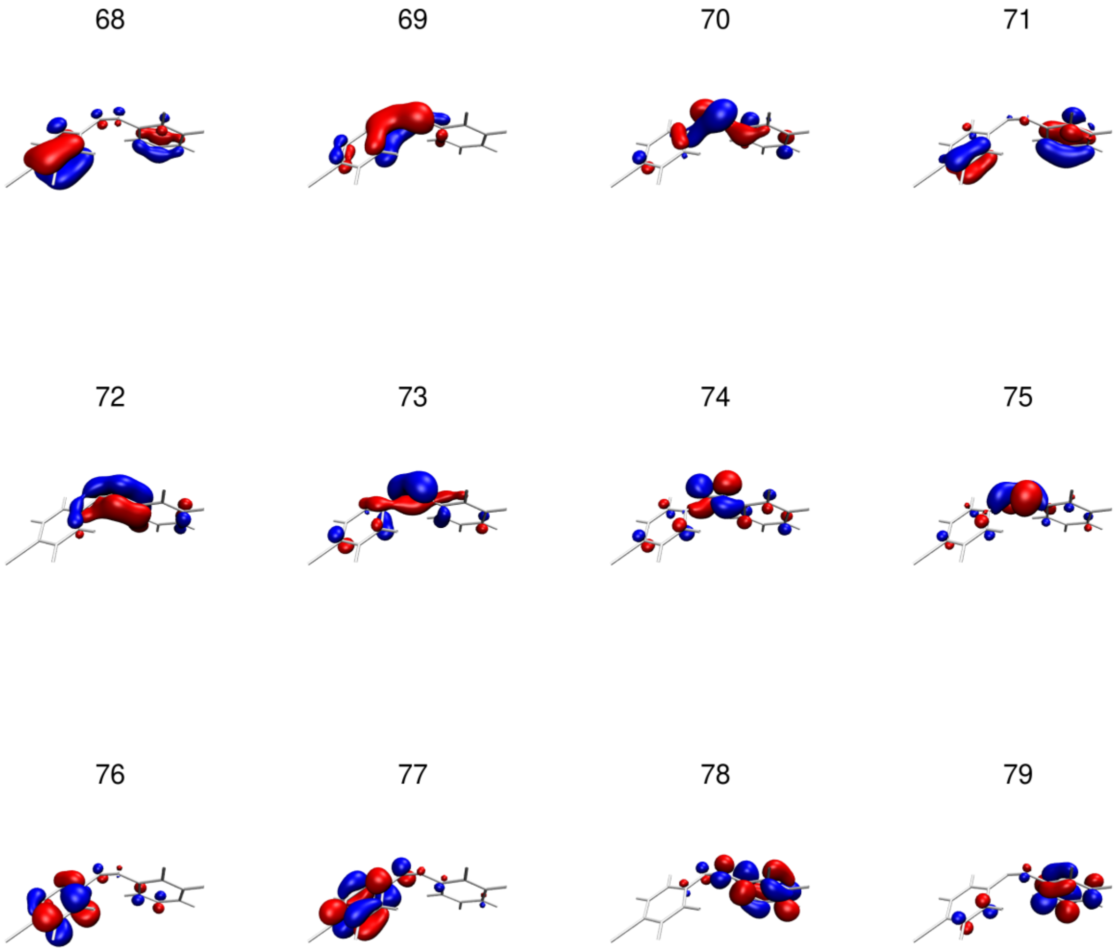

Figure S119. Active space of the MECP2 of iodoazobenzene at the CASSCF/ANO-RCC-VTZP//ωB97X-3c/SMD(toluene) level of theory. The digits represent the orbital number.

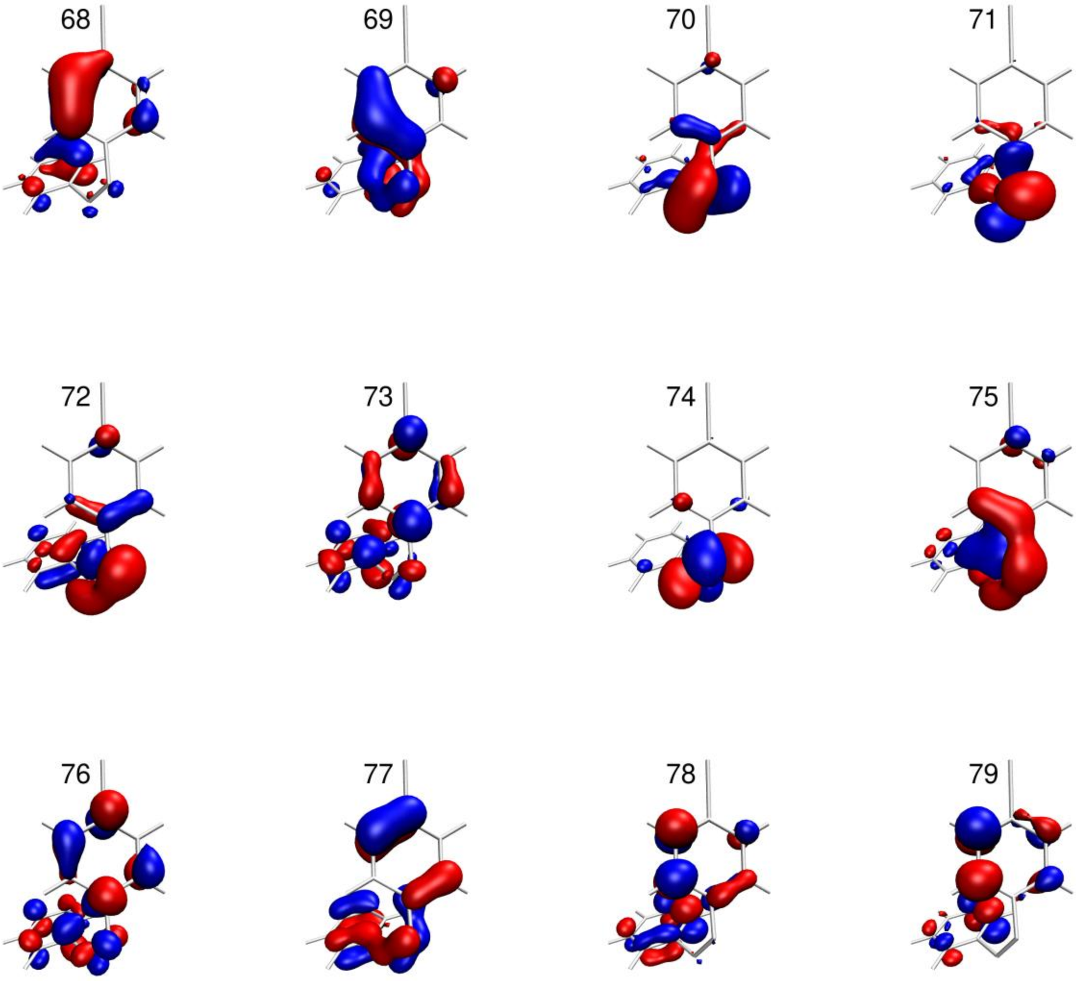

Figure S120. Active space of the Z-form of iodoazobenzene at the CASSCF/ANO-RCC-VTZP//ωB97X-3c/SMD(toluene) level of theory. The digits represent the orbital number.

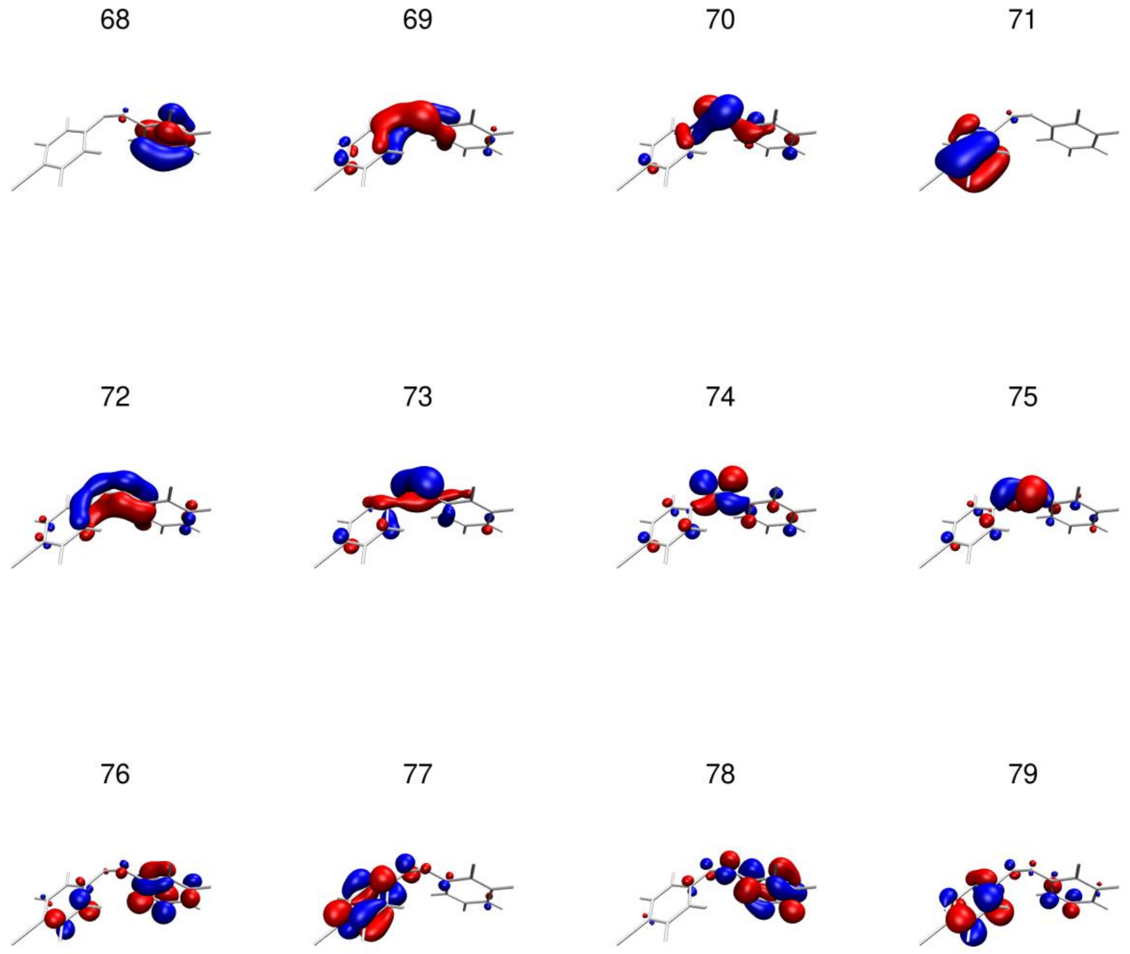

Figure S121. Active space of the T_1 minimum of iodoazobenzene at the CASSCF/ANO-RCC-VTZP//ωB97X-3c/SMD(toluene) level of theory. The digits represent the orbital number.

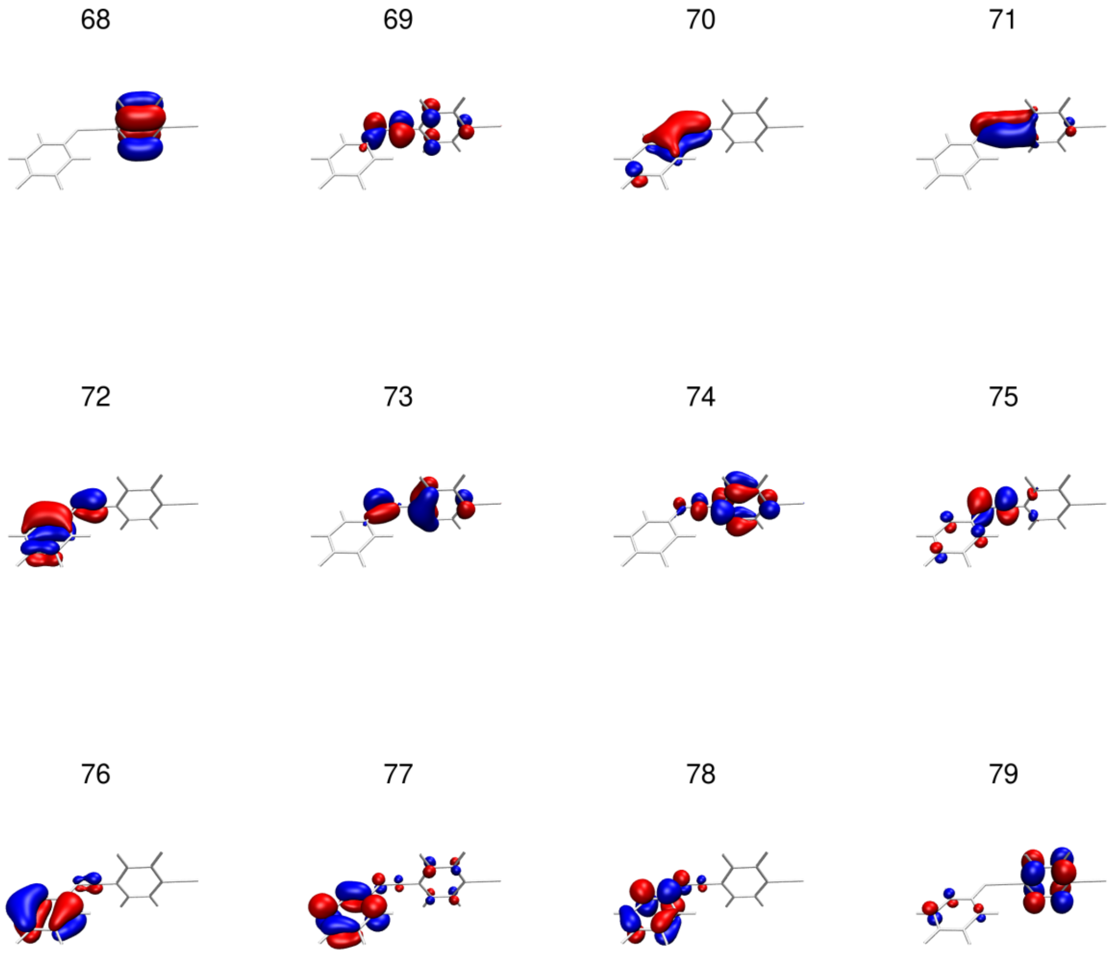

Figure S122. Active space of the TSInv1 of iodoazobenzene at the CASSCF/ANO-RCC-VTZP//ωB97X-3c/SMD(toluene) level of theory. The digits represent the orbital number.

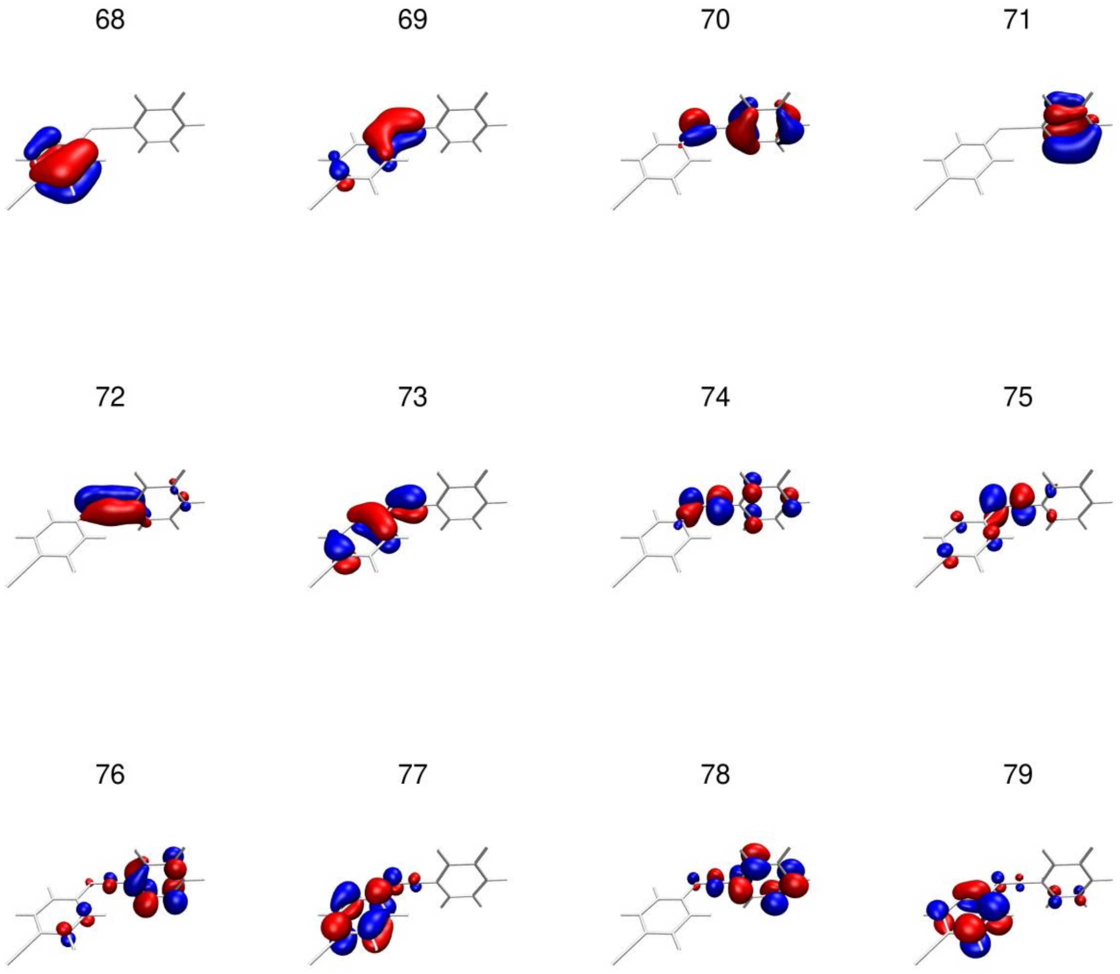

Figure S123. Active space of the TSInv2 of iodoazobenzene at the CASSCF/ANO-RCC-VTZP//ωB97X-3c/SMD(toluene) level of theory. The digits represent the orbital number.

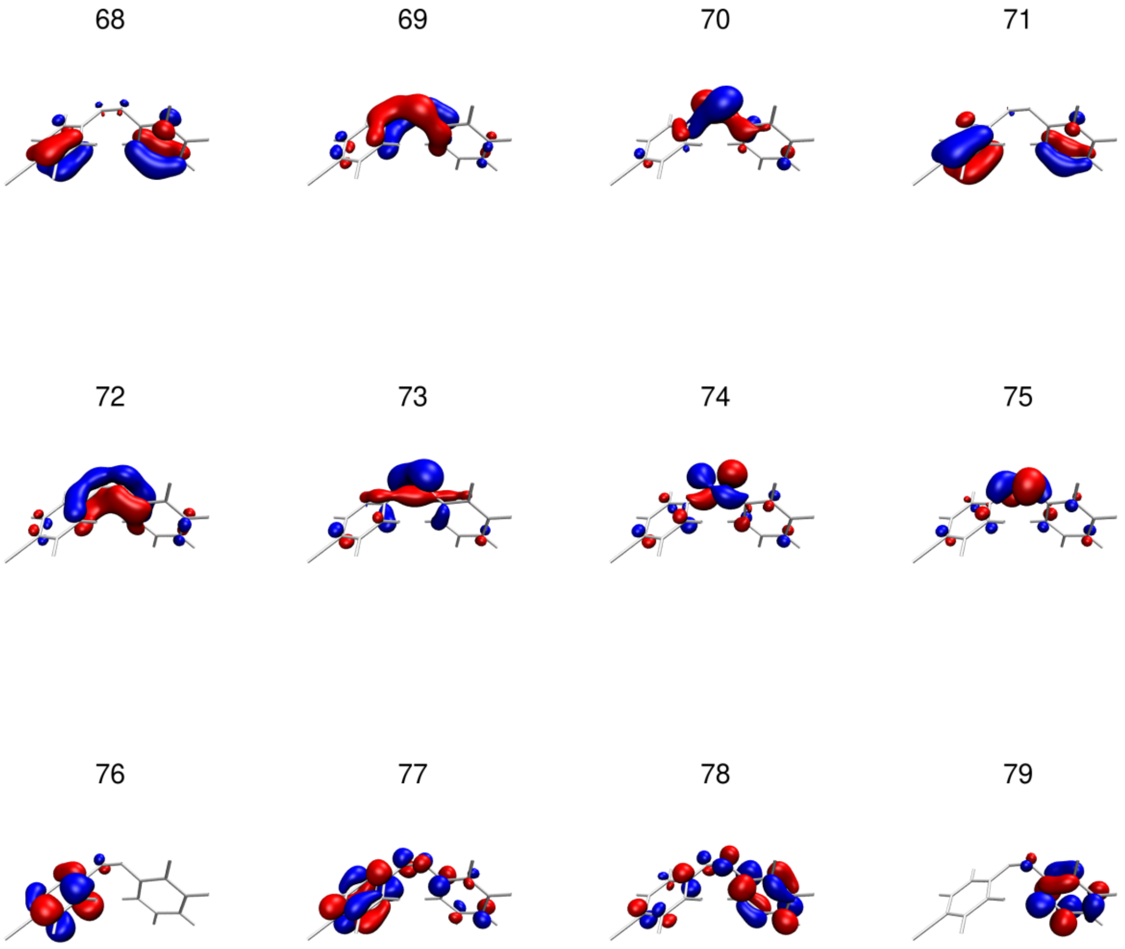

Figure S124. Active space of the TSRot of iodoazobenzene at the CASSCF/ANO-RCC-VTZP//ωB97X-3c/SMD(toluene) level of theory. The digits represent the orbital number.

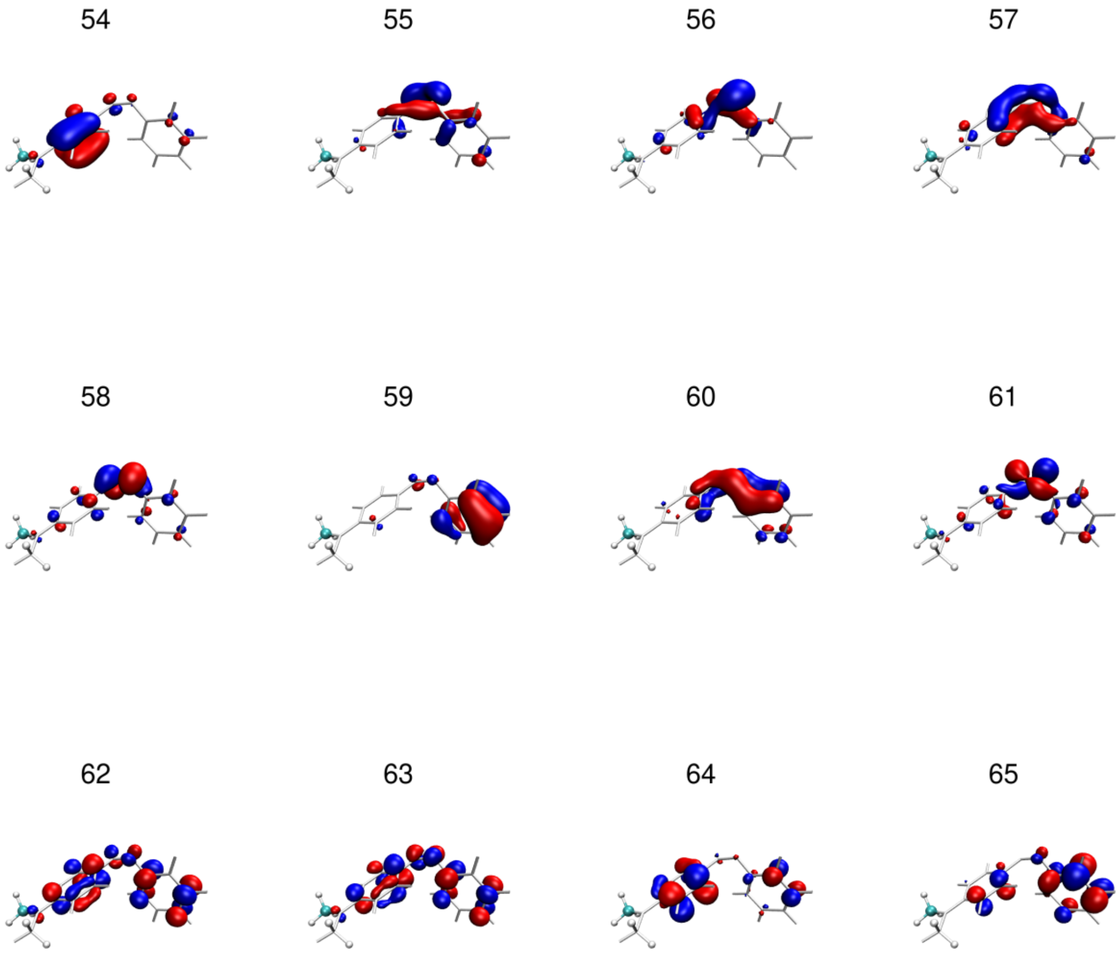

Figure S125. Active space of the MECP1 of NMe_2-azobenzene at the CASSCF/ANO-RCC-VTZP//ωB97X-3c/SMD(toluene) level of theory. The digits represent the orbital number.

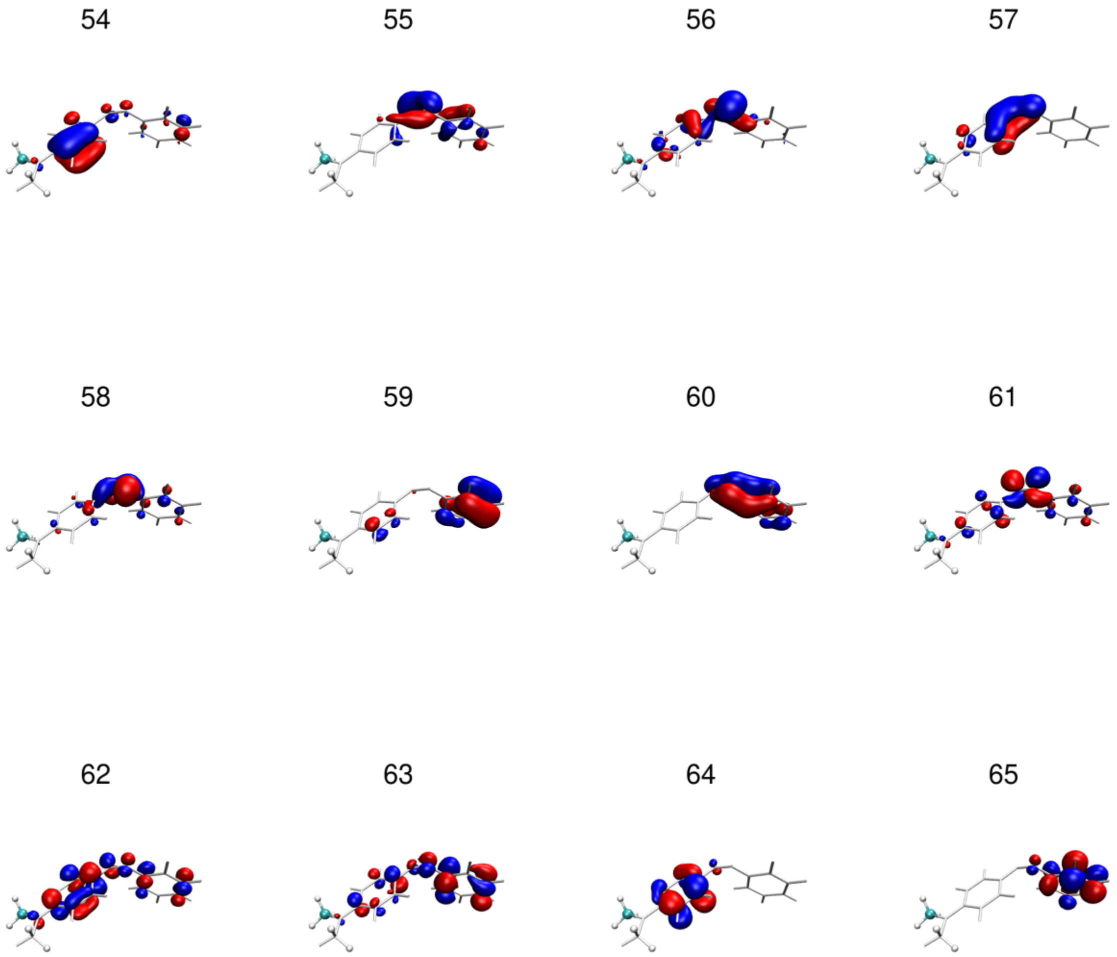

Figure S126. Active space of the MECP2 of NMe_2-azobenzene at the CASSCF/ANO-RCC-VTZP//ωB97X-3c/SMD(toluene) level of theory. The digits represent the orbital number.

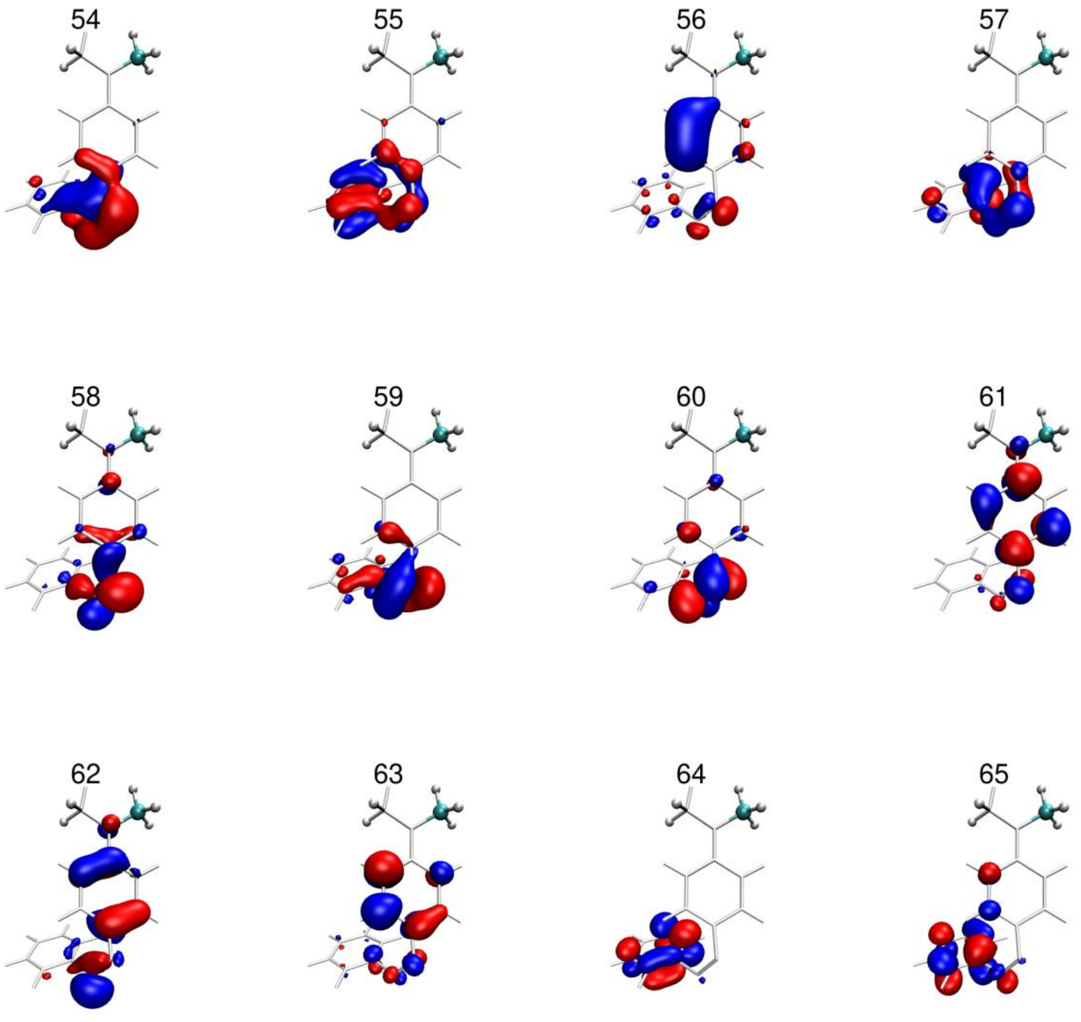

Figure S127. Active space of the Z-form of NMe_2-azobenzene at the CASSCF/ANO-RCC-VTZP//ωB97X-3c/SMD(toluene) level of theory. The digits represent the orbital number.

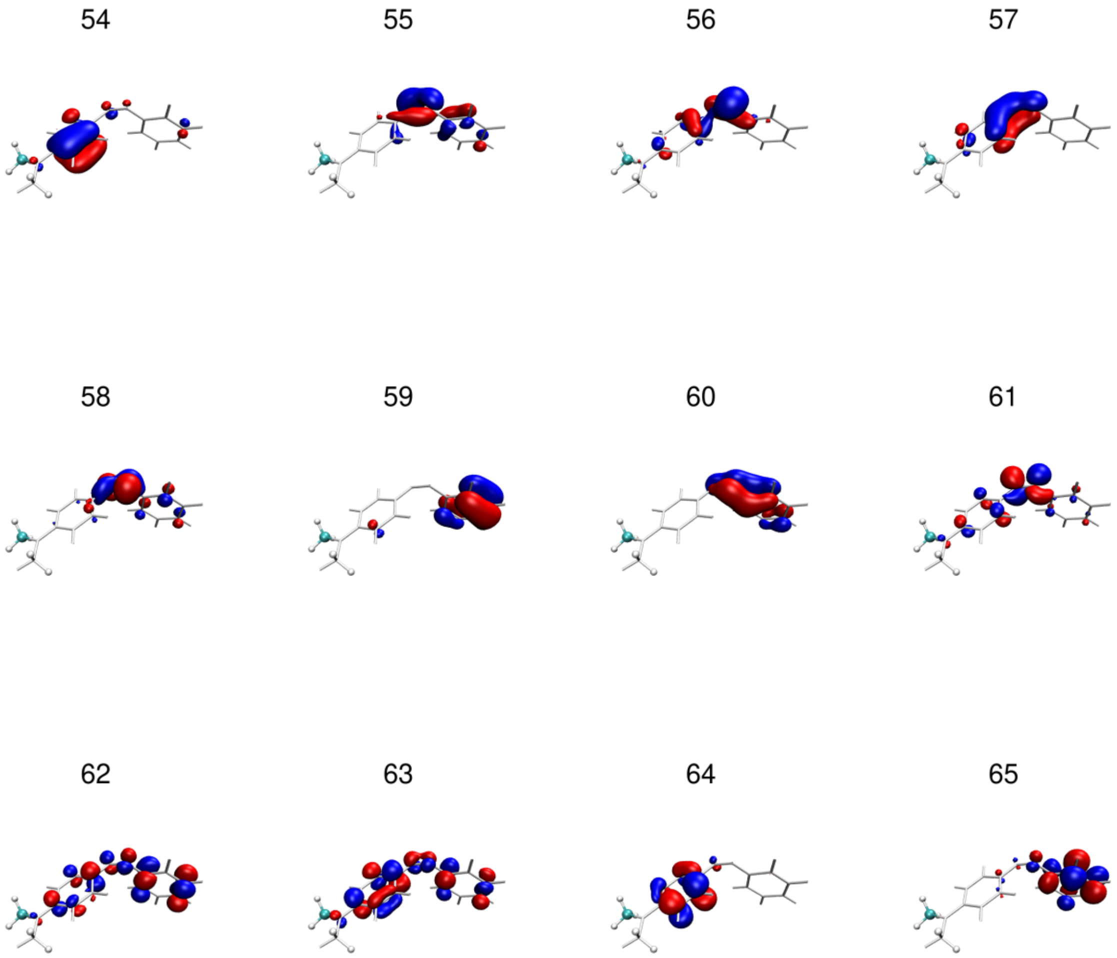

Figure S128. Active space of the T_1 minimum of NMe_2-azobenzene at the CASSCF/ANO-RCC-VTZP//ωB97X-3c/SMD(toluene) level of theory. The digits represent the orbital number.

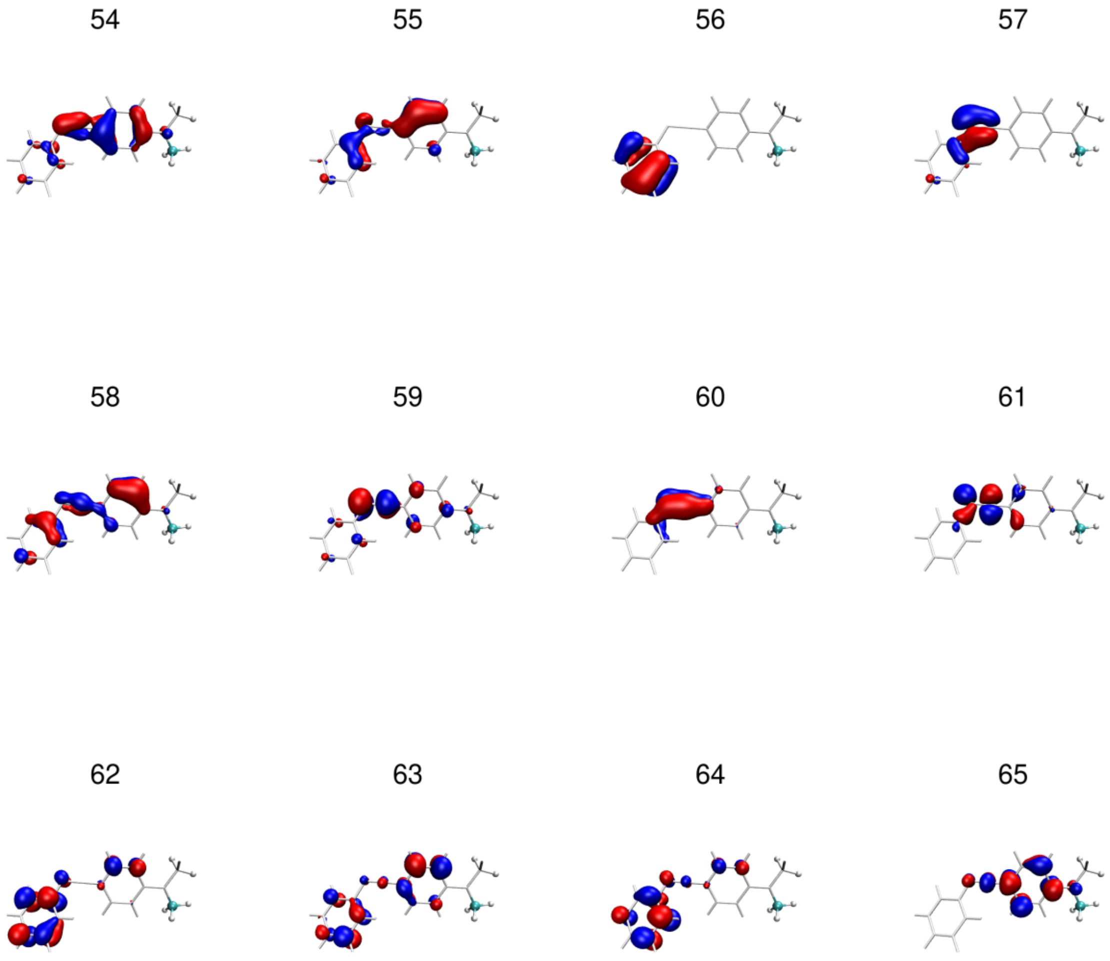

Figure S129. Active space of the TSInv1 of NMe_2-azobenzene at the CASSCF/ANO-RCC-VTZP//ωB97X-3c/SMD(toluene) level of theory. The digits represent the orbital number.

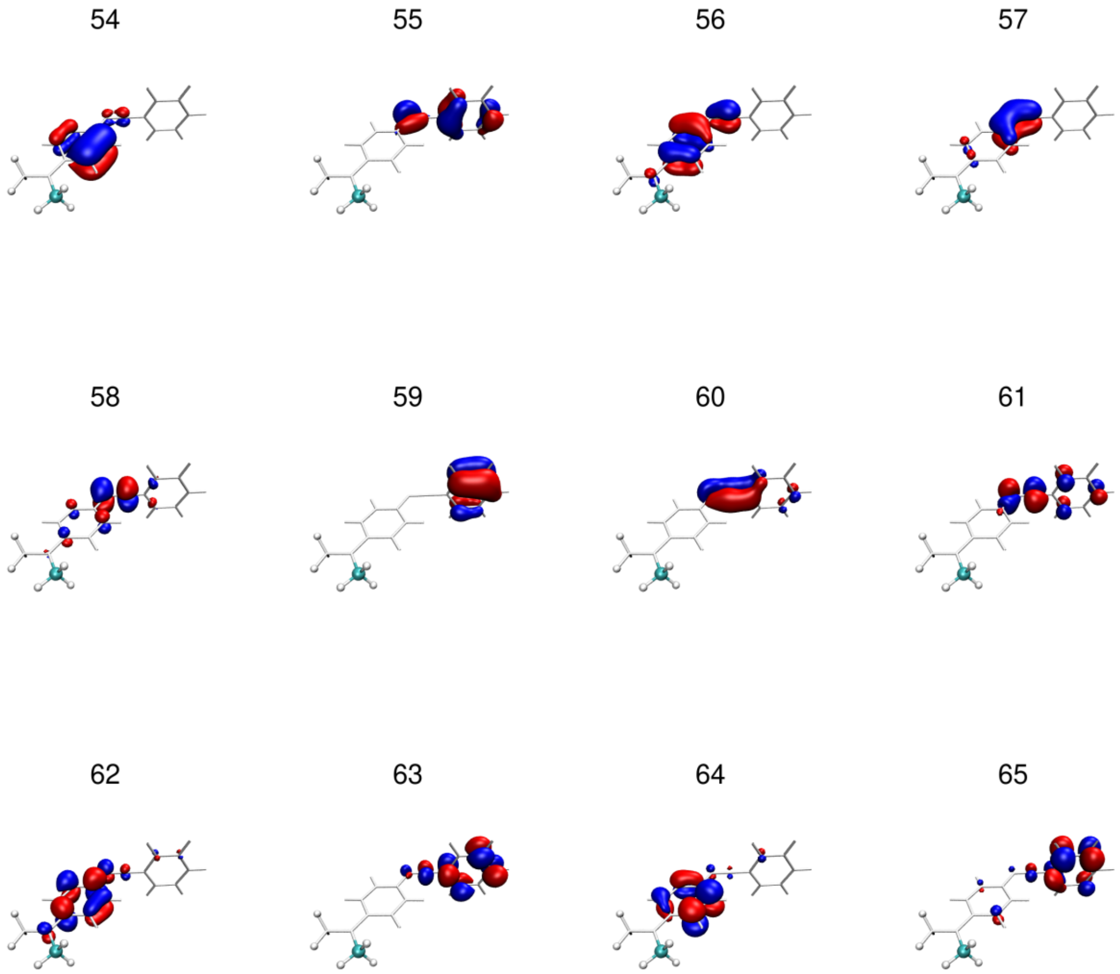

Figure S130. Active space of the TSInv2 of NMe_2-azobenzene at the CASSCF/ANO-RCC-VTZP//ωB97X-3c/SMD(toluene) level of theory. The digits represent the orbital number.

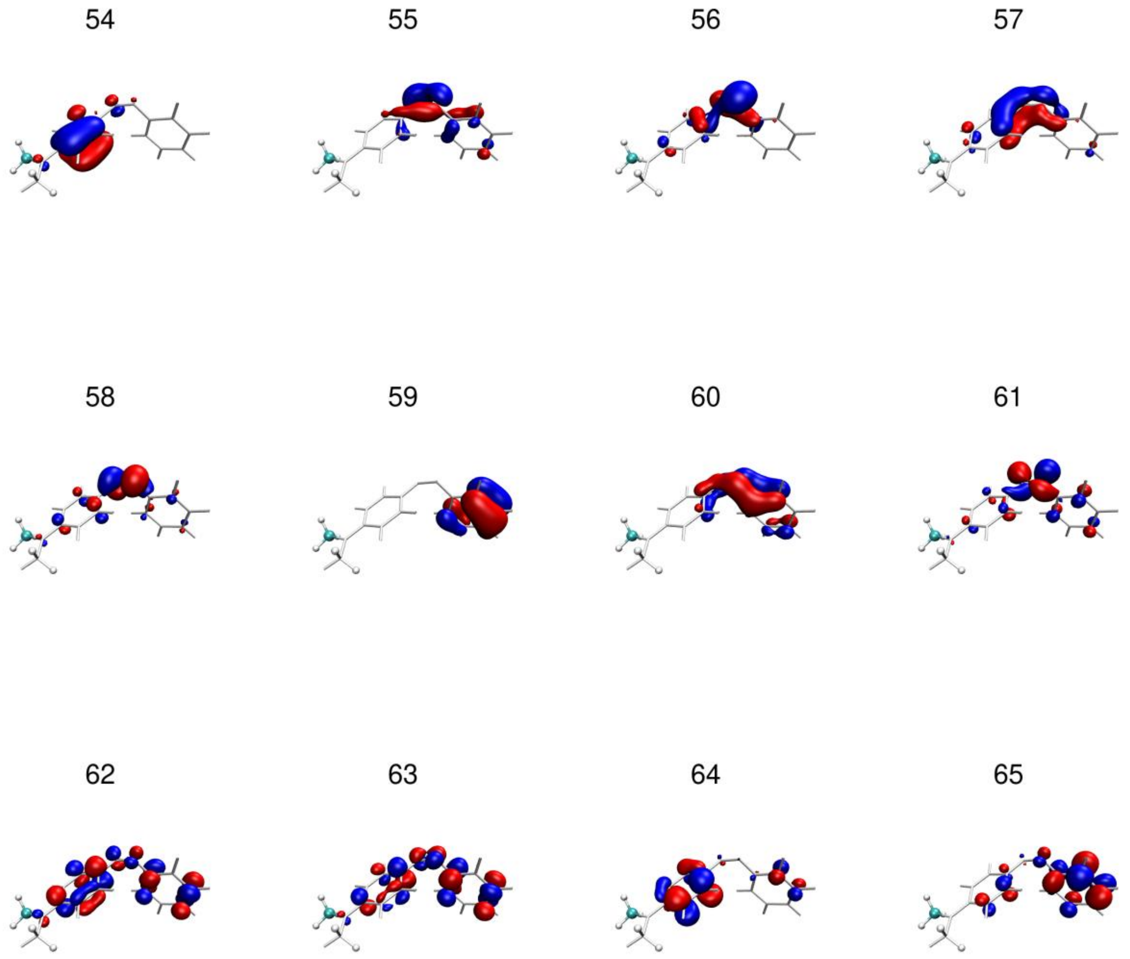

Figure S131. Active space of the TSRot of NMe_2-azobenzene at the CASSCF/ANO-RCC-VTZP//ωB97X-3c/SMD(toluene) level of theory. The digits represent the orbital number.

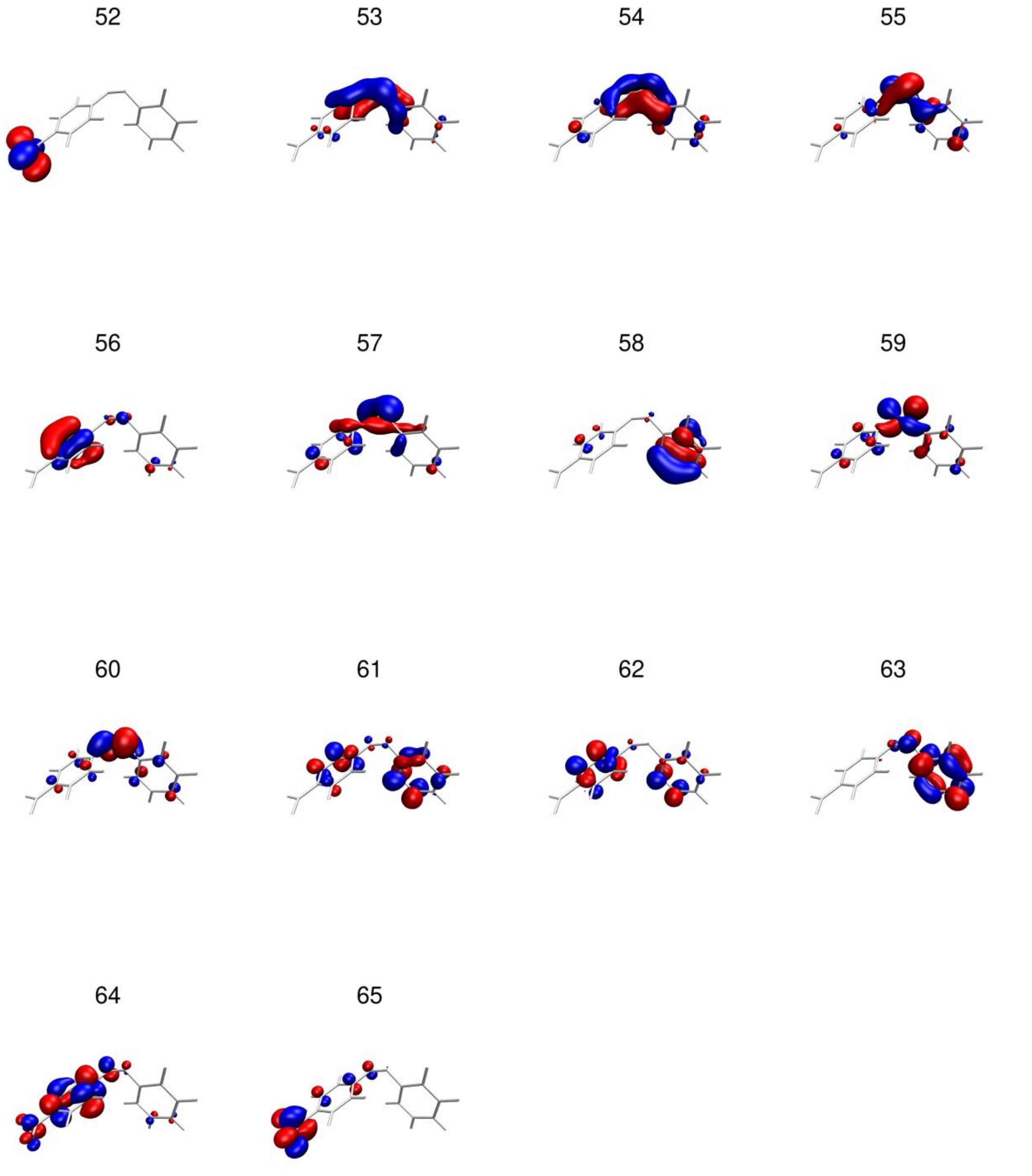

Figure S132. Active space of the MECP1 of NO_2-azobenzene at the CASSCF/ANO-RCC-VTZP//ωB97X-3c/SMD(toluene) level of theory. The digits represent the orbital number.

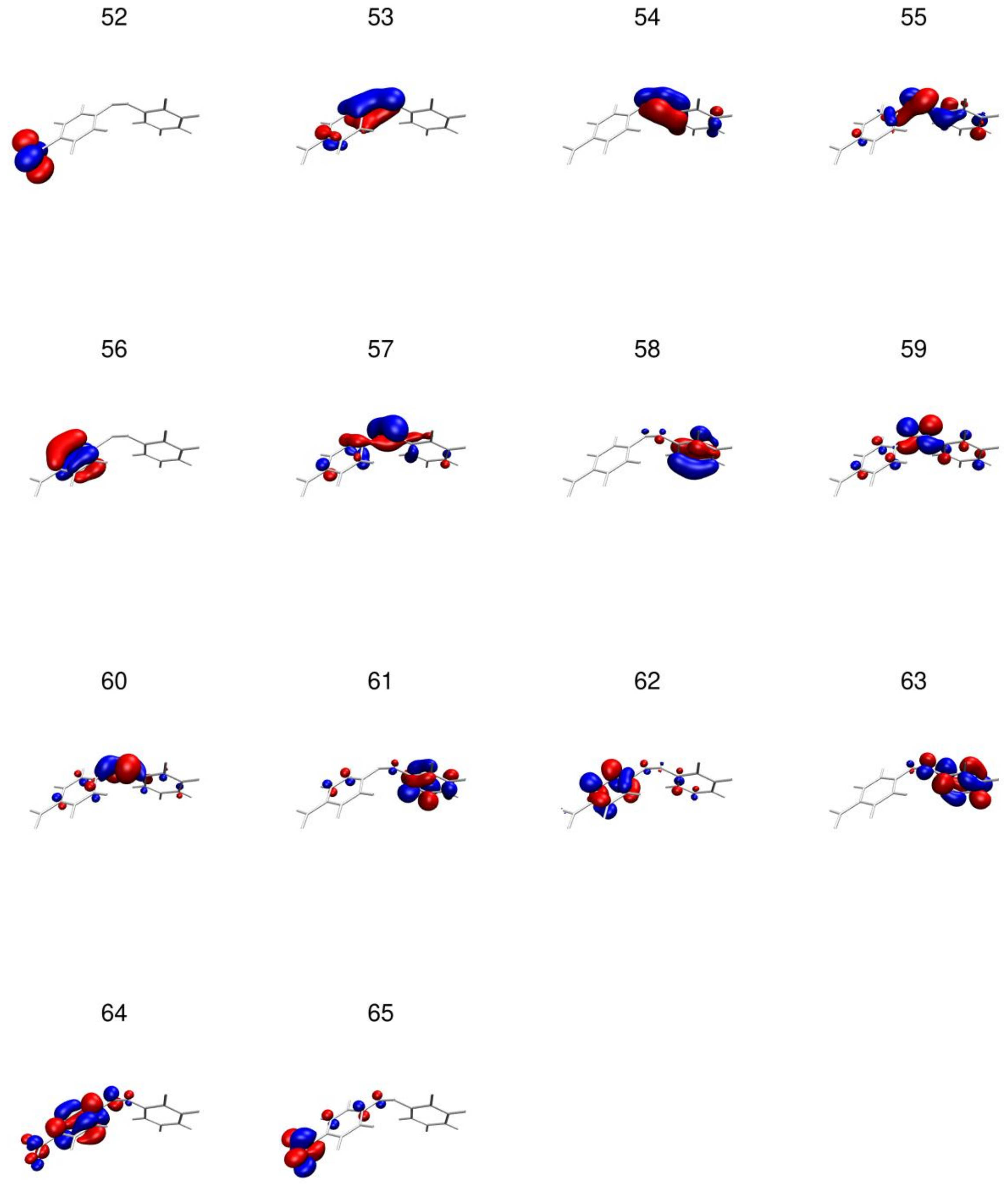

Figure S133. Active space of the MECP2 of NO_2-azobenzene at the CASSCF/ANO-RCC-VTZP//ωB97X-3c/SMD(toluene) level of theory. The digits represent the orbital number.

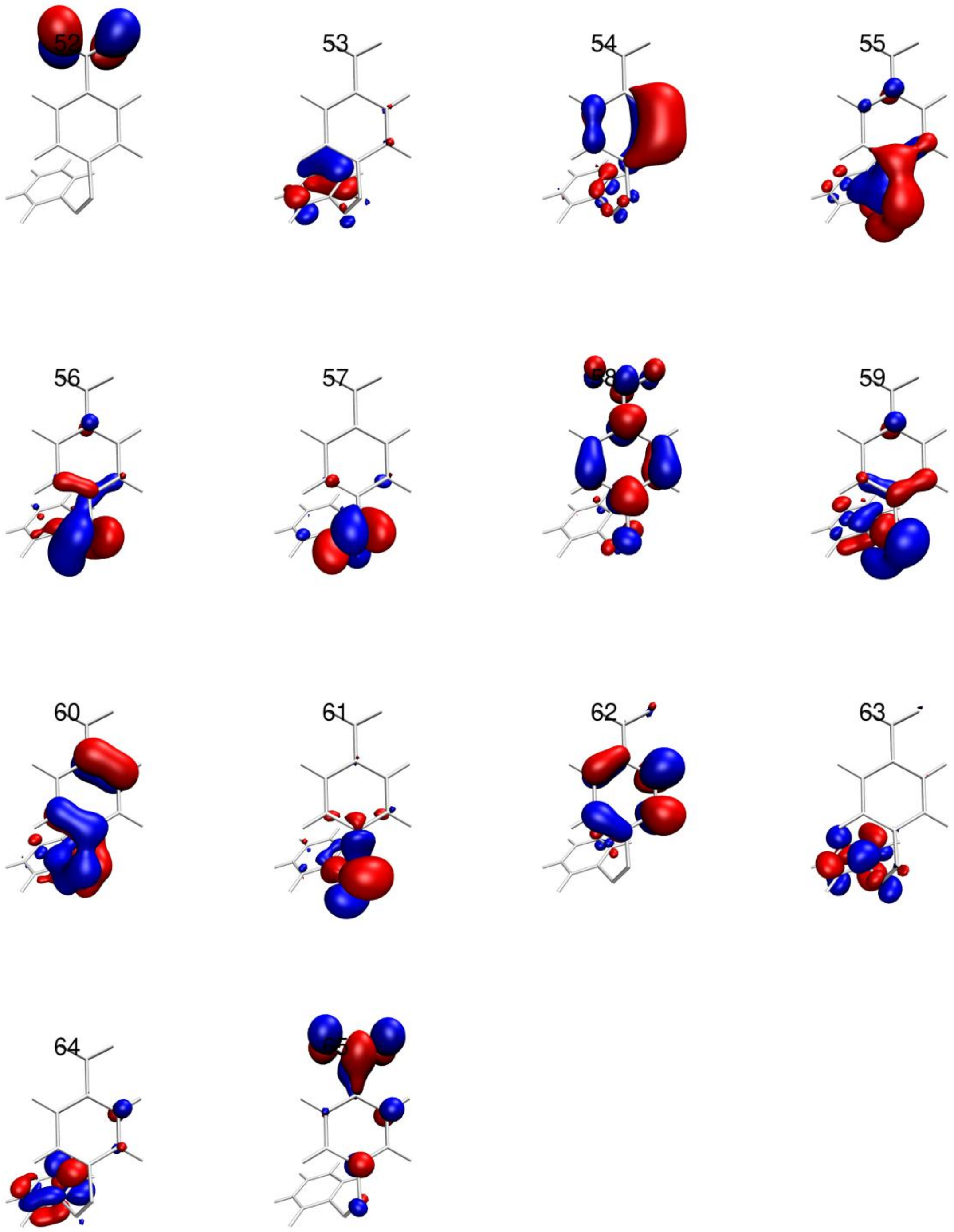

Figure S134. Active space of the Z-form of NO_2-azobenzene at the CASSCF/ANO-RCC-VTZP//ωB97X-3c/SMD(toluene) level of theory. The digits represent the orbital number.

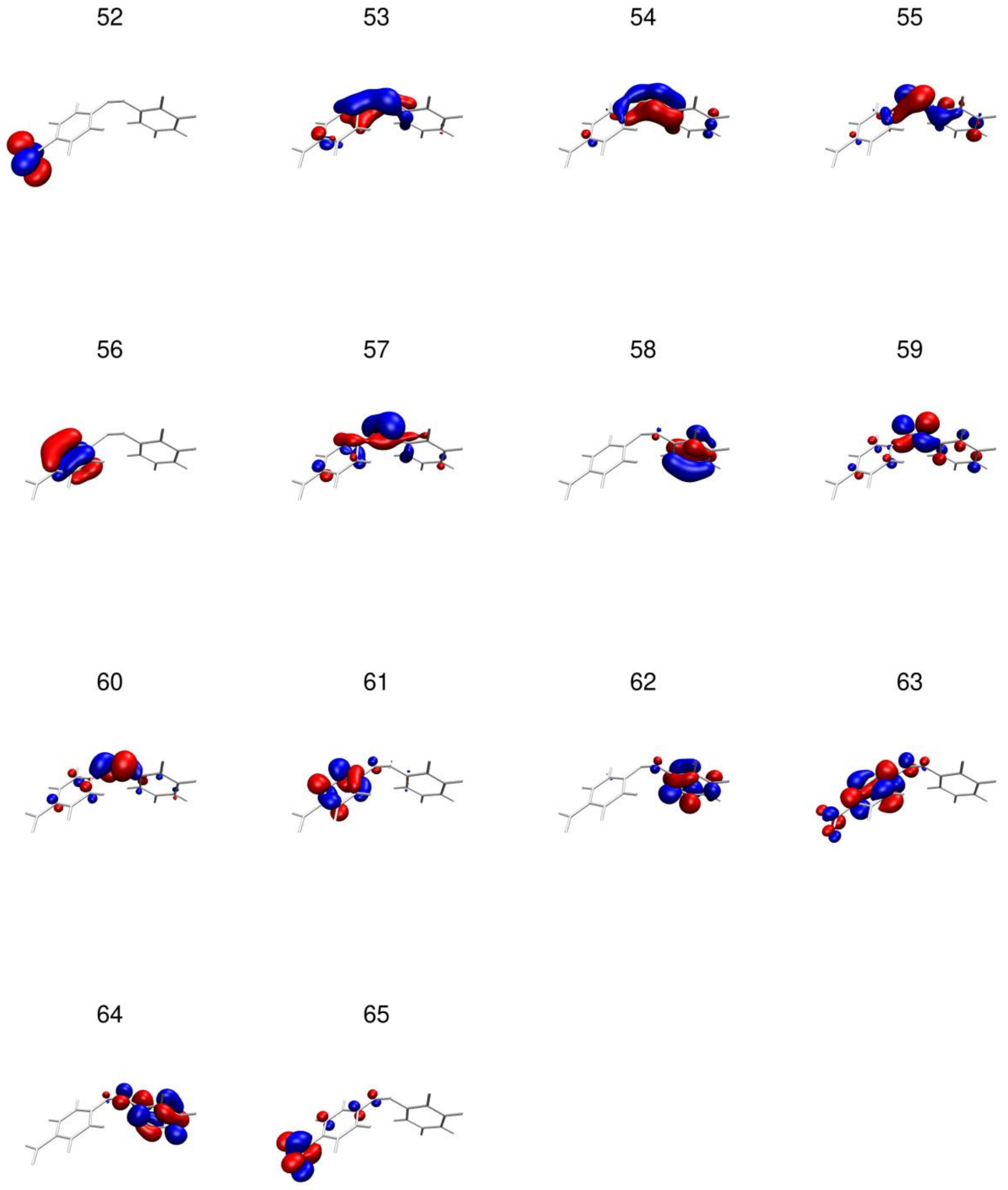

Figure S135. Active space of the T_1 minimum of NO_2-azobenzene at the CASSCF/ANO-RCC-VTZP//ωB97X-3c/SMD(toluene) level of theory. The digits represent the orbital number.

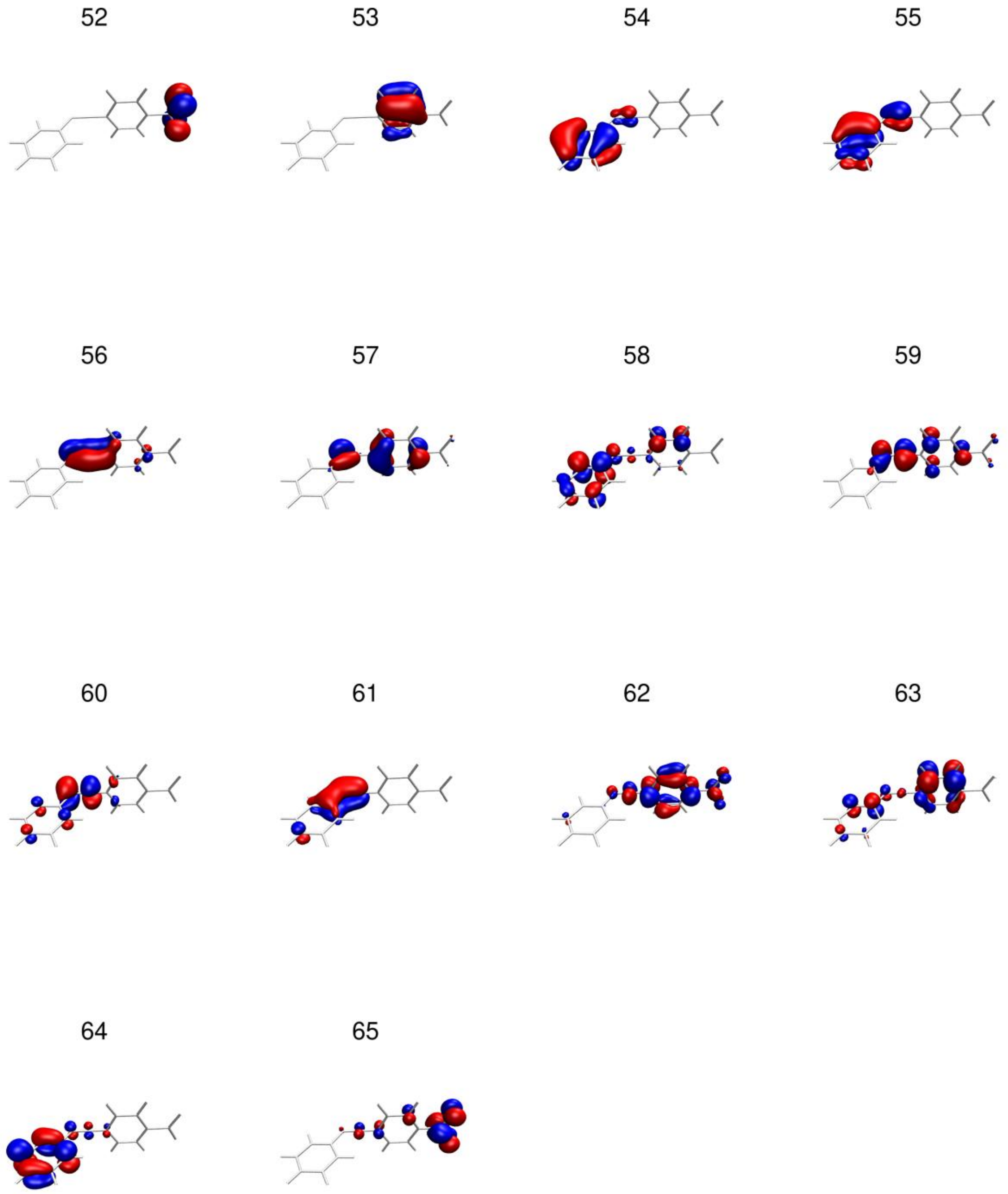

Figure S136. Active space of the TSInv1 of NO_2-azobenzene at the CASSCF/ANO-RCC-VTZP//ωB97X-3c/SMD(toluene) level of theory. The digits represent the orbital number.

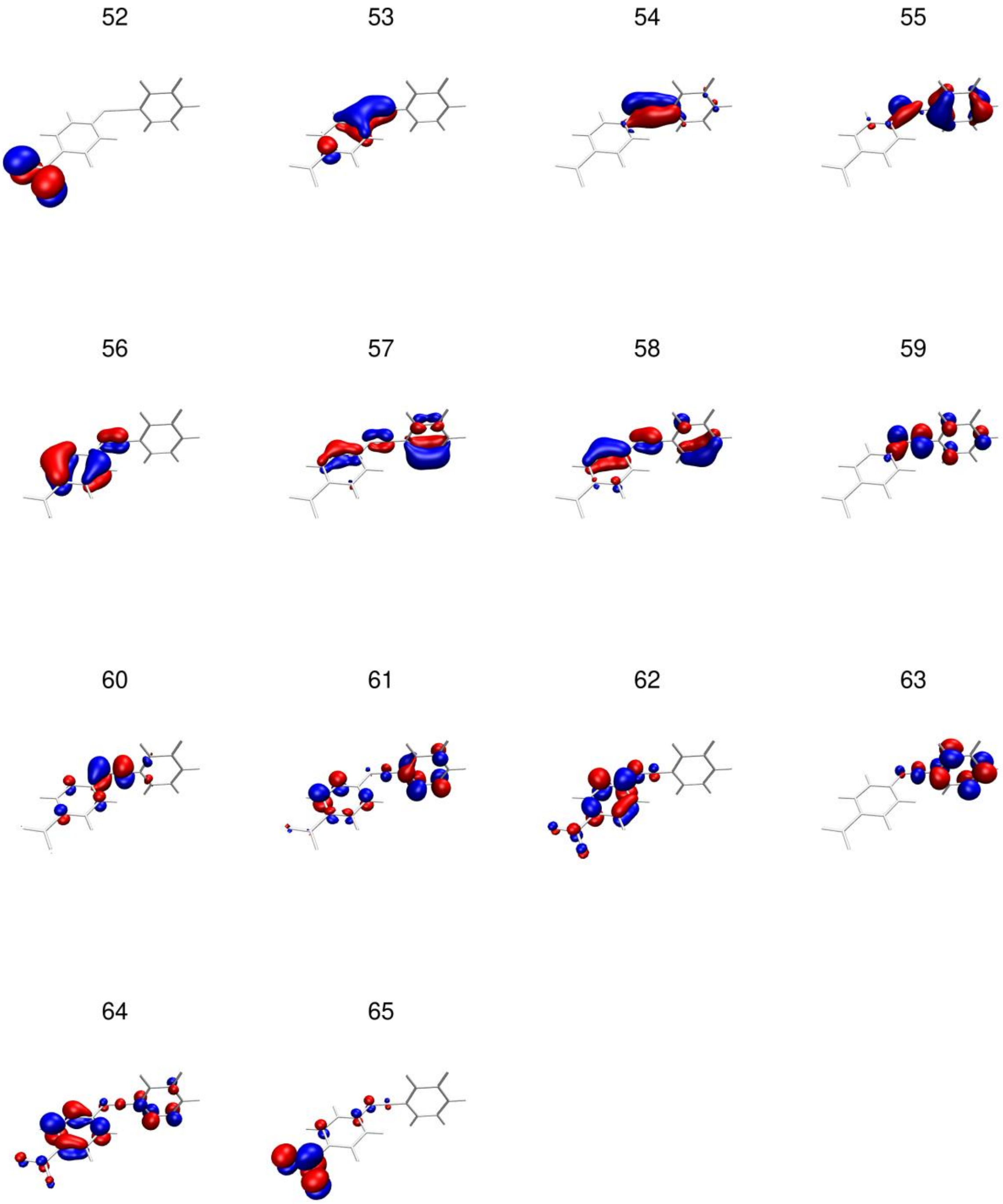

Figure S137. Active space of the TSInv2 of NO_2-azobenzene at the CASSCF/ANO-RCC-VTZP//ωB97X-3c/SMD(toluene) level of theory. The digits represent the orbital number.

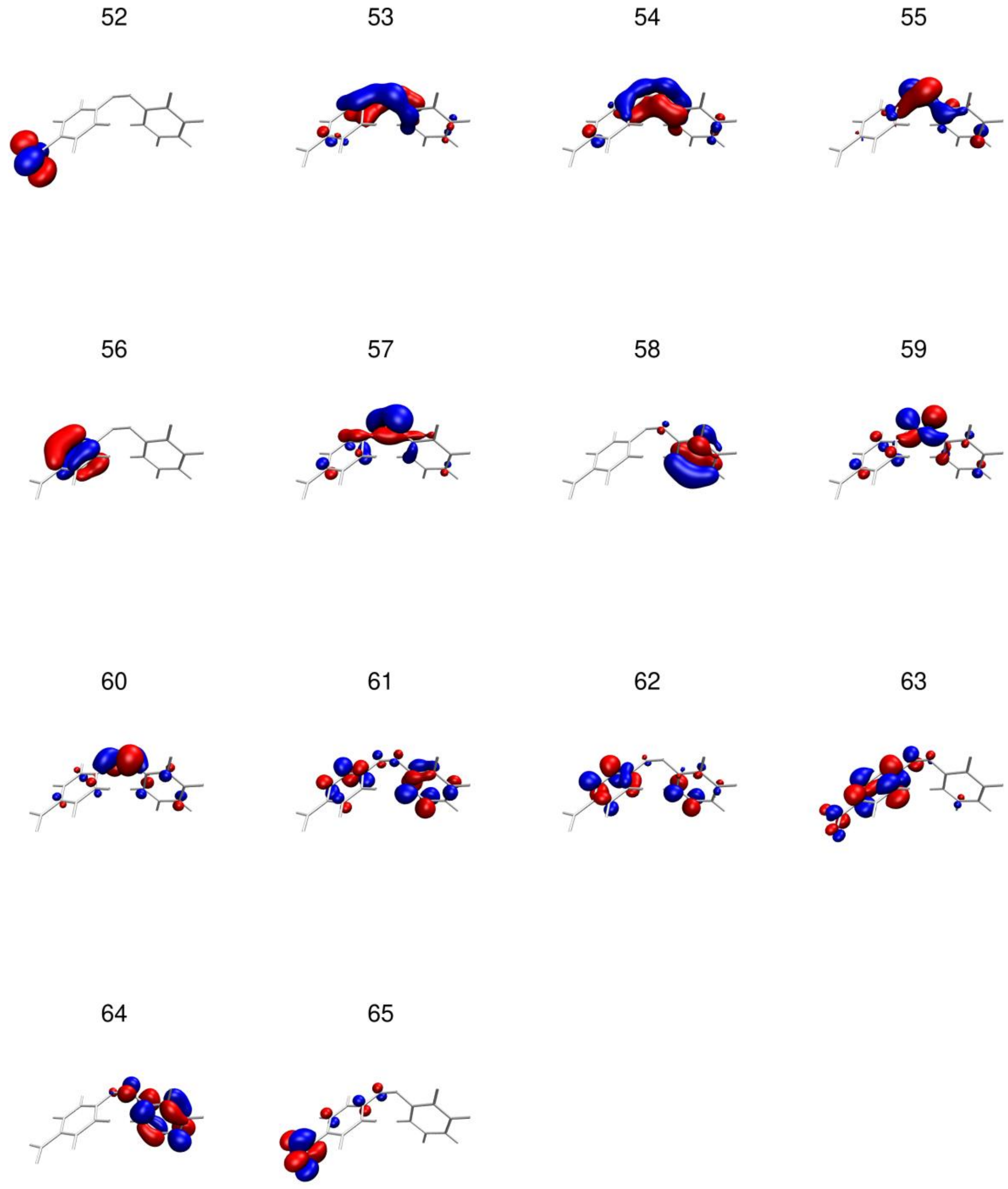

Figure S138. Active space of the TSRot of NO_2-azobenzene at the CASSCF/ANO-RCC-VTZP//ωB97X-3c/SMD(toluene) level of theory. The digits represent the orbital number.

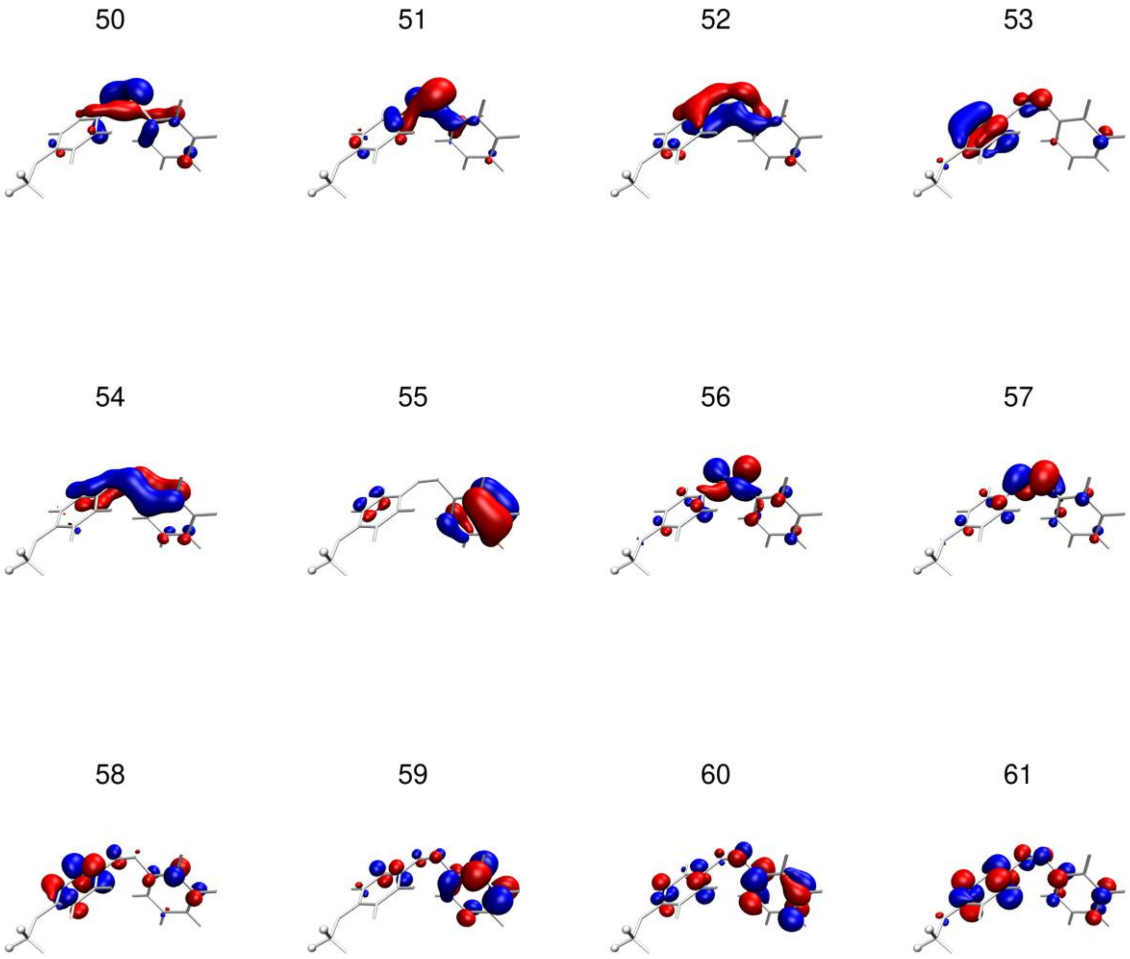

Figure S139. Active space of the MECP1 of MeO-azobenzene at the CASSCF/ANO-RCC-VTZP//ωB97X-3c/SMD(toluene) level of theory. The digits represent the orbital number.

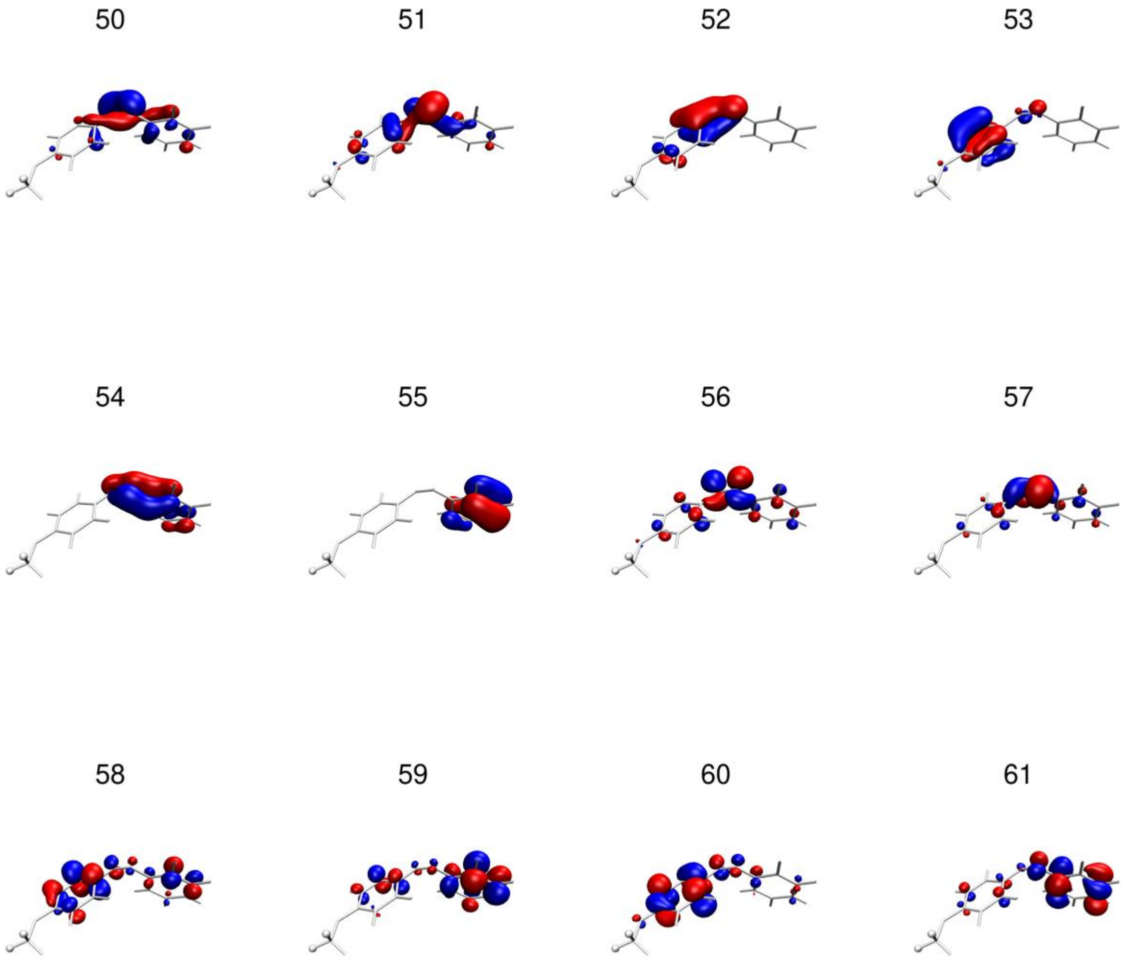

Figure S140. Active space of the MECP2 of MeO-azobenzene at the CASSCF/ANO-RCC-VTZP//ωB97X-3c/SMD(toluene) level of theory. The digits represent the orbital number.

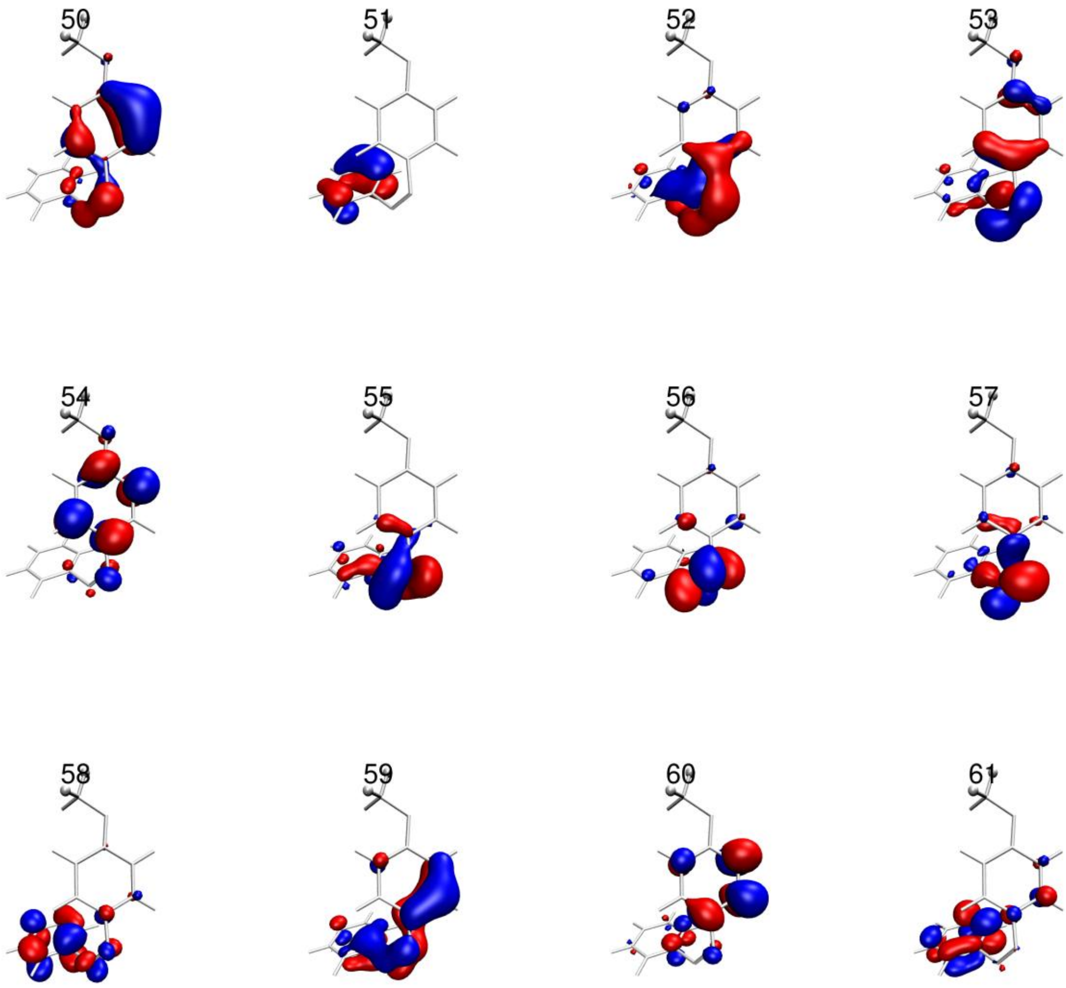

Figure S141. Active space of the Z-form of MeO-azobenzene at the CASSCF/ANO-RCC-VTZP//ωB97X-3c/SMD(toluene) level of theory. The digits represent the orbital number.

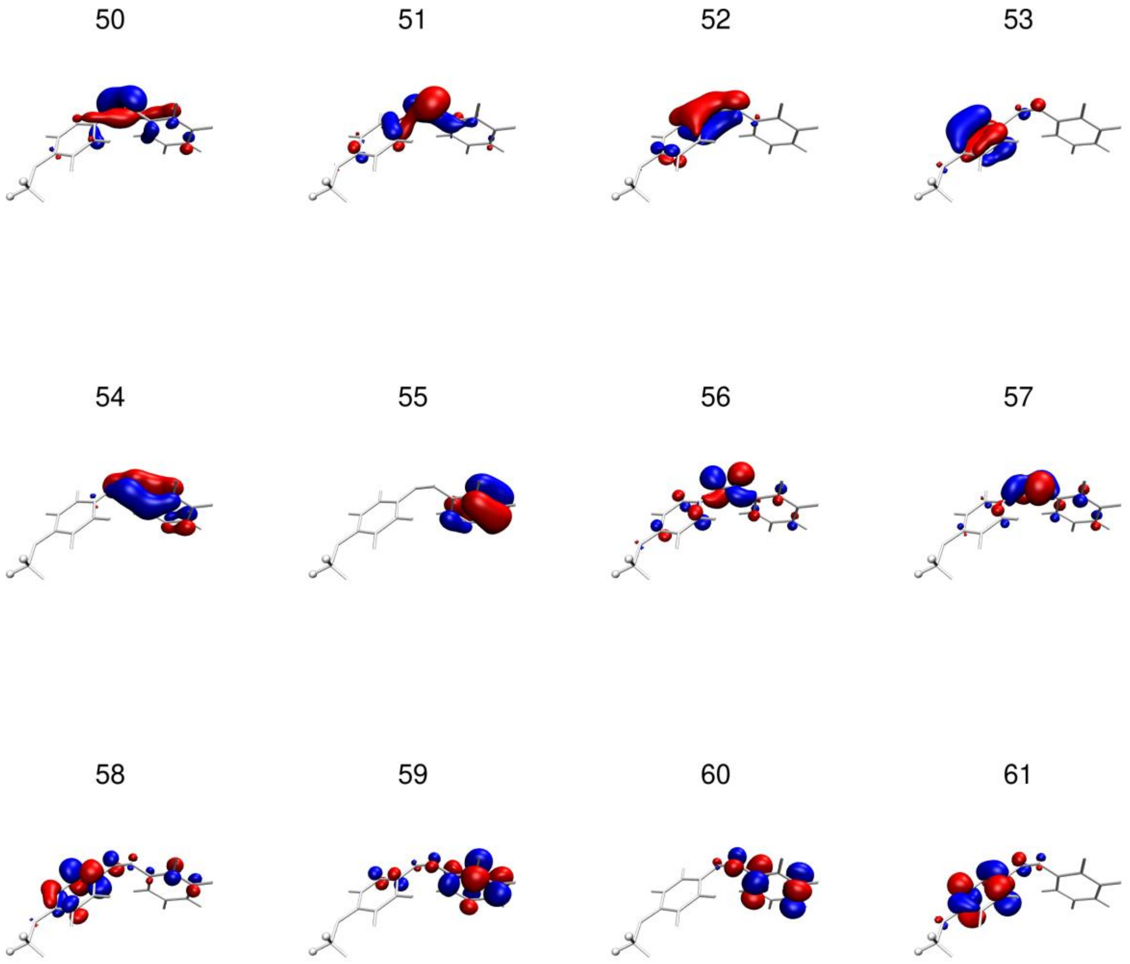

Figure S142. Active space of the T_1 minimum of MeO-azobenzene at the CASSCF/ANO-RCC-VTZP//ωB97X-3c/SMD(toluene) level of theory. The digits represent the orbital number.

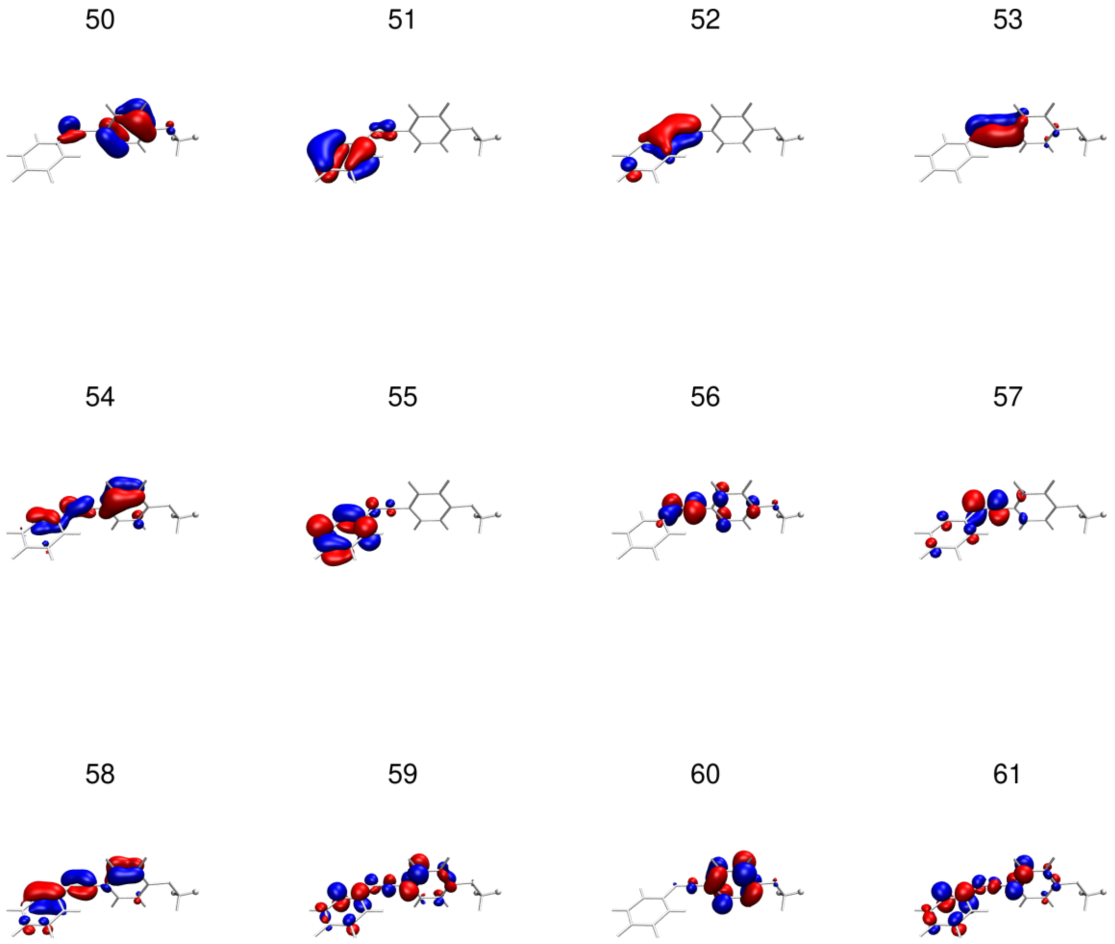

Figure S143. Active space of the TSInv1 of MeO-azobenzene at the CASSCF/ANO-RCC-VTZP//ωB97X-3c/SMD(toluene) level of theory. The digits represent the orbital number.

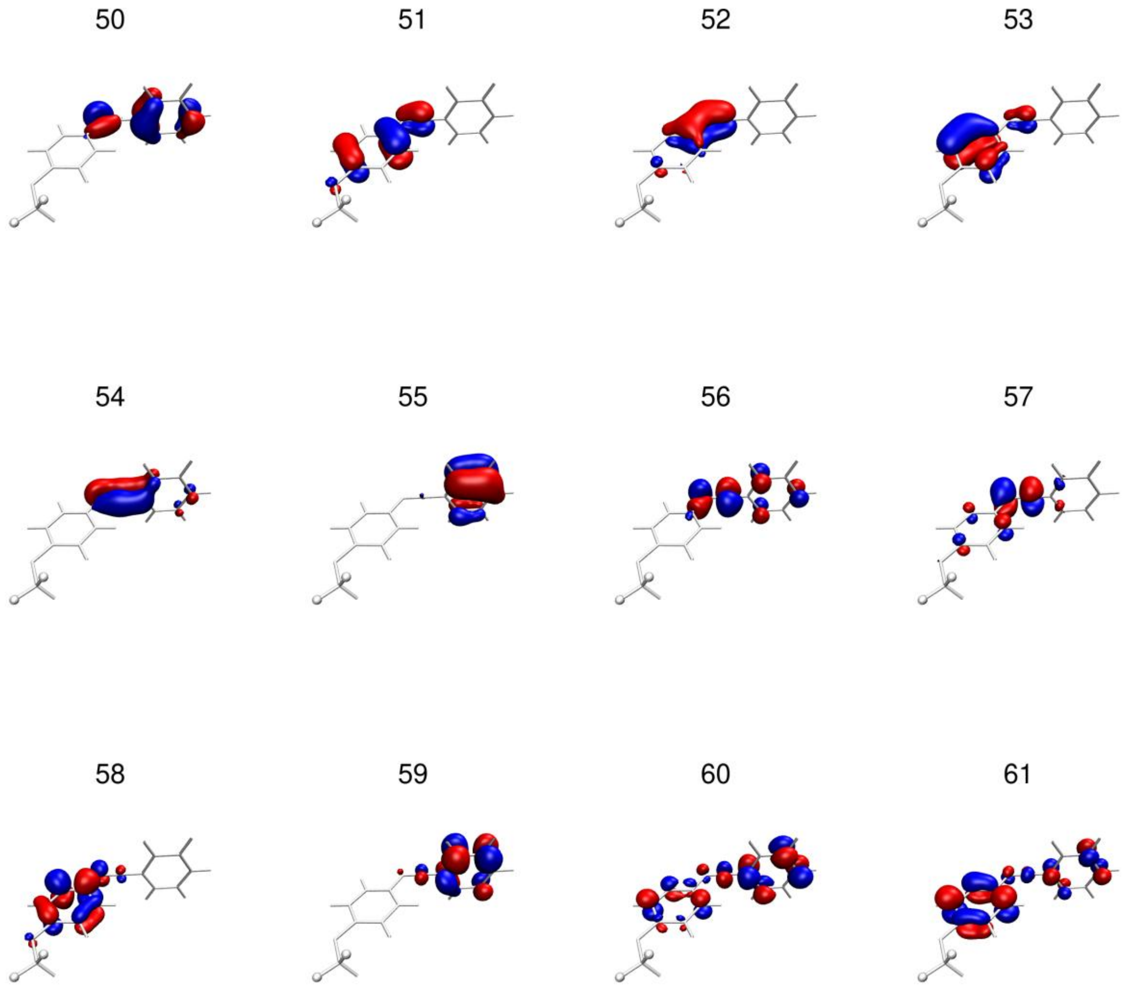

Figure S144. Active space of the TSInv2 of MeO-azobenzene at the CASSCF/ANO-RCC-VTZP//ωB97X-3c/SMD(toluene) level of theory. The digits represent the orbital number.

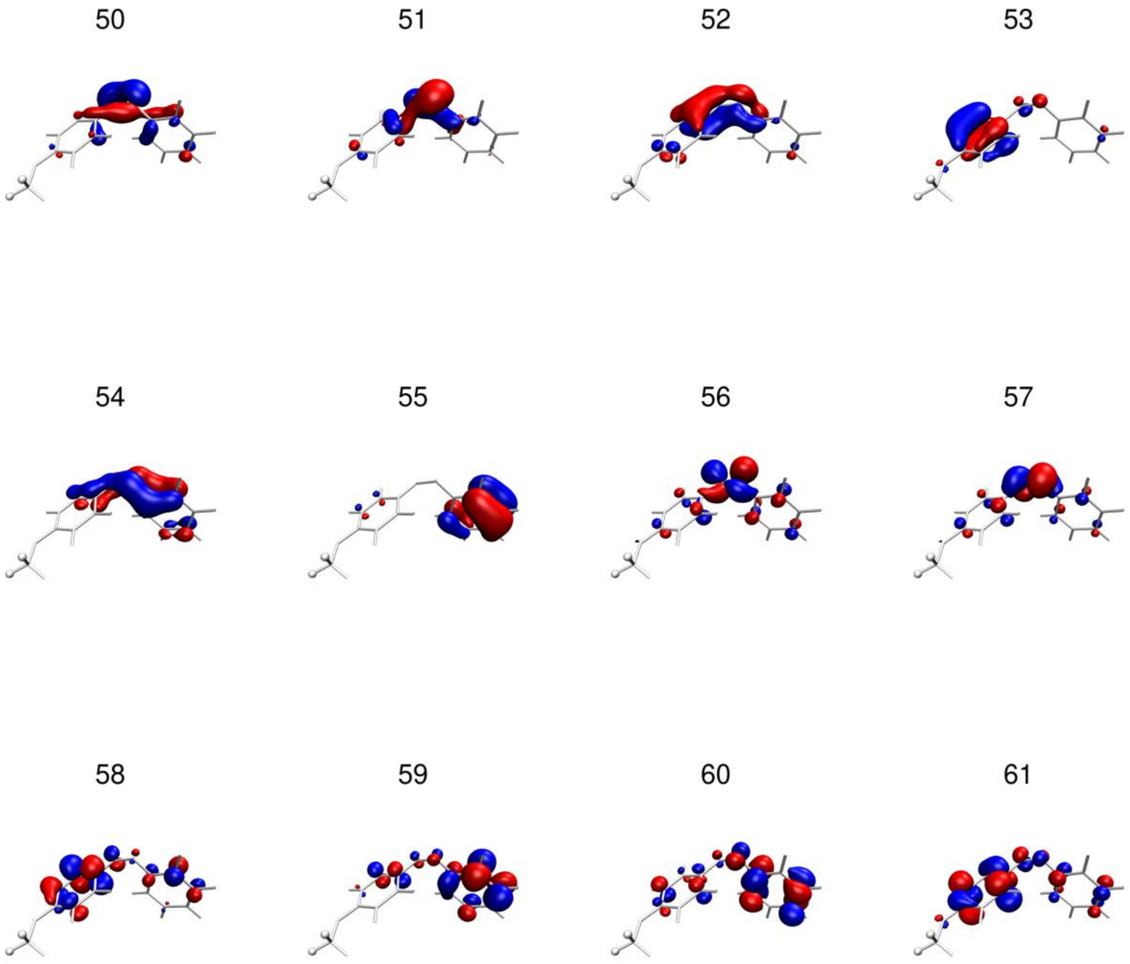

Figure S145. Active space of the TSRot of MeO-azobenzene at the CASSCF/ANO-RCC-VTZP//ωB97X-3c/SMD(toluene) level of theory. The digits represent the orbital number.

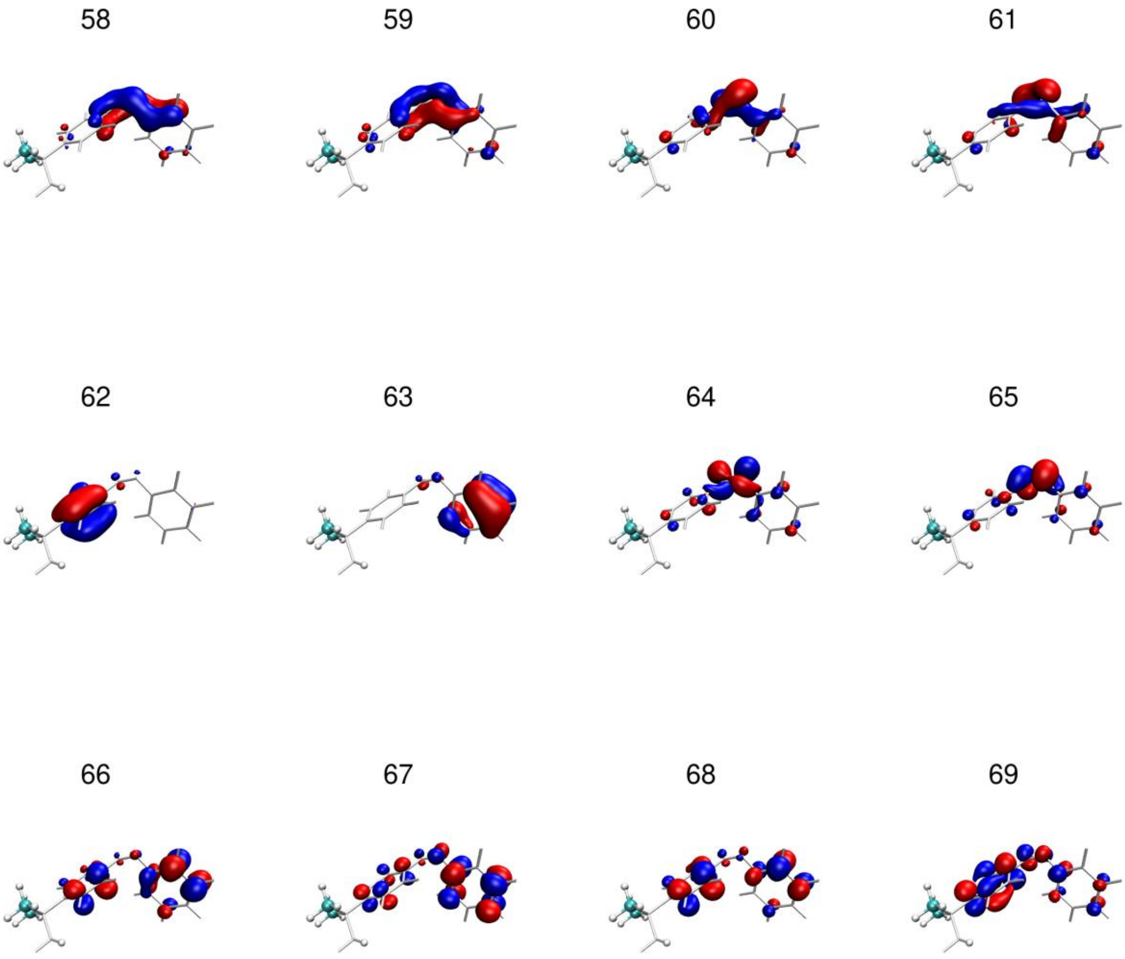

Figure S146. Active space of the MECP1 of *t*Bu-azobenzene at the CASSCF/ANO-RCC-VTZP//ωB97X-3c/SMD(toluene) level of theory. The digits represent the orbital number.

58

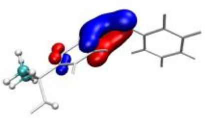

59

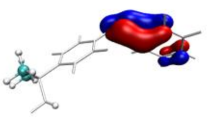

60

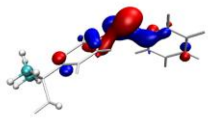

61

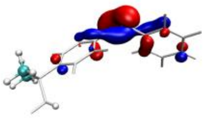

62

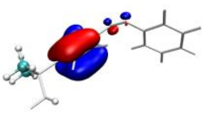

63

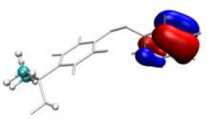

64

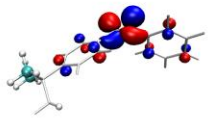

65

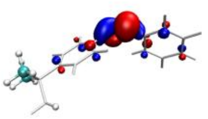

66

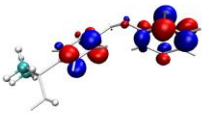

67

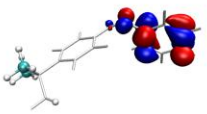

68

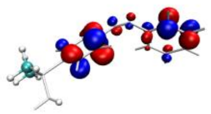

69

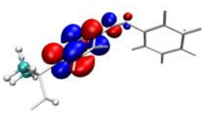

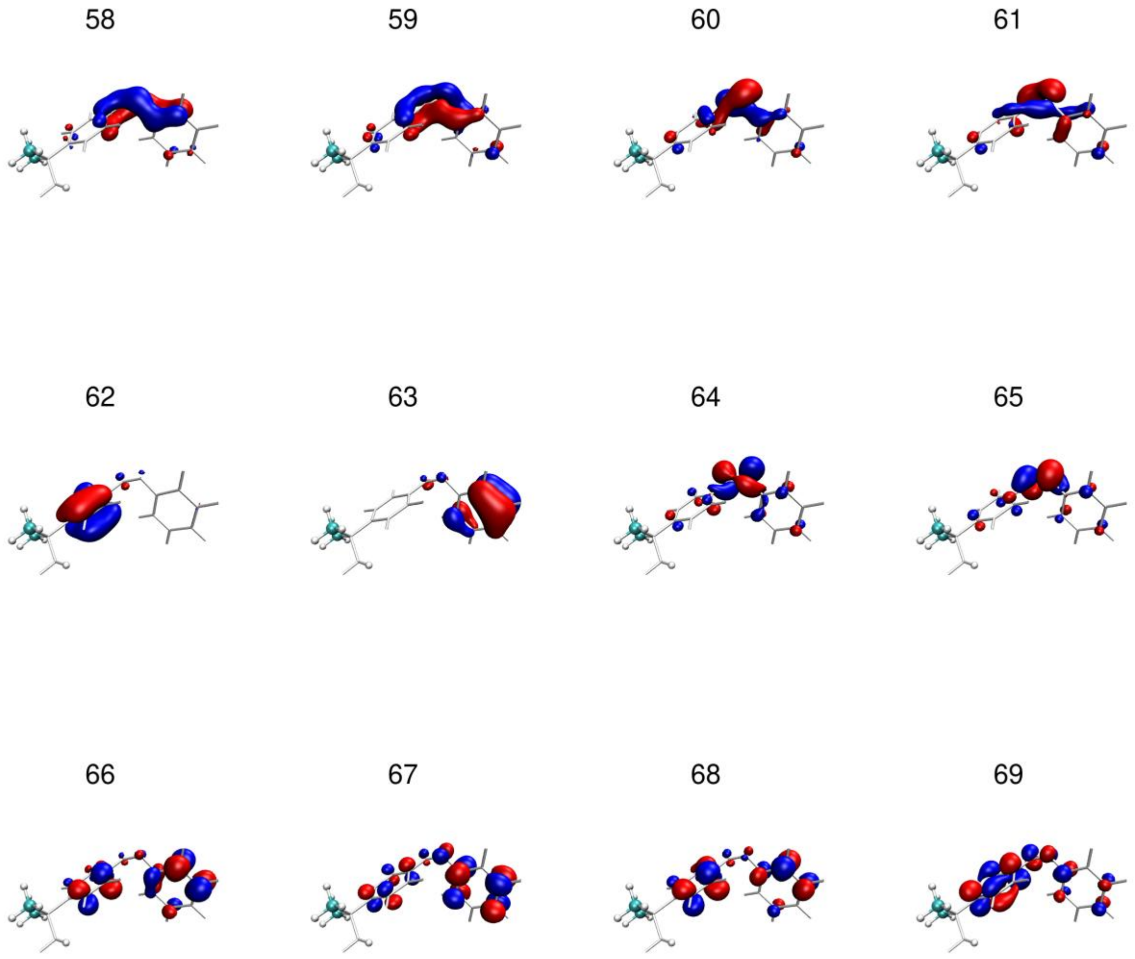

Figure S147. Active space of the MECP2 of *t*Bu-azobenzene at the CASSCF/ANO-RCC-VTZP//ωB97X-3c/SMD(toluene) level of theory. The digits represent the orbital number.

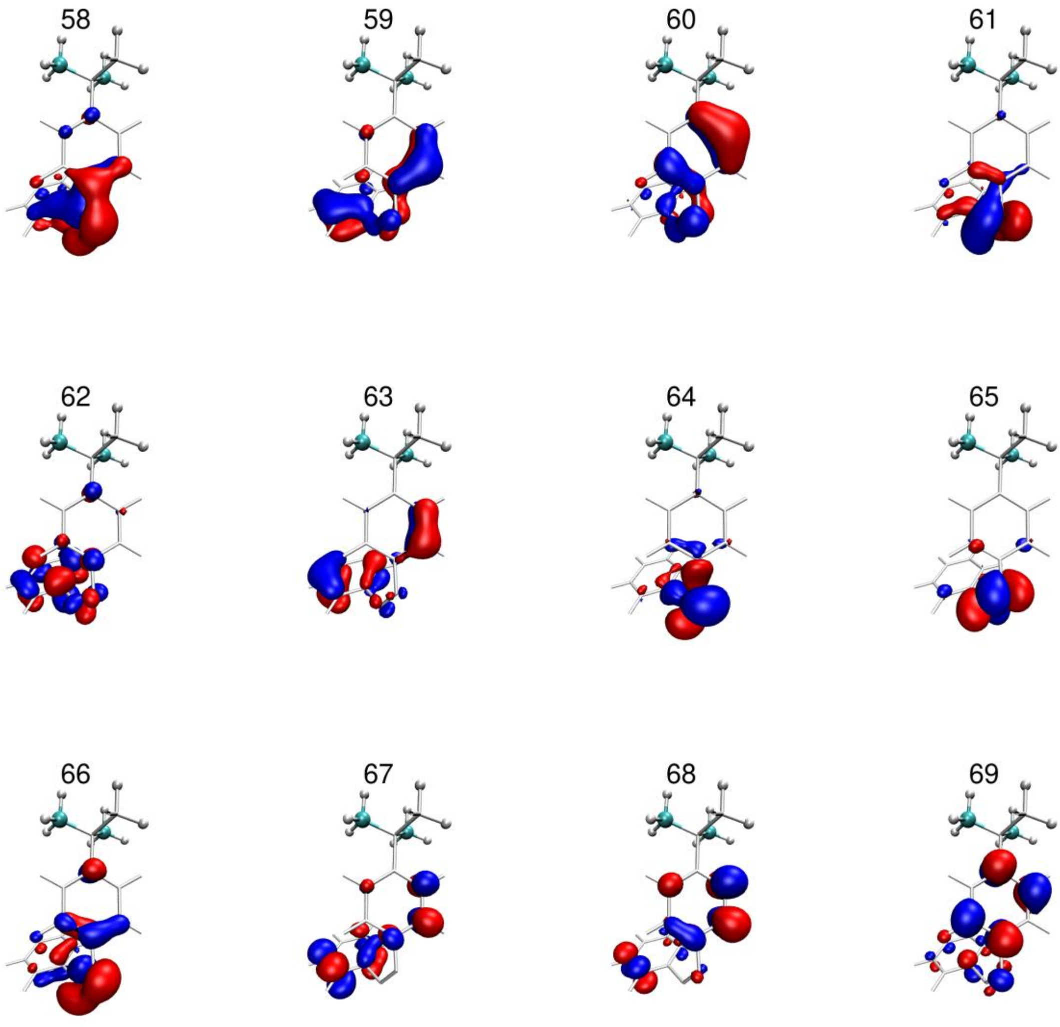

Figure S148. Active space of the Z-form of *t*Bu-azobenzene at the CASSCF/ANO-RCC-VTZP//ωB97X-3c/SMD(toluene) level of theory. The digits represent the orbital number.

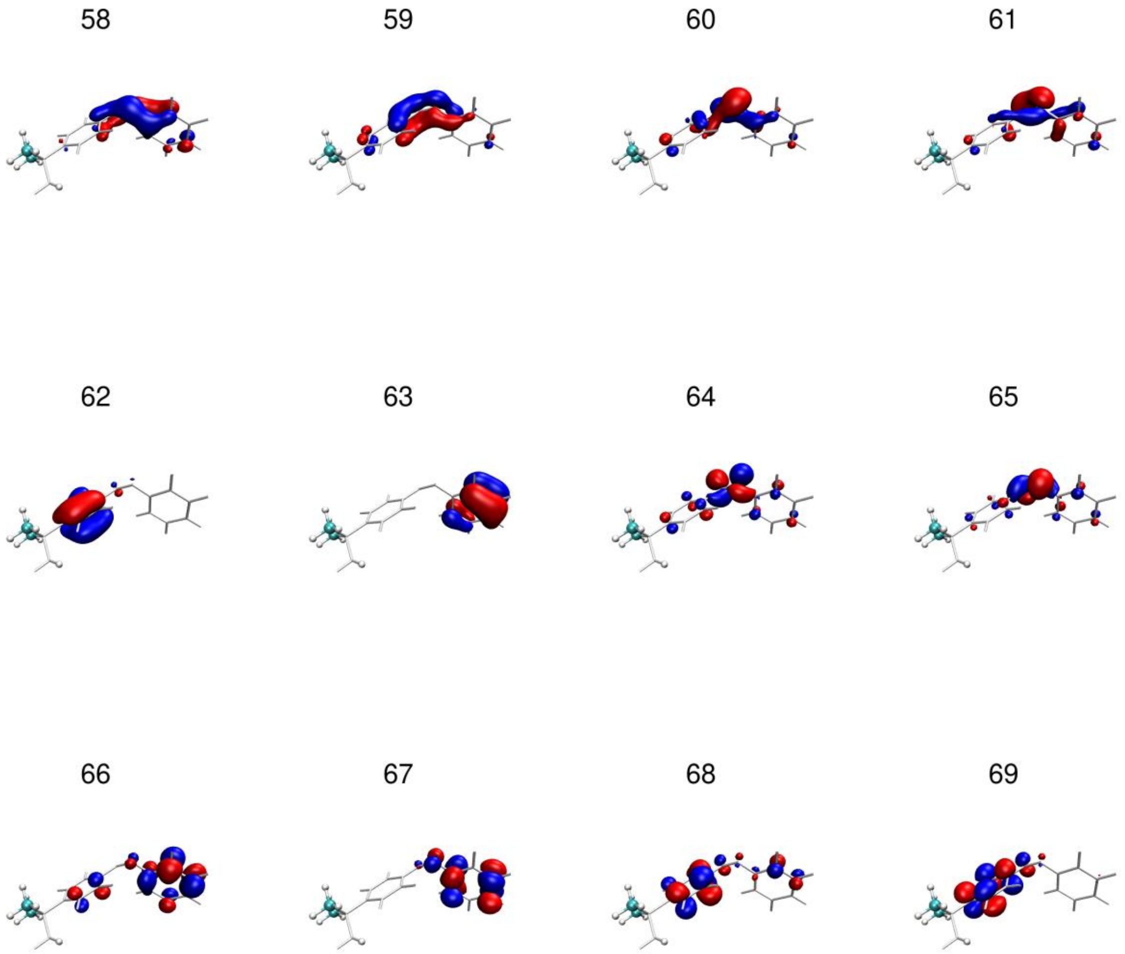

Figure S149. Active space of the T_1 minimum of *t*Bu-azobenzene at the CASSCF/ANO-RCC-VTZP//ωB97X-3c/SMD(toluene) level of theory. The digits represent the orbital number.

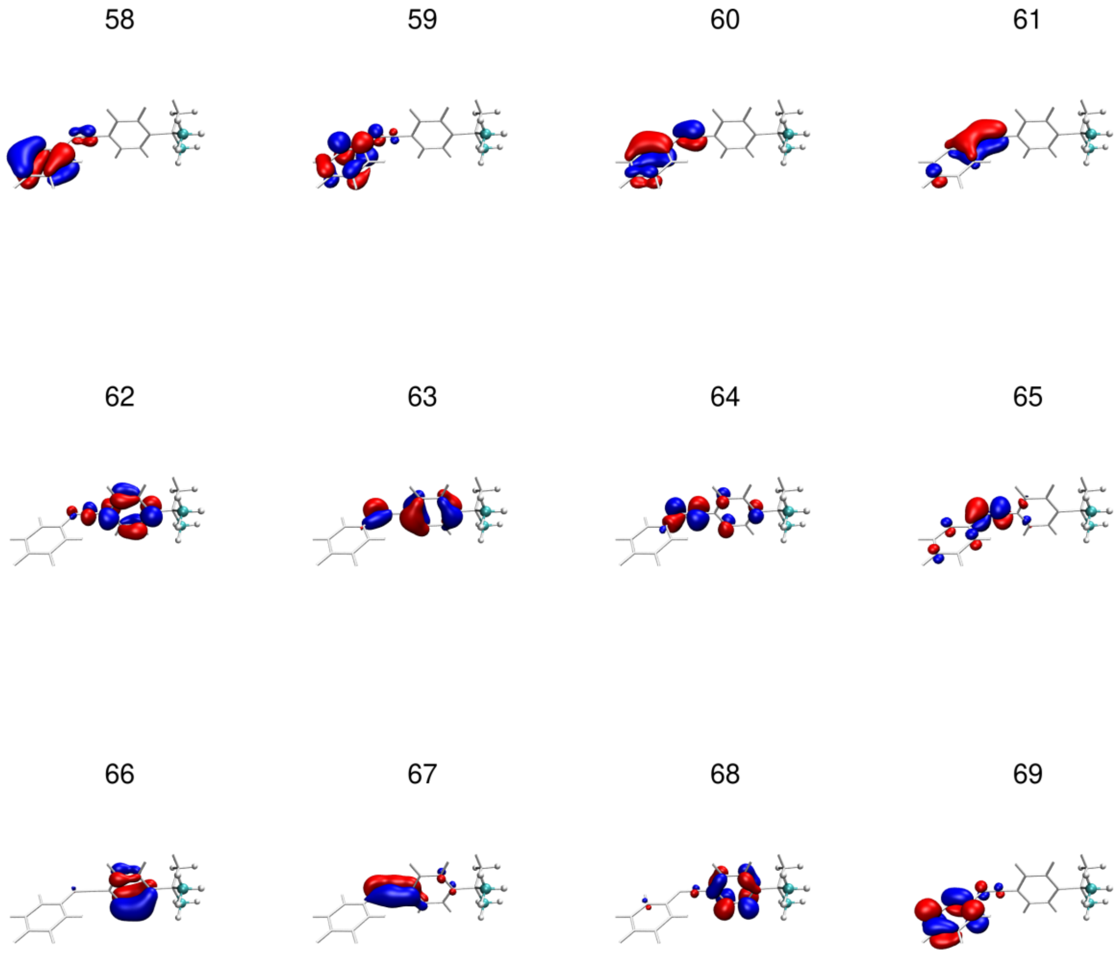

Figure S150. Active space of the TSInv1 of *t*Bu-azobenzene at the CASSCF/ANO-RCC-VTZP//ωB97X-3c/SMD(toluene) level of theory. The digits represent the orbital number.

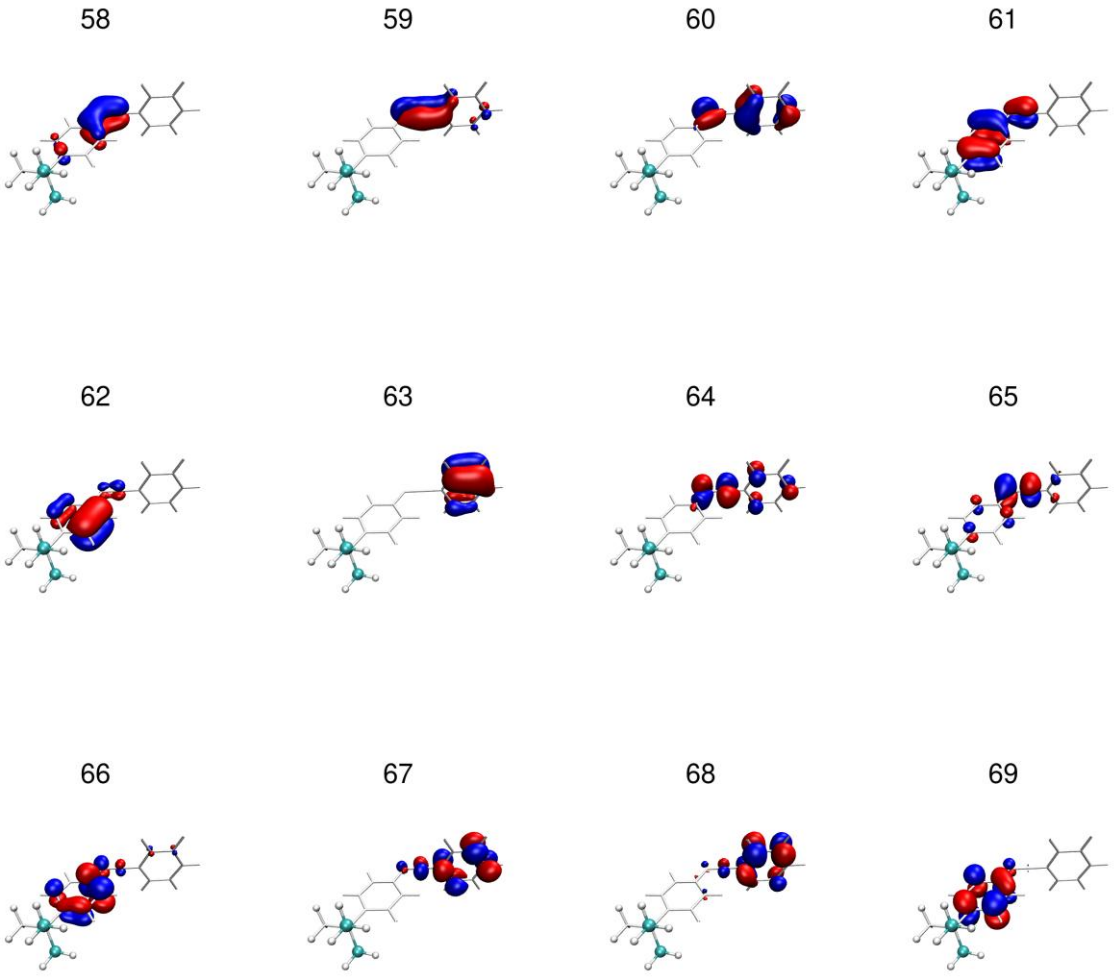

Figure S151. Active space of the TSInv2 of *t*Bu-azobenzene at the CASSCF/ANO-RCC-VTZP//ωB97X-3c/SMD(toluene) level of theory. The digits represent the orbital number.

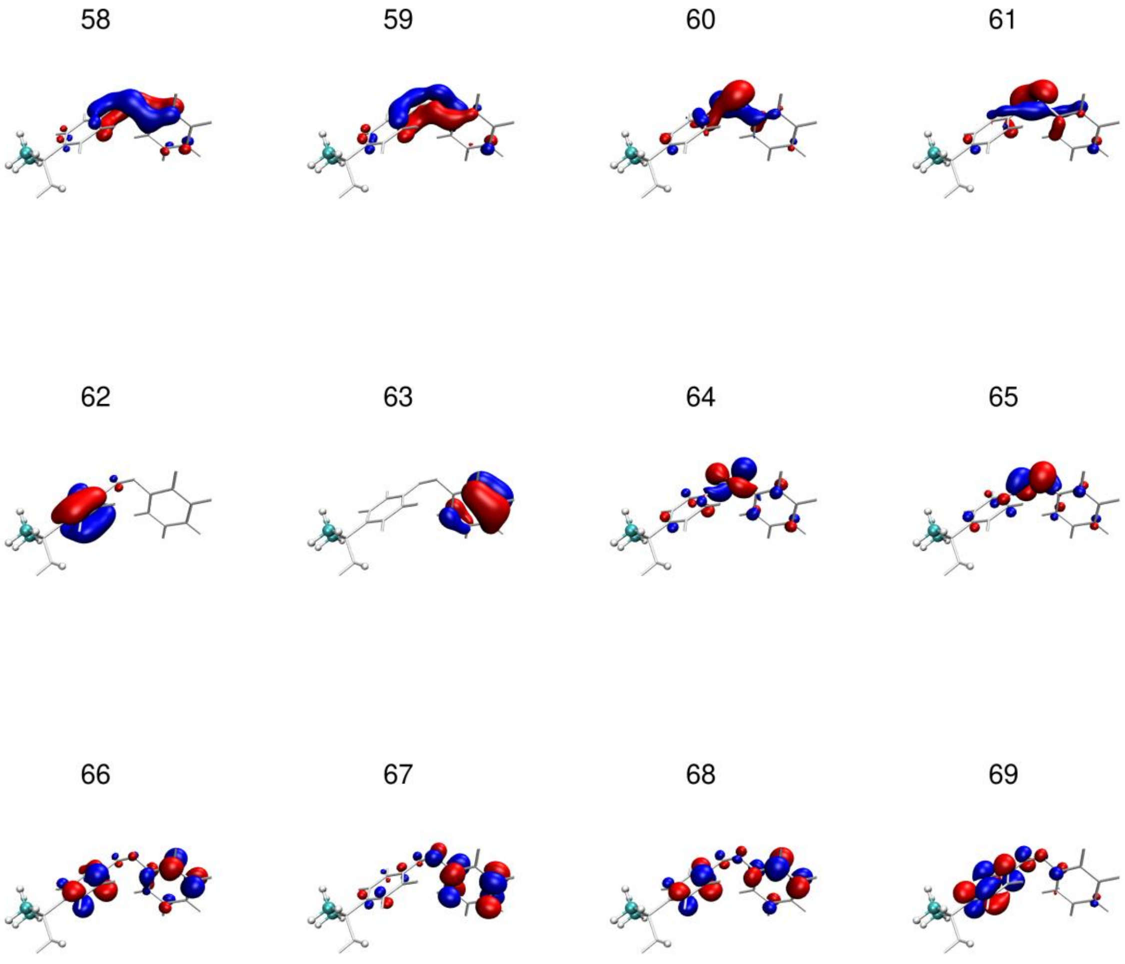

Figure S152. Active space of the TSRot of *t*Bu-azobenzene at the CASSCF/ANO-RCC-VTZP//ωB97X-3c/SMD(toluene) level of theory. The digits represent the orbital number.

9 References

[1] H. Lv, R. D. Laishram, Y. Yang, J. Li, D. Xu, Y. Zhan, Y. Luo, Z. Su, S. More, B. Fan, "TEMPO catalyzed oxidative dehydrogenation of hydrazobenzenes to azobenzenes" *Org. Biomol. Chem.* **2020**, *18*, 3471–3474.

[2] H. Lv, R. D. Laishram, J. Li, Y. Zhou, D. Xu, S. More, Y. Dai, B. Fan, "Photocatalyzed oxidative dehydrogenation of hydrazobenzenes to azobenzenes" *Green Chem.* **2019**, *21*, 4055–4061.

[3] Y. Shima, J. Matsuo, "Formal [4+2] cycloaddition of 3-ethoxycyclobutanones with azo compounds" *Tetrahedron Lett.* **2016**, *57*, 4066–4069.

[4] Corwin. Hansch, A. Leo, R. W. Taft, "A survey of Hammett substituent constants and resonance and field parameters" *Chem. Rev.* **1991**, *91*, 165–195.

[5] X. Creary, P. S. Engel, N. Kavaluskas, L. Pan, A. Wolf, "Methylenecyclopropane Rearrangement as a Probe for Free Radical Substituent Effects. σ• Values for Potent Radical-Stabilizing Nitrogen-Containing Substituents" *J. Org. Chem.* **1999**, *64*, 5634–5643.

[6] X. Jiang, G. Ji, "A self-consistent and cross-checked scale of spin-delocalization substituent constants, the .sigma.JJ.bul. scale" *J. Org. Chem.* **1992**, *57*, 6051–6056.

[7] T. H. Fisher, A. W. Meierhoefer, "Substituent effects in free-radical reactions. A study of 4-substituted 3-cyanobenzyl free radicals" *J. Org. Chem.* **1978**, *43*, 224–228.

[8] T. H. Fisher, A. W. Meierhoefer, "A kinetic study of the N-bromosuccinimide bromination of some 4-substituted 3-cyanotoluenes" *J. Org. Chem.* **1978**, *43*, 220–224.

[9] H. G. Viehe, Z. Janousek, R. Merényi, Eds. , *Substituent Effects in Radical Chemistry*, Springer Netherlands, Dordrecht, **1986**.

[10] O. Exner in *Prog. Phys. Org. Chem.*, John Wiley & Sons, Ltd, **1973**, pp. 411–482.

[11] F. Neese, "Software Update: The ORCA Program System—Version 6.0" *WIREs Comput. Mol. Sci.* **2025**, *15*, e70019.

[12] F. Neese, F. Wennmohs, U. Becker, C. Riplinger, "The ORCA quantum chemistry program package" *J. Chem. Phys.* **2020**, *152*, 224108.

[13] C. Adamo, V. Barone, "Toward reliable density functional methods without adjustable parameters: The PBE0 model" *J. Chem. Phys.* **1999**, *110*, 6158–6170.

[14] F. Weigend, R. Ahlrichs, "Balanced basis sets of split valence, triple zeta valence and quadruple zeta valence quality for H to Rn: Design and assessment of accuracy" *Phys. Chem. Chem. Phys.* **2005**, *7*, 3297–3305.

[15] F. Weigend, "Accurate Coulomb-fitting basis sets for H to Rn" *Phys. Chem. Chem. Phys.* **2006**, *8*, 1057–1065.

[16] J. G. Brandenburg, C. Bannwarth, A. Hansen, S. Grimme, "B97-3c: A revised low-cost variant of the B97-D density functional method" *J. Chem. Phys.* **2018**, *148*, 064104.

[17] M. Müller, A. Hansen, S. Grimme, "ωB97X-3c: A composite range-separated hybrid DFT method with a molecule-optimized polarized valence double-ζ basis set" *J. Chem. Phys.* **2023**, *158*, 014103.

[18] S. Grimme, A. Hansen, S. Ehlert, J.-M. Mewes, "r2SCAN-3c: A 'Swiss army knife' composite electronic-structure method" *J. Chem. Phys.* **2021**, *154*, 064103.

[19] B. O. Roos, R. Lindh, P.-Å. Malmqvist, V. Veryazov, P.-O. Widmark, "New Relativistic ANO Basis Sets for Transition Metal Atoms" *J. Phys. Chem. A* **2005**, *109*, 6575–6579.

[20] B. O. Roos, P. R. Taylor, P. E. M. Sigbahn, "A complete active space SCF method (CASSCF) using a density matrix formulated super-CI approach" *Chem. Phys.* **1980**, *48*, 157–173.

[21] G. Li Manni, I. Fdez. Galván, A. Alavi, F. Aleotti, F. Aquilante, J. Autschbach, D. Avagliano, A. Baiardi, J. J. Bao, S. Battaglia, L. Birnoschi, A. Blanco-González, S. I. Bokarev, R. Broer, R. Cacciari, P. B. Calio, R. K. Carlson, R. Carvalho Couto, L. Cerdán,

L. F. Chibotaru, N. F. Chilton, J. R. Church, I. Conti, S. Coriani, J. Cuéllar-Zuquin, R. E. Daoud, N. Dattani, P. Decleva, C. De Graaf, M. G. Delcey, L. De Vico, W. Dobrautz, S. S. Dong, R. Feng, N. Ferré, M. Filatov(Gulak), L. Gagliardi, M. Garavelli, L. González, Y. Guan, M. Guo, M. R. Hennefarth, M. R. Hermes, C. E. Hoyer, M. Huix-Rotllant, V. K. Jaiswal, A. Kaiser, D. S. Kaliakin, M. Khamesian, D. S. King, V. Kochetov, M. Krośnicki, A. A. Kumaar, E. D. Larsson, S. Lehtola, M.-B. Lepetit, H. Lischka, P. López Ríos, M. Lundberg, D. Ma, S. Mai, P. Marquetand, I. C. D. Merritt, F. Montorsi, M. Mörchen, A. Nenov, V. H. A. Nguyen, Y. Nishimoto, M. S. Oakley, M. Olivucci, M. Oppel, D. Padula, R. Pandharkar, Q. M. Phung, F. Plasser, G. Raggi, E. Rebolini, M. Reiher, I. Rivalta, D. Roca-Sanjuán, T. Romig, A. A. Safari, A. Sánchez-Mansilla, A. M. Sand, I. Schapiro, T. R. Scott, J. Segarra-Martí, F. Segatta, D.-C. Sergentu, P. Sharma, R. Shepard, Y. Shu, J. K. Staab, T. P. Straatsma, L. K. Sørensen, B. N. C. Tenorio, D. G. Truhlar, L. Ungur, M. Vacher, V. Veryazov, T. A. Voß, O. Weser, D. Wu, X. Yang, D. Yarkony, C. Zhou, J. P. Zobel, R. Lindh, "The OpenMolcas *Web* : A Community-Driven Approach to Advancing Computational Chemistry" *J. Chem. Theory Comput.* **2023**, *19*, 6933–6991.

[22] W. Park, K. Komarov, S. Lee, C. H. Choi, "Mixed-Reference Spin-Flip Time-Dependent Density Functional Theory: Multireference Advantages with the Practicality of Linear Response Theory" *J. Phys. Chem. Lett.* **2023**, *14*, 8896–8908.

[23] V. Mironov, K. Komarov, J. Li, I. Gerasimov, H. Nakata, M. Mazaherifar, K. Ishimura, W. Park, A. Lashkaripour, M. Oh, M. Huix-Rotllant, S. Lee, C. H. Choi, "OpenQP: A Quantum Chemical Platform Featuring MRSF-TDDFT with an Emphasis on Open-Source Ecosystem" *J. Chem. Theory Comput.* **2024**, *20*, 9464–9477.

[24] A. D. Becke, "A new mixing of Hartree–Fock and local density-functional theories" *J. Chem. Phys.* **1993**, *98*, 1372–1377.

[25] A. D. Becke, "Density-functional exchange-energy approximation with correct asymptotic behavior" *Phys. Rev. A* **1988**, *38*, 3098–3100.

[26] B. Miehlich, A. Savin, H. Stoll, H. Preuss, "Results obtained with the correlation energy density functionals of becke and Lee, Yang and Parr" *Chem. Phys. Lett.* **1989**, *157*, 200–206.

[27] C. Lee, W. Yang, R. G. Parr, "Development of the Colle-Salvetti correlation-energy formula into a functional of the electron density" *Phys. Rev. B* **1988**, *37*, 785–789.

[28] E. Caldeweyher, S. Ehlert, A. Hansen, H. Neugebauer, S. Spicher, C. Bannwarth, S. Grimme, "A generally applicable atomic-charge dependent London dispersion correction" *J. Chem. Phys.* **2019**, *150*, 154122.

[29] E. Caldeweyher, J.-M. Mewes, S. Ehlert, S. Grimme, "Extension and evaluation of the D4 London-dispersion model for periodic systems" *Phys. Chem. Chem. Phys.* **2020**, *22*, 8499–8512.

[30] J.-D. Chai, M. Head-Gordon, "Systematic optimization of long-range corrected hybrid density functionals" *J. Chem. Phys.* **2008**, *128*, 084106.

[31] D. Casanova, A. I. Krylov, "Spin-flip methods in quantum chemistry" *Phys. Chem. Chem. Phys.* **2020**, *22*, 4326–4342.

[32] A. V. Marenich, C. J. Cramer, D. G. Truhlar, "Universal Solvation Model Based on Solute Electron Density and on a Continuum Model of the Solvent Defined by the Bulk Dielectric Constant and Atomic Surface Tensions" *J. Phys. Chem. B* **2009**, *113*, 6378–6396.

[33], "ExtOptORCA-OpenQP," can be found under https://github.com/CrespiLab/ExtOptORCA-OpenQP, **2025**.

[34] J. Steinmetzer, S. Kupfer, S. Gräfe, "pysisyphus: Exploring potential energy surfaces in ground and excited states" *Int. J. Quantum Chem.* **2021**, *121*, e26390.

[35] M. Reimann, E. Teichmann, S. Hecht, M. Kaupp, "Solving the Azobenzene Entropy Puzzle: Direct Evidence for Multi-State Reactivity" *J. Phys. Chem. Lett.* **2022**, *13*, 10882–10888.
[36] F. A. Martins, R. V. Viesser, J. V. Schober, R. Herges, J. I. Wu, "Triplet Spin Delocalization and Temperature Dependence for Adiabatic and Non-Adiabatic *Z* – *E* Isomerization Pathways in Azoarenes" *J. Am. Chem. Soc.* **2025**, jacs.5c09852.
[37] M. Saitow, F. Neese, "Accurate spin-densities based on the domain-based local pair-natural orbital coupled-cluster theory" *J. Chem. Phys.* **2018**, *149*, 034104.
[38] M. Saitow, U. Becker, C. Riplinger, E. F. Valeev, F. Neese, "A new near-linear scaling, efficient and accurate, open-shell domain-based local pair natural orbital coupled cluster singles and doubles theory" *J. Chem. Phys.* **2017**, *146*, 164105.
[39] G. Bistoni, C. Riplinger, Y. Minenkov, L. Cavallo, A. A. Auer, F. Neese, "Treating Subvalence Correlation Effects in Domain Based Pair Natural Orbital Coupled Cluster Calculations: An Out-of-the-Box Approach" *J. Chem. Theory Comput.* **2017**, *13*, 3220–3227.
[40] C. Riplinger, P. Pinski, U. Becker, E. F. Valeev, F. Neese, "Sparse maps—A systematic infrastructure for reduced-scaling electronic structure methods. II. Linear scaling domain based pair natural orbital coupled cluster theory" *J. Chem. Phys.* **2016**, *144*, 024109.
[41] C. Riplinger, B. Sandhoefer, A. Hansen, F. Neese, "Natural triple excitations in local coupled cluster calculations with pair natural orbitals" *J. Chem. Phys.* **2013**, *139*, 134101.
[42] C. Riplinger, F. Neese, "An efficient and near linear scaling pair natural orbital based local coupled cluster method" *J. Chem. Phys.* **2013**, *138*, 034106.
[43] F. Neese, F. Wennmohs, A. Hansen, "Efficient and accurate local approximations to coupled-electron pair approaches: An attempt to revive the pair natural orbital method" *J. Chem. Phys.* **2009**, *130*, 114108.
[44] A. Hansen, D. G. Liakos, F. Neese, "Efficient and accurate local single reference correlation methods for high-spin open-shell molecules using pair natural orbitals" *J. Chem. Phys.* **2011**, *135*, 214102.
[45] F. Neese, A. Hansen, D. G. Liakos, "Efficient and accurate approximations to the local coupled cluster singles doubles method using a truncated pair natural orbital basis" *J. Chem. Phys.* **2009**, *131*, 064103.
[46] A. Cembran, F. Bernardi, M. Garavelli, L. Gagliardi, G. Orlandi, "On the Mechanism of the cis−trans Isomerization in the Lowest Electronic States of Azobenzene: S_0 , S_1 , and T_1" *J. Am. Chem. Soc.* **2004**, *126*, 3234–3243.
[47] S. Axelrod, E. Shakhnovich, R. Gómez-Bombarelli, "Thermal Half-Lives of Azobenzene Derivatives: Virtual Screening Based on Intersystem Crossing Using a Machine Learning Potential" *ACS Cent. Sci.* **2023**, *9*, 166–176.
[48] L. Hegedüsová, R. KuteI', M. Medved', L. F. Pašteka, M. Cigáň, Š. Budzák, "Thermal isomerization of phenylazoindoles: Inversion or rotation? That is the question" *Int. J. Quantum Chem.* **2023**, *123*, e27120.
[49] N. K. Singer, K. Schlögl, J. P. Zobel, M. D. Mihovilovic, L. González, "Singlet and Triplet Pathways Determine the Thermal *Z* / *E* Isomerization of an Arylazopyrazole-Based Photoswitch" *J. Phys. Chem. Lett.* **2023**, *14*, 8956–8961.
[50] P. Å. Malmqvist, "Calculation of transition density matrices by nonunitary orbital transformations" *Int. J. Quantum Chem.* **1986**, *30*, 479–494.
[51] S. Grimme, "Supramolecular Binding Thermodynamics by Dispersion-Corrected Density Functional Theory" *Chem. – Eur. J.* **2012**, *18*, 9955–9964.
[52] K. Schlögl, N. K. Singer, D. Dreier, H. Kalaus, R. C. O. Conceição, M. D. Mihovilovic, L. González, "Mechanistic Insight into Para-Substituent Control of Thermal Half-Lives in Arylazopyrazole Photoswitches" *Angew. Chem. Int. Ed.* **2025**, *64*, e202514433.
